# Gene silencing pathways found in the green alga *Volvox carteri* reveal insights into evolution and origins of small RNA systems in plants

**DOI:** 10.1186/s12864-016-3202-4

**Published:** 2016-11-02

**Authors:** Anne Dueck, Maurits Evers, Stefan R. Henz, Katharina Unger, Norbert Eichner, Rainer Merkl, Eugene Berezikov, Julia C. Engelmann, Detlef Weigel, Stephan Wenzl, Gunter Meister

**Affiliations:** 1 Biochemistry Center Regensburg (BZR), Laboratory for RNA Biology, University of Regensburg, Universitätsstrasse 31, 93053 Regensburg, Germany; 2 Department of Statistical Bioinformatics, University of Regensburg, Universitätsstrasse 31, 93053 Regensburg, Germany; 3 Present address: The John Curtin School of Medical Research, The Australian National University, Canberra, Australia; 4 Department of Molecular Biology, Max Planck Institute for Developmental Biology, 72076 Tübingen, Germany; 5 Present address: Intomics A/S, Diplomvej 377, 2800 Lyngby, Denmark; 6 Biochemistry Center Regensburg (BZR), Biochemistry II, University of Regensburg, Universitätsstrasse 31, 93053 Regensburg, Germany; 7 European Research Institute for the Biology of Ageing, University of Groningen, University Medical Center Groningen, Antonius Deusinglaan 1, 9713AV Groningen, The Netherlands

**Keywords:** microRNAs, Gene silencing, Transposons, Small RNAs, Evolution, Argonaute, *Volvox carteri*

## Abstract

**Background:**

*Volvox carteri (V. carteri)* is a multicellular green alga used as model system for the evolution of multicellularity. So far, the contribution of small RNA pathways to these phenomena is not understood. Thus, we have sequenced *V. carteri* Argonaute 3 (VcAGO3)-associated small RNAs from different developmental stages.

**Results:**

Using this functional approach, we define the *Volvox* microRNA (miRNA) repertoire and show that miRNAs are not conserved in the closely related unicellular alga *Chlamydomonas reinhardtii*. Furthermore, we find that miRNAs are differentially expressed during different life stages of *V. carteri*. In addition to miRNAs, transposon-associated small RNAs or phased siRNA loci, which are common in higher land plants, are highly abundant in *Volvox* as well. Transposons not only give rise to miRNAs and other small RNAs, they are also targets of small RNAs.

**Conclusion:**

Our analyses reveal a surprisingly complex small RNA network in *Volvox* as elaborate as in higher land plants. At least the identified VcAGO3-associated miRNAs are not conserved in *C. reinhardtii* suggesting fast evolution of small RNA systems. Thus, distinct small RNAs may contribute to multicellularity and also division of labor in reproductive and somatic cells.

**Electronic supplementary material:**

The online version of this article (doi:10.1186/s12864-016-3202-4) contains supplementary material, which is available to authorized users.

## Background

Volvocine algae are a sub-group of green algae ranging from unicellular species such as *Chlamydomonas reinhardtii* (*C. reinhardtii*) to the multicellular genus *Volvox. V. carteri* is a freshwater alga with female and male cells that can reproduce both sexually and asexually and is composed of two different cell types [1]. Asexual forms are characterized by a spheroid that is composed of approx. 2000 somatic cells and encloses several reproductive cells. Each of these so called gonidia can undergo symmetric and asymmetric cell divisions to form a new colony of somatic cells and gonidia [2]. The sexual life cycle is started by a sex-inducing pheromone that can be secreted by male strains and is functional at very low concentrations [3]. Pheromone signaling induces the sexual differentiation program and therefore the generation of egg cells in female strains, while male strains form sperm packages that are subsequently released into the water [4]. Dimorphic sexes have evolved several times in plants, however their origins are unclear. The fact, that the sexually dimorphic and multicellular species *V. carteri* and the unicellular species *C. reinhardtii* had a common ancestor about 200 million years ago [5] makes *Volvox* an ideal system for studying the evolution of these processes [4].

Non-coding RNAs, including small RNAs, have been associated with processes that are crucial to eukaryotic evolution. Small RNAs are processed from longer precursor molecules to their mature forms that are subsequently bound by a member of the Argonaute (AGO) protein family [6]. Argonaute proteins are characterized by distinct domains that anchor both the 5′ and the 3′ end of the small RNA [7]. Some but not all Argonaute proteins are endonucleases and can cleave target RNAs that are complementary to the bound small RNAs [8, 9]. In many organisms, several Argonaute protein genes exist and especially in plants each AGO protein prefers and selects different small RNAs [10]. In land plants, several different pathways that make use of specialized small RNAs are known [10, 11]. For example, short interfering RNAs (siRNAs) are processed from transgene- or virus-derived double stranded (ds) RNAs and guide sequence-specific cleavage of complementary (foreign) RNAs. Furthermore, small RNAs can direct DNA methylation and subsequent gene silencing processes in a pathway known as RNA-dependent DNA methylation (RdDM). MicroRNAs (miRNAs) are processed from endogenous miRNA genes and guide repression of complementary target mRNA expression as a means of gene regulation. Unlike in animals, where miRNAs bind to partially complementary sequences, plant miRNAs mainly repress their target mRNAs by hybridizing to fully complementary sites followed by sequence-specific cleavage. So-called trans acting siRNAs (tasiRNAs) are plant-specific and also regulate gene expression. This pathway is initiated by a miRNA (most prominently miR-390), which guides cleavage of a target. The cleaved transcript is used as template by RNA-dependent RNA polymerases (RdRPs) to generate a long dsRNA, which is cleaved by DCL4 to produce many tasiRNAs thus amplifying the signal. These RNAs, in turn, silence the respective locus by transcript cleavage [12]. Recently, it has been shown in *A. thaliana* that a miRNA can also guide cleavage of transposon transcripts and, similarly, the cleaved transcript serves as template for dsRNA, which gives subsequently rise to siRNAs that repress transposon expression [13]. SiRNAs and miRNAs have been identified in *C. reinhardtii*, indicating that small RNA pathways have evolved at early stages of plant evolution [14, 15]. Given the importance of different small RNAs in processes closely associated with multicellular development in land plants, we speculated that they might have played a role in the transition from uni- to multicellularity in green algae. MiRNA candidates have been reported in *V. carteri*, but their function remains unknown [16].

Here, we have performed small RNA-Seq as well as RNA-Seq of mRNAs from different stages of *V. carteri* providing a global and comprehensive atlas of gene expression in somatic and reproductive cells. To identify functional small RNAs, we isolated VcAGO3 protein and cloned and deep sequenced associated small RNAs. Using this functional approach, we clearly define miRNAs as well as their specific expression under different conditions. Of note, the identified miRNAs are not conserved in *C. reinhardtii*. Furthermore, we find miRNAs as well as siRNAs originating from transposons such as *Jordan* or *Kangaroo*, for example. Strikingly, miRNAs are not only expressed from transposons but also seem to target such elements, suggesting miRNA-guided repression of transposable elements. Finally, we also provide evidence for loci that generate phased siRNAs similar to tasiRNAs found in *A. thaliana*. Although green algae and higher land plants including *A. thaliana* diverged very early and evolution proceeded independently, we provide evidence that many small RNA pathways found in land plants have also evolved in *V. carteri*.

## Results

### Transcriptome sequencing and annotation

We sought to characterize expression activity and dynamics not only of small RNAs, but also of mRNAs in *V. carteri*, since these are often modulated by small RNAs. When we started this study, only very little transcriptomic data had been available for *V. carteri* in general. We therefore cloned and sequenced RNAs under various experimental conditions (Additional file 1: Table S1). We included somatic cells and gonidia under vegetative growth conditions, somatic cells as well as gonidia after induction of sexual reproduction, along with somatic and egg cells from a female culture (fully differentiated). Using all single-end as well as paired-end sequencing data, a transcriptome assembly was generated in order to analyze mRNA transcripts in more detail. This comprehensive data set enabled us to analyze transcripts and their possible connection to small RNAs in better detail. All RNAseq data are available at the NCBI SRA under PRJNA266874 for further analysis.

### AGO-associated small RNAs of *V. carteri*

**Fig. 1:**
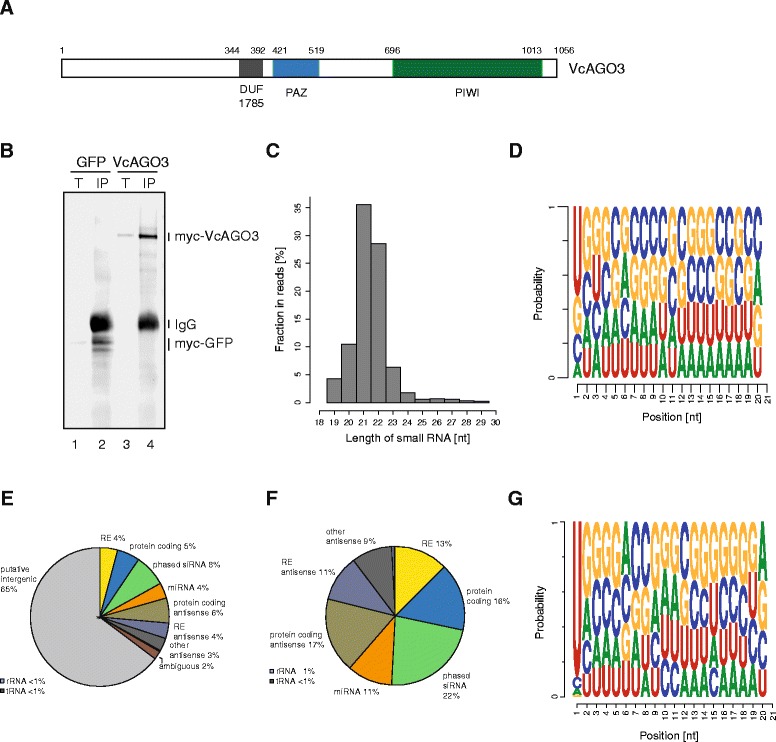
Characterization of small RNAs associated with VcAGO3. **a** Domain structure of *V. carteri* VcAGO3. VcAGO3 contains the canonical domains PAZ and Piwi and a domain of unknown function (DUF1785). Domain borders are indicated by the position of amino acid residues. **b** Western blot showing the expression and successful immunoprecipitation (IP) of over expressed, myc-tagged VcAGO3 and myc-tagged CrGFP (GFP, codon optimized for *C. reinhardtii* [49]). T = total lysate; IP = immunoprecipitation. **c** Size distribution of all small RNA reads associated with VcAGO3 measured by deep sequencing (after adapter trimming and quality filtering, see methods), nt = nucleotide. **d** Nucleotide frequencies of all small RNAs associated with VcAGO3 (**e**) Pie chart showing the categorization of all mapped reads. RE, repetitive elements. **f** Pie chart showing the distribution of all mapped, assigned reads. RE, repetitive elements. **g** Nucleotide frequencies of all miRNA reads

To identify functional small RNA pathways in *V. carteri*, we first examined available transcriptome data and our own RNA-Seq data for the presence of potential factors that are known to be involved in RNA silencing pathways and that are also known to be conserved proteins. We found a PAZ/PIWI domain-containing gene, typical for Argonaute genes, which we refer to as VcAGO3, since a similar transcript had already been annotated at this locus (XM_002952848) (Fig. 1a). Furthermore, we found genes for an additional AGO protein (although we could not amplify the complete transcript from cDNA for this gene so far), one Dicer, one RNA-dependent RNA Polymerase (RdRP) and a HEN1 homolog (Additional file 2: Figure S1), suggesting that several small RNA pathways might operate in *V. carteri* [10].

To identify functional small RNAs, we expressed myc-tagged VcAGO3 in *V. carteri* by gold particle-mediated plasmid delivery, isolated associated small RNAs by anti-myc immunoprecipitation and cloned and sequenced them (Fig. 1b). The length of the VcAGO3-associated RNAs peaks at 21 and 22 nt (Fig. 1c). While the GC content of the genome of *V. carteri* is about 60 %, the 5′ terminal nucleotide of the bound small RNAs is mainly uridine, suggesting that the VcAGO3 MID domain accommodates preferentially 5′ terminal uridines, similar to human Ago2 and *Arabidopsis* Ago1 [17, 18] (Fig. 1d). VcAGO3-associated small RNAs that map to the genome can be grouped into different functional categories including repetitive elements (RE), protein coding regions (sense and antisense), phased siRNAs or miRNAs (Fig. 1e and f). Since the transcriptome and RNA classes in general are only poorly annotated in *V. carteri*, the identity of the largest part of our library cannot be deduced yet. However, these sequences are bound by VcAGO3 and therefore are presumably functional (see also below and Fig. 4).

To predict miRNAs in *Volvox*, we utilized the novel miRNA identification tool miRA [19] using criteria adopted from the prediction of miRNAs in *C. reinhardtii* [14]. In total, 490 miRNAs were predicted when running miRA on the VcAGO3 data set as well as six libraries from different cells and life stages from *V. carteri* (Additional file 3: Table S2 and Additional file 4: Figure S2). These predicted miRNAs fall into 324 miRNA families (miRNAs with the same sequence, but different genomic location), the largest one comprising 48 members and 67 families had two or more members. The nucleotide preference for VcAGO3-bound miRNAs is even more pronounced towards a U at the 5′ end (Fig. 1g), indicating high specificity of our miRNA identification approach. In conclusion, we identified VcAGO3-associated potential miRNAs in different cell types and under different cellular conditions.

### Experimental validation of *V. carteri* miRNAs

**Fig. 2:**
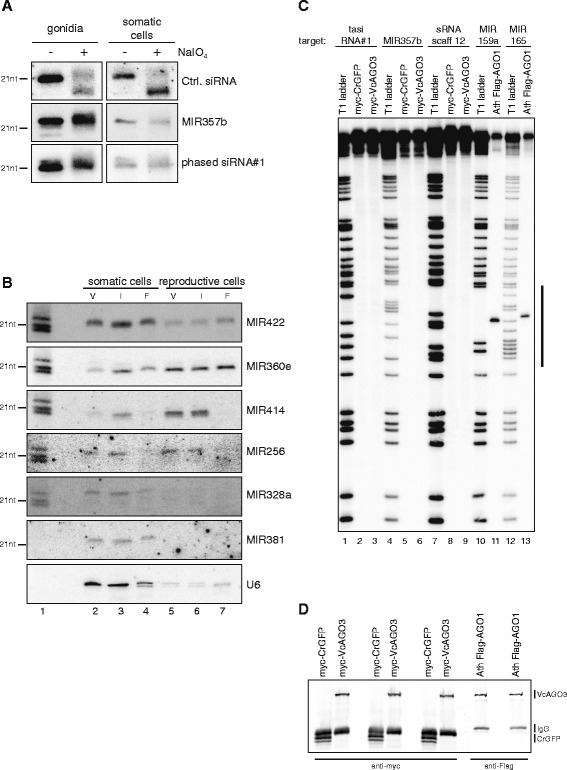
Validation of VcAGO3 and small RNA properties. **a** β-elimination experiment performed with total RNA from gonidia (reproductive cells) as well as from somatic cells showing that small RNAs in *V. carteri* are modified and do not shift upon periodate treatment. Control siRNA carries an unprotected 3′OH and therefore shifts towards a smaller size. **b** Total RNA from somatic cells and reproductive cells in different life stages (vegetative growth, V; induced to the female differentiation program, I; fully differentiated to female, F) was used to validate miRNA by RNA blot. U6 serves as a loading control. All blots shown originate from one membrane. See methods for details on stripping and re-probing of the membrane. **c** Cleavage assay with VcAGO3, *Arabidopsis thaliana* (Ath) AGO1 (positive control) and CrGFP (negative control). For VcAGO3, three different small RNAs were selected according to read abundance in the VcAGO3 library, for AtAGO1, two known and abundant miRNAs were chosen. Immunoprecipitated Ago proteins (loaded with their endogenously bound small RNAs) were incubated with 5′ radiolabeled target RNA carrying a perfectly complementary site to the respective small RNA. After incubation, RNA was extracted and run on a gel. To indicate the position of the cleavage product, each target RNA was digested with RNase T1 (cleaves after G’s) to serve as a ladder. The bar on the side of the gel indicates the site of the complementary sequence and putative cleavage site. **d** Western blot showing the successful immunoprecipitation of myc-VcAGO3, myc-CrGFP and Flag-AthAGO1 used for the cleavage experiment in (**c**). Western blot was performed with part of the immunoprecipitation reaction used for the cleavage assay. Black bars on the side indicate the location of the bands

A general feature of small RNAs or miRNAs in particular in plants is a 2′O-methylation at the 3′ end [20]. To test whether our miRNA candidates contain such characteristic modifications, we performed β-elimination experiments with total RNA from somatic cells and reproductive cells (gonidia). In this chemical reaction, an unmethylated 3′ end will lose its terminal base and as a consequence, migrate faster in polyacrylamide gels. As a positive control, an unrelated RNA oligonucleotide with unprotected 3′ end was spiked in prior to the reaction. While the positive control clearly shifted after treatment (Fig. 2a), all analyzed small RNAs from *V. carteri* migrated at the same length as before indicating that they are indeed modified at their 3’ ends (Fig. 2a). These findings are consistent with the presence of a HEN1 homolog in the *V. carteri* genome (Additional file 2: Figure S1) and suggest that our candidate RNAs are most likely miRNA.

After identifying miRNA sequences in *V. carteri* and establishing that they are modified at their 3′ end, we investigated miRNA expression under varying physiological conditions (Fig. 2b). We harvested samples at different life stages of *V. carteri* (asexual growth (V), 16 h induction of the sexual program (I) and female sexual differentiation (F)) and the culture from each stage was split into the two different cell types of *V. carteri* (somatic cells and reproductive cells). RNA blots performed with these samples show that miRNAs are indeed regulated between cell types and some are even regulated during development (Fig. 2b) indicating that *V. carteri* miRNAs might play an important role not only for cellular maintenance but also during differentiation processes. Of note, the U6 loading control signal is much weaker for the reproductive cells and therefore, only limited comparisons between somatic and reproductive cells can be made.

In plants, functional small RNAs are incorporated into AGO proteins and guide them to complementary target RNAs for cleavage. The VcAGO3 protein has a putative catalytic triad composed of the amino acids DDD. Although the canonical motif for a cleavage-competent Ago protein is DDH, AtAgo2 contains DDD as well and appears to be a catalytically active enzyme [21]. Cleavage activity of AGO proteins is usually necessary for gene silencing in plants, although mechanisms similar to mammalian AGO proteins have also been proposed [22]. In order to test the cleavage activity of AGO proteins, *in vitro* cleavage assays can be performed in which AGO proteins are purified from cell lysates and incubated with radioactively 5′ end-labeled, artificial targets that are designed to be fully complementary to an endogenous small RNA bound by the respective AGO protein. After the catalytic reaction, the target RNA is cleaved into two pieces with only the 5′ end being detectable due to its radiolabeled 5′ end. In order to test VcAGO3 for catalytic activity, *V. carteri* cultures were transformed with a plasmid carrying either myc-VcAGO3 or myc-CrGFP (control, GFP codon-optimized for *C. reinhardtii*). After a short selection period, the cultures were harvested, lysed and an immunoprecipitation (IP) was performed using anti-myc antibodies. Each IP was split into three reactions and cleavage assays were performed using three different small RNA target sequences (Fig. 2c). None of the VcAGO3 reactions generated specific cleavage products (Fig. 2c, lanes 3, 6 and 9), while a Flag-tagged AtAGO1 (used as a positive control) showed clear and strong cleavage activity (Fig. 2c, lanes 11 and 13). All proteins were expressed and precipitated successfully as shown by protein blot (Fig. 2d). VcAGO3 appears to be inactive in standard *in vitro* cleavage assays. We cannot exclude, however, that VcAGO3 functions as slicer endonuclease under different *in vivo* conditions.

### *V. carteri* small RNAs associated with transposable elements

**Fig. 3:**
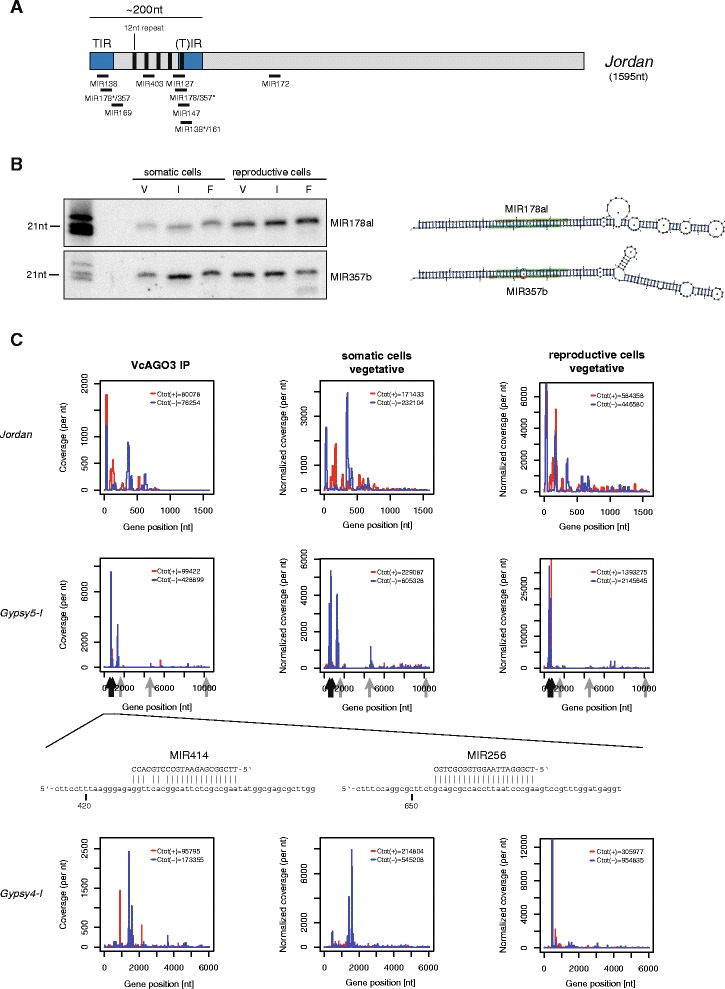
Small RNA mapping to transposable elements. **a** Schematic view of the transposable element *Jordan*. Location of miRNAs targeting *Jordan* are marked with black bars. TIR, terminal inverted repeat; nt, nucleotide. **b** RNA blot validation of the expression two miRNAs encoded by a *Jordan* element. The hairpin structure of the respective putative precursor is shown to the right. The mature (detected) miRNA strand is depicted in red, the star strand in blue. **c** Small RNA coverage of the repetitive elements *Jordan* (*upper* panel), *Gypsy5-I* (*middle* panel) and *Gypsy4-I* (lowest panel). Diagrams show small RNA coverage over the length of the element for the plus (*red*) and the minus (*blue*) strand (consensus sequence from Repbase19.02 was used). The left column shows the reads measured in the VcAGO3 library, the middle column shows reads from the library of somatic cells during vegetative growth and the column on the right shows the reads from the library of reproductive cells during vegetative growth. For *Gypsy5-I*, black arrows indicate miRNA binding sites which are detailed in the panel below. Grey arrows indicate miRNA binding sites where the miRNA expression itself is rather low

To further understand the putative functions of VcAGO3-bound small RNAs, we analyzed their genomic origins in more detail. Interestingly, miR178al and miR357b originate from a transposon termed *Jordan* (Fig. 3a). *Jordan* is a highly abundant transposable element, which resembles the transposable elements *En/Spm* as well as members of the so-called “*CACTA*” family found also in higher plants [23]. These elements transpose via a DNA intermediate and contain terminal inverted repeats (TIRs). In the case of *Jordan*, the TIRs cause a secondary structure that is similar to and recognized as a miRNA precursor and each of these elements gives rise to a miRNA (Fig. 3a). Of note, the precursors of the MIR178 and MIR357 families are highly similar to each other, with one family showing a strong expression of the 5′ arm miRNA sequence (MIR178 family), while the other expresses the 3′ arm (MIR357 family) more strongly. Thus, both miRNAs could target each other’s precursors with perfect complementarity (Additional file 5: Figure S3). To support the inferences from our small RNA-Seq experiments, we performed RNA blots using probes against the *Jordan*-encoded miRNAs (Fig. 3b), which readily validate our sequencing data.

Because transposon-derived miRNAs can trigger a wave of secondary siRNA species in *A. thaliana* [13], we searched for additional VcAGO3-associated small RNAs from *Jordan*. We found many small RNAs originating mainly from the 5′ 0.5 kb portion (Fig. 3c, upper panel). Whether these transposon-derived siRNAs depend on the *Jordan*-encoded miRNAs is currently unknown. Furthermore, we analyzed *Jordan* for potential miRNA target sites and found that several *V. carteri* miRNAs can target *Jordan* (Fig. 3a, black bars). Again, these target sites are largely located in the 5′ 0.5 kb portion of *Jordan*. Taken together, our data suggest that the transposon *Jordan* not only generates two miRNAs and a number of siRNAs, but might also be targeted by the VcAGO3-associated small RNA system, presumably to repress *Jordan* expression. Of note, VcAGO3 appears to be catalytically inactive *in vitro* (Fig. 2a). This might suggest that cleavage-independent silencing mechanisms (e.g. transcriptional silencing by heterochromatin formation) could be active in this process.

We next analyzed whether other transposons are similar sources of different classes of small RNAs. Close examination of VcAGO3-associated small RNAs did not reveal miRNA genes that are encoded by transposons and pass our annotation criteria. However, transposons such as *Gypsy5-I* (Fig. 3c, middle panel) or *Gypsy4-I* (bottom panel) produce a large number of VcAGO3-associated siRNAs. Furthermore, *Gypsy5-I* is in addition targeted by MIR414 and MIR254. Strikingly, a global analysis of our sequencing data revealed that most transposons give rise to small RNAs (Additional file 6: Figure S4), suggesting control of expression through small RNA-guided gene silencing pathways.

At least one miRNA, miR178al, appears to be expressed more strongly in reproductive cells (Fig. 3b), which might suggest that *Jordan* expression is generally lower in such cells. Indeed, our RNAseq data reveals that less *Jordan*-derived reads were cloned from reproductive cells compared to somatic cells. Generally, we observe, with some exceptions, a tendency of reduced transposon activity in reproductive cells (Additional file 7: Figure S5) suggesting repression of such elements in reproductive cells.

### Other *V. carteri* small RNAs

**Fig. 4:**
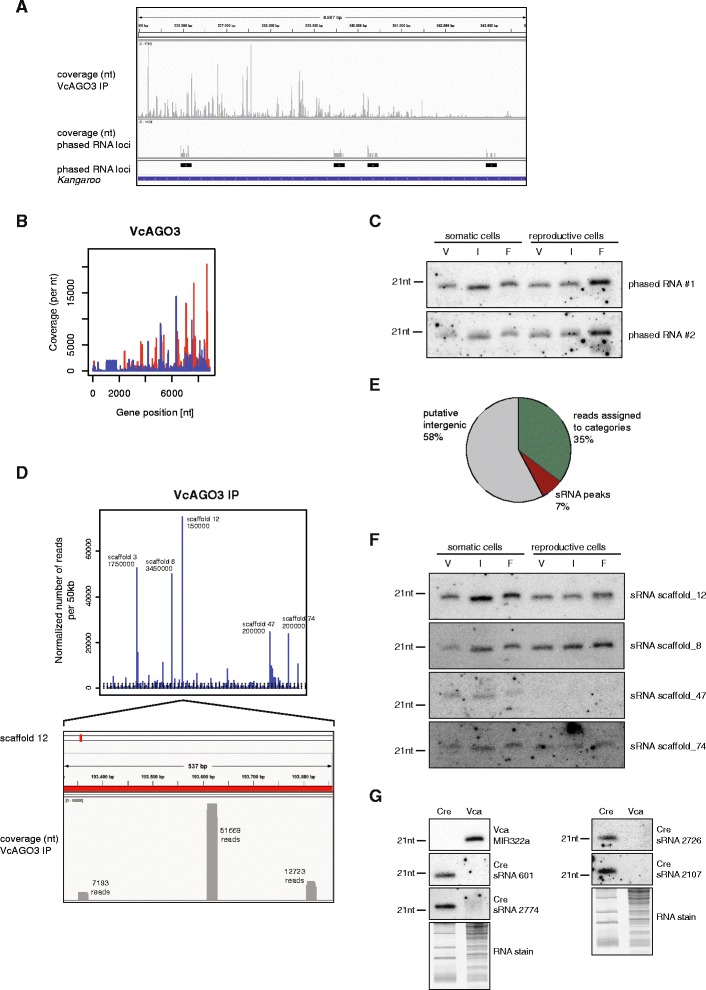
Phased siRNAs and highly expressed small RNAs of unknown function. **a** Exemplary genetic locus of a *Kangaroo* element harboring predicted phased siRNA loci. Upper panel: all reads measured in the VcAGO3 IP library, middle panel: read coverage of the predicted phased siRNA loci, lower panel: bar depiction of phased siRNA loci (*black* bars) and *Kangaroo* (*blue*). **b** Coverage of small RNAs associated with VcAGO3 of the consensus sequence (Repbase19.02) of the repetitive element *Kangaroo.* Red bars represent reads mapping to the plus strand, blue bars represent reads mapping to the negative strand. **c** Validation of expression of small RNA originating from a predicted phased RNA locus. **d** All reads of the putative intergenic fraction that are associated with VcAGO3 are displayed. The x-axis of the diagram represents the length of an artificial genome sequence that was created by aligning all available genome scaffolds in a row. This sequence was divided into 50 kb long sections and all reads that were mapped falling into one section were summed up and displayed by blue bars. The location for an exceptionally high read count was marked next to the corresponding bar. The number below each scaffold indicates the starting point of that section within the scaffold. Lower panel: blow-up of the section in scaffold 12 with the highest read count. **e** Pie chart visualizing the amount of small RNAs reads stemming from highly expressed hotspots from **d** with respect to all reads. **f** Validation of expression by RNA blot. The small RNA with the highest read count within the respective scaffold section (see **d**) was blotted. **g** Total RNA isolated from *C. reinhardtii* (Cre) or *V. carteri* (Vca) was blotted and robed for the indicated small RNAs. sRNA sequences of *C. reinhardtii* were taken from Molnar et al. [14]

In *A. thaliana*, tasiRNAs are produced from RNAs that are specifically cleaved by miRNAs such as miR390. Biogenesis involves dsRNA synthesis by RDR6 and cleavage by DCL4, which progressively cleaves the RNA from the ends and produces phased tasiRNAs [24]. Since we find a putative RdRP in the *V. carteri* genome, we examined our VcAGO3-associated siRNAs for phased siRNA signatures. Indeed, we found several loci that produce phased siRNAs. Most prominently, phased siRNA loci can be found within the transposon *Kangaroo* (Fig. 4a). Whether these siRNAs require miRNA-guided cleavage as observed for tasiRNAs in *A. thaliana,* is unclear. Similar to other transposons, *Kangaroo* produces various other VcAGO3-associated small RNAs in addition to the phased siRNAs (Fig. 4a upper part and b). To further investigate phased siRNAs, we performed RNA blots (Fig. 4c). The two most abundant single phased siRNAs were readily detectable, with the highest expression in reproductive female cells.

A large portion of the VcAGO3-bound small RNAs did not fit into any known small RNA category, but could be aligned to the genome, indicating that they originated from *V. carteri*, and not from a potential associated microbe. In fact, about 11 % of the putative intergenic reads (7 % of total reads) came from eight specific loci (Fig. 4d and e), further supporting that such sequences are likely not due to promiscuous binding of VcAGO3 to RNA degradation products. The specificity is further supported by the observation that most of the small RNAs within a peak are identical. It is also unlikely that these reads are unspecifically amplified during library construction since RNA blots supported robust expression and stability (Fig. 4f). For expression analysis, we performed Northern blotting of total RNA obtained from somatic and reproductive cells. Probes directed against the most prominent scaffolds (Fig. 4d) are readily detectable thus suggesting that these reads are indeed abundant small RNAs (Fig. 4f). It is therefore tempting to speculate that these VcAGO3-associated reads might represent a novel class of small RNAs.

### Comparative genomics of small RNAs in green algae

Our sequencing data demonstrate that *V. carteri* contains many miRNA candidates. It has been shown before that *C. reinhardtii* expresses miRNAs and other small RNA classes [14, 15]. Since both species are closely related, we analyzed conservation of our VcAGO3-associated miRNAs. For direct examination, total RNA from *V. carteri* as well as *C. reinhardtii* was extracted and several miRNAs were assayed with RNA blots (Fig. 4g). Indeed, *V. carteri* MIR322a is not detected in *C. reinhardtii*. Vice versa, a number of sRNAs were only found in *C. reinhardtii* but not in *V. carteri* samples. Subsequently, a global analysis of miRNA sequences was performed using multiple sequence alignments of every miRNA of *V. carteri* with every miRNA of *C. reinhardtii* [25] or the liverwort *Pellia endiviifolia* [26]*.* Consistent with our RNA blot experiments, these global analyses revealed very little conservation between *V. carteri* and *C. reinhardtii* (Additional file 8: Figure S6A) or *V. carteri* and *P. endiviifolia* (Additional file 8: Figure S6B). Only three miRNAs had before been reported as potentially conserved between *C. reinhardtii* and *P. endiviifolia* [26].

## Discussion

We have cloned and sequenced VcAGO3-associated small RNAs from different *V. carteri* stages. Since we select for VcAGO3-bound small RNAs only, it is very likely that we identify functional small RNAs and minimize background cloning that would contaminate our libraries. Indeed, our VcAGO3 pull down approach allows for a clear definition of *V. carteri* miRNAs [19]. Of note, our miRNAs are distinct from those reported recently [16]. This might be due to different *V. carteri* strains that have been used or, more likely, to mis-annotation of miRNAs as a result of background sequencing from total RNA samples.

An intriguing observation from our small RNA profiles was that many VcAGO3-bound small RNAs originate from transposons. Different classes including miRNAs, siRNAs and even phased siRNAs have been identified in conjunction with transposons. Since miRNA genes are encoded by the transposon *Jordan*, it is conceivable that this mobile genetic element influences spreading of miRNA genes and might therefore contribute to evolution. Furthermore, transposons are at the same time targeted by miRNAs suggesting that miRNAs might repress the expression of such elements. In addition, transposons appear to produce large numbers of siRNAs especially in reproductive cells. It is therefore likely that transposon targeting by small RNAs might be involved in repressing transposable elements in reproductive cells, a phenomenon that is often observed in animals [27]. Interestingly, computational analyses have suggested that both *A. thaliana* and *Oryza sativa* have transposable elements that encode simultaneously siRNAs and miRNAs [28]. Whether this suggests a common origin, or convergent evolution of such mechanisms in algae and land plants is unclear.

Our analyses reveal also interesting evolutionary aspects when analyzing conservation of closely related species. At least the VcAGO3-associated miRNAs are not conserved in the closely related single cellular alga *C. reinhardtii*, suggesting evolution of different miRNAs presumably required for multicellularity and division of labor between somatic and reproductive cells. This is remarkable given that so many protein-coding genes are well conserved between the two species. It will be interesting to analyze whether other small RNAs, e.g. small RNAs that target transposable elements such as *Jordan*, are conserved. Our findings suggest that targeting such mobile genetic elements by the small RNA pathways developed very early during evolution and the last universal common ancestor of all eukaryotes used such strategies already.

## Conclusion

We conclude from our analyses that VcAGO3 not only associates with miRNAs but also many other classes of functional small RNAs. These small RNAs are most likely not conserved in *C. reinhardtii* suggesting evolution of small RNA repertoires that are important for multicellularity and the division of labor. Interestingly, a distinct portion of the VcAGO3-associated small RNAs is derived from transposable elements. These include miRNAs, classical siRNAs and even phased siRNAs, which are common to higher land plants. Taken together, our data identify an extended small RNA system in *V. carteri*, which appears to be as complex as in higher plants.

## Methods

### Separation of cell types

About 5000 asexual growing *V. carteri* spheroids at the stage shortly before the onset of embryogenesis were collected by filtration on a 100 μm mesh nylon screen and broken by passing them through a 0.6 mm hypodermic needle. The suspension was filtered on a 100 μm mesh nylon screen, which allows free reproductive cells (gonidia), free somatic cells and small fragments of the somatic cell layer to pass but retaining larger fragments of somatic cell layers nearly free from gonidia. The filtrate was passed in a second step through a 40 μm mesh nylon screen which retained only gonidia and small fragments of the somatic cell layer. The resuspended residue was allowed to settle down repeatedly in a small volume separating the reproductive cells from somatic cell layers.

The separation of gonidia and somatic cell layers of sexual induced *V. carteri* spheroids at the stage shortly before the onset of embryogenesis and 16 h after application of the sex-inducing pheromone was done in the same manner as described above.

The separation of egg cells and somatic cells of sexual growing *V. carteri* spheroids 64 h after application of the sex-inducing pheromone was done in a modified procedure: Egg cells were set free by passing the spheroids twice through a 0.5 mm hypodermic needle. A first filtration step on a 40 μm mesh nylon screen retained large fragments of the somatic cell layer. Egg cells which pass through were collected on a 10 μm mesh nylon screen and purified further by let them settle down repeatedly in a small volume. The residue of the first filtration step was dissociated once more by passing through a 0.4 mm hypodermic needle. Fragments of the somatic cell layer were collected on a 40 μm mesh nylon screen.

### Production of stable *V. carteri* strains using the gold particle gun

#### Precipitation of DNA (plasmids) onto gold-microcarriers

For each transformation, 10 μg of plasmid DNA encoding the selection marker (pPmr3, see [29]) and 10 μg plasmid encoding the target gene were used. The DNA solution was added to 0.5 μmol gold particles while mixing vigorously. Continuing the shaking, 125 μmol CaCl_2_ (50 μl of 2.5 M) and 2 μmol spermidin (20 μl of 100 mM) were added. The mixture was further incubated at 4 °C for 30 min under continuous shaking. To precipitate the DNA onto the gold, 200 μl of 100 % ethanol were added, followed by a short centrifugation step (3–4 s at 8000 rpm). The gold particles were washed three times with ice cold 100 % ethanol and taken up in a total volume of 40 μl. The particles were stored on ice until transformation.

#### Nuclear transformation

Nuclear transformation was carried out according to Jakobiak et al., 2004 [29]. One aliquot of gold particles were used to transform *V. carteri* spheroids from one Fernbach flask. For this, the gold particle mixture was spread over the center of 6 macrocarriers (fixed in their metal carriers). The macrocarriers were warmed on a heating block at 37 °C to evaporate residual ethanol. One macrocarrier, stopping screen and rupture disk (900 psi) were placed into the apparatus. The *V. carteri* spheroids were harvested using a sieve and spread in its center. The sieve was placed directly below the macrocarriers. Transformation was carried out under vacuum using the biolistic PDS 1000/He particle gun (BioRad Laboratories, Hercules, USA). For one transformation, the *V. carteri* spheroids were six times spread on the sieve, bombarded with gold particles and submerged in *V. carteri* medium during change of the carriers and disks.

Following bombardment, the spheroids were split into ten petri dishes containing 30 ml medium. Two days after transformation, 30 μg/ml paromomycin (selection marker) was added to each plate. After selection of paromomycin positive clones, the concentration was decreased to 10 μg/ml.

### Cloning and sequencing of RNA transcripts

The RNA Seq libraries were generated from 4 μg total RNA per sample. The TruSeq Sample Preparation Kit (Illumina) was used according to the manufacturer’s instructions. The only derivation of the protocol was the substitution of the Superscript II reverse transcriptase with the Superscript III reverse transcriptase (both Life Technologies). Accordingly, the cDNA synthesis temperature was raised from 42 °C to 50 °C. Libraries of two biological replicates were generated on different days and sequenced on a HiSeq 2000 at the facilities of Illumina in a 100 bp paired end run. Additionally, the same libraries were sequenced in single runs (100 bp) with GATC (Konstanz, Germany). For the generation of strand-specific libraries, samples of all cells in all life stages were pooled. Total RNA was enriched for mRNA using the polyA Purist Kit (Life Technologies Cat No AM1919). Library was made with SOLiD total RNA-seq kit (Life Technologies Cat No 4445374) in accordance with manufacturer’s protocol and sequence on SOLiD 3 platform.

### Northern blotting

Northern blotting was performed as described before [30]. In short, 5–10 μg of total RNA were run on a 12 % urea gel (UreaGel System, National diagnostics). For determining the approximate size of the small RNAs, ribooligonucleotides with a length of 19, 21 and 24 nucleotides (nt) were labeled with ^32^P prior to loading. The gel was run for 1 h at 250–350 V and semi-dry blotted onto an Amersham Hybond-N membrane (GE Healthcare) at 20 V for 30 min. Cross-linking with EDC-solution was performed at 50 °C for 1 h. The membrane was subsequently rinsed in water, dried and incubated with hybridization solution at 50 °C. After pre-hybridization, a radiolabeled probe antisense to the target small RNA was added and incubated over night at 50 °C. For labeling, 20 pmol of the probe (DNA oligonucleotide) were incubated with 20 μCi of ^32^P in a T4 PNK reaction (Fermentas) and it was purified with a G-25 column (GE Healthcare). After the incubation, the membrane was washed twice with 5xSSC, 1 % SDS, once with 1xSSC, 1 % SDS and wrapped in saran. The detection of signals was performed by the exposure to a screen and scanning with the PMI (Biorad).

For the re-probing of a membrane, the membrane was incubated with hot water with 0.1 % SDS for at least 15 min on a tumbler. The membrane was exposed to a screen for at least overnight to control for residual signal.

For the assessment of band heights when the radioactive marker had faded, the distance between the blue dye band heights was measured (marked with pencil on the membrane before blotting) as well as the distances of the marker bands to the blue dye bands. These figures were used to estimate band heights in later blots.

### Data analysis

#### Analysis of RNA-Seq data

All small RNA sequencing data were adapter- and quality score-trimmed (trailing/leading base Phred score > 20). Resulting reads were aligned to the *V. carteri* reference genome version 9.0 [31] using tophat2/bowtie2 [32].

Paired-end total RNA sequencing data were also adapter- and quality score-trimmed (trailing/leading base Phred score > 30), and aligned to the same *V. carteri* reference genome version 9.0.

#### Transcriptome assembly

Paired-end and single-end reads from libraries V1-V12 were mapped each separately to the *V. carteri* genome assembly vc199 (Phytozome v.8 [31]) using TopHat software v.1.4.1 [33] and separate de novo transcriptome assemblies were generated for each library using Cufflinks software V 2.0.1 [33] with default parameters. Next, all assemblies were merged using cuffmerge program from the Cufflinks package. Finally, transcript orientation was corrected by mapping strand-specific SOLiD RNA-seq data on the transcripts using Bowtie software v.0.12.7 [32] and assigning particular orientation if the number of reads mapping to one strand was at least two times greater than the number of reads mapping to the opposite strand. In cases where this was not possible, no strand correction was performed.

#### Identification of transcripts with a putative role in RNAi

HHblits [34], which is part of the HHsuite, was used to search for fragments of RNAi processing proteins in the transcriptome of *V. carteri*. We used the current transcript assembly v.2 of the JGI (Joint Genome Institute), which is freely available at the Phytozome 10 database [31]. In addition, the transcriptome data generated by us was used for this analysis (see paragraph above). HHblits requires for each protein to be searched for a hidden-Markov-model (HMM) to be generated by the user. These HMMs constitute a custom database, which was compiled according to the protocol detailed in chapter 3.4 of the HHSuite User Guide (downloaded from https://toolkit.tuebingen.mpg.de). To initiate the compilation of the respective HHMs, the following proteins from *Arabidopsis thaliana* were used as a seed: DCL4 (UniProtKB ID P84634), DCL1 (Q9SP32), HEN1 (Q9C5Q8) and RdRP6 (Q9SG02). For the search of additional Argonaute genes, the modified *V. carteri* AGO3 was used. For each sequence, a multiple sequence alignment was created by using HHblits applied to the database uniprot20_2013_03 from the EBI (http://www.uniprot.org/). As required, secondary structure was predicted by means of psipred_3.5 [35] and added to the HHblits alignments. The compilation of the database was finalized according to the above-mentioned protocol. Since the open reading frames of annotated *V. carteri* proteins were not always supporting full-length proteins with start and stop codons, different protein sequences were created. DNA was translated in the six putative reading frames to protein sequences by means of methods from the Biophython package (https://github.com/biopython/biopython.github.io/). HHblits hits with an E-value ≤ 10E^−5^ were considered important and further processed. Next, the genomic locus of each transcript was extracted and the list of transcripts was sorted accordingly. Transcripts were grouped according to coverage of the query protein, e.g. for Argonaute, two transcripts in close proximity with one encoding the N-terminal and the other encoding the C-terminal part were considered to be an important hit. To further validate the candidate genes, the protein search algorithm of Pfam version 27.0 [36] and Panther HMM Sequence Scoring [37] were employed to identify single important domains in the respective transcripts.

#### miRNA identification

The miRNA identification was performed using the novel miRNA identification tool miRA [19]. miRA does not assume sequence conservation, and was developed to characterize the miRNA landscape in organisms exhibiting heterogeneous miRNA precursor populations. Key parameters were chosen based on an analysis of miRBase-annotated miRNAs in *C. reinhardtii*. Details involving the identification method can be found in Evers et al. [19].

#### Annotation of other small RNAs

Reads overlapping annotated exons of mRNA transcripts were assigned to the mRNA fraction. Gene annotations were based on the *V. carteri* reference genome version 9.0 [31]. Since there are no databases listing *V. carteri* rRNA and tRNA genes in full, reads were mapped against the database entries for *C. reinhardtii* tRNAs (PlantRNA database, [38] and rRNAs from *A. thaliana* (SILVA database, [39] and the order Volvocales (exported from GenBank, NCBI). Repeats were assigned using Repbase Update 19.02 [40], phased RNAs were predicted using the ta-si prediction tool from the UEA small RNA Workbench [41] which is based on the algorithm by Chen et al. [42].

#### miRNA target prediction

Potential miRNA targets in *V. carteri* were identified by requiring the following set of matching rules between the miRNA seed region and complementary mRNA binding site (adapted from [14]): (a) the binding site of the miRNA should have no more than four mismatches, (b) there should not be a mismatch at positions 10 an 11 of the miRNA, because complementarity here is required for cleavage, (c) there should not be adjacent mismatches at positions 2–12, (d) no more than two adjacent mismatches for positions > 12 are allowed and (e) there should be no bulge in the miRNA.

#### Mapping of small RNAs to repetitive elements

To investigate transcription of small RNAs from repetitive elements, the following strategy was employed: Small RNA reads were mapped to a list of RepBase-derived *V. carteri* repeat elements using tophat2/bowtie2. Overall the AgoIP sequencing library showed the strongest repeat element-associated expression, with *Kangaroo*, *Gypsy3–5*, and Jordan being amongst the most highly expressed repeat elements in the sample.

#### Generation of logos

All sequence logos were based on the adapter- and quality score-trimmed reads, and were generated using the R package motifStack (Ou and Zhu, R package version 1.12.0).

#### Generation of multiple sequence alignment

All sequences of miRNAs of *V. carteri* and *C. reinhardtii* (miRBase) were subjected to a multiple sequence alignment using ClustalW [43]. The resulting matrix of alignment quality scores was plotted as a heat map of the pairwise score as percentage identity. The same analysis was performed with miRNAs from the liverwort *Pellia endiviifolia* [26].

### Strains and culture conditions of *V. carteri* and *C. reinhardtii*

The female *Volvox carteri f. nagariensis* wild-type strain HK10 was originally obtained from R.C. Starr (Culture Collection of Algae, University of Texas, Austin, Texas, USA). The strain Vol6♀ was obtained from A. Hallmann (University of Bielefeld, Germany). Synchronous cultures were grown in *Volvox* medium at 28 °C under an 8 h dark/16 h light (10 000 lux) cycle [3]. The sex-inducing pheromone was used as described by Haas and Sumper [44].

The cell-wall deficient strain CW15 mt- was obtained from Jörg Nickelsen (University of Munich) and cultivated on agar plates from TAP medium at room temperature on a shelf exposed to natural sunlight. TAP medium and its ingredients were made as described at chlamy.org, originally described in Gorman et al. [45]. Liquid cultures were grown in TAP medium in Erlenmeyer flasks at room temperature and exposed to natural sunlight.

### Immunoprecipitation and protein blots

Approximately 1 ml (myc-VcAGO3 transformed) or 400 μl (myc-CrGFP transformed) of *V. carteri* spheroids were lysed in 5 ml or 2 ml of lysis buffer (150 mM KCl, 25 mM Tris/HCl pH7.4, 0.5 % NP-40, 2 mM EDTA, 1 mM NaF), respectively, and sonicated twice for 30s (cycle 5, 10 % intensity, MS72 tip, Bandelin Sonoplus). After centrifugation at 17000 *g*, 4 °C and 30 min, the supernatant was flash frozen in liquid nitrogen. After thawing on ice the next day, the lysates were incubated with anti-c-myc-beads (Sigma) and incubated for 2.5 h at 4 °C on a rotating wheel. Beads were washed three times with wash buffer (300 mM KCl, 50 mM Tris/HCl pH7.4, 1 mM MgCl_2_, 0.1 % NP-40) and once with PBS.

For protein blots, 10 μl of the respective lysate as input and 20 % of the immunoprecipitation (IP) of the myc-VcAGO3 transformation and all of the myc-CrGFP transformation was used. For the control of immunoprecipitation of the cleavage assays, 10 % of each reaction was used.

Standard protein blots were performed using a 10 % separating gel. Semi-dry blotting using Towbin buffer was performed with 1.5 mA/cm^2^ for 2 h on Hybond ECL membrane (GE Healthcare). After blocking for 30 min with 5 % milk powder in TBS-T (TBS with 0.2 % Tween20), the membrane was incubated with the primary antibody (anti-myc, Sigma, 1:1500; anti-Flag 1:1000) over night at 4 °C. After washing with TBS-T, the membrane was incubated with the secondary antibody (anti-rabbit IRDye 800CW, Licor, 1:10000) for 1 h at room temperature. After washing, the membrane was scanned on a Licor Odyssey reader.

### RNA preparations

For the extraction of RNA from somatic cells or reproductive cells, 3 ml peqGold Trifast (Peqlab) or Trizol (Life technologies) was added per 100 μl cell suspension. Cells were lysed at room temperature for 10 min and RNA preparation was then carried out according to the manufacturer’s instructions. Precipitation of the RNA was performed over night at −20 °C in the presence of 20 μg glycogen (RNA grade, Life technologies) per ml of Trifast/Trizol. The RNA was collected by centrifugation, washed once with cold 80 % ethanol and solved in pure water.

RNA from immunoprecipitation samples was prepared as described [30].

### Cleavage assay

Before assessing the cleavage activity, AGO proteins and the negative control CrGFP were precipitated via their respective tags. For VcAGO3 and CrGFP, the IP was carried out with 0.5 ml pellets as described under section “Immunoprecipitation and protein blots”. For lysing Arabidopsis tissue, 250 mg of tissue powder were incubated with 1 ml of ice cold extraction buffer (50 mM Tris HCl pH 7.5, 150 mM NaCl, 10 % Glycerol, 5 mM MgCl2, 0.1 % NP-40, 5 mM DTT), mixed well and incubated on a rotating wheel at 4 °C for 1 h. After clearing the cell debris by centrifugation (17,000 *g*, 4 °C, 30 min), 1 ml of lysate was added to 30 μl of packed Flag-beads (anti-Flag M2, Sigma) and incubated for 2 h at 4 °C on a rotating wheel. After three washes with wash buffer (50 mM Tris HCl pH 7.5, 500 mM NaCl, 10 % Glycerol, 5 mM MgCl_2_, 0.1 % NP-40, 4 mM DTT), PBS was used to split the immunoprecipitated AtAGO1 into fresh tubes. For checking the success of immunoprecipitation, 10 % of each IP (*Volvox* and *Arabidopsis*) were used for Western blotting.

The preparation of ^32^P-cap-labeled target RNA was performed as described before [8]. For VcAGO3 cleavage assays, targets were generated carrying a perfectly complementary site to the small RNAs tasiRNA#1, MIR357b and sRNA scaff 12, while target RNAs for AtAGO1 cleavage assays carried sites for MIR159a and MIR165. The *in vitro* cleavage reaction was carried out with 50 % (v/v) immunoprecipitated protein (beads) in 66.7 mM KCl, 6.7 mM MgCl_2_, 8.3 mM DTT, 1.7 mM ATP, 0.3 mM GTP and 3.2 U RiboLock RNase inhibitor (Thermo Scientific). The addition of target RNA (1–2 Bq/cm^2^) initiated the reaction which was carried out for 1.5 h at 25 °C. The RNA was subsequently extracted using Proteinase K digestion and phenol-chloroform extraction as described before [46].

To visualize the cleavage products, samples were run on an 8 % urea polyacrylamide sequencing gel (National diagnostics), dried on Whatman paper and exposed to a screen.

### Cloning and sequencing of small RNAs

Small RNAs from total RNA samples were converted into sequencing libraries as described before [30]. The libraries were sequenced by Fasteris SA (Geneva, Switzerland) on an Illumina HiSeq2000 in a 50 bp single-end run. The RNA that was extracted from a VcAGO3 immunoprecipitation was cloned as described before [47] and sequenced on a MiSeq (Illumina) in a 66 bp single-end run.

### β-elimination treatment of small RNAs

Total RNA from vegetative somatic cells and vegetative gonidia was mixed with 20 pmol of a random oligo RNA (5′-UUAGUGAGAGUCCAAUUAAUU-3′, Biomers). Beta-elimination was performed as described previously [48].

13.5 μl of total RNA (10–20 μg, vegetative somatic cells or vegetative gonidia) were mixed with 4.5 μl of 5x borate buffer (148 mM borax, 148 mM boric acid, pH 8.6) and 2.5 μl of freshly dissolved 200 mM NaIO_4_. After incubation for 10 min at room temperature, 2 μl of glycerol were added to quench unreacted NaIO_4_. Samples were incubated for another 10 min at room temperature and then dried by vacuum centrifugation for 1 h at room temperature.

Samples were dissolved in 50 μl 1x borax buffer (30 mM borax, 30 mM boric acid, 50 mM NaOH, pH 9.5) and incubated at 45 °C for 90 min. 20 μg glycogen was added to each sample and the RNA was precipitated with 2.5 volumes of ethanol at −20 °C over night. RNA was collected by centrifugation at 17000 *g*, 4 °C, 30 min. Pellets were directly dissolved in RNA loading dye.

## Additional files

Additional file 1: Table S1. Read count and mapping efficiencies of small RNA and RNA-Seq libraries used in this study. Most libraries could be mapped with more than 80 % of all reads. Rep = replicate. (PDF 165 kb)
Additional file 2: Figure S1. Gene structure and putative domain structure of proteins involved in small RNA biogenesis. (A) Description of a second full length Argonaute protein in *V. carteri*. Potential transcripts at this locus are shown in green, the annotation by the Joint Genome Institute (JGI) as well as our own transcriptome assembly (“Cufflinks assembly”) were analyzed. The transcripts marked by a blue rectangle are overlapping and could manually be assembled to yield the transcript shown in black and marked with a red rectangle. The putative domain structure of the protein is depicted below. (B) The domain structure of a Dicer-like protein was assembled using several transcripts marked here in red rectangles. (C) Transcript and domain structure of a putative RNA-dependent RNA Polymerase (RdRP). (D) Transcript and domain structure of a putative HEN-1 homolog. (PDF 58 kb)
Additional file 3: Table S2. Full list of identified miRNA sequences in this study. The table shows the location and sequence of each miRNA within the genome, also the position of the precursor (pre) is given. Since sometimes multiple miRNAs originated from one precursor, an assignment was made in order to be able to correctly pair and differentiate each mature and star sequence. (PDF 433 kb)
Additional file 4: Figure S2. Categorization of small RNA reads. (A) All six total small RNA libraries (vegetative somatic cells, induced somatic cells, female somatic cells, vegetative gonidia, induced gonidia and female egg cells were mapped according to Fig. 1E. In short, reads overlapping annotated exons of mRNA transcripts were assigned to the mRNA fraction. For the annotation of tRNAs and rRNAs, reads were mapped against the database entries for *C. reinhardtii* tRNAs and rRNAs from *A. thaliana* and the order Volvocales. Repeats were assigned using Repbase Update 19.02 [40], phased RNAs were predicted using the ta-si prediction tool from the UEA small RNA Workbench [41] which is based on the algorithm by Chen et al. [42]. MiRNAs were predicted using the tool miRA [19]. RE, repetitive elements. (B) Mapped reads from the libraries in (A) are shown according to Fig. 1F, i.e. categories are depicted without the putative intergenic reads. RE, repetitive elements. (PDF 42 kb)
Additional file 5: Figure S3. MiRNA target sites on *Jordan*. Two exemplary target sites are shown, where a miRNA that originates from a Jordan element targets also a Jordan element. The miRNA is highlighted in grey and shown in 3′ to 5′ sequence. Part of the respective target sequence is shown below, lines indicate pairing of bases. The numbers on the right give the genome coordinates of the respective Jordan element. (PDF 36 kb)
Additional file 6: Figure S4. Small RNA coverage of repetitive elements. All seven small RNA libraries (VcAGO3 IP (“AgoIP”), vegetative somatic cells (“asx_som”), induced somatic cells (“ind_som”), female somatic cells (“fem_som”), vegetative gonidia (“asx_gon”), induced gonidia (“ind_gon”) and female egg cells (“fem_egg”) were mapped to the consensus sequence of all repetitive elements deposited in Repbase19.02 (see also main Figs. 3 and 4). Each page shows the coverage of one repetitive element. Each graph depicts the read abundance, i.e. coverage by nucleotide (y-axis) over the length of the repetitive element (x-axis). Coverage on the plus strand is depicted in red, coverage on the minus strand in blue. Ctot = total count. (PDF 2101 kb)
Additional file 7: Figure S5. Normalized expression of repetitive elements that are targeted by miRNAs. The diagrams show the normalized expression of the elements measured by RNA-Seq in the six conditions: Vegetative somatic cells (“som,veg”), induced somatic cells (“som,ind”), female somatic cells (“som,fem”), vegetative gonidia (“rep,veg”), induced gonidia (“rep,ind”) and female egg cells (“rep,fem”). (PDF 46 kb)
Additional file 8: Figure S6. Multiple sequence alignment of *V. carteri* miRNAs with (A) *C. reinhardtii* miRNAs or (B) miRNAs of the liverwort *Pellia endiviifolia*. The alignment was constructed using ClustalW [43]. The color code indicates the measure of similarity between the sequences. Red indicates no or low similarity, a light yellow or white shows a high similarity. The plot on the right depicts the frequencies of similarity over the whole data set. No sequence with conservation/high similarity could be found. (PDF 1889 kb)

## Abbreviations

*A. thaliana*: *Arabidopsis thaliana*
*C. reinhardtii*: *Chlamydomonas reinhardtii*
CrGFP: GFP codon-optimized for *C. reinhardtii*
miRNA: microRNA
nt: Nucleotide(s)
tasiRNA: Trans-acting short interfering RNA
*V. carteri*: *Volvox carteri*
VcAGO3: *Volvox carteri* ARGONAUTE 3
