# Supplementary material for: Gene silencing pathways found in the green alga Volvox carteri reveal insights into evolution and origins of small RNA systems in plants
Source: BMC Genomics. 2016 Nov 2;17:853. doi: 10.1186/s12864-016-3202-4 (PMC5093975; doi:10.1186/s12864-016-3202-4)
Supplement: Additional file 1: Table S1. — Read count and mapping efficiencies of small RNA and RNA-Seq libraries used in this study. Most libraries could be mapped with more than 80 % of all reads. Rep = replicate. (PDF 165 kb) [file 12864_2016_3202_MOESM1_ESM.pdf]

Additional file 1: Table S1: Read count and mapping efficiencies of small RNA and RNA-Seq libraries used in this study.

Small RNA sequencing

Library	Number of mapped reads	Mapping efficiency
Ago3 IP	2.4×10^6	89.2%
Vegetative, somatic cells	4.4×10^6	91.5%
Vegetative, gonidia	8.6×10^6	83.4%
Induced, somatic cells	13.0×10^6	92.4%
Induced, gonidia	17.0×10^6	90.3%
Female, somatic cells	8.0×10^6	91.0%
Female, egg cells	17.2×10^6	92.7%

RNA-Seq

Library	Number of mapped read pairs	Mapping efficiency
Vegetative, somatic cells, rep 1	26.2×10^6	90.6%
Vegetative, somatic cells, rep 2	26.3×10^6	83.3%
Vegetative, gonidia, rep 1	26.0×10^6	89.8%
Vegetative, gonidia, rep 2	18.9×10^6	83.0%
Induced, somatic cells, rep 1	19.7×10^6	88.4%
Induced, somatic cells, rep 2	20.0×10^6	78.5%
Induced, gonidia, rep 1	4.6×10^6	69.4%
Induced, gonidia, rep 2	3.0×10^6	65.2%
Female, somatic cells, rep 1	16.4×10^6	87.5%
Female, somatic cells, rep 2	22.3×10^6	81.5%
Female, egg cells, rep 1	19.2×10^6	80.7%
Female, egg cells, rep 2	33.3×10^6	87.3%
