# Supplementary figures and images for: Gene silencing pathways found in the green alga Volvox carteri reveal insights into evolution and origins of small RNA systems in plants
Source: BMC Genomics. 2016 Nov 2;17:853. doi: 10.1186/s12864-016-3202-4 (PMC5093975; doi:10.1186/s12864-016-3202-4)

A

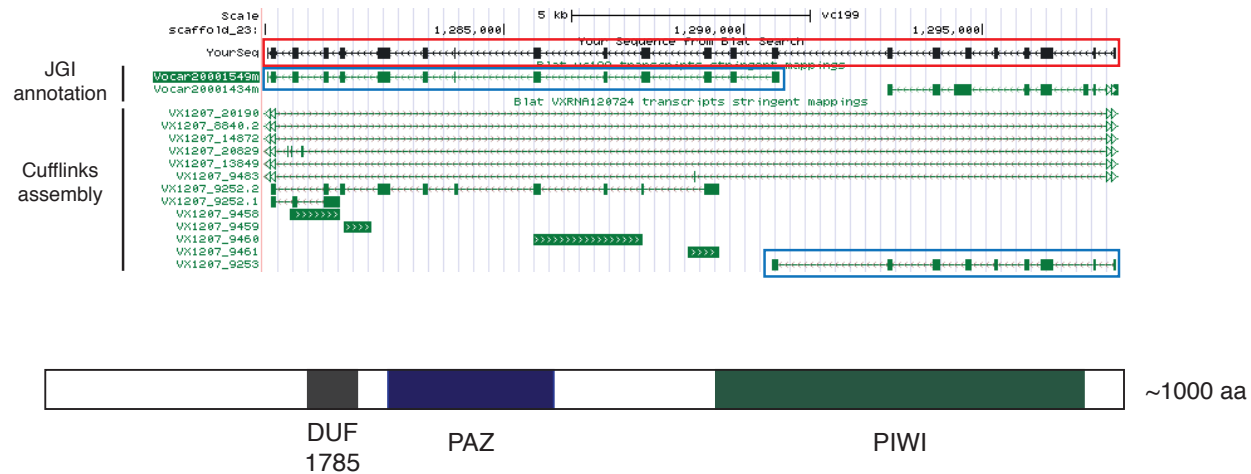

B

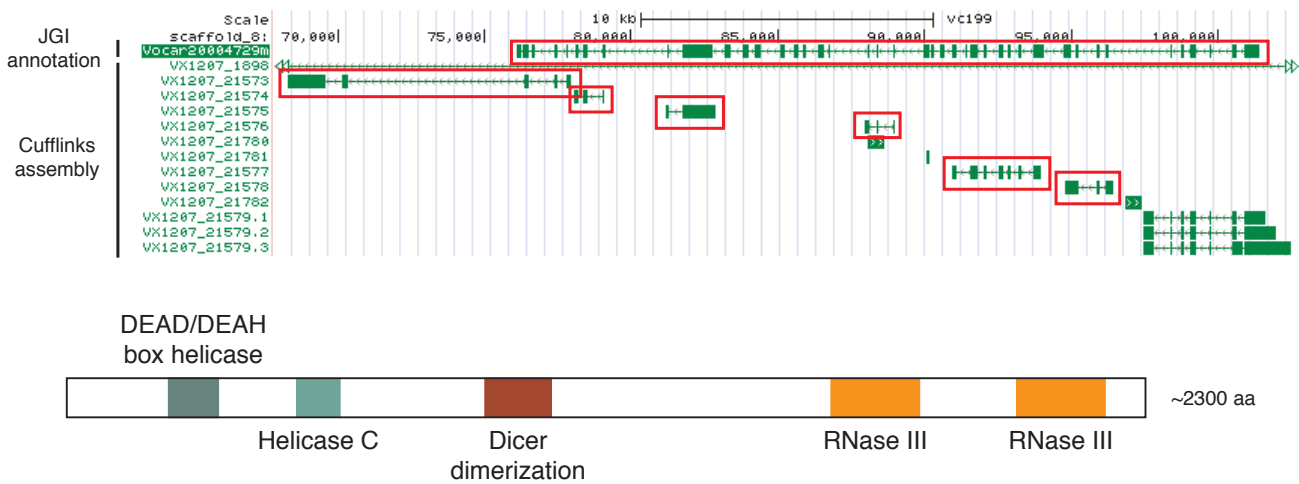

C

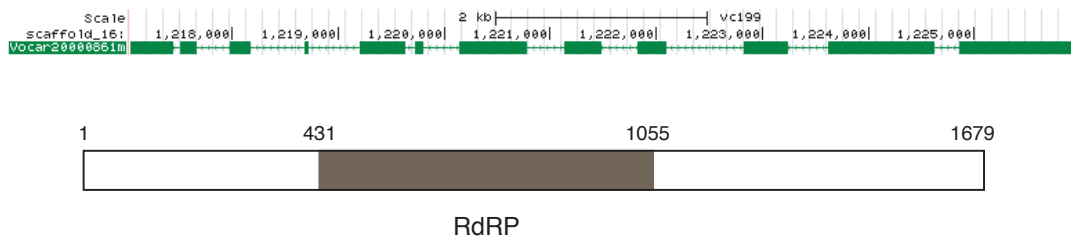

D

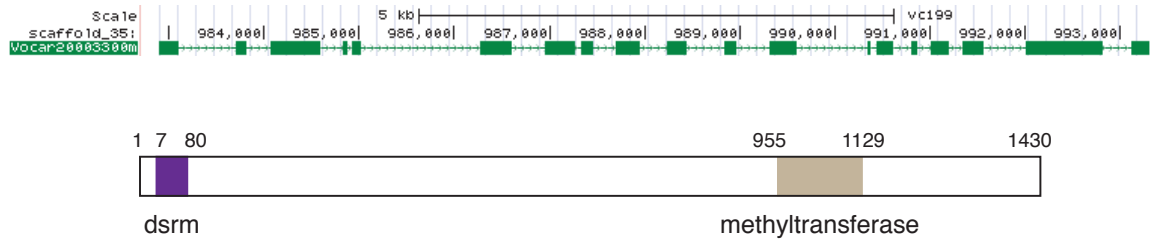

Supplement: Additional file 2: Figure S1. — Gene structure and putative domain structure of proteins involved in small RNA biogenesis. (A) Description of a second full length Argonaute protein in V. carteri. Potential transcripts at this locus are shown in green, the annotation by the Joint Genome Institute (JGI) as well as our own transcriptome assembly (“Cufflinks assembly”) were analyzed. The transcripts marked by a blue rectangle are overlapping and could manually be assembled to yield the transcript shown in black and marked with a red rectangle. The putative domain structure of the protein is depicted below. (B) The domain structure of a Dicer-like protein was assembled using several transcripts marked here in red rectangles. (C) Transcript and domain structure of a putative RNA-dependent RNA Polymerase (RdRP). (D) Transcript and domain structure of a putative HEN-1 homolog. (PDF 58 kb) [file 12864_2016_3202_MOESM2_ESM.pdf]

A

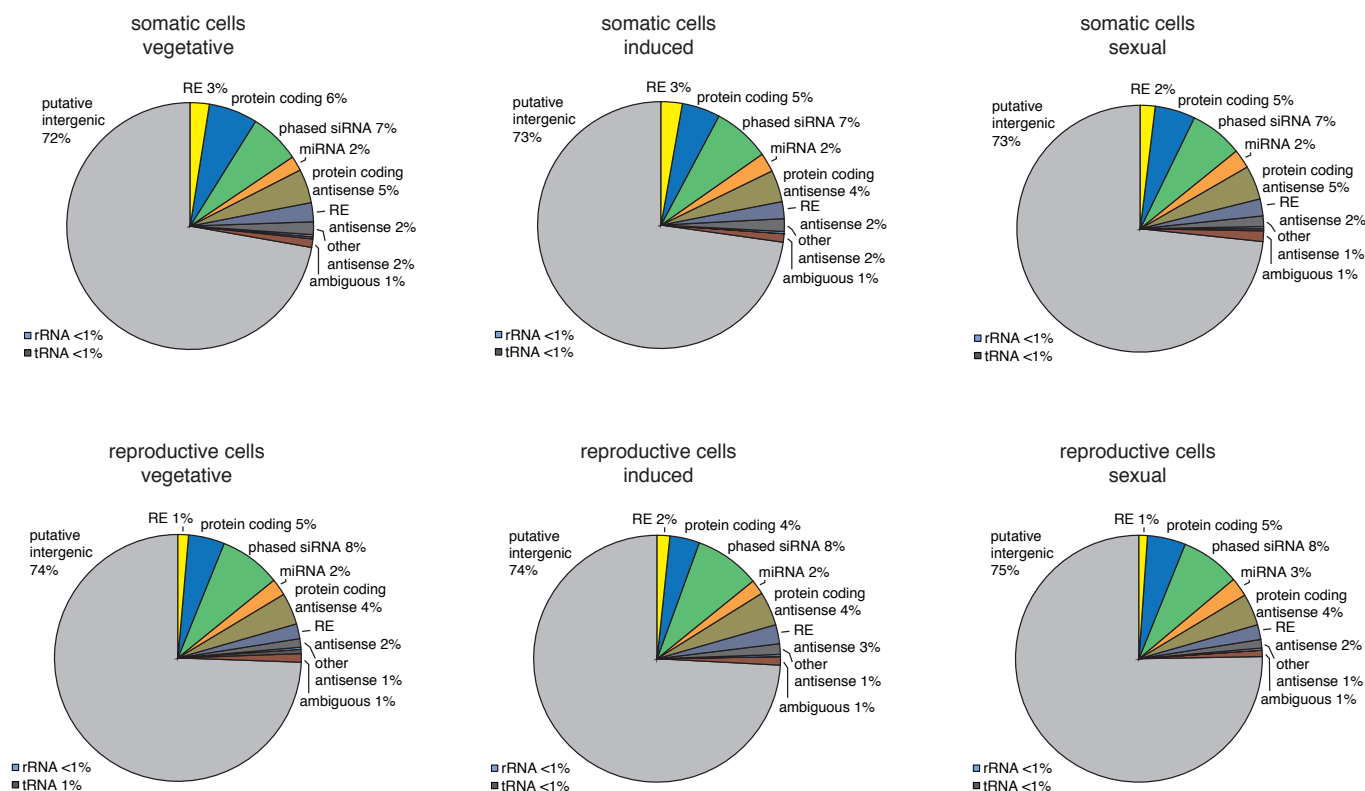

B

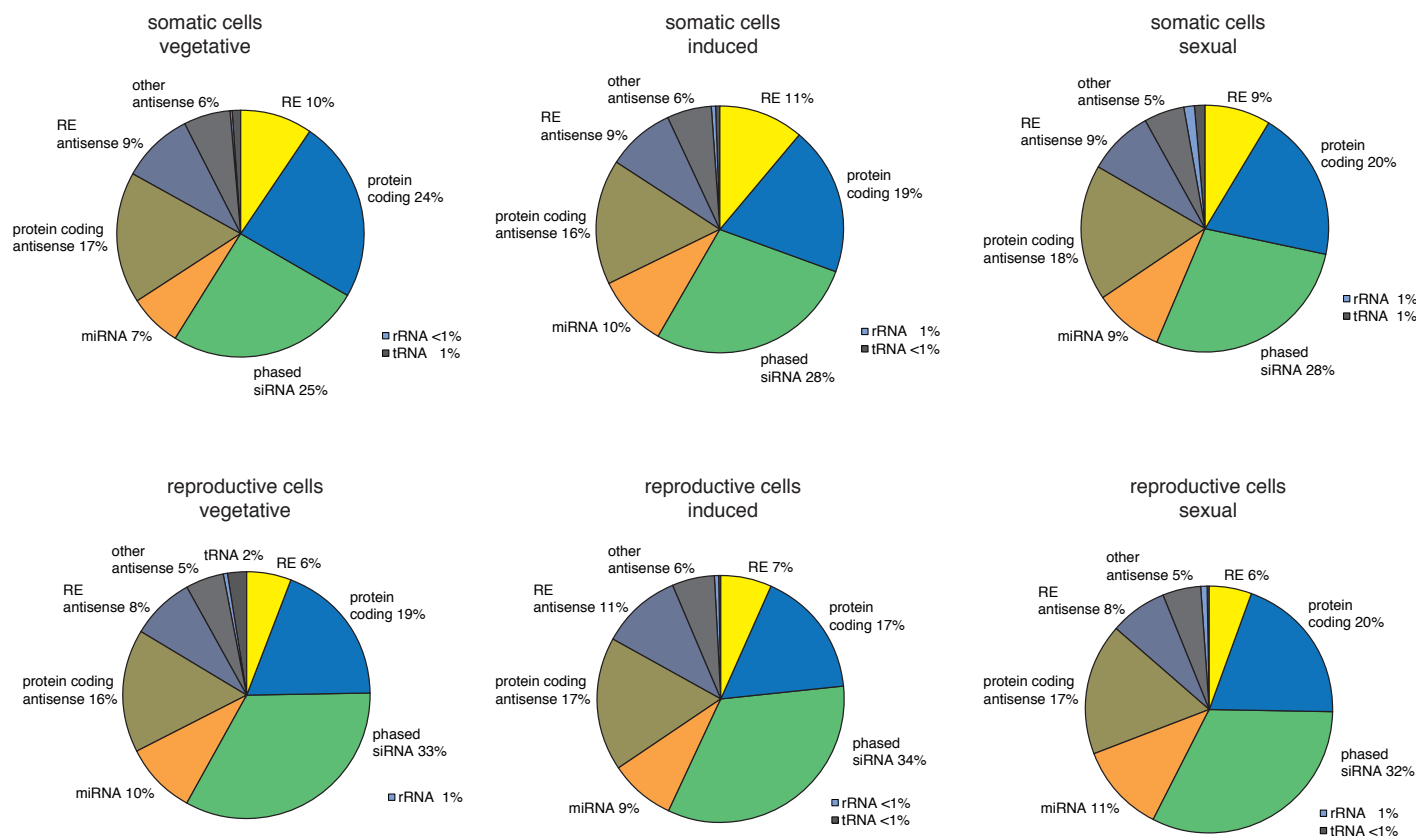

Supplement: Additional file 4: Figure S2. — Categorization of small RNA reads. (A) All six total small RNA libraries (vegetative somatic cells, induced somatic cells, female somatic cells, vegetative gonidia, induced gonidia and female egg cells were mapped according to Fig. 1E. In short, reads overlapping annotated exons of mRNA transcripts were assigned to the mRNA fraction. For the annotation of tRNAs and rRNAs, reads were mapped against the database entries for C. reinhardtii tRNAs and rRNAs from A. thaliana and the order Volvocales. Repeats were assigned using Repbase Update 19.02 [40], phased RNAs were predicted using the ta-si prediction tool from the UEA small RNA Workbench [41] which is based on the algorithm by Chen et al. [42]. MiRNAs were predicted using the tool miRA [19]. RE, repetitive elements. (B) Mapped reads from the libraries in (A) are shown according to Fig. 1F, i.e. categories are depicted without the putative intergenic reads. RE, repetitive elements. (PDF 42 kb) [file 12864_2016_3202_MOESM4_ESM.pdf]

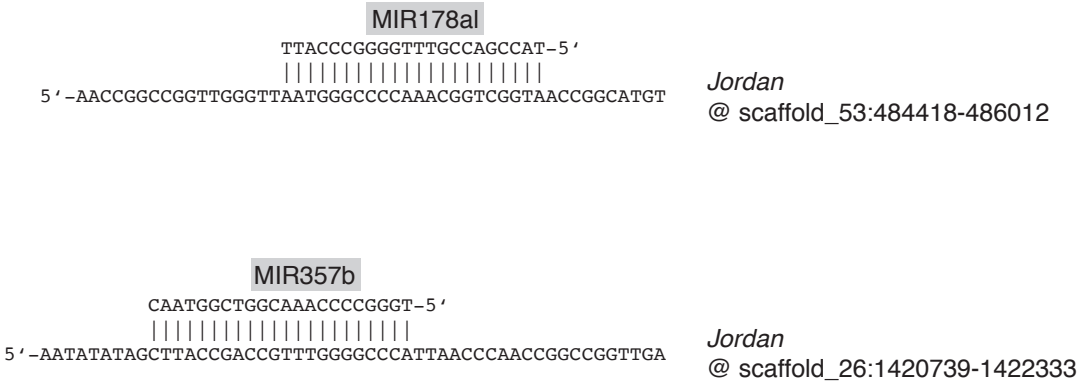

Supplement: Additional file 5: Figure S3. — MiRNA target sites on Jordan. Two exemplary target sites are shown, where a miRNA that originates from a Jordan element targets also a Jordan element. The miRNA is highlighted in grey and shown in 3′ to 5′ sequence. Part of the respective target sequence is shown below, lines indicate pairing of bases. The numbers on the right give the genome coordinates of the respective Jordan element. (PDF 36 kb) [file 12864_2016_3202_MOESM5_ESM.pdf]

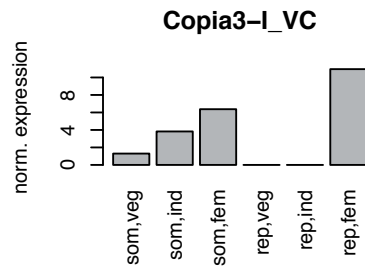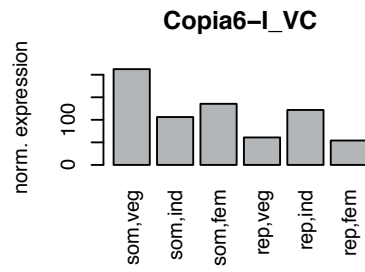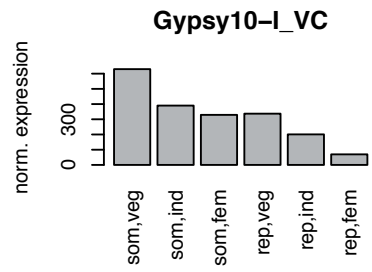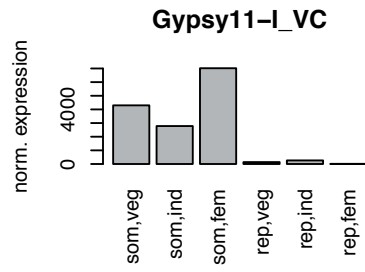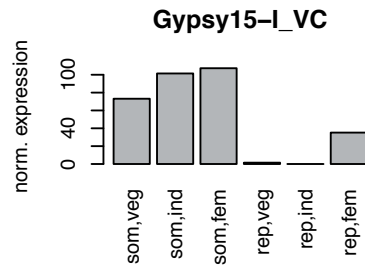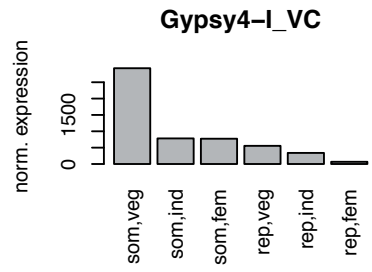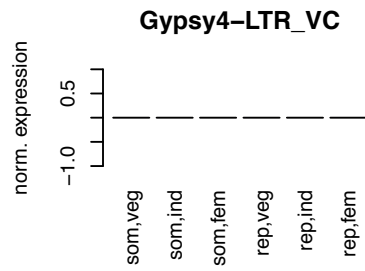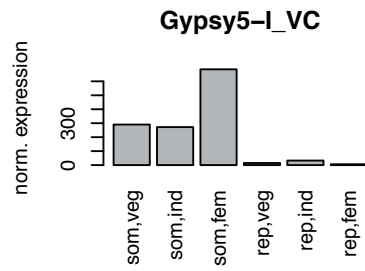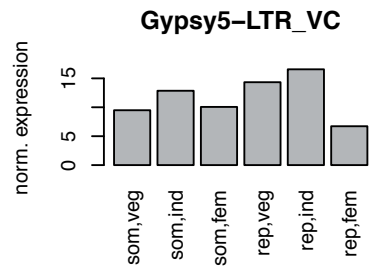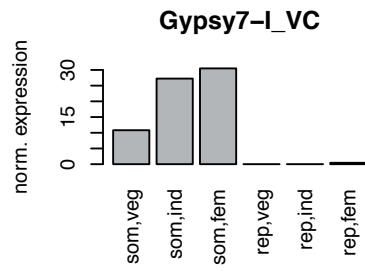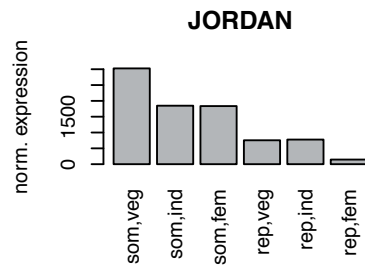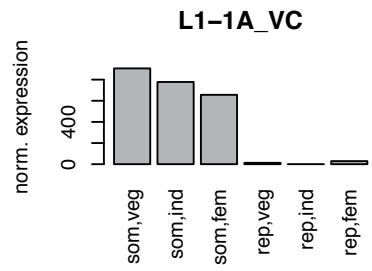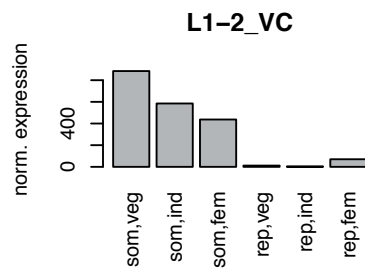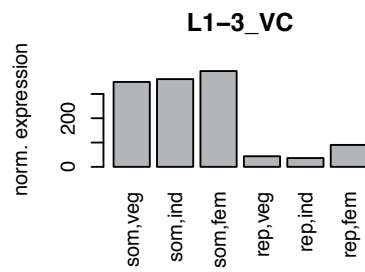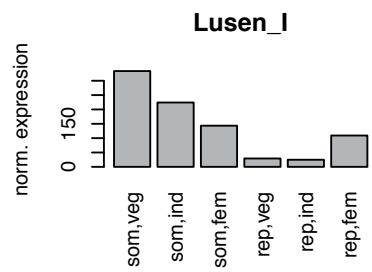

Supplement: Additional file 7: Figure S5. — Normalized expression of repetitive elements that are targeted by miRNAs. The diagrams show the normalized expression of the elements measured by RNA-Seq in the six conditions: Vegetative somatic cells (“som,veg”), induced somatic cells (“som,ind”), female somatic cells (“som,fem”), vegetative gonidia (“rep,veg”), induced gonidia (“rep,ind”) and female egg cells (“rep,fem”). (PDF 46 kb) [file 12864_2016_3202_MOESM7_ESM.pdf]

**B**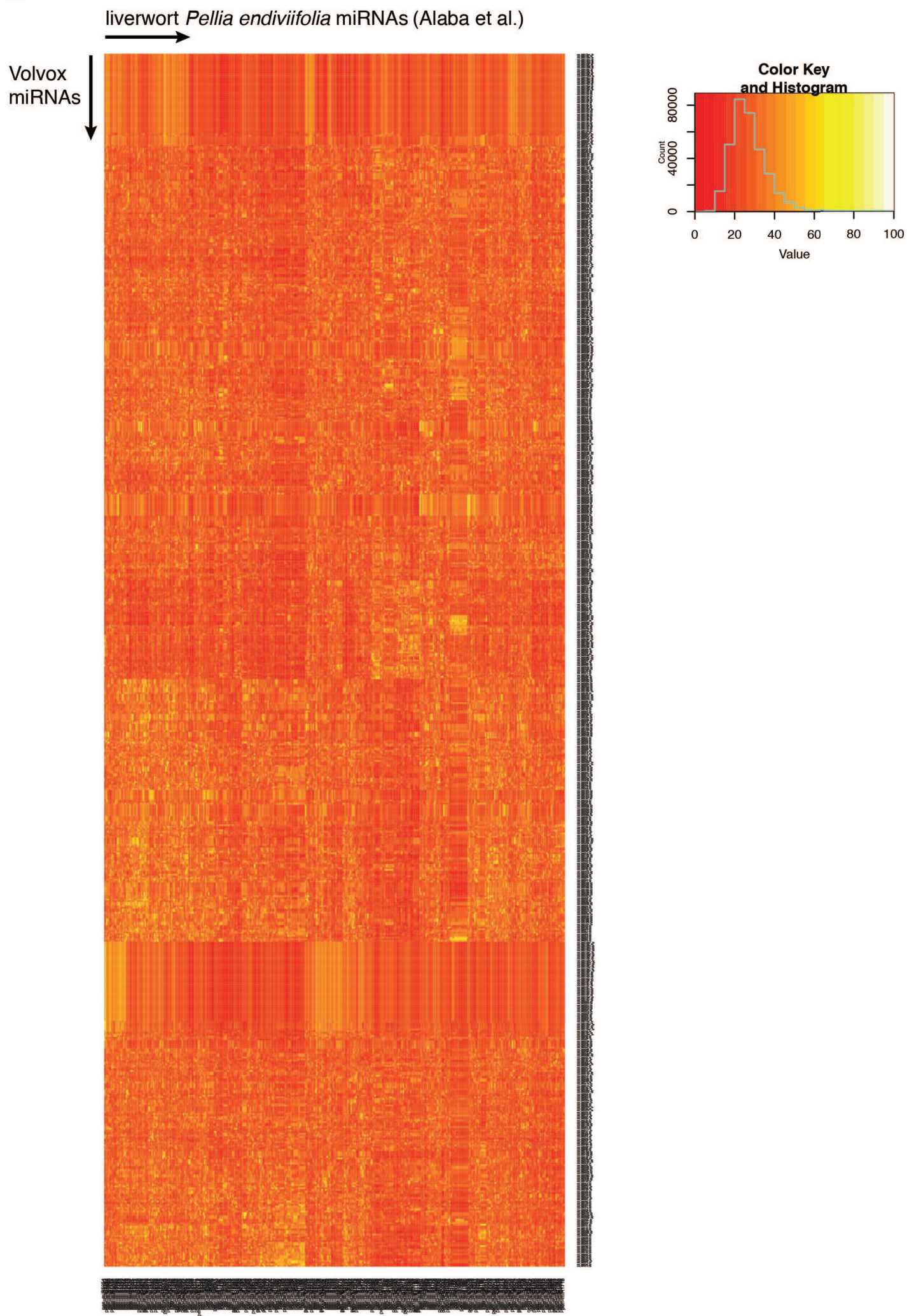

Supplement: Additional file 8: Figure S6. — Multiple sequence alignment of V. carteri miRNAs with (A) C. reinhardtii miRNAs or (B) miRNAs of the liverwort Pellia endiviifolia. The alignment was constructed using ClustalW [43]. The color code indicates the measure of similarity between the sequences. Red indicates no or low similarity, a light yellow or white shows a high similarity. The plot on the right depicts the frequencies of similarity over the whole data set. No sequence with conservation/high similarity could be found. (PDF 1889 kb) [file 12864_2016_3202_MOESM8_ESM.pdf]
