# Supplementary material for: Gene silencing pathways found in the green alga Volvox carteri reveal insights into evolution and origins of small RNA systems in plants
Source: BMC Genomics. 2016 Nov 2;17:853. doi: 10.1186/s12864-016-3202-4 (PMC5093975; doi:10.1186/s12864-016-3202-4)
Supplement: Additional file 3: Table S2. — Full list of identified miRNA sequences in this study. The table shows the location and sequence of each miRNA within the genome, also the position of the precursor (pre) is given. Since sometimes multiple miRNAs originated from one precursor, an assignment was made in order to be able to correctly pair and differentiate each mature and star sequence. (PDF 433 kb) [file 12864_2016_3202_MOESM3_ESM.pdf]

Additional file 3: Table S2. Full list of identified miRNA sequences in this study.

miRNA	Arm	Start	Stop	Chr	Strand	Start(pre)	Stop(pre)	Assignment	Sequence
MIR125	5p	4726198	4726219	scaffold_3	plus	4726163	4726380	mature1	AACCACCTGGAGCTGTGGATGG
MIR126	3p	805580	805600	scaffold_39	minus	805533	805783	mature1	AAGAAGTCCAACCCAGCGGCA
MIR127	5p	486015	486036	scaffold_53	minus	485815	486046	mature2	ACCGACCGTTTGGGGCCCATTA
MIR128	3p	3611562	3611581	scaffold_5	minus	3611556	3611879	mature3	ACGCATAGACATAGACATGT
MIR129	5p	1495329	1495349	scaffold_3	plus	1495303	1495399	mature1	ACGGGTTCACCAGGTTGGTCA
MIR130	3p	10194	10214	scaffold_3	plus	10041	10233	mature1	AGCAGCGGTACACGACCCGGT
MIR130*	5p	10063	10082	scaffold_3	plus	10041	10233	star1	TCGGTCGTATATCGCTGCTC
MIR131	5p	343927	343948	scaffold_62	plus	343925	343990	mature1	AGGGATTGGGTTCGGGTACCGG
MIR132	3p	7202337	7202360	scaffold_1	minus	7202286	7202667	mature3	AGTAGACGAGAACGCACTCAGGAA
MIR133	5p	110377	110398	scaffold_81	minus	110176	110519	mature2	ATCTTCGTAGGCCATGAAGGCC
MIR134	3p	1279127	1279147	scaffold_27	minus	1279066	1279381	mature1	ATGGAAATGCCGACTTGGGTT
MIR134*	5p	1279334	1279355	scaffold_27	minus	1279066	1279381	star1	ACCCAAGTCGGCATTTCCATGC
MIR135a	3p	31072	31093	scaffold_15	minus	31055	31256	mature1	ATGGCGACGGTGGCAGTGGCGA
MIR135a*	5p	31220	31240	scaffold_15	minus	31055	31256	star1	TCGCCATTGCTGCCGTCGCCA
MIR135b	3p	102568	102589	scaffold_50	minus	102566	102675	mature1	ATGGCGACGGTGGCAGTGGCGA
MIR135b*	5p	102649	102669	scaffold_50	minus	102566	102675	star1	TGCCGCTGTCGCCATCCCTGC
MIR135c	3p	468240	468261	scaffold_38	minus	468202	468446	mature1	ATGGCGACGGTGGCAGTGGCGA
MIR135c*	5p	468388	468408	scaffold_38	minus	468202	468446	star1	TCGCCATTGCCGCCGTCGCCA
MIR136	3p	4726355	4726375	scaffold_3	plus	4726163	4726380	mature2	CAAATCTCTGCAAGGCACGGA
MIR137	5p	366807	366827	scaffold_67	minus	366629	366893	mature1	CATGTGGATGCTGTATTGTCA
MIR137*	3p	366695	366716	scaffold_67	minus	366629	366893	star1	TGACAATACAGCATCCACATGT
MIR138	3p	275506	275526	scaffold_38	plus	275353	275551	mature1	CCAAACGGTCGGTAACCGGCA
MIR138*	5p	275371	275391	scaffold_38	plus	275353	275551	star1	TAAACATGCCGGTTACCGACC
MIR139	3p	150722	150743	scaffold_74	minus	150629	150848	mature2	CCATGGCGCTCGTGACGGAGGT
MIR140	3p	1326333	1326355	scaffold_2	minus	1326323	1326644	mature2	CCCACGACATCGGCCAGCTGGTT
MIR141	3p	2460	2481	scaffold_966	plus	2310	2517	mature1	CCCCTTTAGGGGAAGAAGGGGA
MIR142a	5p	953294	953315	scaffold_38	plus	953249	953383	mature1	CCCTAAAGGGGAACTAAAGGGG
MIR142a*	5p	953261	953283	scaffold_38	plus	953249	953383	star1	CCCTTTGGTTCCCCTTTAGGGGA
MIR142b	3p	7444	7466	scaffold_80	minus	7432	7506	mature1	CCCTAAAGGGGAACTAAAGGGGA
MIR142b*	5p	7477	7499	scaffold_80	minus	7432	7506	star1	CCCTTTGGTTCCCCTTTAGGGGA
MIR142c	3p	1055790	1055812	scaffold_19	plus	1055740	1055860	mature1	CCCTAAAGGGGAACTAAAGGGGA
MIR143	5p	75172	75192	scaffold_11	plus	75139	75230	mature1	CCCTTTAGGGGAAGAAGGGGA
MIR144	3p	10309626	10309653	scaffold_1	plus	10309579	10309660	mature1	CCCTTTAGGGGATGAAGAACTAAAGGGG
MIR145	3p	10309885	10309907	scaffold_1	plus	10309809	10309921	mature1	CCCTTTAGTTCCCCTTTAGGGGA
MIR145*	5p	10309828	10309850	scaffold_1	plus	10309809	10309921	star1	AGGGGACGAACACTAAAGGGGAC
MIR146	5p	219373	219395	scaffold_54	minus	219336	219400	mature1	CCCTTTGGTTCCCCTAAAGGGGA
MIR147	5p	1901494	1901515	scaffold_20	minus	1901332	1901534	mature2	CCGGTTACCGACCGTTCGGGGC
MIR148	5p	1697868	1697889	scaffold_3	plus	1697828	1698039	mature1	CGAAACTAGTCGCAGTGCTTGT
MIR148*	3p	1697981	1698001	scaffold_3	plus	1697828	1698039	star1	CAAGCACTGCGACTAGTTTCG
MIR149	3p	3611656	3611677	scaffold_5	minus	3611556	3611879	mature2	CGGAAGAAACCTTTGCAGCATT
MIR150	3p	1901335	1901356	scaffold_20	minus	1901332	1901534	mature1	CGGCATGTTTATGCCATAGGGC
MIR151	5p	12358	12380	scaffold_18	minus	12152	12423	mature2	CTGTACGCGAGCACCCACCTGGC
MIR152	5p	169276	169295	scaffold_28	plus	169217	169357	mature1	CTGTCTGAGCATAATAAGCT
MIR153	5p	2517150	2517169	scaffold_17	minus	2517118	2517176	mature1	CTGTCTGAGCATAATTTTAG
MIR154	3p	379961	379981	scaffold_8	minus	379870	380165	mature2	GAAATAGAATGGACTCCGTTT
MIR155a	5p	21735	21757	scaffold_39	plus	21702	22029	mature1	GCAGGGTAGGCTCCGGCAGTGCC
MIR155b	5p	476082	476104	scaffold_17	plus	476049	476360	mature1	GCAGGGTAGGCTCCGGCAGTGCC
MIR156	3p	1397467	1397486	scaffold_31	minus	1397432	1397636	mature1	GGAGGGCGGTGTTGGGCTGG
MIR157	5p	63843	63866	scaffold_85	minus	63583	63928	mature2	GGCGGCTGGACAGGATTGTCCGCT
MIR158	3p	1728500	1728522	scaffold_27	plus	1728277	1728550	mature2	GTGGGGAGCCGTCTGCACAACTC
MIR158*	5p	1728309	1728329	scaffold_27	plus	1728277	1728550	star2	TTGTGCAGACGGCTCCCCACC
MIR159	3p	3826986	3827005	scaffold_5	plus	3826655	3827079	mature1	TAAAAGAAGAGATCGTGGGG
MIR159*	5p	3826732	3826752	scaffold_5	plus	3826655	3827079	star1	CCACGATCTCTTCTTTTAGCT
MIR160	3p	324188	324209	scaffold_51	plus	323928	324247	mature1	TAAAAGACTCGCTAACCTGAGG
MIR160*	5p	323969	323991	scaffold_51	plus	323928	324247	star1	TCAGGTTAGCGAGTCTTTTAGCA
MIR161	5p	326778	326798	scaffold_23	plus	326756	327011	mature1	TAAACATGCCGGTTACCGACC
MIR162	5p	238423	238445	scaffold_13	plus	238352	238683	mature2	TAAACTTGCAGGACGCTAACCCT
MIR163	3p	1809887	1809908	scaffold_27	minus	1809869	1810180	mature1	TAAAGGGACTCCGTACGCGAAA
MIR163*	5p	1810140	1810161	scaffold_27	minus	1809869	1810180	star1	TCGCGTACGGAGCCCCTTTACG
MIR164a	5p	626328	626348	scaffold_7	minus	626265	626356	mature1	TAACAAGGCGTGTGGGGCTCC
MIR164b	5p	172821	172841	scaffold_1	minus	172757	172847	mature1	TAACAAGGCGTGTGGGGCTCC
MIR164c	5p	285557	285577	scaffold_60	minus	285442	285585	mature1	TAACAAGGCGTGTGGGGCTCC
MIR164d	5p	1439653	1439673	scaffold_29	minus	1439549	1439679	mature1	TAACAAGGCGTGTGGGGCTCC
MIR165	3p	283792	283811	scaffold_55	plus	283509	283879	mature1	TAACCAGGTCGGAGCGAAGC
MIR165*	5p	283631	283650	scaffold_55	plus	283509	283879	star1	TTCGCTCCGACCTGGTTACT
MIR166	3p	552290	552310	scaffold_40	minus	552236	552619	mature1	TAACGACGTCGTGAGTTTACT
MIR166*	5p	552437	552459	scaffold_40	minus	552236	552619	star1	TAAACTCATGACGTCGCTTTAAG
MIR167a	3p	122671	122690	scaffold_80	plus	122573	122706	mature1	TAACGGACAGCATAGGACGG
MIR167b	3p	1084772	1084791	scaffold_4	minus	1084755	1084836	mature1	TAACGGACAGCATAGGACGG
MIR167c	3p	656905	656924	scaffold_9	plus	656859	656940	mature1	TAACGGACAGCATAGGACGG
MIR167d	3p	1462634	1462653	scaffold_7	plus	1462588	1462669	mature1	TAACGGACAGCATAGGACGG
MIR167e	3p	1462605	1462624	scaffold_7	minus	1462585	1462672	mature1	TAACGGACAGCATAGGACGG
MIR167f	3p	656876	656895	scaffold_9	minus	656859	656940	mature1	TAACGGACAGCATAGGACGG
MIR168	3p	239481	239502	scaffold_47	minus	239480	239887	mature2	TAACGGCCTGCTTGCAAGACAC
MIR168*	5p	239862	239883	scaffold_47	minus	239480	239887	star2	TCTTGCAAGCAGGCCGTTAGTG

MIR169	3p	14115811	14115833	scaffold_1	minus	14115752	14115955	mature1	TAACGGTCGGTTGGGTTAATGGG
MIR170	3p	239511	239533	scaffold_47	minus	239480	239887	mature1	TAAGAGACTTGGTCTACACATGC
MIR170*	5p	239837	239858	scaffold_47	minus	239480	239887	star1	TAGCATGTGTAGACCAAGTCTC
MIR171	5p	469947	469969	scaffold_38	minus	469890	470021	mature1	TAAGCCAAGCCGTCTCAAGACCC
MIR172	3p	125051	125071	scaffold_58	minus	125033	125161	mature1	TAAGCTTGTGAGTTGGTGGCC
MIR173a	5p	972574	972594	scaffold_8	plus	972518	972804	mature1	TAATATGGGTTGGAGTTGGGC
MIR173b	5p	972730	972750	scaffold_8	minus	972518	972805	mature1	TAATATGGGTTGGAGTTGGGC
MIR174	3p	515656	515676	scaffold_44	minus	515628	515796	mature1	TAATGCCAAAGGACGTGTGGC
MIR175a	3p	1815827	1815847	scaffold_13	plus	1815684	1815876	mature1	TACAGTCGAGTACGGCACTTC
MIR175a*	5p	1815718	1815736	scaffold_13	plus	1815684	1815876	star1	TGCCGTACTCGACTGTACC
MIR175b	3p	1815714	1815734	scaffold_13	minus	1815684	1815876	mature1	TACAGTCGAGTACGGCACTTC
MIR175b*	5p	1815825	1815843	scaffold_13	minus	1815684	1815876	star1	TGCCGTACTCGACTGTACC
MIR176	5p	646274	646296	scaffold_6	minus	646139	646300	mature1	TACAGTCTCCCCCCTCTGAAGCG
MIR177	5p	114085	114105	scaffold_49	minus	113928	114160	mature1	TACCCAGGAATGTGTTGCTCA
MIR178a	5p	1420751	1420772	scaffold_26	plus	1420750	1420919	mature1	TACCGACCGTTTGGGGCCCATT
MIR178a*	3p	1420883	1420904	scaffold_26	plus	1420750	1420919	star1	TGGGCCCCAAACGGTCGGTAAC
MIR178aa	5p	125131	125152	scaffold_15	minus	124943	125208	mature1	TACCGACCGTTTGGGGCCCATT
MIR178aa*	3p	124998	125019	scaffold_15	minus	124943	125208	star1	TGGGCCCCAAACGGTCGGTAAC
MIR178ab	5p	257016	257037	scaffold_47	plus	256973	257190	mature1	TACCGACCGTTTGGGGCCCATT
MIR178ab*	3p	257148	257169	scaffold_47	plus	256973	257190	star1	TGGGCCCCAAACGGTCGGTAAC
MIR178ac	5p	76066	76087	scaffold_95	minus	75912	76110	mature1	TACCGACCGTTTGGGGCCCATT
MIR178ac*	3p	75934	75955	scaffold_95	minus	75912	76110	star1	TGGGCCCCAAACGGTCGGTAAC
MIR178ad	5p	1996409	1996430	scaffold_14	minus	1996255	1996453	mature1	TACCGACCGTTTGGGGCCCATT
MIR178ad*	3p	1996277	1996298	scaffold_14	minus	1996255	1996453	star1	TGGGCCCCAAACGGTCGGTAAC
MIR178ae	5p	8480	8501	scaffold_141	plus	8454	8640	mature1	TACCGACCGTTTGGGGCCCATT
MIR178ae*	3p	8596	8617	scaffold_141	plus	8454	8640	star1	TGGGCCCCAAACGGTCGGTAAC
MIR178af	5p	11645	11666	scaffold_115	plus	11621	11803	mature1	TACCGACCGTTTGGGGCCCATT
MIR178af*	3p	11761	11782	scaffold_115	plus	11621	11803	star1	TGGGCCCCAAACGGTCGGTAAC
MIR178ag	5p	452131	452152	scaffold_31	plus	452107	452305	mature1	TACCGACCGTTTGGGGCCCATT
MIR178ag*	3p	452263	452284	scaffold_31	plus	452107	452305	star1	TGGGCCCCAAACGGTCGGTAAC
MIR178ah	5p	1996279	1996300	scaffold_14	plus	1996255	1996453	mature1	TACCGACCGTTTGGGGCCCATT
MIR178ah*	3p	1996411	1996432	scaffold_14	plus	1996255	1996453	star1	TGGGCCCCAAACGGTCGGTAAC
MIR178ai	5p	692129	692150	scaffold_31	minus	691973	692175	mature1	TACCGACCGTTTGGGGCCCATT
MIR178ai*	3p	691997	692018	scaffold_31	minus	691973	692175	star1	TGGGCCCCAAACGGTCGGTAAC
MIR178aj	5p	2823600	2823621	scaffold_14	minus	2823446	2823644	mature1	TACCGACCGTTTGGGGCCCATT
MIR178aj*	3p	2823468	2823489	scaffold_14	minus	2823446	2823644	star1	TGGGCCCCAAACGGTCGGTAAC
MIR178ak	5p	2215802	2215823	scaffold_20	minus	2215648	2215846	mature1	TACCGACCGTTTGGGGCCCATT
MIR178ak*	3p	2215670	2215691	scaffold_20	minus	2215648	2215846	star1	TGGGCCCCAAACGGTCGGTAAC
MIR178al	5p	340368	340389	scaffold_62	minus	340214	340412	mature1	TACCGACCGTTTGGGGCCCATT
MIR178al*	3p	340236	340257	scaffold_62	minus	340214	340412	star1	TGGGCCCCAAATGGTCGGTAAC
MIR178am	5p	98374	98395	scaffold_42	plus	98367	98531	mature1	TACCGACCGTTTGGGGCCCATT
MIR178am*	3p	98506	98527	scaffold_42	plus	98367	98531	star1	TGGGCCCCAAACGGTCGGTAAC
MIR178an	5p	117275	117296	scaffold_84	minus	117121	117319	mature1	TACCGACCGTTTGGGGCCCATT
MIR178an*	3p	117143	117164	scaffold_84	minus	117121	117319	star1	TGAGCCCCAAACGGTCGGTAAC
MIR178ao	5p	2924148	2924169	scaffold_11	plus	2924122	2924325	mature1	TACCGACCGTTTGGGGCCCATT
MIR178ao*	3p	2924281	2924302	scaffold_11	plus	2924122	2924325	star1	TGGGCCCCAAACGGTCGGTAAC
MIR178ap	5p	21956	21977	scaffold_95	minus	21818	22000	mature1	TACCGACCGTTTGGGGCCCATT
MIR178aq	5p	2513212	2513233	scaffold_11	minus	2513058	2513256	mature1	TACCGACCGTTTGGGGCCCATT
MIR178aq*	3p	2513080	2513101	scaffold_11	minus	2513058	2513256	star1	TGCGCCCCAAACGGTCGGTAAC
MIR178ar	5p	68124	68145	scaffold_100	minus	67970	68168	mature1	TACCGACCGTTTGGGGCCCATT
MIR178ar*	3p	67992	68013	scaffold_100	minus	67970	68168	star1	TGGGCCCCAAACGGTCGGTAAC
MIR178as	5p	8594	8615	scaffold_141	minus	8437	8640	mature1	TACCGACCGTTTGGGGCCCATT
MIR178as*	3p	8478	8499	scaffold_141	minus	8437	8640	star1	TGGGCCCCAAACGGTCGGTAAC
MIR178at	5p	103309	103330	scaffold_14	minus	103105	103404	mature1	TACCGACCGTTTGGGGCCCATT
MIR178at*	3p	103178	103199	scaffold_14	minus	103105	103404	star1	TGGGCCCCAAACGGTCGGTAAC
MIR178au	5p	125000	125021	scaffold_15	plus	124912	125239	mature1	TACCGACCGTTTGGGGCCCATT
MIR178au*	3p	125133	125154	scaffold_15	plus	124912	125239	star1	TGGGCCCCAAACGGTCGGTAAC
MIR178b	5p	218352	218373	scaffold_60	minus	218198	218396	mature1	TACCGACCGTTTGGGGCCCATT
MIR178b*	3p	218220	218241	scaffold_60	minus	218198	218396	star1	TGGGCCCCAAACGGTCGGTAAC
MIR178c	5p	411377	411398	scaffold_31	plus	411353	411535	mature1	TACCGACCGTTTGGGGCCCATT
MIR178c*	3p	411493	411514	scaffold_31	plus	411353	411535	star1	TGGGCCCCAAACGGTCGGTAAC
MIR178d	5p	568536	568557	scaffold_27	minus	568379	568580	mature1	TACCGACCGTTTGGGGCCCATT
MIR178d*	3p	568420	568441	scaffold_27	minus	568379	568580	star1	TGGGCCCCAAACGGTCGGTAAC
MIR178e	5p	86816	86837	scaffold_5	minus	86659	86860	mature1	TACCGACCGTTTGGGGCCCATT
MIR178e*	3p	86700	86721	scaffold_5	minus	86659	86860	star1	TGGGCCCCAAACGGTCGGTAAC
MIR178f	5p	1036211	1036232	scaffold_11	minus	1036057	1036255	mature1	TACCGACCGTTTGGGGCCCATT
MIR178f*	3p	1036079	1036100	scaffold_11	minus	1036057	1036255	star1	TGGGCCCCAAACAGTCGGTAAC
MIR178g	5p	691999	692020	scaffold_31	plus	691973	692175	mature1	TACCGACCGTTTGGGGCCCATT
MIR178g*	3p	692131	692152	scaffold_31	plus	691973	692175	star1	TGGGCCCCAAACGGTCGGTAAC
MIR178h	5p	67994	68015	scaffold_100	plus	67970	68168	mature1	TACCGACCGTTTGGGGCCCATT
MIR178h*	3p	68126	68147	scaffold_100	plus	67970	68168	star1	TGGGCCCCAAACGGTCGGTAAC
MIR178i	5p	411491	411512	scaffold_31	minus	411353	411535	mature1	TACCGACCGTTTGGGGCCCATT
MIR178i*	3p	411375	411396	scaffold_31	minus	411353	411535	star1	TGGGCCCCAAACGGTCGGTAAC
MIR178j	5p	86702	86723	scaffold_5	plus	86659	86860	mature1	TACCGACCGTTTGGGGCCCATT
MIR178j*	3p	86818	86839	scaffold_5	plus	86659	86860	star1	TGGGCCCCAAACGGTCGGTAAC
MIR178k	5p	568422	568443	scaffold_27	plus	568398	568580	mature1	TACCGACCGTTTGGGGCCCATT
MIR178k*	3p	568538	568559	scaffold_27	plus	568398	568580	star1	TGGGCCCCAAACGGTCGGTAAC

MIR178l	5p	218222	218243	scaffold_60	plus	218198	218396	mature1	TACCGACCGTTTGGGGCCCATT
MIR178l*	3p	218354	218375	scaffold_60	plus	218198	218396	star1	TGGGCCCCAAACGGTCGGTAAC
MIR178m	5p	84935	84956	scaffold_47	minus	84781	84979	mature1	TACCGACCGTTTGGGGCCCATT
MIR178n	5p	75936	75957	scaffold_95	plus	75912	76110	mature1	TACCGACCGTTTGGGGCCCATT
MIR178n*	3p	76068	76089	scaffold_95	plus	75912	76110	star1	TGGGCCCCAAACGGTCGGTAAC
MIR178o	5p	2924279	2924300	scaffold_11	minus	2924105	2924325	mature1	TACCGACCGTTTGGGGCCCATT
MIR178o*	3p	2924146	2924167	scaffold_11	minus	2924105	2924325	star1	TGGGCCCCAAACGGTCGGTAAC
MIR178p	5p	2215672	2215693	scaffold_20	plus	2215648	2215847	mature1	TACCGACCGTTTGGGGCCCATT
MIR178p*	3p	2215804	2215825	scaffold_20	plus	2215648	2215847	star1	TGGGCCCCAAACGGTCGGTAAC
MIR178q	5p	98504	98525	scaffold_42	minus	98365	98533	mature1	TACCGACCGTTTGGGGCCCATT
MIR178q*	3p	98372	98393	scaffold_42	minus	98365	98533	star1	TGGGCCCCAAACGGTCGGTAAC
MIR178r	5p	486016	486037	scaffold_53	minus	485815	486046	mature1	TACCGACCGTTTGGGGCCCATT
MIR178s	5p	255521	255542	scaffold_68	plus	255497	255695	mature1	TACCGACCGTTTGGGGCCCATT
MIR178s*	3p	255653	255674	scaffold_68	plus	255497	255695	star1	TGAGCCCCAAACGGTCGGTAAC
MIR178t	5p	452261	452282	scaffold_31	minus	452107	452305	mature1	TACCGACCGTTTGGGGCCCATT
MIR178t*	3p	452129	452150	scaffold_31	minus	452107	452305	star1	TGGGCCCCAAACGGTCGGTAAC
MIR178u	5p	3120029	3120050	scaffold_1	plus	3120005	3120203	mature1	TACCGACCGTTTGGGGCCCATT
MIR178u*	3p	3120161	3120182	scaffold_1	plus	3120005	3120203	star1	TGAGCCCCAAACGGTCGGTAAC
MIR178v	5p	28980	29001	scaffold_95	plus	28956	29154	mature1	TACCGACCGTTTGGGGCCCATT
MIR178v*	3p	29112	29133	scaffold_95	plus	28956	29154	star1	TGAGCCCCAAACGGTCGGTAAC
MIR178w	5p	2823470	2823491	scaffold_14	plus	2823446	2823644	mature1	TACCGACCGTTTGGGGCCCATT
MIR178w*	3p	2823602	2823623	scaffold_14	plus	2823446	2823644	star1	TGGGCCCCAAACGGTCGGTAAC
MIR178x	5p	257146	257167	scaffold_47	minus	256992	257190	mature1	TACCGACCGTTTGGGGCCCATT
MIR178x*	3p	257014	257035	scaffold_47	minus	256992	257190	star1	TGGGCCCCAAACGGTCGGTAAC
MIR178y	5p	117259	117280	scaffold_23	minus	117104	117303	mature1	TACCGACCGTTTGGGGCCCATT
MIR178y*	3p	117127	117148	scaffold_23	minus	117104	117303	star1	TGGTCCCCAAACGGTCGGTAAC
MIR178z	5p	11759	11780	scaffold_115	minus	11621	11803	mature1	TACCGACCGTTTGGGGCCCATT
MIR178z*	3p	11643	11664	scaffold_115	minus	11621	11803	star1	TGGGCCCCAAACGGTCGGTAAC
MIR179a	5p	37219	37240	scaffold_72	minus	37065	37263	mature1	TACCGACCGTTTGGGGCTCATT
MIR180	3p	1960101	1960123	scaffold_11	minus	1960030	1960325	mature1	TACCGATCCATCCTAAGATGAGA
MIR181	5p	4061796	4061817	scaffold_1	plus	4061756	4062016	mature1	TACCGATCCTTCCTGGTGCTAG
MIR182	5p	46259	46279	scaffold_86	minus	46134	46314	mature1	TACCGATTACTGGGGACGGTT
MIR183	3p	1547438	1547458	scaffold_18	plus	1547226	1547549	mature2	TACCGCTGGACAGCATCTCGT
MIR184	5p	278395	278416	scaffold_63	plus	278359	278625	mature2	TACCGGAAGACAGAAATAGACC
MIR185a	3p	1356152	1356174	scaffold_18	minus	1356052	1356368	mature2	TACGGGTGTACGGATGGAGGAGC
MIR185a*	3p	1356151	1356173	scaffold_18	minus	1356052	1356368	star2	ACGGGTGTACGGATGGAGGAGCT
MIR185b	3p	1356247	1356269	scaffold_18	plus	1356052	1356368	mature2	TACGGGTGTACGGATGGAGGAGC
MIR186	3p	71874	71894	scaffold_24	minus	71871	72120	mature1	TACGTCATCCATCGCCCTGTA
MIR186*	5p	72088	72107	scaffold_24	minus	71871	72120	star1	TGGATGACGTACAACAAAGG
MIR187	5p	6687	6707	scaffold_321	minus	6541	6736	mature2	TACGTGACTCATCAATGAGCT
MIR187*	3p	6542	6565	scaffold_321	minus	6541	6736	star1	TGGAAGCTGGGTCTGGAGGGCATG
MIR188a	3p	1091753	1091774	scaffold_25	plus	1091660	1091795	mature1	TACTGACCCGGTACGACTTGGA
MIR188b	3p	64438	64459	scaffold_68	plus	64345	64480	mature1	TACTGACCCGGTACGACTTGGA
MIR189	3p	2393268	2393289	scaffold_16	minus	2393261	2393390	mature1	TACTGGGTCGCTACTGGGTCGC
MIR190	5p	116303	116323	scaffold_12	minus	115981	116328	mature1	TAGAAAGGGGATTAGTGCACC
MIR190*	3p	115983	116004	scaffold_12	minus	115981	116328	star1	TGCACTAATCCCCTTTCTACCC
MIR191a	5p	2206742	2206763	scaffold_8	plus	2206685	2206889	mature1	TAGACGAGGAACTTGCCGAAGC
MIR191a*	3p	2206838	2206859	scaffold_8	plus	2206685	2206889	star1	TTCGGCAAGTTCCTCGTCTAGT
MIR191b	5p	2206836	2206857	scaffold_8	minus	2206685	2206890	mature1	TAGACGAGGAACTTGCCGAAGC
MIR191b*	3p	2206740	2206761	scaffold_8	minus	2206685	2206890	star1	TTCGGCAAGTTCCTCGTCTAGT
MIR192	3p	709607	709629	scaffold_12	plus	709380	709663	mature2	TAGACTTTTAGTCGGCTGCATCT
MIR193a	5p	169407	169428	scaffold_84	plus	169364	169612	mature1	TAGCAACTGGACTAGTTACTCC
MIR193a*	3p	169560	169581	scaffold_84	plus	169364	169612	star1	TAGTCCAGTTGCTAGGGGGGGG
MIR193b	5p	1699962	1699983	scaffold_26	plus	1699922	1700209	mature1	TAGCAACTGGACTAGTTACTCC
MIR193b*	3p	1700159	1700180	scaffold_26	plus	1699922	1700209	star1	TAGTCCAGTTGCTAGGGGGGGG
MIR193c	5p	84353	84374	scaffold_69	plus	84313	84590	mature1	TAGCAACTGGACTAGTTACTCC
MIR193c*	3p	84539	84560	scaffold_69	plus	84313	84590	star1	TAGTCCAGTTGCTAGGGGGGGG
MIR194	5p	176368	176389	scaffold_40	plus	176345	176593	mature2	TAGCCCCGTCACTGCCGTTATT
MIR195	3p	123893	123913	scaffold_51	minus	123877	124081	mature1	TAGCGATGAGAGGAAAGAGGC
MIR195*	5p	124042	124063	scaffold_51	minus	123877	124081	star1	TCTTTCCTCTCATCGCTACAGC
MIR196a	3p	1356130	1356151	scaffold_18	minus	1356052	1356368	mature1	TAGGACTCGGGACTGCATGAAC
MIR196a*	5p	1356268	1356289	scaffold_18	minus	1356052	1356368	star1	TCATGCAGTCCCGAGTCCTAGC
MIR196b	3p	1356270	1356291	scaffold_18	plus	1356052	1356368	mature1	TAGGACTCGGGACTGCATGAAC
MIR196b*	5p	1356132	1356153	scaffold_18	plus	1356052	1356368	star1	TCATGCAGTCCCGAGTCCTAGC
MIR197a	3p	20807	20829	scaffold_73	minus	20762	20908	mature1	TAGGGTGACTGGATGAGGGTGCC
MIR197a*	3p	20795	20814	scaffold_73	minus	20762	20908	star1	AGGGTGCCGCGAAATACCCG
MIR197b	3p	1244603	1244625	scaffold_13	minus	1244562	1244720	mature1	TAGGGTGACTGGATGAGGGTGCC
MIR198	5p	1276796	1276816	scaffold_23	minus	1276735	1276850	mature1	TAGGGTTAATGTAGGTGCGGG
MIR199	3p	226344	226365	scaffold_20	minus	226331	226446	mature1	TAGGTTAGGCACTACTGGTAGA
MIR200	5p	531757	531778	scaffold_43	plus	531715	531910	mature1	TAGTACGCGTGCATTCGTTAGG
MIR200*	3p	531851	531871	scaffold_43	plus	531715	531910	star1	TAACGAATGCACGCGTACTAT
MIR201	3p	7202339	7202361	scaffold_1	minus	7202286	7202667	mature1	TAGTAGACGAGAACGCACTCAGG
MIR201*	5p	7202590	7202612	scaffold_1	minus	7202286	7202667	star1	TGAGTGCGTTCTCGTCTACTACG
MIR202	3p	199862	199882	scaffold_16	minus	199852	200113	mature1	TAGTCAGGTCCGCCGGCGGCA
MIR202*	3p	985651	985672	scaffold_16	minus	985594	985886	star1	TGCACTGTCTTGATCCGCCAGG
MIR203a	3p	284173	284194	scaffold_69	plus	284024	284225	mature1	TAGTCCAGTTGCTAGGGGGGGG
MIR203a*	5p	284061	284083	scaffold_69	plus	284024	284225	star1	CCCTTAGAAACTGGACTAGTAGC

MIR204	5p	1969966	1969988	scaffold_12	plus	1969908	1970111	mature1	TAGTCTCCGCCTGGCTGTTCACT
MIR204*	3p	1970053	1970072	scaffold_12	plus	1969908	1970111	star1	TGAACAGCCAGGCGGAGACT
MIR205	5p	1842371	1842392	scaffold_22	plus	1842233	1842611	mature1	TAGTTTATAACATTGGGTCGCC
MIR206	5p	1597988	1598008	scaffold_24	minus	1597782	1598074	mature2	TATACAAAACGCACAGACAGA
MIR207	5p	518665	518686	scaffold_25	minus	518459	518711	mature1	TATACCGACCGGCGGATGCAGA
MIR207*	3p	518483	518504	scaffold_25	minus	518459	518711	star1	TGCATCCGCCGGTCGGTGTACT
MIR208	5p	104832	104854	scaffold_78	plus	104774	105084	mature2	TATATAGTAAACTCTTGGAAAGG
MIR209	3p	1547334	1547353	scaffold_18	minus	1547266	1547511	mature1	TATCGCTGGACAGCATCTCG
MIR210	3p	964507	964528	scaffold_26	minus	964491	964684	mature1	TATCTCTGTAGAACAAGAAGCA
MIR210*	3p	964550	964573	scaffold_26	minus	964491	964684	star1	TCAGCTTGTTCTGGATAACCTGCG
MIR211a	5p	1994393	1994414	scaffold_20	plus	1994272	1994615	mature1	TATCTTCGTAGGCCATGAAGGC
MIR211b	5p	63785	63806	scaffold_85	minus	63583	63928	mature1	TATCTTCGTAGGCCATGAAGGC
MIR212a	5p	120457	120477	scaffold_58	plus	120407	120575	mature1	TATGGATGTGTGTGAGATGCC
MIR212a*	3p	120503	120524	scaffold_58	plus	120407	120575	star1	TATGTCGCCACGTTTCTGGATG
MIR212b	5p	52159	52179	scaffold_78	plus	52109	52277	mature1	TATGGATGTGTGTGAGATGCC
MIR212b*	3p	52205	52226	scaffold_78	plus	52109	52277	star1	TATGTCGCCACGTTTCTGGATG
MIR212c	5p	1067343	1067363	scaffold_15	plus	1067293	1067461	mature1	TATGGATGTGTGTGAGATGCC
MIR212c*	3p	1067389	1067410	scaffold_15	plus	1067293	1067461	star1	TATGTCGCCACGTTTCTGGATG
MIR213	3p	1226993	1227015	scaffold_27	plus	1226679	1227017	mature2	TATGGTGCCTTCCTGTGAACTCC
MIR214	5p	193210	193229	scaffold_12	plus	193142	193502	mature1	TATGGTTGAGAGTAGATAGG
MIR214*	3p	193401	193422	scaffold_12	plus	193142	193502	star1	TCCTATCTACTCTCAACCACAA
MIR215	5p	1377999	1378020	scaffold_6	plus	1377942	1378255	mature1	TATTCACCGAACAACACTGACG
MIR216	3p	208213	208234	scaffold_60	plus	207940	208287	mature1	TATTCCGAACTGCTGCAACTGC
MIR217	5p	1464555	1464574	scaffold_23	plus	1464526	1464786	mature1	TATTGTGGGCGGTGTACTGA
MIR217*	3p	1464740	1464760	scaffold_23	plus	1464526	1464786	star1	TCAGTACACCGCCCACAATAG
MIR218a	3p	3336868	3336890	scaffold_4	minus	3336856	3337037	mature1	TATTTACAGCGTCGTCATCATTC
MIR218b	3p	124488	124510	scaffold_86	minus	124478	124649	mature1	TATTTACAGCGTCGTCATCATTC
MIR219	3p	278549	278570	scaffold_63	plus	278359	278625	mature1	TATTTGTGGTTTCGGGAACGGG
MIR220	3p	3069987	3070008	scaffold_13	plus	3069817	3070075	mature1	TCAACTAACCACCTTACCGGCG
MIR220*	5p	3069877	3069898	scaffold_13	plus	3069817	3070075	star1	CCGGTAAGGTGGTTAATTGAAG
MIR221	3p	237456	237475	scaffold_47	plus	237389	237493	mature1	TCAAGACCCTGTCCACCATG
MIR222a	5p	1278084	1278104	scaffold_8	minus	1277838	1278126	mature1	TCAAGGCCCAGACTGAAGACC
MIR222a*	3p	1277904	1277924	scaffold_8	minus	1277838	1278126	star1	TCAGTCTGGGCCTTGAAGCGG
MIR222b	5p	1277909	1277929	scaffold_8	plus	1277854	1278157	mature1	TCAAGGCCCAGACTGAAGACC
MIR222b*	3p	1278088	1278109	scaffold_8	plus	1277854	1278157	star1	TTCAGTCTGGGCCTTGAAGCGG
MIR223	5p	176390	176410	scaffold_40	plus	176345	176593	mature1	TCAAGTGTTGCGGAATGTTGC
MIR224	5p	235621	235641	scaffold_61	minus	235413	235674	mature1	TCAAGTTCTCTGCAGTCATCA
MIR225	3p	1121323	1121344	scaffold_6	minus	1121305	1121548	mature1	TCACAGCCAGCGGAGAGATCGT
MIR225*	5p	1121526	1121547	scaffold_6	minus	1121305	1121548	star1	TGCATTGCGATCTCTCCGCTGG
MIR226a	5p	3025801	3025822	scaffold_1	minus	3025659	3025871	mature1	TCACCTCCGGATCAGCACGTGC
MIR226a*	3p	3025686	3025706	scaffold_1	minus	3025659	3025871	star1	TGCTGTGCCCTCACCTCCGGG
MIR226b	5p	12165	12186	scaffold_3	minus	12023	12235	mature1	TCACCTCCGGATCAGCACGTGC
MIR226b*	3p	12050	12070	scaffold_3	minus	12023	12235	star1	TGCTGTGCCCTCACCTCCGGG
MIR226c	3p	102605	102626	scaffold_81	plus	102299	102640	mature1	TCACCTCCGGATCAGCACGTGC
MIR227a	5p	1074853	1074874	scaffold_23	plus	1074837	1074982	mature1	TCACCTGCCCACTGTGGCCTGG
MIR227a*	3p	1074906	1074925	scaffold_23	plus	1074837	1074982	star1	TGCTGCTGGGCGAGTGCTGC
MIR227b	5p	659427	659448	scaffold_31	minus	659319	659472	mature1	TCACCTGCCCACTGTGGCCTGG
MIR227b*	3p	659376	659396	scaffold_31	minus	659319	659472	star1	TTGCTGCTGGGCGAGTGCTGC
MIR227c	5p	2129209	2129230	scaffold_12	plus	2129193	2129338	mature1	TCACCTGCCCACTGTGGCCTGG
MIR227c*	3p	2129261	2129281	scaffold_12	plus	2129193	2129338	star1	TTGCTGCTGGGCGAGTGCTGC
MIR227d	5p	2794752	2794773	scaffold_1	plus	2794736	2794881	mature1	TCACCTGCCCACTGTGGCCTGG
MIR227d*	3p	2794803	2794823	scaffold_1	plus	2794736	2794881	star1	TTTGCTGCTGGGCGAGTGCTG
MIR228	5p	390694	390715	scaffold_66	minus	390626	390733	mature1	TCACGGCCGCTGCATACGAGGG
MIR228*	3p	390665	390685	scaffold_66	minus	390626	390733	star1	CGTTTGAGGGGCCTGGTTGGC
MIR229	5p	1962932	1962951	scaffold_15	plus	1962924	1963123	mature1	TCAGAGGACACACATGGAGT
MIR230	3p	199883	199903	scaffold_16	minus	199852	200113	mature2	TCAGCTGTGTCGTGTAATTGG
MIR231	5p	183133	183155	scaffold_21	plus	183107	183328	mature1	TCAGGGCTCTAGTCGCAGTATGG
MIR232	3p	985834	985856	scaffold_16	plus	985610	985871	mature2	TCAGGGTGGGAGCTAGCTACTGG
MIR233a	3p	538900	538921	scaffold_21	minus	538897	539004	mature1	TCATCGGTCGCCGCTGGCAGCA
MIR233b	3p	2680874	2680895	scaffold_3	plus	2680791	2680897	star1	TCATCGGTCGCCGCTGGCAGCA
MIR234	5p	815551	815572	scaffold_39	plus	815449	815678	mature1	TCATCGTGCAGGTCGGTGCTGG
MIR234*	5p	815516	815536	scaffold_39	plus	815449	815678	star1	CACCGACAGGCTCCACAGTGG
MIR235	5p	1960103	1960125	scaffold_11	plus	1960035	1960317	mature1	TCATCTTAGGATGGATCGGTATT
MIR236	3p	1988016	1988035	scaffold_14	minus	1987951	1988228	mature1	TCATGTGGTTGACTGCCAGG
MIR237	5p	6708	6729	scaffold_321	minus	6541	6736	mature1	TCCAGACCCAGCTTCCACTCCT
MIR238	5p	3737633	3737653	scaffold_7	plus	3737624	3737967	mature1	TCCAGGACGCGGATGAATTCC
MIR238*	3p	3737945	3737965	scaffold_7	plus	3737624	3737967	star1	TCATCCGCGTCCTGGAGAAGC
MIR239a	3p	409139	409159	scaffold_43	plus	409067	409161	mature1	TCCATGAAGTCGGGCCTTTCA
MIR239a*	5p	409073	409093	scaffold_43	plus	409067	409161	star1	AAGGCCCGACTTCATGGATTC
MIR239b	3p	409070	409090	scaffold_43	minus	409067	409161	mature1	TCCATGAAGTCGGGCCTTTCA
MIR239b*	5p	409136	409156	scaffold_43	minus	409067	409161	star1	AAGGCCCGACTTCATGGATTC
MIR240a	3p	150799	150820	scaffold_74	plus	150694	150850	mature1	TCCATGGCGCTCGTGACGGAGG
MIR240a*	5p	150725	150746	scaffold_74	plus	150694	150850	star1	TCCGTCACGAGCGCCATGGACG
MIR240b	3p	150723	150744	scaffold_74	minus	150629	150848	mature1	TCCATGGCGCTCGTGACGGAGG
MIR240b*	5p	150797	150818	scaffold_74	minus	150629	150848	star1	TCCGTCACGAGCGCCATGGACG
MIR241	3p	3920470	3920490	scaffold_1	minus	3920429	3920622	mature2	TCCCGAATTATTGTAGCACGA
MIR242	3p	2678363	2678384	scaffold_4	plus	2678192	2678429	mature1	TCCCGACGATAGAAAAGGCGCG

MIR242*	5p	2678231	2678252	scaffold_4	plus	2678192	2678429	star1	TTGAACACGCGCCTTTTCTATC
MIR243a	3p	7263717	7263738	scaffold_1	minus	7263643	7263963	mature2	TCCCGCCTTCTGCAAAACTGTC
MIR243b	3p	7263878	7263899	scaffold_1	plus	7263655	7263960	mature2	TCCCGCCTTCTGCAAAACTGTC
MIR244	5p	518643	518664	scaffold_25	minus	518459	518711	mature2	TCCCGCGCTGTTGTATCCTGGA
MIR244*	3p	518505	518526	scaffold_25	minus	518459	518711	star2	TCCAGGATACAACAACGGGATC
MIR245	3p	2074958	2074979	scaffold_26	minus	2074952	2075082	mature1	TCCCTGCTATCGCCACTGCCGC
MIR246a	3p	194342	194362	scaffold_55	minus	194337	194402	mature1	TCCGAAAGGCACTCGACTGTA
MIR246a*	5p	194381	194401	scaffold_55	minus	194337	194402	star1	TACAGTCGAGTGCCTTTCGGA
MIR246b	3p	194381	194401	scaffold_55	plus	194340	194407	mature1	TCCGAAAGGCACTCGACTGTA
MIR246b*	5p	194342	194362	scaffold_55	plus	194340	194407	star1	TACAGTCGAGTGCCTTTCGGA
MIR247	5p	1450304	1450324	scaffold_20	plus	1450285	1450626	mature3	TCCGCAGGTAGATAATTTTGT
MIR247*	3p	1450589	1450609	scaffold_20	plus	1450285	1450626	star3	CAAAATTATCTACCTGCGGAA
MIR248	3p	7263736	7263758	scaffold_1	minus	7263643	7263963	mature4	TCCGTGATGGCTGAGAGACATCC
MIR249	3p	1290319	1290342	scaffold_27	plus	1290035	1290357	mature1	TCCTTGTCATCTTTCTCGTCGCCC
MIR249*	5p	1290054	1290076	scaffold_27	plus	1290035	1290357	star1	TGACGAGAAAGATGACAAGGAAG
MIR250	3p	1326365	1326385	scaffold_2	minus	1326323	1326644	mature3	TCGCGCGTCCACTGGTGCAAC
MIR251a	5p	1250678	1250698	scaffold_14	plus	1250672	1250760	mature1	TCGCGTAGAAGGCCGGGCTGG
MIR251b	5p	165437	165457	scaffold_76	plus	165395	165519	mature1	TCGCGTAGAAGGCCGGGCTGG
MIR252	5p	76316	76337	scaffold_21	plus	76131	76523	mature1	TCGCTACGGGGTCGCTACTGGG
MIR253	3p	12210	12231	scaffold_18	minus	12152	12423	mature1	TCGGAGAGCCAGTCGAGAGCGC
MIR253*	5p	12340	12361	scaffold_18	minus	12152	12423	star1	TGGCGCTCTCGACTGGCTCTCC
MIR254	5p	4800077	4800096	scaffold_2	plus	4800060	4800185	mature1	TCGGCGATGAGGGATGTGGC
MIR255a	3p	1396361	1396382	scaffold_18	minus	1396352	1396483	mature1	TCGGCTGAAGCTCCAGTATTCA
MIR255a*	5p	1396453	1396475	scaffold_18	minus	1396352	1396483	star1	TGAGACCCAGAAGGCTGATCGGG
MIR255b	3p	1486489	1486510	scaffold_18	minus	1486480	1486611	mature1	TCGGCTGAAGCTCCAGTATTCA
MIR255b*	5p	1486581	1486603	scaffold_18	minus	1486480	1486611	star1	TGAGACCCAGAAGGCTGATCGGG
MIR256	3p	653322	653342	scaffold_41	plus	653085	653474	mature1	TCGGGATTAAGGTGGCGCTGC
MIR257a	5p	137729	137749	scaffold_85	minus	137576	137786	mature1	TCGGTCGTATATCGCTGCTCC
MIR257a*	3p	137611	137631	scaffold_85	minus	137576	137786	star1	AGCAGCGGTACACGACCCGGT
MIR257b	5p	648	668	scaffold_48	plus	626	818	mature1	TCGGTCGTATATCGCTGCTCC
MIR257b*	3p	779	799	scaffold_48	plus	626	818	star1	AGCAGCGGTACACGACCCGGT
MIR258	3p	7263676	7263695	scaffold_1	minus	7263643	7263963	mature3	TCGTAGTTCTCGCTTGTGAG
MIR259	3p	1547467	1547490	scaffold_18	plus	1547226	1547549	mature1	TCGTGACGCCGGGGAAGGGGAGGG
MIR259*	5p	1547283	1547306	scaffold_18	plus	1547226	1547549	star1	CTCCCCTTCCCCGGCGTCACGACC
MIR260	3p	944552	944573	scaffold_19	minus	944520	944655	mature2	TCTAACGAGGCTGCGACGTACG
MIR261	5p	224461	224482	scaffold_4	minus	224234	224523	mature2	TCTCGCCGGGGAATGAAGGAAA
MIR261*	3p	224274	224295	scaffold_4	minus	224234	224523	star2	TCTTTTGTTCCCCGGCGAGAGG
MIR262	3p	578041	578062	scaffold_51	plus	577972	578070	mature1	TCTCTGCACTGGGCTGTGAGCT
MIR263	5p	7202610	7202630	scaffold_1	minus	7202286	7202667	mature2	TCTGATGCCGGAGCTTCCTGA
MIR264a	3p	123214	123234	scaffold_97	plus	123143	123241	mature1	TCTGCACTGGGCTGTGAGCTT
MIR264b	3p	244844	244864	scaffold_74	plus	244785	244871	mature1	TCTGCACTGGGCTGTGAGCTT
MIR264c	3p	1552302	1552322	scaffold_31	plus	1552231	1552329	mature1	TCTGCACTGGGCTGTGAGCTT
MIR264d	3p	140867	140887	scaffold_93	plus	140793	140894	mature1	TCTGCACTGGGCTGTGAGCTT
MIR264e	3p	27617	27637	scaffold_64	minus	27609	27707	mature1	TCTGCACTGGGCTGTGAGCTT
MIR264f	3p	39286	39306	scaffold_32	minus	39278	39376	mature1	TCTGCACTGGGCTGTGAGCTT
MIR264g	3p	3611	3631	scaffold_53	minus	3603	3701	mature1	TCTGCACTGGGCTGTGAGCTT
MIR264h	3p	123476	123496	scaffold_15	minus	123468	123554	mature1	TCTGCACTGGGCTGTGAGCTT
MIR264i	3p	29417	29437	scaffold_8	minus	29409	29510	mature1	TCTGCACTGGGCTGTGAGCTT
MIR264j	3p	992	1012	scaffold_82	minus	984	1082	mature1	TCTGCACTGGGCTGTGAGCTT
MIR264k	3p	22653	22673	scaffold_71	minus	22645	22743	mature1	TCTGCACTGGGCTGTGAGCTT
MIR264l	3p	83340	83360	scaffold_50	minus	83332	83430	mature1	TCTGCACTGGGCTGTGAGCTT
MIR265	5p	132536	132557	scaffold_25	minus	132327	132630	mature2	TCTGGATCGAGGATTTCCGAGC
MIR265*	3p	132398	132419	scaffold_25	minus	132327	132630	star2	TCGGAAATCCTCGATCCAGATG
MIR266a	3p	869238	869258	scaffold_41	plus	869110	869261	mature1	TCTTGGGCGGCCGTACCTTGC
MIR266b	3p	231411	231431	scaffold_75	plus	231255	231433	mature1	TCTTGGGCGGCCGTACCTTGC
MIR267a	3p	1864028	1864048	scaffold_24	minus	1864000	1864265	mature1	TCTTGTCCCGAACCTTGTCCT
MIR267b	3p	1864219	1864239	scaffold_24	plus	1864016	1864252	mature1	TCTTGTCCCGAACCTTGTCCT
MIR268	3p	4647552	4647572	scaffold_2	plus	4647404	4647580	mature1	TCTTGTGGACGGTGTAGGACG
MIR269	3p	926999	927019	scaffold_24	minus	926962	927139	mature1	TCTTTCAACATGCAGAATTGC
MIR270	3p	366842	366862	scaffold_67	plus	366632	366889	mature2	TGAAAATAAAGAGCAAGGCGC
MIR271a	3p	1256	1276	scaffold_17	minus	1225	1483	mature1	TGAAAGTGAGGATCTATTGAA
MIR271a*	5p	1431	1451	scaffold_17	minus	1225	1483	star1	CAATAGATCCTCACTTTCAGC
MIR271b	3p	1433	1453	scaffold_17	plus	1225	1483	mature2	TGAAAGTGAGGATCTATTGAA
MIR272	3p	380056	380076	scaffold_8	plus	379878	380156	mature1	TGAAATAGAATGGACTCCGTT
MIR273	5p	3371151	3371172	scaffold_5	plus	3371149	3371227	mature1	TGAACGTGTGCAAGTCCTGAGC
MIR274	5p	102720	102741	scaffold_91	plus	102638	102990	mature1	TGAACTCATCCTTGTCGCTGCC
MIR274*	3p	102919	102940	scaffold_91	plus	102638	102990	star1	TTGAAGGGCAGCAACAAGGATG
MIR275	3p	12985384	12985405	scaffold_1	minus	12985330	12985532	mature1	TGAATGCGGAACAAGCAGCAGG
MIR276	3p	2708107	2708129	scaffold_3	minus	2707962	2708314	mature1	TGACAAACGCGACTTTGTTCGGG
MIR277	3p	2666338	2666360	scaffold_14	minus	2666320	2666650	mature1	TGACATCAACCTCTCTGCAGACG
MIR278	5p	231215	231236	scaffold_61	plus	231205	231290	mature1	TGACCCGGATGGGCTGCAGATC
MIR279a	3p	430322	430343	scaffold_40	minus	430258	430543	mature2	TGACCTTCTTGCATCCCGCTGC
MIR279b	3p	430322	430343	scaffold_40	minus	430264	430542	mature2	TGACCTTCTTGCATCCCGCTGC
MIR280	3p	3167846	3167870	scaffold_4	minus	3167787	3168030	mature1	TGACGACAGTGGCAATGGTGATGGC
MIR280*	5p	3167951	3167972	scaffold_4	minus	3167787	3168030	star1	CGCCATTGCCACTGTCGCCATC
MIR281	3p	1232	1255	scaffold_17	minus	1225	1483	mature2	TGACGGTGATATGGATGATGAGGC
MIR281*	5p	1410	1430	scaffold_17	minus	1225	1483	star2	TTCAGACCACTCTGACTGATC

MIR282	3p	733193	733212	scaffold_37	plus	733003	733259	mature1	TGACTGCGGAGAGCTTCGGA
MIR283	3p	124167	124189	scaffold_86	plus	124095	124227	mature1	TGAGAGGCCAGATCGCAGCATGA
MIR283*	5p	124140	124160	scaffold_86	plus	124095	124227	star1	CTTGACATCGGTCTCCTCACT
MIR284	3p	270825	270846	scaffold_51	plus	270573	270864	mature2	TGAGATTCTCGGCTGTAATTGA
MIR284*	5p	270598	270619	scaffold_51	plus	270573	270864	star2?	TACAGCCAAGAATTTCATGGAC
MIR285	5p	696225	696246	scaffold_3	minus	696026	696274	mature1	TGAGCGATCCCTGTAGTAACCC
MIR285*	3p	696049	696070	scaffold_3	minus	696026	696274	star1	TACTACAGGGATCGCTTATGTT
MIR286	3p	3683373	3683393	scaffold_7	plus	3683241	3683409	mature1	TGAGCGCATGGGGCTAGTTGG
MIR286*	5p	3683262	3683284	scaffold_7	plus	3683241	3683409	star1	CAGCCCCACGCGCTCATCCCGGC
MIR287	5p	4501983	4502003	scaffold_1	plus	4501912	4502231	mature1	TGAGGAGTGAGTCAGATATGG
MIR288a	3p	538384	538405	scaffold_26	minus	538343	538638	mature1	TGAGGCTGTGTCCCGGGGCGGT
MIR288a*	5p	538610	538631	scaffold_26	minus	538343	538638	star1	CGCCCCGGGACACAGCCTCAGG
MIR288b	3p	538612	538633	scaffold_26	plus	538377	538638	mature1	TGAGGCTGTGTCCCGGGGCGGT
MIR288b*	5p	538386	538407	scaffold_26	plus	538377	538638	star1	CGCCCCGGGACACAGCCTCAGG
MIR289	5p	682492	682512	scaffold_13	plus	682490	682588	mature1	TGAGGTATAGCTGTGGCTGAC
MIR290a	5p	3115057	3115078	scaffold_1	plus	3114929	3115354	mature1	TGATGAACTTGCTTGCTTTACT
MIR290a*	3p	3115215	3115236	scaffold_1	plus	3114929	3115354	star1	TAAAGCAAGCAAGTTCATCAGG
MIR290b	5p	3115213	3115234	scaffold_1	minus	3114930	3115354	mature1	TGATGAACTTGCTTGCTTTACT
MIR290b*	3p	3115055	3115076	scaffold_1	minus	3114930	3115354	star1	TAAAGCAAGCAAGTTCATCAGG
MIR291	3p	1403	1424	scaffold_17	plus	1225	1483	mature1	TGATGATGATCAGTCAGAGTGG
MIR292	3p	142492	142513	scaffold_70	plus	142284	142548	mature1	TGCAAACACACCCTGCCCGCCA
MIR292*	5p	142318	142340	scaffold_70	plus	142284	142548	star1	TATGGCGGGCAGGGTGTGTTTGC
MIR293	3p	199139	199160	scaffold_62	minus	199106	199353	mature1	TGCAACTCGCTTCTTGGGAAGA
MIR293*	5p	199297	199318	scaffold_62	minus	199106	199353	star1	TGCCTAAGAAACCAGATGCAGA
MIR294	5p	833432	833456	scaffold_38	minus	833302	833462	mature1	TGCAAGACTGGGCGCTTGAAACGGC
MIR295	5p	122460	122480	scaffold_49	plus	122432	122585	mature1	TGCACGACGATGCCCAGGACG
MIR296	5p	775647	775668	scaffold_12	plus	775621	775776	mature1	TGCAGCACGGATGACGAAGACA
MIR296*	3p	775729	775750	scaffold_12	plus	775621	775776	star1	TCTTCATCATCCCTGCTGCAGT
MIR297	5p	1039895	1039916	scaffold_4	plus	1039891	1040201	mature1	TGCAGCCTGATCAAGACGAAGG
MIR297*	3p	1040181	1040200	scaffold_4	plus	1039891	1040201	star1	TTCGTCTTGATCAGGCTGCA
MIR298	3p	2140197	2140220	scaffold_18	plus	2140009	2140261	mature1	TGCAGGAGTGTGCTGGTGACCGGC
MIR298*	5p	2140053	2140076	scaffold_18	plus	2140009	2140261	star1	CGGTCACCAGCATGCTCCTGCAGA
MIR299	3p	3062223	3062244	scaffold_13	minus	3062192	3062459	mature1	TGCAGGCCTTGCCCGATGCGGC
MIR299*	5p	3062403	3062423	scaffold_13	minus	3062192	3062459	star1	TCGGGCAAGGCCTGCATGTGA
MIR300	5p	748428	748450	scaffold_44	plus	748399	748555	mature1	TGCAGGTCGGTGCTGGTGACACC
MIR301	5p	247614	247635	scaffold_68	plus	247600	247696	mature1	TGCAGTGTCGGTGCCAGATGGG
MIR302	3p	21293	21314	scaffold_48	plus	21224	21359	mature1	TGCAGTGTGCATCGACCATGGC
MIR303a	5p	2354029	2354049	scaffold_16	plus	2354022	2354116	mature1	TGCATCAGGAAGTGGGACTTC
MIR303a*	3p	2354090	2354111	scaffold_16	plus	2354022	2354116	star1	TTGTACAAGTTCTTCGATCCAG
MIR303b	5p	2072278	2072298	scaffold_17	plus	2072273	2072362	mature1	TGCATCAGGAAGTGGGACTTC
MIR303b*	3p	2072339	2072360	scaffold_17	plus	2072273	2072362	star1	TTGTACAAGTTCTTCGATCCAG
MIR303c	5p	8586	8606	scaffold_3	plus	8581	8670	mature1	TGCATCAGGAAGTGGGACTTC
MIR303c*	3p	8647	8668	scaffold_3	plus	8581	8670	star1	TTGTACAAGTTCTTCGATCCAG
MIR304a	3p	7263695	7263716	scaffold_1	minus	7263643	7263963	mature1	TGCATCCGCCAGAACCAGAACT
MIR304a*	5p	7263898	7263919	scaffold_1	minus	7263643	7263963	star1	TTCTGGTTCTGGCGGATGCAGA
MIR304b	3p	7263900	7263921	scaffold_1	plus	7263655	7263960	mature1	TGCATCCGCCAGAACCAGAACT
MIR304b*	5p	7263700	7263720	scaffold_1	plus	7263655	7263960	star1	TGGTTCTGGCGGATGCAGACA
MIR305a	5p	446742	446761	scaffold_19	minus	446579	446789	mature1	TGCATGGGATGGACAGCCGC
MIR305b	5p	6137	6156	scaffold_54	plus	6109	6330	mature2	TGCATGGGATGGACAGCCGC
MIR306	5p	1366404	1366425	scaffold_23	plus	1366401	1366698	mature1	TGCCAAGAAGTGCTGTTTGCTC
MIR306*	3p	1366673	1366695	scaffold_23	plus	1366401	1366698	star1	TAGGGCAAACAGCACTTCCTGGC
MIR307	3p	2139355	2139374	scaffold_21	minus	2139251	2139577	mature1	TGCCGCCCTCGTCCTTCTGG
MIR308	5p	1000	1021	scaffold_1028	plus	988	1060	mature1	TGCCGGATAATCTCTACTACGG
MIR308*	3p	1031	1052	scaffold_1028	plus	988	1060	star1	TACTAGGATAATGTTGGTGGGG
MIR309	3p	5039488	5039508	scaffold_2	minus	5039337	5039669	mature1	TGCGAGACCACTGACATGACA
MIR309*	5p	5039610	5039630	scaffold_2	minus	5039337	5039669	star1	TGTCATGTCAACGGTCTCGCA
MIR310	3p	85768	85789	scaffold_35	plus	85694	85795	mature1	TGCGATGCGTTGATTCTCTCAG
MIR310*	5p	85703	85724	scaffold_35	plus	85694	85795	star1	GAGAGAATCGACGCACCGCAGT
MIR311	3p	1130276	1130300	scaffold_23	plus	1130116	1130325	mature1	TGCGGACAGGGCAGATGTGGAGCGG
MIR312	5p	1728335	1728356	scaffold_27	plus	1728277	1728550	mature1	TGCGGACGAGGTCGAGAAGAGC
MIR312*	3p	1728471	1728492	scaffold_27	plus	1728277	1728550	star1	TGCTCTTCTCGACCTCGTCCGC
MIR313	5p	286651	286672	scaffold_59	plus	286634	286971	mature1	TGCGGATAGCCTTTGCGATGCA
MIR313*	3p	286932	286955	scaffold_59	plus	286634	286971	star1	CGTGCATCGCAAAGGCTATCCGCA
MIR314a	5p	111325	111347	scaffold_81	minus	111228	111367	mature1	TGCGGGTGGCTGCTGTCCAGTCC
MIR314b	5p	64582	64604	scaffold_85	minus	64489	64621	mature1	TGCGGGTGGCTGCTGTCCAGTCC
MIR314b*	3p	64504	64524	scaffold_85	minus	64489	64621	star1	TGCACAGCAGCCATTCGCAGG
MIR314c	5p	1993443	1993465	scaffold_20	plus	1993425	1993557	mature1	TGCGGGTGGCTGCTGTCCAGTCC
MIR314c*	3p	1993523	1993543	scaffold_20	plus	1993425	1993557	star1	TGCACAGCAGCCATTCGCAGG
MIR315	3p	173309	173331	scaffold_17	plus	173107	173372	mature1	TGCGTATGCACAGAGAATCGGAT
MIR315*	5p	173150	173169	scaffold_17	plus	173107	173372	star1	TGCGATTCTCTGTGCATACG
MIR316	3p	904914	904935	scaffold_2	minus	904897	905138	mature1	TGCTGACTGTTATTCCGGAAGG
MIR316*	5p	905101	905122	scaffold_2	minus	904897	905138	star1	TTCCGGAATAACAATTAGCGTT
MIR317	3p	3046386	3046406	scaffold_8	plus	3046179	3046483	mature1	TGCTGCCCAACGTATGCCGGC
MIR318	5p	180868	180888	scaffold_38	plus	180848	180938	mature1	TGCTGCCGGCATGCAAGCTGT
MIR319	3p	1690486	1690508	scaffold_12	plus	1690217	1690534	mature1	TGCTGCGGTGTGTAGGGTGTGGA
MIR319*	5p	1690249	1690271	scaffold_12	plus	1690217	1690534	star1	CACACCATACATGCTGCAGCAGA
MIR320a	5p	79376	79396	scaffold_21	plus	79364	79510	mature1	TGCTGGCACAGCAAGCTGACG

MIR320a*	3p	79487	79507	scaffold_21	plus	79364	79510	star1	TTGCTGTGCCAGCAATGGACT
MIR320b	5p	79480	79500	scaffold_21	minus	79364	79511	mature1	TGCTGGCACAGCAAGCTGACG
MIR320b*	3p	79369	79389	scaffold_21	minus	79364	79511	star1	TTGCTGTGCCAGCAATGGACT
MIR321a	3p	4738836	4738855	scaffold_2	plus	4738707	4738874	mature1	TGCTTGATGATGAACGGGGG
MIR321b	3p	4738727	4738746	scaffold_2	minus	4738707	4738874	mature1	TGCTTGATGATGAACGGGGG
MIR322a	3p	12369	12390	scaffold_7	minus	12352	12518	mature1	TGGAAAACTTAGGCTGTGAAAC
MIR322a*	5p	12477	12499	scaffold_7	minus	12352	12518	star1	TTCCGAGCTTAAGTTTTCCCAAA
MIR322b	3p	10548	10569	scaffold_47	minus	10531	10697	mature1	TGGAAAACTTAGGCTGTGAAAC
MIR322b*	5p	10656	10678	scaffold_47	minus	10531	10697	star1	TTCCGAGCTTAAGTTTTCCCAAA
MIR322c	3p	13539	13560	scaffold_192	minus	13522	13688	mature1	TGGAAAACTTAGGCTGTGAAAC
MIR322c*	5p	13647	13669	scaffold_192	minus	13522	13688	star1	TTCCGAGCTTAAGTTTTCCCAAA
MIR323a	3p	1994505	1994525	scaffold_14	plus	1994452	1994560	mature1	TGGAAGACCGGGTGTAGGCGG
MIR323b	5p	645056	645076	scaffold_22	plus	645003	645139	mature1	TGGAAGACCGGGTGTAGGCGG
MIR323c	3p	46016	46036	scaffold_78	plus	45956	46080	mature1	TGGAAGACCGGGTGTAGGCGG
MIR324	5p	115551	115570	scaffold_85	plus	115514	115808	mature1	TGGAAGATCCGGAGGTGAGG
MIR324*	3p	115676	115695	scaffold_85	plus	115514	115808	star1	TCCTGCACCCTCACCTCCGG
MIR325a	3p	10097908	10097928	scaffold_1	plus	10097709	10097959	mature1	TGGAAGCTGGGTCTGGAGGGC
MIR325a*	5p	10097740	10097759	scaffold_1	plus	10097709	10097959	star1	TGCCCTCCAGACCCAGCTTC
MIR325b	3p	2479	2502	scaffold_321	plus	2280	2530	mature1	TGGAAGCTGGGTCTGGAGGGCATG
MIR325b*	5p	2311	2330	scaffold_321	plus	2280	2530	star1	TGCCCTCCAGACCCAGCTTC
MIR325c	3p	6713	6736	scaffold_321	plus	6541	6736	mature1	TGGAAGCTGGGTCTGGAGGGCATG
MIR325c*	5p	6544	6566	scaffold_321	plus	6541	6736	star1	TGCCCTCCAGACCCAGCTTCCAC
MIR326	3p	673289	673310	scaffold_38	minus	673287	673351	mature1	TGGAAGGTGGGGTGGATGCGCA
MIR327	3p	1572547	1572567	scaffold_13	minus	1572540	1572638	mature1	TGGACAAGACAGGAGCTGCGC
MIR328a	5p	270615	270635	scaffold_51	plus	270573	270864	mature1	TGGACACGATATCGTCTGGCT
MIR328a*	3p	270803	270826	scaffold_51	plus	270573	270864	star1	TAGCCAGACGATATCGTGTCCATG
MIR328b	5p	270804	270824	scaffold_51	minus	270611	270827	mature1	TGGACACGATATCGTCTGGCT
MIR328b*	3p	270614	270635	scaffold_51	minus	270611	270827	star1	AGCCAGACGATATCGTGTCCAT
MIR329	3p	1972	1993	scaffold_94	plus	1861	2089	mature1	TGGACAGGGTCTTGAGACGGCT
MIR329*	3p	2013	2038	scaffold_94	plus	1861	2089	star1	TATCAAGACGCTGTTCATCATGTGAT
MIR330a	5p	1428388	1428409	scaffold_26	plus	1428362	1428623	mature1	TGGACTAGTTACTCCTGAGACG
MIR330a*	3p	1428581	1428603	scaffold_26	plus	1428362	1428623	star1	TCAGGAGTAACTAGTCCAGTTGC
MIR330b	5p	277086	277107	scaffold_58	minus	276851	277149	mature1	TGGACTAGTTACTCCTGAGACG
MIR330b*	3p	276900	276920	scaffold_58	minus	276851	277149	star1	TAACCCGTCTCAGGAGTAACT
MIR330c	5p	276894	276915	scaffold_58	plus	276856	277142	mature1	TGGACTAGTTACTCCTGAGACG
MIR330c*	3p	277090	277112	scaffold_58	plus	276856	277142	star1	TCAGGAGTAACTAGTCCAGTTGC
MIR331	5p	6263	6282	scaffold_76	minus	6054	6320	mature1	TGGACTGAGGGTCAGCAGGC
MIR331*	3p	6088	6107	scaffold_76	minus	6054	6320	star1	TGCTGACCCTCAGTCCATGC
MIR332a	3p	6270	6290	scaffold_101	plus	6142	6379	mature1	TGGAGACTGCTTCATTTTGGA
MIR332b	3p	371807	371827	scaffold_61	minus	371713	371958	mature1	TGGAGACTGCTTCATTTTGGA
MIR332c	5p	236883	236903	scaffold_47	minus	236745	236907	mature1	TGGAGACTGCTTCATTTTGGA
MIR333	3p	224296	224315	scaffold_4	minus	224234	224523	mature1	TGGAGATTTCTGCACGTGTT
MIR333*	5p	224441	224460	scaffold_4	minus	224234	224523	star1	CGCGGGCCGAAATCTCCAGC
MIR334	5p	92708	92728	scaffold_47	minus	92544	92755	mature1	TGGAGGTGCCTTTTCTTAGGG
MIR335	5p	641654	641675	scaffold_28	minus	641411	641737	mature1	TGGAGTGAAGGTGGGCTGTTGG
MIR336	3p	4788180	4788199	scaffold_2	plus	4788034	4788298	mature1	TGGAGTTGGATTGGCAGGGG
MIR337	3p	1115349	1115371	scaffold_26	minus	1115340	1115506	mature1	TGGATCGAGATTCGCACGCTGGA
MIR337*	3p	1115382	1115402	scaffold_26	minus	1115340	1115506	star1	TGCTGAGATGTGGAGTTGAGG
MIR338	3p	53829	53850	scaffold_12	plus	53758	53853	mature1	TGGATGCTGGTGGTGGGTGCGG
MIR339	3p	366700	366723	scaffold_67	plus	366632	366889	mature1	TGGATGCTGTATTGTCAGAACAGG
MIR339*	3p	366802	366823	scaffold_67	plus	366632	366889	star1	TGTCCTGACAATACAGCATCCA
MIR340	3p	359707	359728	scaffold_25	minus	359690	359818	mature1	TGGCAAGGAGAGACTGATGCGC
MIR340*	5p	359778	359799	scaffold_25	minus	359690	359818	star1	CATCAGTCTATCCTTGCCATCA
MIR341	3p	1275307	1275327	scaffold_22	minus	1275258	1275501	mature2	TGGCAATGGCGGCAGTGGGGA
MIR342	3p	1733154	1733174	scaffold_28	plus	1733083	1733185	mature1	TGGCAATTGTGATGGCAGGGA
MIR342*	5p	1733104	1733125	scaffold_28	plus	1733083	1733185	star1	TGCCATCACCATTGCCGCCGTC
MIR343	5p	21762	21783	scaffold_39	plus	21702	22029	mature2	TGGCAGAGGAAGATAGGTGAGG
MIR344a	5p	2075309	2075329	scaffold_26	minus	2075105	2075377	mature1	TGGCGACGGCGGCAATGGCGA
MIR344b	5p	115256	115276	scaffold_21	minus	115021	115283	mature1	TGGCGACGGCGGCAATGGCGA
MIR344b*	3p	115034	115057	scaffold_21	minus	115021	115283	star1	TCCCTGCCATCGCCACTGCCACCG
MIR344c	5p	1733373	1733393	scaffold_28	minus	1733051	1733441	mature1	TGGCGACGGCGGCAATGGCGA
MIR344c*	5p	1733310	1733330	scaffold_28	minus	1733051	1733441	star1	TCGCCATTGCCGCCGTCGCCA
MIR345a	3p	1347773	1347793	scaffold_27	minus	1347671	1347904	mature1	TGGCGACGGTGGCAATAGCGA
MIR345b	3p	1275286	1275306	scaffold_22	minus	1275258	1275501	mature1	TGGCGACGGTGGCAATAGCGA
MIR345b*	5p	1275447	1275467	scaffold_22	minus	1275258	1275501	star1	ACAATTGCCACCGTCGCCATC
MIR345c	3p	3167983	3168003	scaffold_4	plus	3167787	3168030	mature1	TGGCGACGGTGGCAATAGCGA
MIR345c*	5p	3167822	3167842	scaffold_4	plus	3167787	3168030	star1	ACAATTGCCACCGTCGCCATC
MIR346a	3p	70000	70020	scaffold_84	minus	69965	70089	mature1	TGGCGACGGTGGCAATTGTGA
MIR346a*	5p	70036	70056	scaffold_84	minus	69965	70089	star1	TCACCATTGCCGCCGTCGCCA
MIR346b	3p	3167820	3167840	scaffold_4	minus	3167787	3168030	mature2	TGGCGACGGTGGCAATTGTGA
MIR347a	5p	985653	985674	scaffold_16	plus	985610	985871	mature1	TGGCGGATCAAGACAGTGCAGG
MIR347a*	3p	985803	985824	scaffold_16	plus	985610	985871	star1	TGCACTGTCTTGATCCGCCAGG
MIR347b	5p	985801	985822	scaffold_16	minus	985594	985886	mature1	TGGCGGATCAAGACAGTGCAGG
MIR348	3p	1515979	1516000	scaffold_18	plus	1515868	1516008	mature1	TGGCGGATGTACCTAAAAGGCA
MIR348*	3p	1515932	1515953	scaffold_18	plus	1515868	1516008	star1	TGCCTTTTAAGTGTCTTTAGGG
MIR349a	5p	8499	8519	scaffold_107	minus	8447	8537	mature1	TGGCGGCCGTCCTCTGCGGGC
MIR349b	5p	11423	11443	scaffold_48	minus	11371	11461	mature1	TGGCGGCCGTCCTCTGCGGGC

MIR350	5p	1994332	1994355	scaffold_20	plus	1994272	1994615	mature2	TGGCGGCTGGACAGGATTGTCCGC
MIR351	5p	110437	110460	scaffold_81	minus	110176	110519	mature1	TGGCGGCTGGACAGGATTGTCTGC
MIR351*	3p	110239	110259	scaffold_81	minus	110176	110519	star1	TGCTGCAGCGGACGATCCTGC
MIR352a	5p	11151	11171	scaffold_147	plus	11126	11382	mature1	TGGCGGCTGGGCAAGATTGTC
MIR352a*	3p	11352	11372	scaffold_147	plus	11126	11382	star1	TGCTGCAGCGGACGATCCTGC
MIR352b	5p	2004069	2004089	scaffold_20	plus	2004067	2004300	mature1	TGGCGGCTGGGCAAGATTGTC
MIR352b*	3p	2004270	2004290	scaffold_20	plus	2004067	2004300	star1	TGCTGCAGCGGACGATCCTGC
MIR352c	5p	2050158	2050178	scaffold_20	plus	2050155	2050389	mature1	TGGCGGCTGGGCAAGATTGTC
MIR352c*	3p	2050359	2050379	scaffold_20	plus	2050155	2050389	star1	TGCTGCAGCGGACGATCCTGC
MIR352d	5p	1605196	1605216	scaffold_25	plus	1605194	1605427	mature1	TGGCGGCTGGGCAAGATTGTC
MIR352d*	3p	1605397	1605417	scaffold_25	plus	1605194	1605427	star1	TGCTGCAGCGGACGATCCTGC
MIR352e	5p	1804300	1804320	scaffold_28	minus	1804088	1804321	mature1	TGGCGGCTGGGCAAGATTGTC
MIR352e*	3p	1804099	1804119	scaffold_28	minus	1804088	1804321	star1	TGCTGCAGCGGACGATCCTGC
MIR352f	5p	81233	81253	scaffold_56	plus	81230	81464	mature1	TGGCGGCTGGGCAAGATTGTC
MIR352f*	3p	81434	81454	scaffold_56	plus	81230	81464	star1	TGCTGCAGCGGACGATCCTGC
MIR352g	5p	49679	49699	scaffold_68	minus	49467	49700	mature1	TGGCGGCTGGGCAAGATTGTC
MIR352g*	3p	49478	49498	scaffold_68	minus	49467	49700	star1	TGCTGCAGCGGACGATCCTGC
MIR353	3p	132419	132439	scaffold_25	minus	132327	132630	mature1	TGGGACCGCTGATACGAAGCT
MIR353*	5p	132516	132535	scaffold_25	minus	132327	132630	star1	TTCGTATCAGCGGTCCCAGG
MIR354	3p	3920511	3920533	scaffold_1	minus	3920429	3920622	mature1	TGGGATACCTTAGAATTTGTACG
MIR355a	5p	4114708	4114728	scaffold_4	plus	4114604	4114936	mature1	TGGGATGAGGGCTGGGACTGG
MIR355a*	3p	4114812	4114832	scaffold_4	plus	4114604	4114936	star1	TCCAGTCCCAGCCCTCATCCC
MIR355b	5p	4114813	4114833	scaffold_4	minus	4114609	4114931	mature1	TGGGATGAGGGCTGGGACTGG
MIR355b*	3p	4114709	4114729	scaffold_4	minus	4114609	4114931	star1	TCCAGTCCCAGCCCTCATCCC
MIR356	3p	272011	272031	scaffold_58	minus	272008	272096	mature1	TGGGATGTCACGATGAGGACG
MIR357a	3p	486018	486039	scaffold_53	plus	485814	486048	mature2	TGGGCCCCAAACGGTCGGTAAC
MIR357b	3p	340370	340391	scaffold_62	plus	340214	340412	mature1	TGGGCCCCAAACGGTCGGTAAC
MIR357c	3p	2513214	2513235	scaffold_11	plus	2513058	2513256	mature1	TGGGCCCCAAACGGTCGGTAAC
MIR357d	3p	28978	28999	scaffold_95	minus	28956	29154	mature1	TGGGCCCCAAACGGTCGGTAAC
MIR357d*	5p	29109	29131	scaffold_95	minus	28956	29154	star1	TACCGACCGTTTGGGGCTCATTA
MIR357e	3p	255519	255540	scaffold_68	minus	255497	255695	mature1	TGGGCCCCAAACGGTCGGTAAC
MIR357e*	5p	255651	255672	scaffold_68	minus	255497	255695	star1	TACCGACCGTTTGGGGCTCATT
MIR357f	3p	11926	11947	scaffold_90	minus	11904	12102	mature1	TGGGCCCCAAACGGTCGGTAAC
MIR357g	3p	21958	21979	scaffold_95	plus	21818	22000	mature1	TGGGCCCCAAACGGTCGGTAAC
MIR357g*	5p	21843	21863	scaffold_95	plus	21818	22000	star1	ACCGACCGCTTGGGGCCCATT
MIR357h	3p	3120027	3120048	scaffold_1	minus	3120005	3120203	mature1	TGGGCCCCAAACGGTCGGTAAC
MIR357h*	5p	3120159	3120180	scaffold_1	minus	3120005	3120203	star1	TACCGACCGTTTGGGGCTCATT
MIR357i	3p	1036213	1036234	scaffold_11	plus	1036057	1036255	mature1	TGGGCCCCAAACGGTCGGTAAC
MIR357i*	5p	1036081	1036102	scaffold_11	plus	1036057	1036255	star1	TACCGACTGTTTGGGGCCCATT
MIR357j	3p	485957	485978	scaffold_53	plus	485814	486048	mature1	TGGGCCCCAAACGGTCGGTAAC
MIR357k	3p	256294	256315	scaffold_74	plus	256121	256353	mature1	TGGGCCCCAAACGGTCGGTAAC
MIR357l	3p	117277	117298	scaffold_84	plus	117102	117319	mature1	TGGGCCCCAAACGGTCGGTAAC
MIR357l*	5p	117145	117166	scaffold_84	plus	117102	117319	star1	TACCGACCGTTTGGGGCTCATT
MIR358	3p	14115793	14115814	scaffold_1	minus	14115752	14115955	mature2	TGGGCCCCGAACGGTCGGTAAC
MIR359	3p	1428401	1428421	scaffold_26	minus	1428369	1428623	mature1	TGGGCCGTAACCCGTCTCAGG
MIR360a	3p	10354	10376	scaffold_172	plus	10239	10405	mature1	TGGGCTGTGAGCTTTGGAAAACT
MIR360a*	5p	10272	10295	scaffold_172	plus	10239	10405	star1	TTTTCCCAAAGCCCTCAGCCCAGT
MIR360b	3p	13341	13363	scaffold_118	plus	13226	13392	mature1	TGGGCTGTGAGCTTTGGAAAACT
MIR360c	3p	19944	19966	scaffold_115	plus	19829	19995	mature1	TGGGCTGTGAGCTTTGGAAAACT
MIR360c*	5p	19862	19885	scaffold_115	plus	19829	19995	star1	TTTTCCCAAAGCCCTCAGCCCAGT
MIR360d	3p	1855	1877	scaffold_936	plus	1740	1906	mature1	TGGGCTGTGAGCTTTGGAAAACT
MIR360e	3p	14147990	14148012	scaffold_1	plus	14147875	14148043	mature1	TGGGCTGTGAGCTTTGGAAAACT
MIR360f	3p	137854	137876	scaffold_93	plus	137719	137930	mature1	TGGGCTGTGAGCTTTGGAAAACT
MIR360f*	5p	137772	137795	scaffold_93	plus	137719	137930	star1	TTTTCCCAAAGCCCTCAGCCCAGT
MIR361	3p	12924	12945	scaffold_3	plus	12753	12963	mature1	TGGGGGCATACTCTGGTGTGGC
MIR361*	5p	12776	12797	scaffold_3	plus	12753	12963	star1	TGCCTTGCGCCGGCGCATGCCC
MIR362	3p	1861553	1861577	scaffold_12	minus	1861518	1861704	mature1	TGGGGTTAGGGACTCCTGCTGAACA
MIR362*	5p	1861633	1861657	scaffold_12	minus	1861518	1861704	star1	CTCAGCAGGGTTGCCTAGCCCCAGC
MIR363	3p	733003	733024	scaffold_37	minus	732996	733244	mature1	TGGGTACCTGCGAGCAGAAGAG
MIR364	3p	873089	873109	scaffold_3	minus	873087	873145	mature1	TGGGTCCGGAGCGGCGGTAAG
MIR365	5p	397275	397295	scaffold_62	plus	397266	397327	mature1	TGGTAGGTTGGTCGTGCTGCC
MIR366	3p	2104464	2104485	scaffold_12	plus	2104328	2104497	mature1	TGGTCAGGACCATCCAACGGGC
MIR366*	5p	2104343	2104364	scaffold_12	plus	2104328	2104497	star1	CTGCTGGGTAGTCCTGACCACC
MIR367	3p	3881168	3881189	scaffold_6	plus	3881126	3881196	mature1	TGGTCGCTGCTGTTGCTGCTGC
MIR368	3p	104021	104044	scaffold_50	plus	103849	104081	mature1	TGGTGACGGTGGGGATGGCAACGG
MIR368*	5p	103853	103874	scaffold_50	plus	103849	104081	star1	TGTCGCCATCCCCTCCATCGCC
MIR369	5p	2375495	2375516	scaffold_4	plus	2375490	2375563	mature1	TGGTGGTTGGACTCCGGCAGCA
MIR370	3p	2509137	2509157	scaffold_11	minus	2509129	2509456	mature1	TGGTGTGAAGATATGCTGGCG
MIR370*	5p	2509427	2509447	scaffold_11	minus	2509129	2509456	star1	CCAGCATATCTTCACACCAAT
MIR371	3p	4012988	4013009	scaffold_5	minus	4012905	4013258	mature1	TGGTTGGGATGTCAAGATGGTT
MIR371*	5p	4013078	4013099	scaffold_5	minus	4012905	4013258	star1	CATTTGTGACATCCCAACGAGA
MIR372	5p	944620	944642	scaffold_19	minus	944520	944655	mature1	TGTACTTGTGCAGGGTCTCCTCT
MIR373	3p	2403024	2403043	scaffold_5	plus	2402794	2403117	mature1	TGTAGAAGTGACCCTTGGAC
MIR374	3p	18678	18698	scaffold_33	plus	18610	18713	mature1	TGTAGAGCCGTACTGTGTACC
MIR375	3p	124044	124066	scaffold_51	plus	123877	124081	mature1	TGTAGCGATGAGAGGAAAGAGGC
MIR375*	5p	123896	123917	scaffold_51	plus	123877	124081	star1	TCTTTCCTCTCATCGCTACAGC
MIR376	3p	1861531	1861552	scaffold_12	minus	1861518	1861704	mature2	TGTATGCATGTAGTCGGGACGG

MIR377	3p	1597828	1597850	scaffold_24	minus	1597782	1598074	mature1	TGTATGTTTTCTCCTTCGGTTGC
MIR378	3p	1367485	1367506	scaffold_23	plus	1367269	1367545	mature1	TGTCATCAAGTGCCCCAGCTGC
MIR378*	5p	1367300	1367321	scaffold_23	plus	1367269	1367545	star1	AGCTGTGGCACTTGACGATATT
MIR379a	5p	1446797	1446817	scaffold_25	plus	1446695	1447038	mature1	TGTCATGTCAACGGTCTCGCA
MIR379a*	3p	1446918	1446938	scaffold_25	plus	1446695	1447038	star1	TGCGAGACCGTTGACATGACA
MIR379b	3p	428239	428259	scaffold_21	minus	428235	428288	mature1	TGTCATGTCAACGGTCTCGCA
MIR379c	5p	1446918	1446938	scaffold_25	minus	1446682	1447041	mature1	TGTCATGTCAACGGTCTCGCA
MIR379c*	3p	1446797	1446817	scaffold_25	minus	1446682	1447041	star1	TGCGAGACCGTTGACATGACA
MIR380	3p	469925	469946	scaffold_38	minus	469890	470021	mature2	TGTCCACCATGTGATAAGAACA
MIR381	5p	431963	431984	scaffold_56	plus	431937	432245	mature2	TGTCCAGGGACCTGAAGGGGCT
MIR382	3p	1991821	1991841	scaffold_23	plus	1991648	1991863	mature1	TGTCCCGGTAACTGATGCACT
MIR382*	5p	1991670	1991690	scaffold_23	plus	1991648	1991863	star1	TGCATCAGTTGCCCGGACATG
MIR383a	5p	220944	220964	scaffold_4	plus	220903	221153	mature1	TGTCGATTACCGGGAATAGCA
MIR383a*	3p	221095	221115	scaffold_4	plus	220903	221153	star1	TGCTATTCCCGGTAATCGACA
MIR383b	5p	221095	221115	scaffold_4	minus	220903	221154	mature1	TGTCGATTACCGGGAATAGCA
MIR383b*	3p	220944	220964	scaffold_4	minus	220903	221154	star1	TGCTATTCCCGGTAATCGACA
MIR384	5p	364840	364858	scaffold_23	plus	364827	364926	mature1	TGTCTGAGCATAATAAGCT
MIR385	3p	163937	163958	scaffold_54	plus	163724	163978	mature1	TGTGAACGACTCTGGGGCTGGC
MIR386	5p	1381990	1382010	scaffold_2	plus	1381982	1382073	mature1	TGTGCAGCTGCCACCGCCGGC
MIR387	3p	1597153	1597173	scaffold_30	plus	1597023	1597195	mature1	TGTGCCTGCTGTGATGACTGC
MIR388	3p	1071257	1071278	scaffold_25	plus	1071164	1071282	mature1	TGTGCTGTCGTGCCGCTCGATG
MIR389	5p	709395	709414	scaffold_12	plus	709380	709663	mature1	TGTGGACGGACTTCAACGGG
MIR389*	3p	709639	709659	scaffold_12	plus	709380	709663	star1	TGAAGTCCTTCCACAGCCGGA
MIR390a	5p	367043	367063	scaffold_58	plus	367025	367187	mature1	TGTGGCCAGTTGCACCGTATG
MIR390a*	3p	367153	367173	scaffold_58	plus	367025	367187	star1	TACGGTGCAACTGGCCACACA
MIR390b	5p	367151	367171	scaffold_58	minus	367026	367188	mature1	TGTGGCCAGTTGCACCGTATG
MIR390b*	3p	367041	367061	scaffold_58	minus	367026	367188	star1	TACGGTGCAACTGGCCACACA
MIR391	3p	1482220	1482240	scaffold_32	minus	1482190	1482273	mature1	TGTGGGACGAGGCAGCAGACC
MIR392	5p	458561	458581	scaffold_41	plus	458530	458781	mature1	TGTGGGACGCGACTTATAAGC
MIR392*	3p	458732	458752	scaffold_41	plus	458530	458781	star1	TTATAAGTCGCGTCCCATAGC
MIR393	3p	66055	66075	scaffold_38	plus	65869	66105	mature1	TGTGGGGCGGCGCAAAGAACT
MIR394	3p	12901	12921	scaffold_3	plus	12753	12963	mature2	TGTGTACGGACGCTGTGCAGC
MIR395a	3p	52253	52274	scaffold_78	plus	52109	52277	mature2	TGTGTAGGGACGAAGGGAGAAG
MIR395a*	5p	52110	52131	scaffold_78	plus	52109	52277	star?	CTTTTCCTCCGTCTGGGAATGC
MIR395b	3p	1067437	1067460	scaffold_15	plus	1067293	1067461	mature2	TGTGTAGGGACGAAGGGAGAAGTT
MIR396	3p	1450545	1450565	scaffold_20	plus	1450285	1450626	mature2	TGTGTATGTGGGCGAAAATCT
MIR396*	5p	1450350	1450370	scaffold_20	plus	1450285	1450626	star2	TTTTCACCCACATGCACAACG
MIR397	3p	13802131	13802152	scaffold_1	minus	13802062	13802340	mature1	TGTGTTGGAATTGCGAGCAGCG
MIR398a	3p	805726	805747	scaffold_39	plus	805539	805782	mature1	TGTTAGTCCTCACCGAGGCAGG
MIR398b	3p	805601	805622	scaffold_39	minus	805533	805783	mature2	TGTTAGTCCTCACCGAGGCAGG
MIR399	3p	530705	530725	scaffold_15	minus	530684	530950	mature1	TGTTCAGATGAATCGTCCGTC
MIR399*	5p	530911	530932	scaffold_15	minus	530684	530950	star1	TGGACGGACGATTCATCTGACC
MIR400	5p	1341080	1341100	scaffold_26	plus	1341051	1341300	mature1	TGTTGAAATGTTGACCGTAGC
MIR401	3p	380097	380120	scaffold_8	plus	379878	380156	mature2	TGTTGCACCAAGGATCGAGGGACT
MIR402	3p	1563114	1563135	scaffold_18	minus	1563092	1563210	mature1	TGTTTGAGGAGTGATGAAATGG
MIR403	5p	218258	218280	scaffold_60	plus	218198	218396	mature2?	TTAACCCAGCTGATCGTTAACCC
MIR404a	3p	1385477	1385497	scaffold_8	plus	1385272	1385546	mature1	TTAAGAACAATGACCGGCATA
MIR404a*	5p	1385303	1385324	scaffold_8	plus	1385272	1385546	star1	TATGCCGGTCATTGTTCTTAAG
MIR404b	3p	1385302	1385323	scaffold_8	minus	1385263	1385527	mature1	TTAAGAACAATGACCGGCATAT
MIR404b*	5p	1385476	1385497	scaffold_8	minus	1385263	1385527	star1	TATGCCGGTCATTGTTCTTAAG
MIR405	3p	2206716	2206736	scaffold_8	minus	2206685	2206890	mature2	TTAAGCGAAGATGACGACTCC
MIR406	3p	7676064	7676084	scaffold_1	minus	7676008	7676278	mature1	TTAATCGGATGGGGATAAAGG
MIR407	3p	379918	379939	scaffold_8	minus	379870	380165	mature1	TTACACCAAAGGTCGAGCGACT
MIR407*	5p	380097	380118	scaffold_8	minus	379870	380165	star1	TCCCTCGATCCTTGGTGCAACA
MIR408a	5p	4503412	4503434	scaffold_1	minus	4503196	4503466	mature1	TTAGATTGCGAGACTCATGGAGA
MIR408a*	3p	4503218	4503240	scaffold_1	minus	4503196	4503466	star1	TCCATGAGTCTCGCAATCTAACA
MIR408b	5p	4503220	4503242	scaffold_1	plus	4503207	4503445	mature1	TTAGATTGCGAGACTCATGGAGA
MIR408b*	3p	4503414	4503436	scaffold_1	plus	4503207	4503445	star1	TCCATGAGTCTCGCAATCTAACA
MIR409	3p	1450566	1450589	scaffold_20	plus	1450285	1450626	mature1	TTAGGACAAAGGGGATATTTGGGC
MIR409*	5p	1450325	1450348	scaffold_20	plus	1450285	1450626	star1	CCAAACATCCCCTTTGTCCTAAAG
MIR410	3p	1052138	1052160	scaffold_5	plus	1052028	1052208	mature1	TTCACCCGTGGCAGCTACCCGGC
MIR411	5p	6163	6184	scaffold_54	plus	6109	6330	mature1	TTCACTGTGCAGCAGGTCTTGG
MIR412	3p	948906	948926	scaffold_23	minus	948820	949127	mature1	TTCCCGCTCTGACCCCATGCC
MIR412*	5p	949022	949042	scaffold_23	minus	948820	949127	star1	TGGCAAGGGGTCAGAACGGGA
MIR413	5p	3611742	3611763	scaffold_5	minus	3611556	3611879	mature1	TTCCGACGGTGCTGGTCCCACG
MIR413*	3p	3611671	3611692	scaffold_5	minus	3611556	3611879	star1	TGGGACCAGCACCATCGGAAGA
MIR414	3p	12674117	12674138	scaffold_1	minus	12674068	12674407	mature1	TTCGGCGAGAATGCCCTGCACC
MIR414*	5p	12674381	12674402	scaffold_1	minus	12674068	12674407	star1	TGCAGGGCATTCTCGCCGAATA
MIR415	3p	520662	520683	scaffold_31	minus	520637	520741	mature1	TTCGTCAAGCTGCTCACGCTGC
MIR416	3p	530880	530900	scaffold_15	plus	530690	530944	mature1	TTCGTTGACGTGAATCCCCCT
MIR416*	5p	530733	530753	scaffold_15	plus	530690	530944	star1	TGAGGGGGATTCACGTCAACT
MIR417	5p	7654214	7654233	scaffold_1	minus	7653849	7654271	mature2	TTCTCAGCGCTCTGCCCGGC
MIR418	5p	1963103	1963125	scaffold_15	minus	1962905	1963143	mature1	TTCTGGATATCAGAGGACACACA
MIR418*	3p	1962922	1962943	scaffold_15	minus	1962905	1963143	star1	TGTGTCCTCTGATATGCAGAAT
MIR419	5p	60660	60681	scaffold_66	plus	60650	60753	mature1	TTCTTATCACATGGTGGACAGG
MIR420a	5p	33606	33626	scaffold_86	plus	33594	33668	mature1	TTCTTCTTCTGGTTACGTAGC
MIR420b	5p	888109	888129	scaffold_9	minus	888066	888140	mature1	TTCTTCTTCTGGTTACGTAGC

MIR420c	5p	8192	8212	scaffold_38	plus	8180	8256	mature1	TTCTTCTTCTGGTTACGTAGC
MIR421	3p	432158	432179	scaffold_56	plus	431937	432245	mature1	TTGAACAAGTCGAGCGGCACTG
MIR421*	5p	431998	432019	scaffold_56	plus	431937	432245	star1	TATTTCACAGTGCCGCTCGACT
MIR422	3p	874574	874594	scaffold_24	minus	874558	874756	mature1	TTGAAGGGCTTAATCCACTGG
MIR423	5p	4503386	4503408	scaffold_1	minus	4503196	4503466	mature2	TTGACAGGGAAATACAGAAGATC
MIR423*	3p	4503241	4503261	scaffold_1	minus	4503196	4503466	star2	TGTATTTCCCTATCAAGGTTC
MIR424	3p	1853006	1853026	scaffold_27	minus	1852911	1853359	mature1	TTGACCAGTGGAATTCCGAGC
MIR424*	5p	1853241	1853262	scaffold_27	minus	1852911	1853359	star1	TCGGAATTCCATTGGTCAACGG
MIR425	5p	145576	145596	scaffold_40	plus	145536	145839	mature1	TTGACTTGTCGCAAATGGACC
MIR426	5p	104855	104876	scaffold_78	plus	104774	105084	mature1	TTGAGCTGGGATTTAGTGCAGA
MIR427	5p	1226785	1226807	scaffold_27	plus	1226679	1227017	mature1	TTGATGTAGAGCTTGCGACCGGG
MIR428	5p	1937459	1937479	scaffold_3	plus	1937371	1937630	mature1	TTGCATAACTGCCACCATAGA
MIR429	5p	1437642	1437663	scaffold_23	minus	1437556	1437665	mature1	TTGCCACCGTCGCCATCCCTGC
MIR429*	3p	1437557	1437578	scaffold_23	minus	1437556	1437665	star1	GGGGATGGTGATGGCGGCAGTG
MIR430	5p	4834897	4834918	scaffold_1	plus	4834891	4834951	mature1	TTGCGTGCGGTGCCATCGGTAT
MIR431	3p	2822256	2822277	scaffold_5	minus	2822187	2822451	mature1	TTGCTGCTAGACCTCGGTCCGG
MIR432	3p	4726173	4726193	scaffold_3	minus	4726163	4726384	mature1	TTGGCAAATCTCTGCAAGGCA
MIR432*	5p	4726350	4726371	scaffold_3	minus	4726163	4726384	star1	TGCCTTGCAGAGATTTGCTAAG
MIR433	5p	3520136	3520157	scaffold_4	plus	3520095	3520350	mature1	TTGGCAGGCTCCGGAGCGGACT
MIR434	3p	9765	9785	scaffold_31	plus	9583	9822	mature1	TTGGGTCGCCGAAGACATTCC
MIR434*	5p	9625	9645	scaffold_31	plus	9583	9822	star1	TGTCCTCGTCAACCCAATGCA
MIR435	3p	7601	7621	scaffold_86	plus	7511	7706	mature1	TTGGTGGTGCTTTGCACGGCA
MIR436	5p	293642	293662	scaffold_54	plus	293636	293695	mature1	TTGTATACTGTAATTTTAGTT
MIR437	5p	430206	430226	scaffold_40	minus	430127	430227	mature1	TTGTCAAAGTCAAAGAGTGGG
MIR438	5p	1728477	1728498	scaffold_27	minus	1728260	1728568	mature1	TTGTCTGCGGACGAGGTCGAGA
MIR438*	3p	1728336	1728357	scaffold_27	minus	1728260	1728568	star1	TGCTCTTCTCGACCTCGTCCGC
MIR439	5p	516710	516730	scaffold_12	minus	516508	516786	mature1	TTGTTGGTGTCAGTTTCCTGA
MIR439*	3p	516562	516582	scaffold_12	minus	516508	516786	star1	AGGAAACTGACACCAACAAGT
MIR440	5p	2680793	2680812	scaffold_3	plus	2680791	2680897	mature1	TTGTTGTCGAGCTGATTGGG
MIR441	5p	238379	238400	scaffold_13	plus	238352	238683	mature1	TTTAAATGCTGTTGCCTTGGCC
MIR442	3p	173331	173351	scaffold_17	plus	173107	173372	mature2	TTTCAATCTCAACTTAGACTT
MIR442*	5p	173128	173150	scaffold_17	plus	173107	173372	star2	TATGTCTAAGTTGAGATCGAAAT
MIR443	3p	7653905	7653926	scaffold_1	minus	7653849	7654271	mature1	TTTCTGAACCGCAGTGGAAAGC
MIR444a	3p	430487	430508	scaffold_40	plus	430264	430542	mature1	TTTCTTCATCGCTACCTCTGAG
MIR444b	3p	430300	430321	scaffold_40	minus	430258	430543	mature1	TTTCTTCATCGCTACCTCTGAG
MIR445a	3p	541208	541229	scaffold_20	minus	541204	541297	mature1	TTTGCAAGACGGTTGGAGTGGG
MIR445b	3p	265280	265301	scaffold_31	plus	265211	265304	mature1	TTTGCAAGACGGTTGGAGTGGG
MIR446a	3p	114981	115001	scaffold_49	plus	114893	115038	mature1	TTTGGCCTGTCTGCGGAGATG
MIR446b	3p	1333521	1333541	scaffold_5	plus	1333482	1333549	mature1	TTTGGCCTGTCTGCGGAGATG
MIR447a	3p	594518	594538	scaffold_38	minus	594473	594770	mature1	TTTGGTAGCTGTGTTGAGACA
MIR447b	3p	594706	594726	scaffold_38	plus	594449	594794	mature1	TTTGGTAGCTGTGTTGAGACA
MIR448	5p	842816	842837	scaffold_6	plus	842709	843072	mature1	TTTGTCATGTCAACGGTCTCGC
MIR449	3p	1326423	1326444	scaffold_2	minus	1326323	1326644	mature1	TGAGTGTGAGTCCTGAGCTGGG
MIR449*	5p	1326516	1326538	scaffold_2	minus	1326323	1326644	star1	TCAGGACTCACACTCATGTCTGT
