# Supplementary material for: Gene silencing pathways found in the green alga Volvox carteri reveal insights into evolution and origins of small RNA systems in plants
Source: BMC Genomics. 2016 Nov 2;17:853. doi: 10.1186/s12864-016-3202-4 (PMC5093975; doi:10.1186/s12864-016-3202-4)
Supplement: Additional file 6: Figure S4. — Small RNA coverage of repetitive elements. All seven small RNA libraries (VcAGO3 IP (“AgoIP”), vegetative somatic cells (“asx_som”), induced somatic cells (“ind_som”), female somatic cells (“fem_som”), vegetative gonidia (“asx_gon”), induced gonidia (“ind_gon”) and female egg cells (“fem_egg”) were mapped to the consensus sequence of all repetitive elements deposited in Repbase19.02 (see also main Figs. 3 and 4). Each page shows the coverage of one repetitive element. Each graph depicts the read abundance, i.e. coverage by nucleotide (y-axis) over the length of the repetitive element (x-axis). Coverage on the plus strand is depicted in red, coverage on the minus strand in blue. Ctot = total count. (PDF 2101 kb) [file 12864_2016_3202_MOESM6_ESM.pdf]

Dueck_FigS4

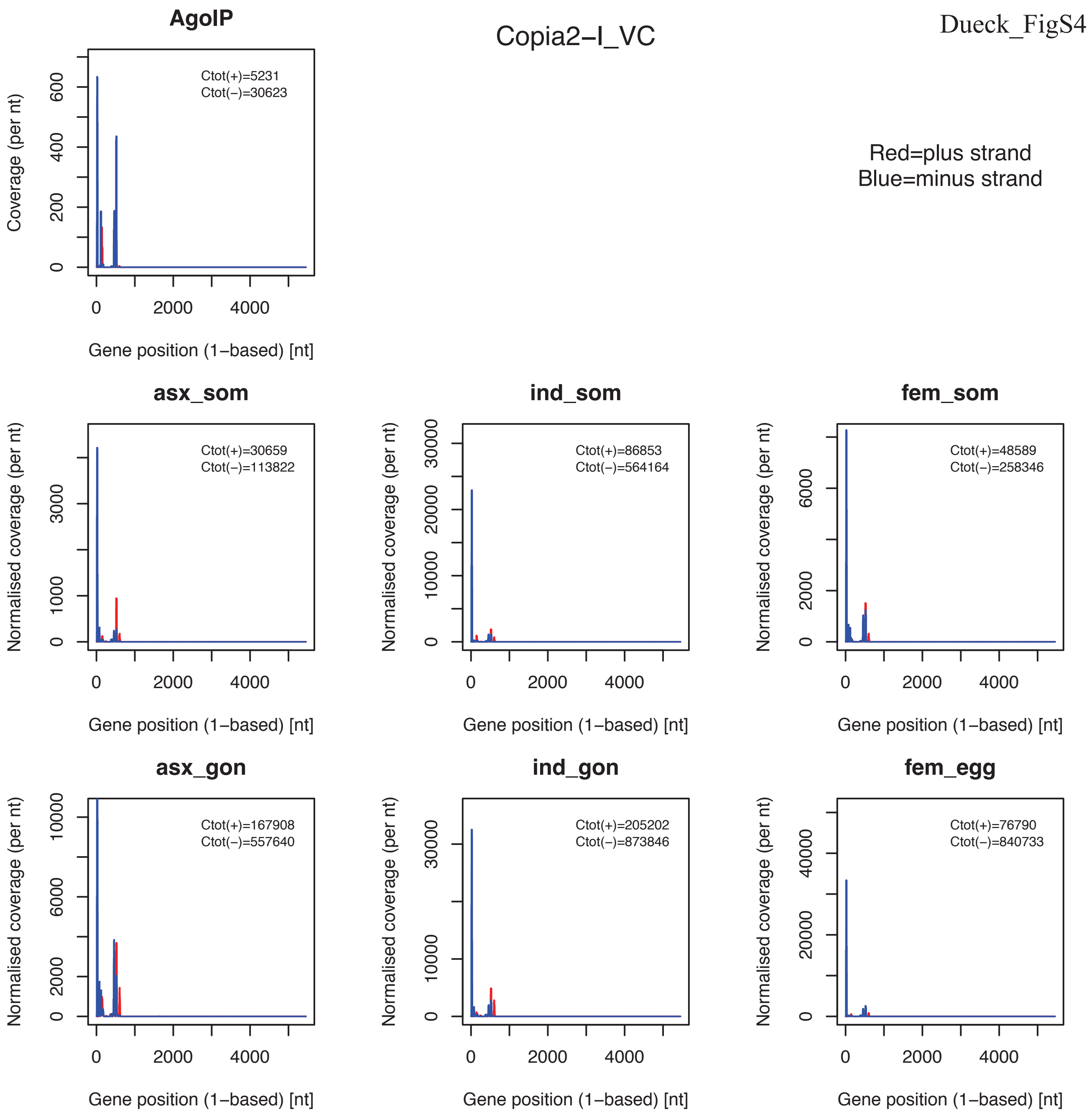
Copia2−I_VC
Red=plus strand
Blue=minus strand
AgoIP
Ctot(+)=5231
Ctot(−)=30623
Coverage (per nt)
Gene position (1−based) [nt]
asx_som
Ctot(+)=30659
Ctot(−)=113822
ind_som
Ctot(+)=86853
Ctot(−)=564164
fem_som
Ctot(+)=48589
Ctot(−)=258346
asx_gon
Ctot(+)=167908
Ctot(−)=557640
ind_gon
Ctot(+)=205202
Ctot(−)=873846
fem_egg
Ctot(+)=76790
Ctot(−)=840733
Normalised coverage (per nt)
Gene position (1−based) [nt]

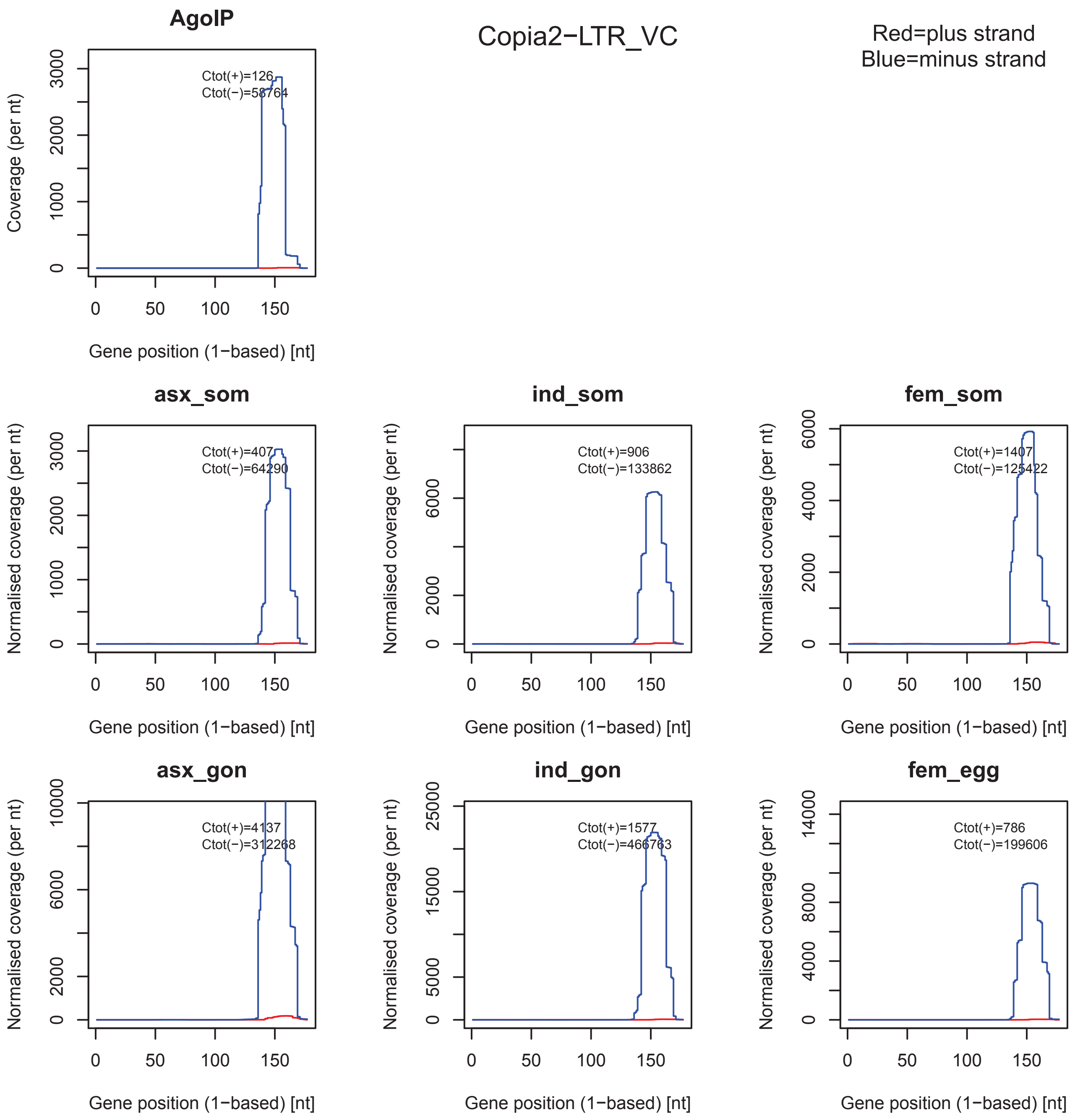

Copia2−LTR_VC
Red=plus strand
Blue=minus strand
AgoIP
Ctot(+)=126
Ctot(−)=58764
Coverage (per nt)
asx_som
Ctot(+)=407
Ctot(−)=64290
ind_som
Ctot(+)=906
Ctot(−)=133862
fem_som
Ctot(+)=1407
Ctot(−)=125422
asx_gon
Ctot(+)=4137
Ctot(−)=312268
ind_gon
Ctot(+)=1577
Ctot(−)=466763
fem_egg
Ctot(+)=786
Ctot(−)=199606
Normalised coverage (per nt)
Gene position (1−based) [nt]

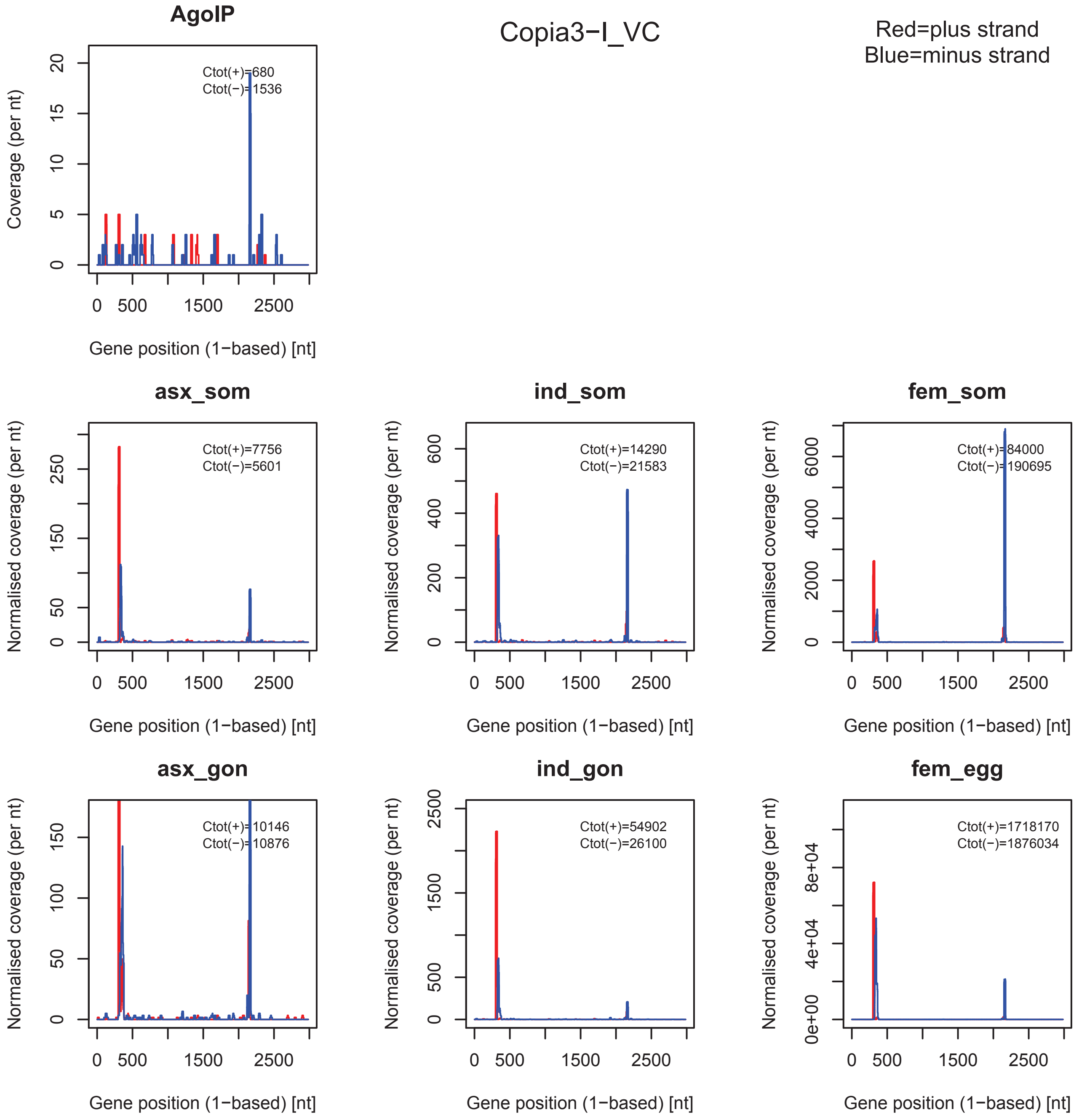

Copia3−I_VC
Red=plus strand
Blue=minus strand
AgoIP
Ctot(+)=680
Ctot(−)=1536
asx_som
Ctot(+)=7756
Ctot(−)=5601
ind_som
Ctot(+)=14290
Ctot(−)=21583
fem_som
Ctot(+)=84000
Ctot(−)=190695
asx_gon
Ctot(+)=10146
Ctot(−)=10876
ind_gon
Ctot(+)=54902
Ctot(−)=26100
fem_egg
Ctot(+)=1718170
Ctot(−)=1876034
Coverage (per nt)
Normalised coverage (per nt)
Gene position (1−based) [nt]

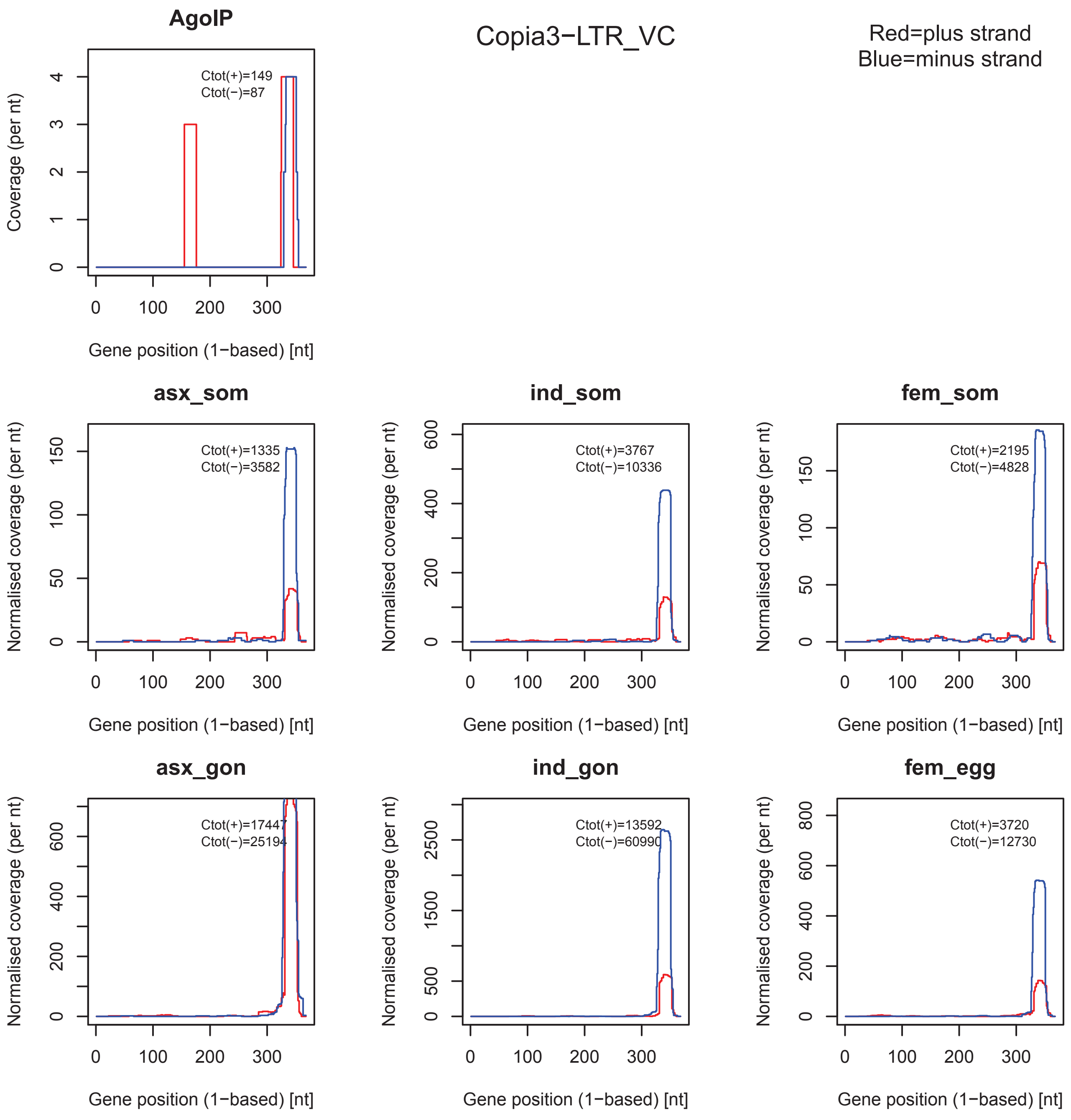
Copia3−LTR_VC
Red=plus strand
Blue=minus strand
AgoIP
Ctot(+)=149
Ctot(−)=87
Coverage (per nt)
asx_som
Ctot(+)=1335
Ctot(−)=3582
ind_som
Ctot(+)=3767
Ctot(−)=10336
fem_som
Ctot(+)=2195
Ctot(−)=4828
asx_gon
Ctot(+)=17447
Ctot(−)=25194
ind_gon
Ctot(+)=13592
Ctot(−)=60990
fem_egg
Ctot(+)=3720
Ctot(−)=12730
Normalised coverage (per nt)
Gene position (1−based) [nt]

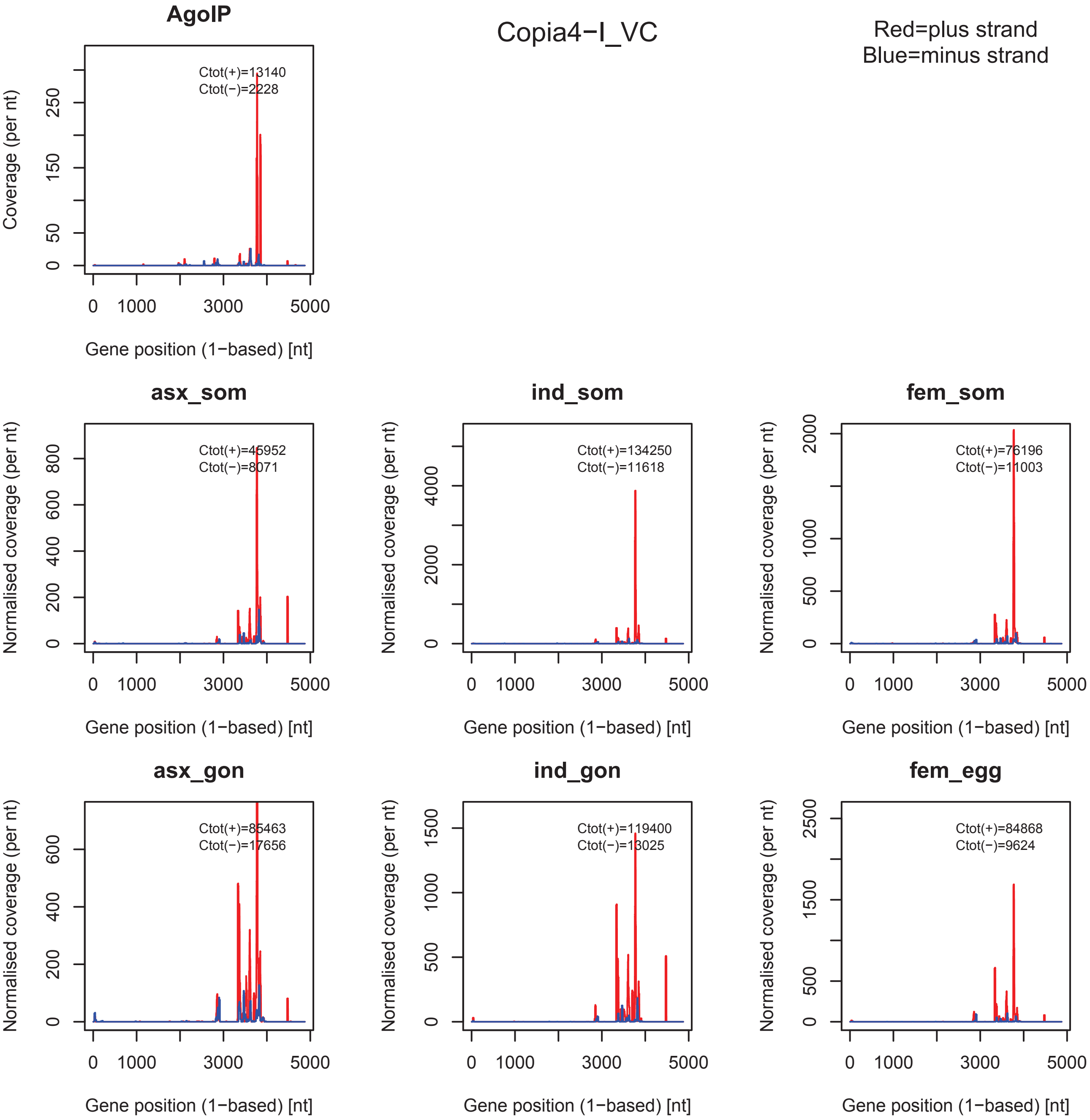

Copia4−I_VC
Red=plus strand
Blue=minus strand
AgoIP
Ctot(+)=13140
Ctot(−)=2228
Coverage (per nt)
Gene position (1−based) [nt]
asx_som
Ctot(+)=45952
Ctot(−)=8071
ind_som
Ctot(+)=134250
Ctot(−)=11618
fem_som
Ctot(+)=76196
Ctot(−)=11003
asx_gon
Ctot(+)=85463
Ctot(−)=17656
ind_gon
Ctot(+)=119400
Ctot(−)=13025
fem_egg
Ctot(+)=84868
Ctot(−)=9624
Normalised coverage (per nt)
Gene position (1−based) [nt]

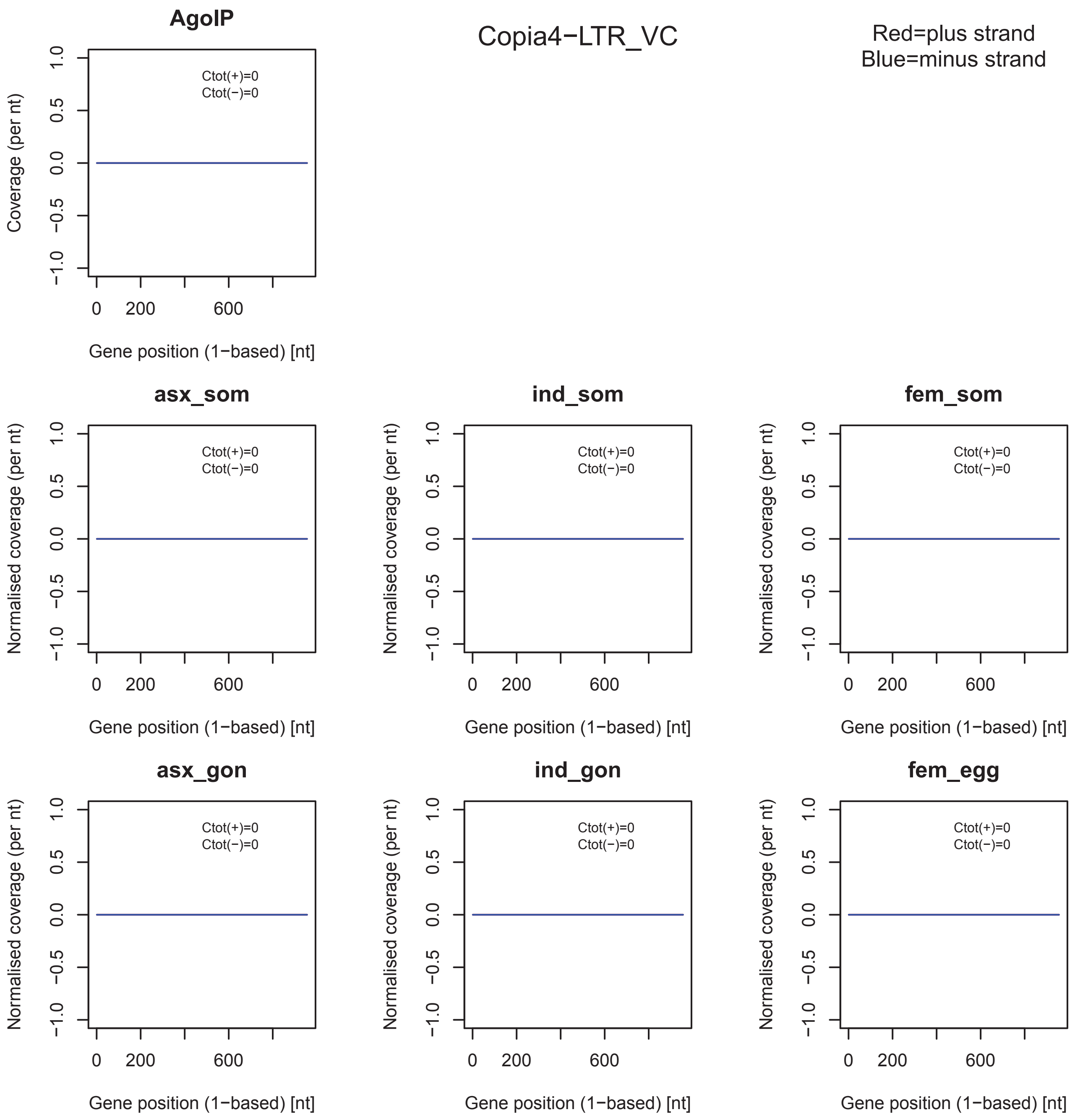
Copia4−LTR_VC
Red=plus strand
Blue=minus strand
AgoIP
Coverage (per nt)
Ctot(+)=0
Ctot(−)=0
Gene position (1−based) [nt]
asx_som
ind_som
fem_som
asx_gon
ind_gon
fem_egg
Normalised coverage (per nt)
1.0
0.5
0.0
−0.5
−1.0
0
200
600

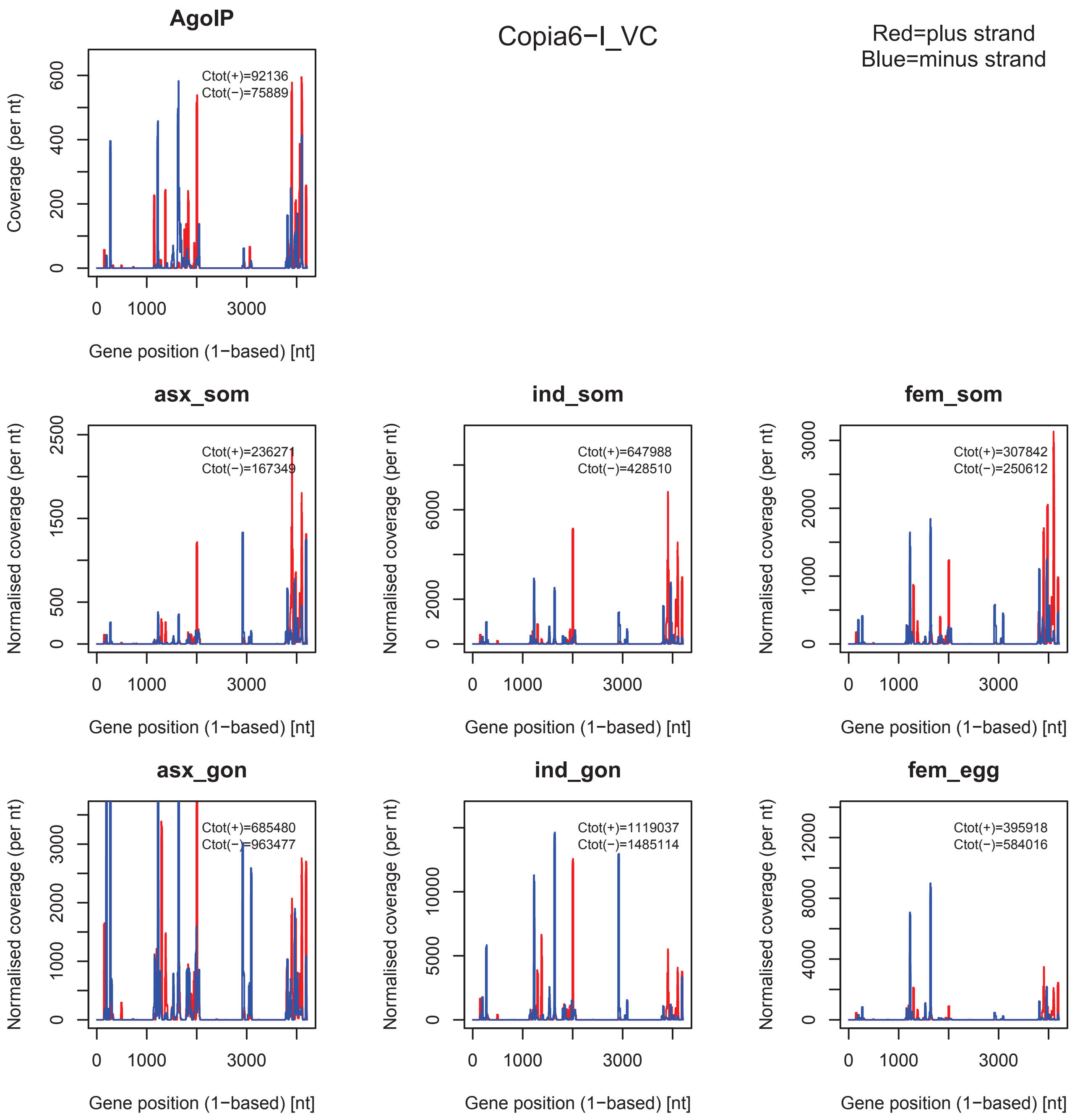

Copia6−I_VC
Red=plus strand
Blue=minus strand
AgoIP
Ctot(+)=92136
Ctot(−)=75889
Coverage (per nt)
0
200
400
600
Gene position (1−based) [nt]
0
1000
3000
asx_som
Ctot(+)=236271
Ctot(−)=167349
Normalised coverage (per nt)
0
500
1500
2500
ind_som
Ctot(+)=647988
Ctot(−)=428510
0
2000
6000
fem_som
Ctot(+)=307842
Ctot(−)=250612
0
1000
2000
3000
asx_gon
Ctot(+)=685480
Ctot(−)=963477
0
1000
2000
3000
ind_gon
Ctot(+)=1119037
Ctot(−)=1485114
0
5000
10000
fem_egg
Ctot(+)=395918
Ctot(−)=584016
0
4000
8000
12000

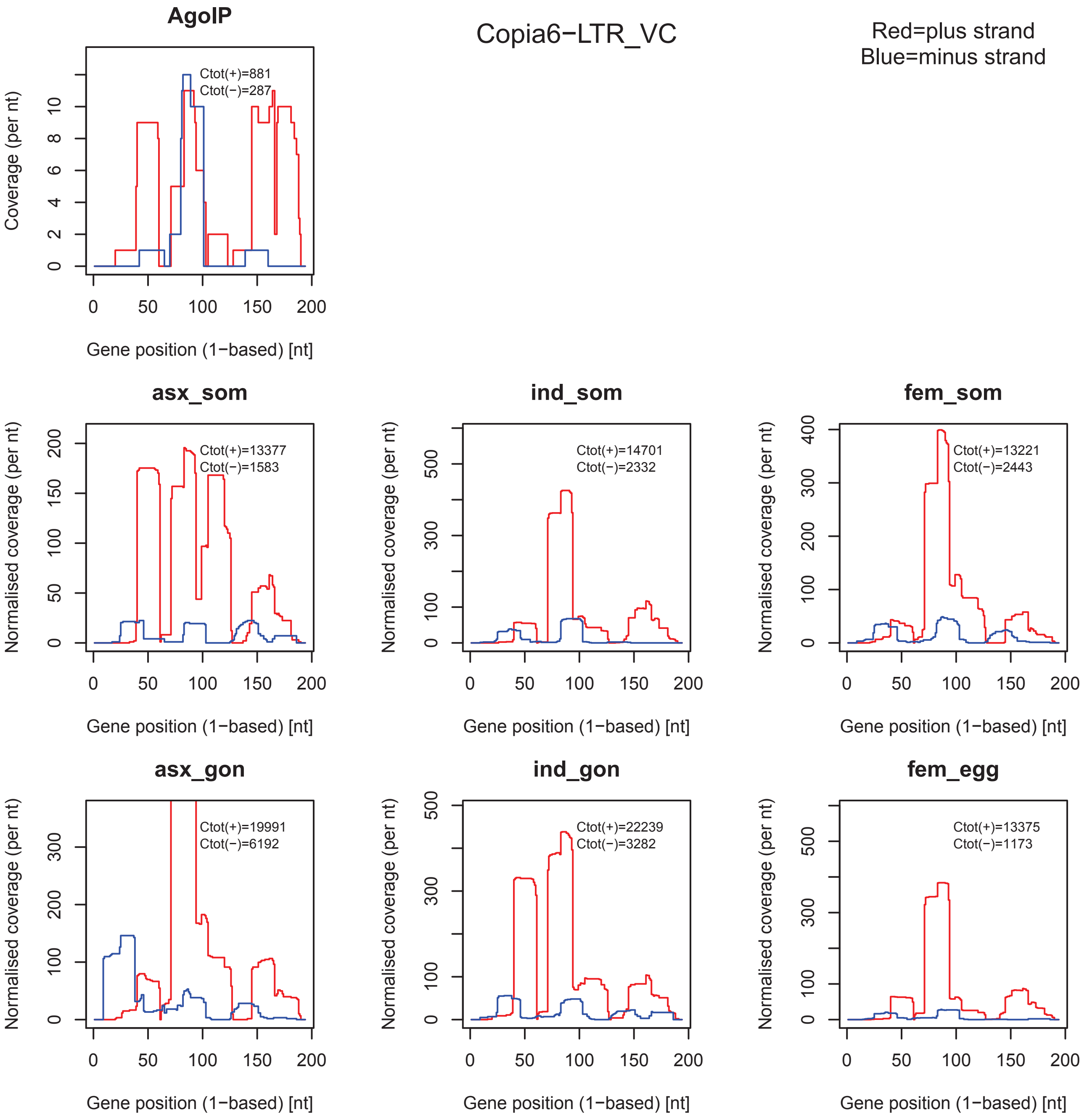
Copia6−LTR_VC
Red=plus strand
Blue=minus strand
AgoIP
Ctot(+)=881
Ctot(−)=287
Coverage (per nt)
Gene position (1−based) [nt]
asx_som
Ctot(+)=13377
Ctot(−)=1583
ind_som
Ctot(+)=14701
Ctot(−)=2332
fem_som
Ctot(+)=13221
Ctot(−)=2443
asx_gon
Ctot(+)=19991
Ctot(−)=6192
ind_gon
Ctot(+)=22239
Ctot(−)=3282
fem_egg
Ctot(+)=13375
Ctot(−)=1173
Normalised coverage (per nt)
Gene position (1−based) [nt]

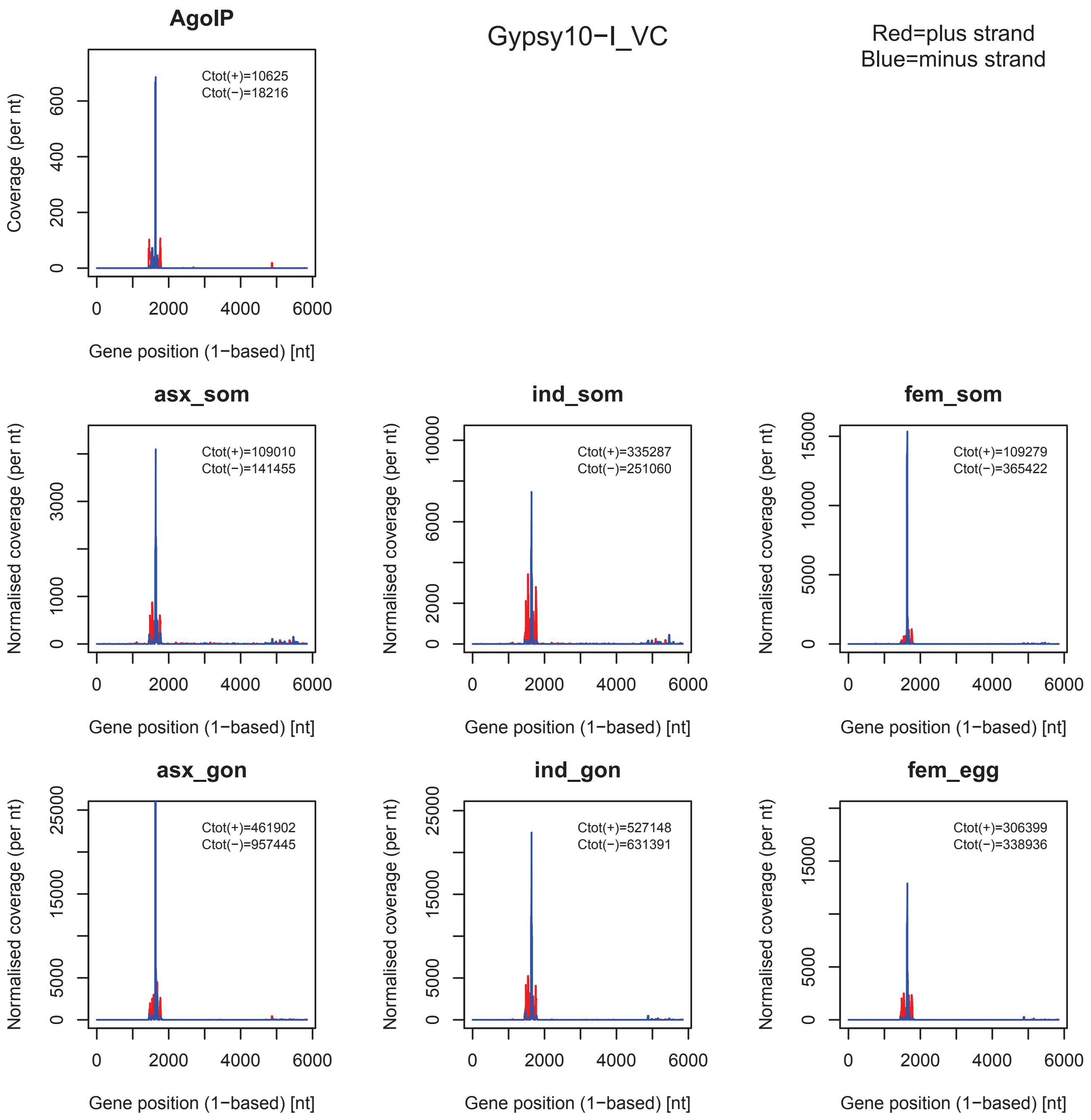

Gypsy10−I_VC
Red=plus strand
Blue=minus strand
AgoIP
Ctot(+)=10625
Ctot(−)=18216
Coverage (per nt)
Gene position (1−based) [nt]
asx_som
Ctot(+)=109010
Ctot(−)=141455
ind_som
Ctot(+)=335287
Ctot(−)=251060
fem_som
Ctot(+)=109279
Ctot(−)=365422
asx_gon
Ctot(+)=461902
Ctot(−)=957445
ind_gon
Ctot(+)=527148
Ctot(−)=631391
fem_egg
Ctot(+)=306399
Ctot(−)=338936
Normalised coverage (per nt)
Gene position (1−based) [nt]

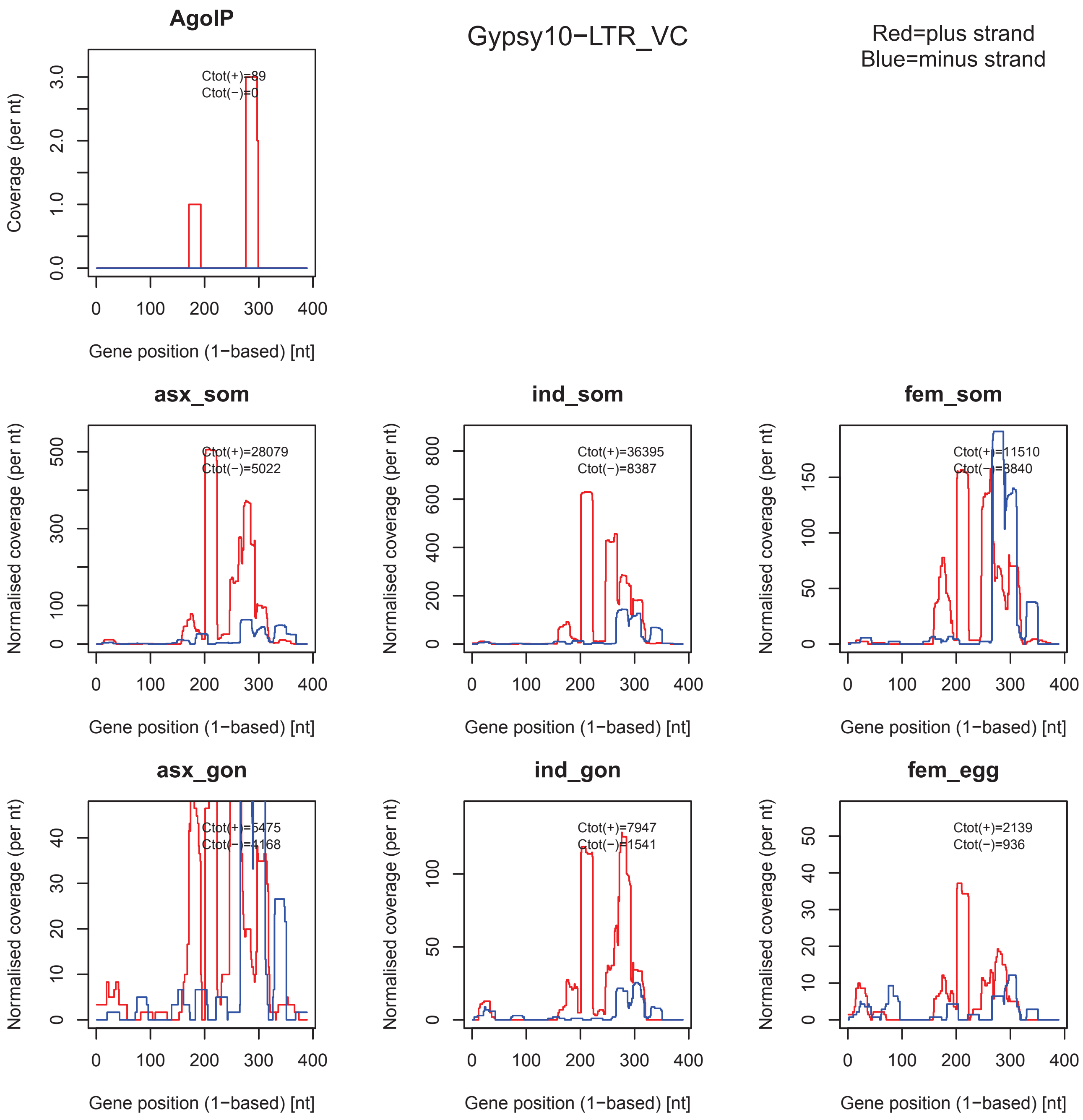
Gypsy10−LTR_VC
Red=plus strand
Blue=minus strand
AgoIP
Ctot(+)=89
Ctot(−)=0
Coverage (per nt)
Gene position (1−based) [nt]
asx_som
Ctot(+)=28079
Ctot(−)=5022
ind_som
Ctot(+)=36395
Ctot(−)=8387
fem_som
Ctot(+)=11510
Ctot(−)=8840
asx_gon
Ctot(+)=5475
Ctot(−)=4168
ind_gon
Ctot(+)=7947
Ctot(−)=1541
fem_egg
Ctot(+)=2139
Ctot(−)=936
Normalised coverage (per nt)
Gene position (1−based) [nt]

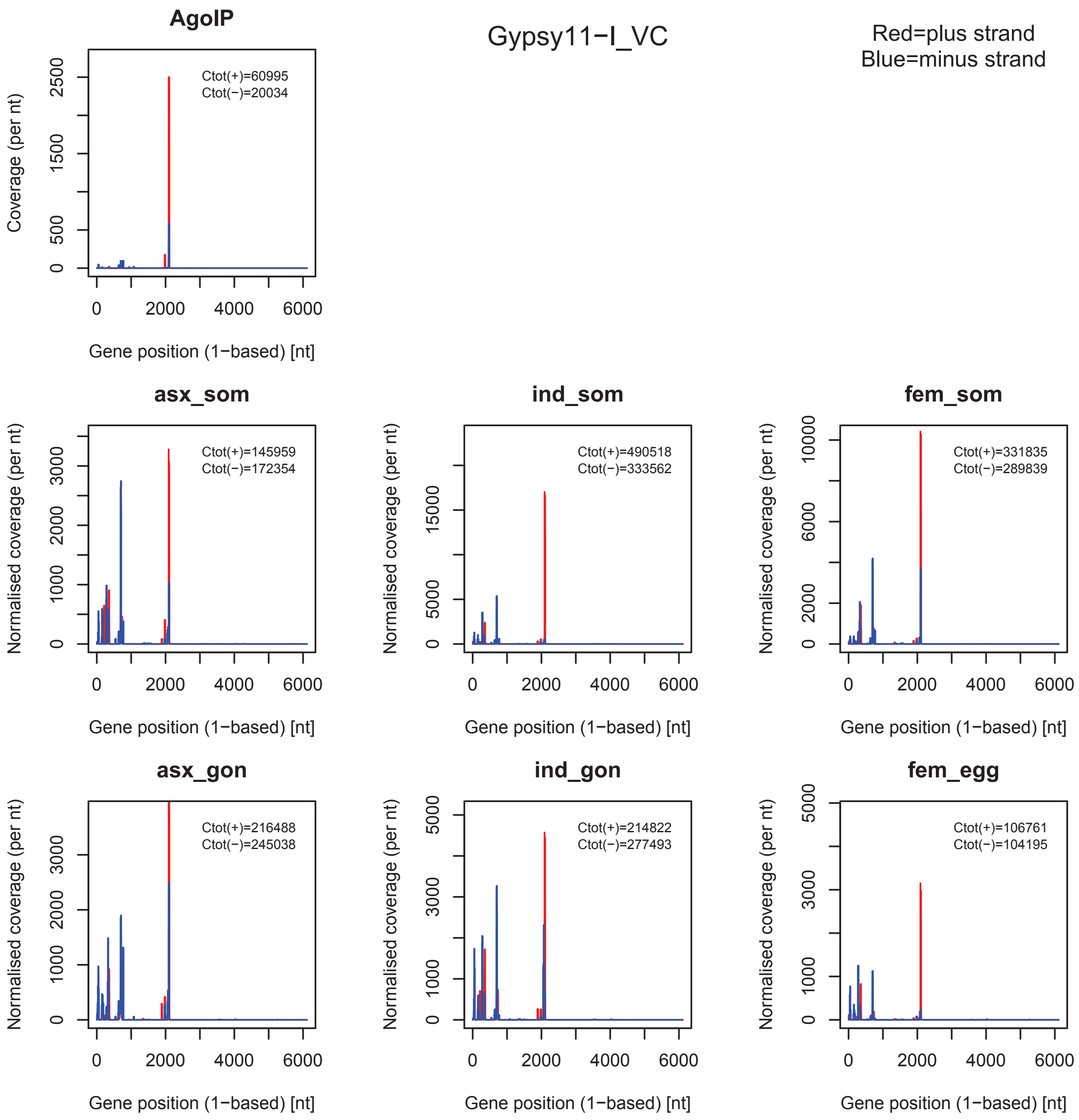

Gypsy11−I_VC
Red=plus strand
Blue=minus strand
AgoIP
Ctot(+)=60995
Ctot(−)=20034
Coverage (per nt)
asx_som
Ctot(+)=145959
Ctot(−)=172354
ind_som
Ctot(+)=490518
Ctot(−)=333562
fem_som
Ctot(+)=331835
Ctot(−)=289839
asx_gon
Ctot(+)=216488
Ctot(−)=245038
ind_gon
Ctot(+)=214822
Ctot(−)=277493
fem_egg
Ctot(+)=106761
Ctot(−)=104195
Normalised coverage (per nt)
Gene position (1−based) [nt]

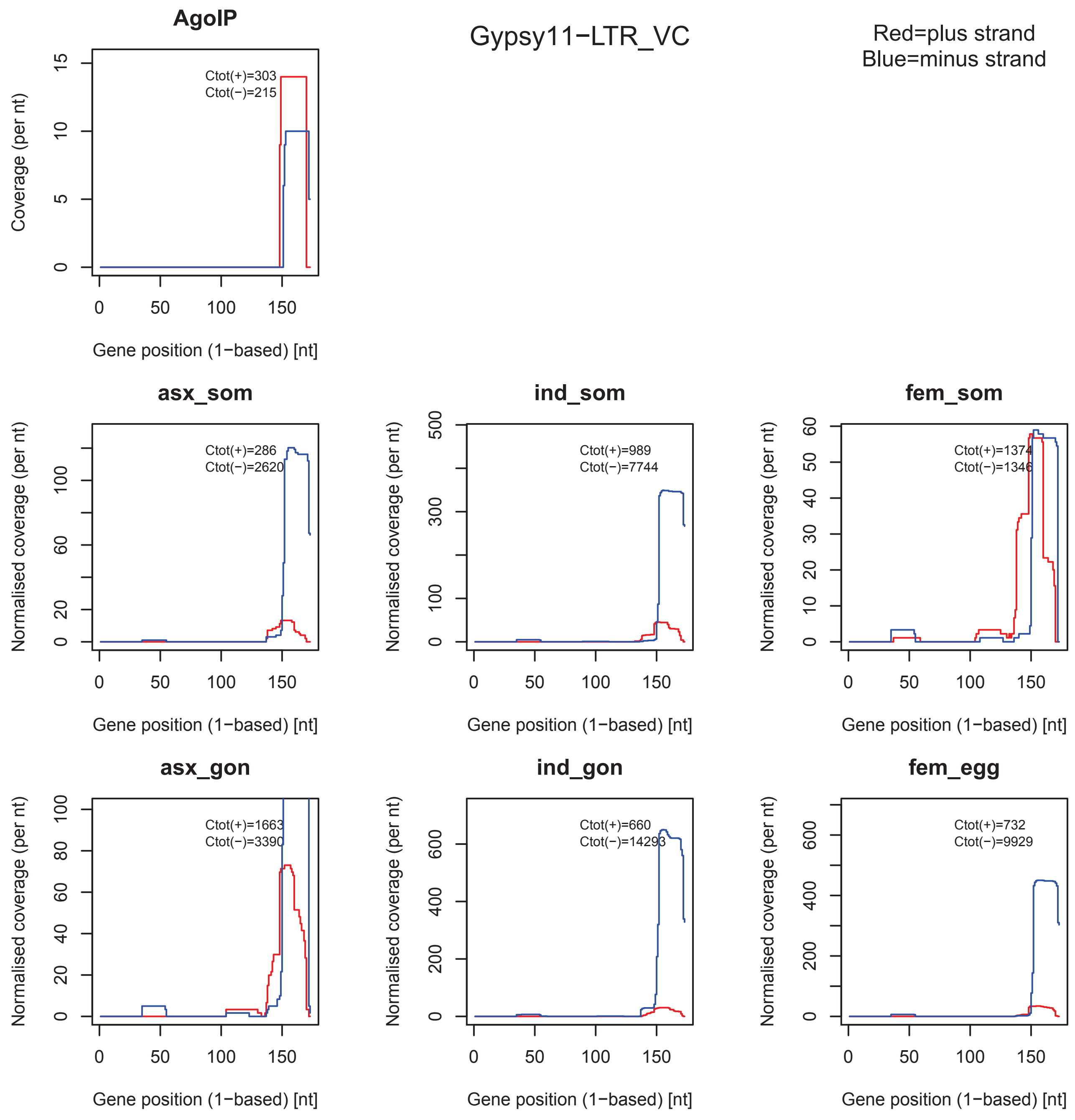
Gypsy11−LTR_VC
Red=plus strand
Blue=minus strand
AgoIP
Ctot(+)=303
Ctot(−)=215
Coverage (per nt)
asx_som
Ctot(+)=286
Ctot(−)=2620
ind_som
Ctot(+)=989
Ctot(−)=7744
fem_som
Ctot(+)=1374
Ctot(−)=1346
asx_gon
Ctot(+)=1663
Ctot(−)=3390
ind_gon
Ctot(+)=660
Ctot(−)=14293
fem_egg
Ctot(+)=732
Ctot(−)=9929
Normalised coverage (per nt)
Gene position (1−based) [nt]

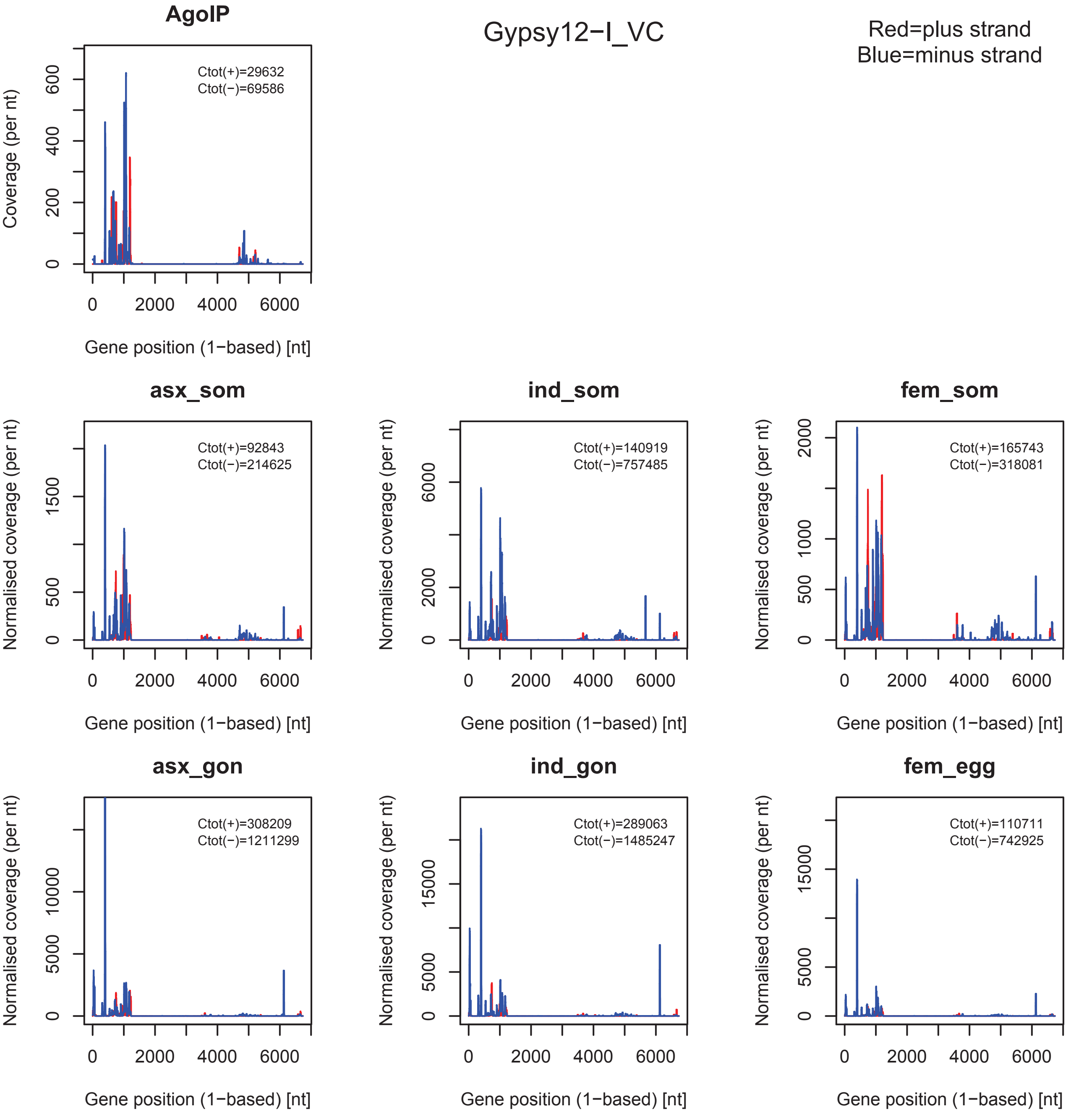

Gypsy12−I_VC
Red=plus strand
Blue=minus strand
AgoIP
Ctot(+)=29632
Ctot(−)=69586
Coverage (per nt)
Gene position (1−based) [nt]
asx_som
Ctot(+)=92843
Ctot(−)=214625
ind_som
Ctot(+)=140919
Ctot(−)=757485
fem_som
Ctot(+)=165743
Ctot(−)=318081
asx_gon
Ctot(+)=308209
Ctot(−)=1211299
ind_gon
Ctot(+)=289063
Ctot(−)=1485247
fem_egg
Ctot(+)=110711
Ctot(−)=742925
Normalised coverage (per nt)
Gene position (1−based) [nt]

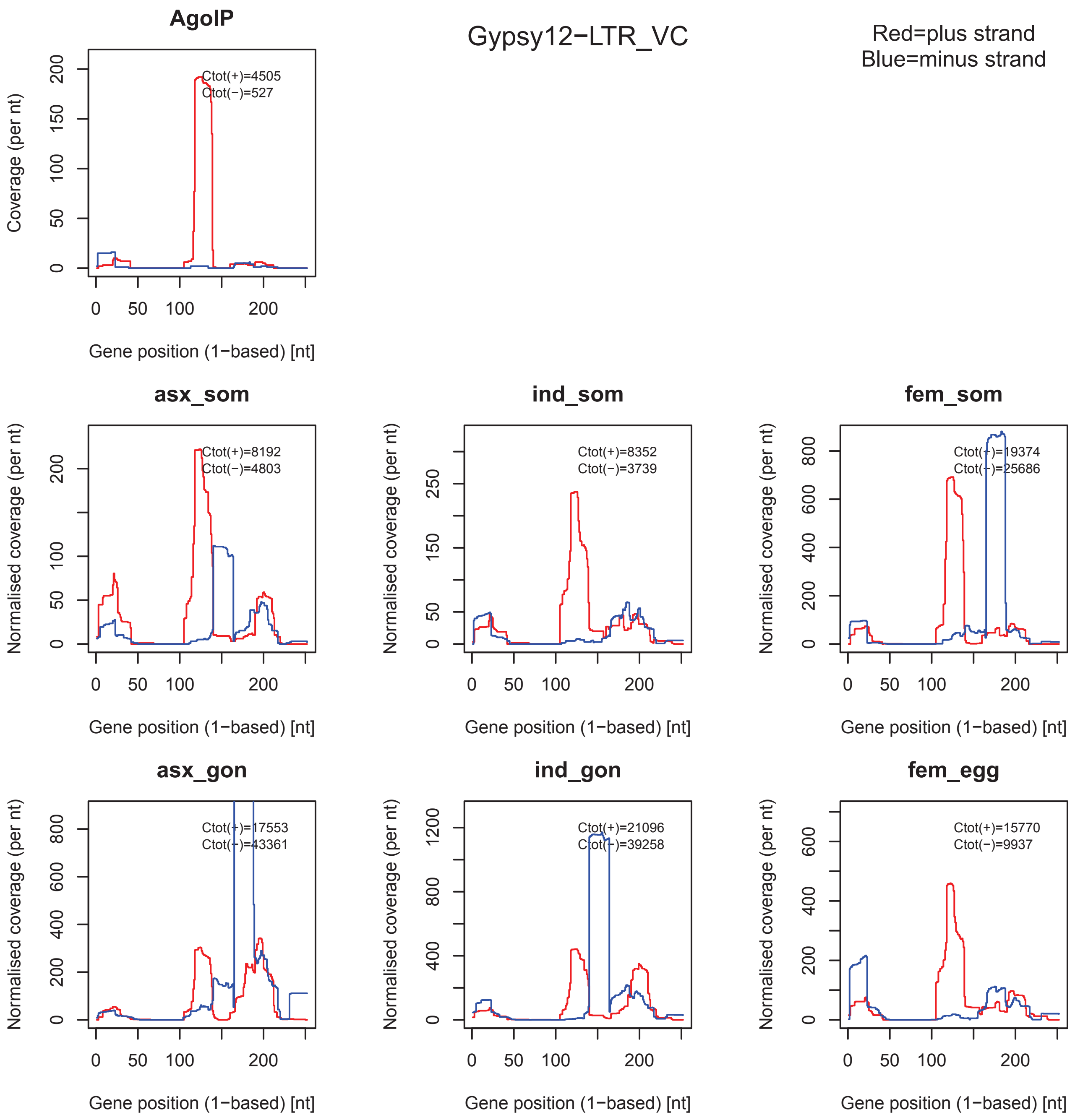

AgoIP
Gypsy12−LTR_VC
Red=plus strand
Blue=minus strand
Coverage (per nt)
Ctot(+)=4505
Ctot(−)=527
Gene position (1−based) [nt]
asx_som
Ctot(+)=8192
Ctot(−)=4803
ind_som
Ctot(+)=8352
Ctot(−)=3739
fem_som
Ctot(+)=19374
Ctot(−)=25686
asx_gon
Ctot(+)=17553
Ctot(−)=43361
ind_gon
Ctot(+)=21096
Ctot(−)=39258
fem_egg
Ctot(+)=15770
Ctot(−)=9937
Normalised coverage (per nt)
Gene position (1−based) [nt]

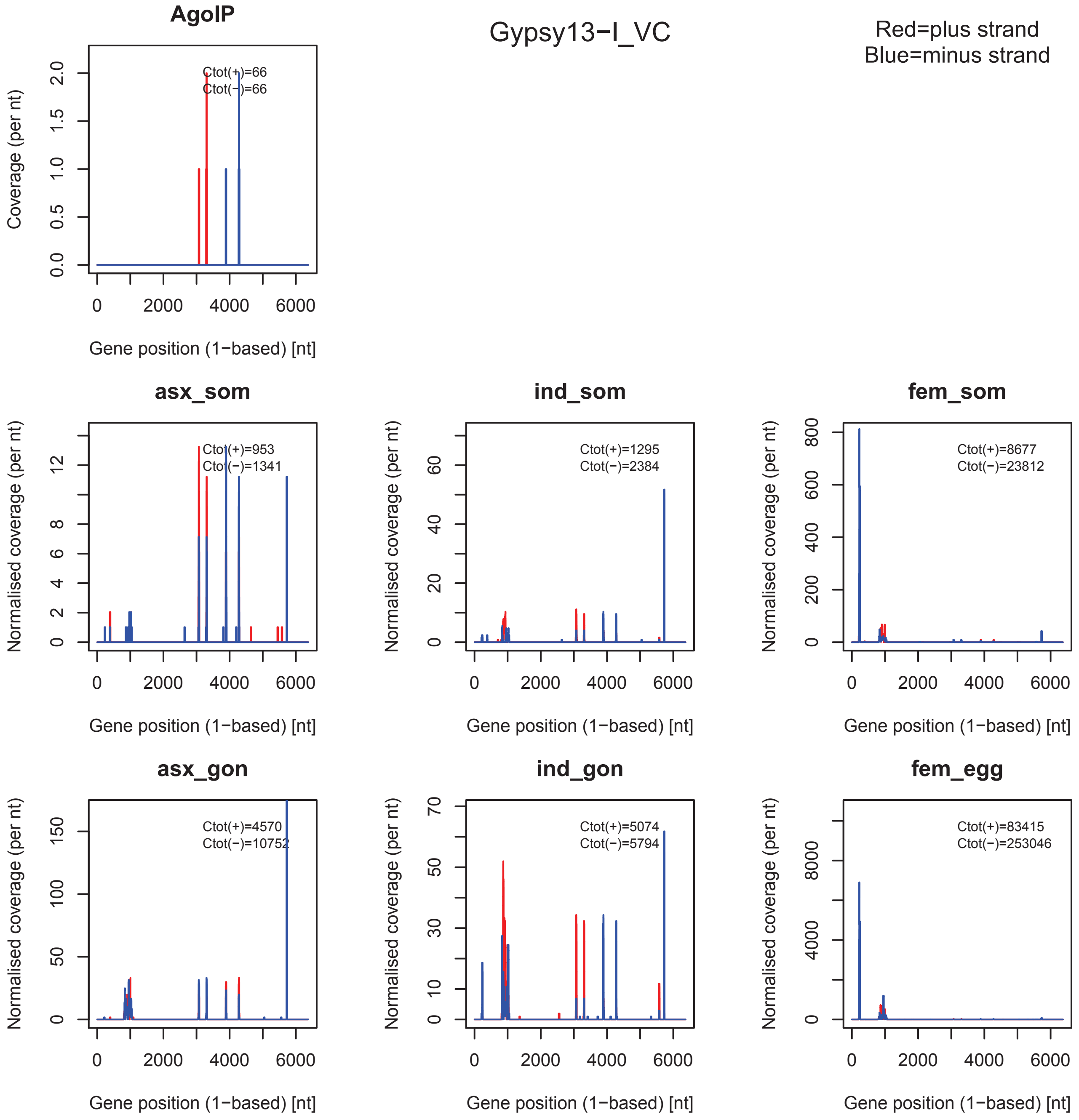

Gypsy13−I_VC
Red=plus strand
Blue=minus strand
AgoIP
Ctot(+)=66
Ctot(−)=66
Coverage (per nt)
Gene position (1−based) [nt]
asx_som
Ctot(+)=953
Ctot(−)=1341
ind_som
Ctot(+)=1295
Ctot(−)=2384
fem_som
Ctot(+)=8677
Ctot(−)=23812
asx_gon
Ctot(+)=4570
Ctot(−)=10752
ind_gon
Ctot(+)=5074
Ctot(−)=5794
fem_egg
Ctot(+)=83415
Ctot(−)=253046
Normalised coverage (per nt)

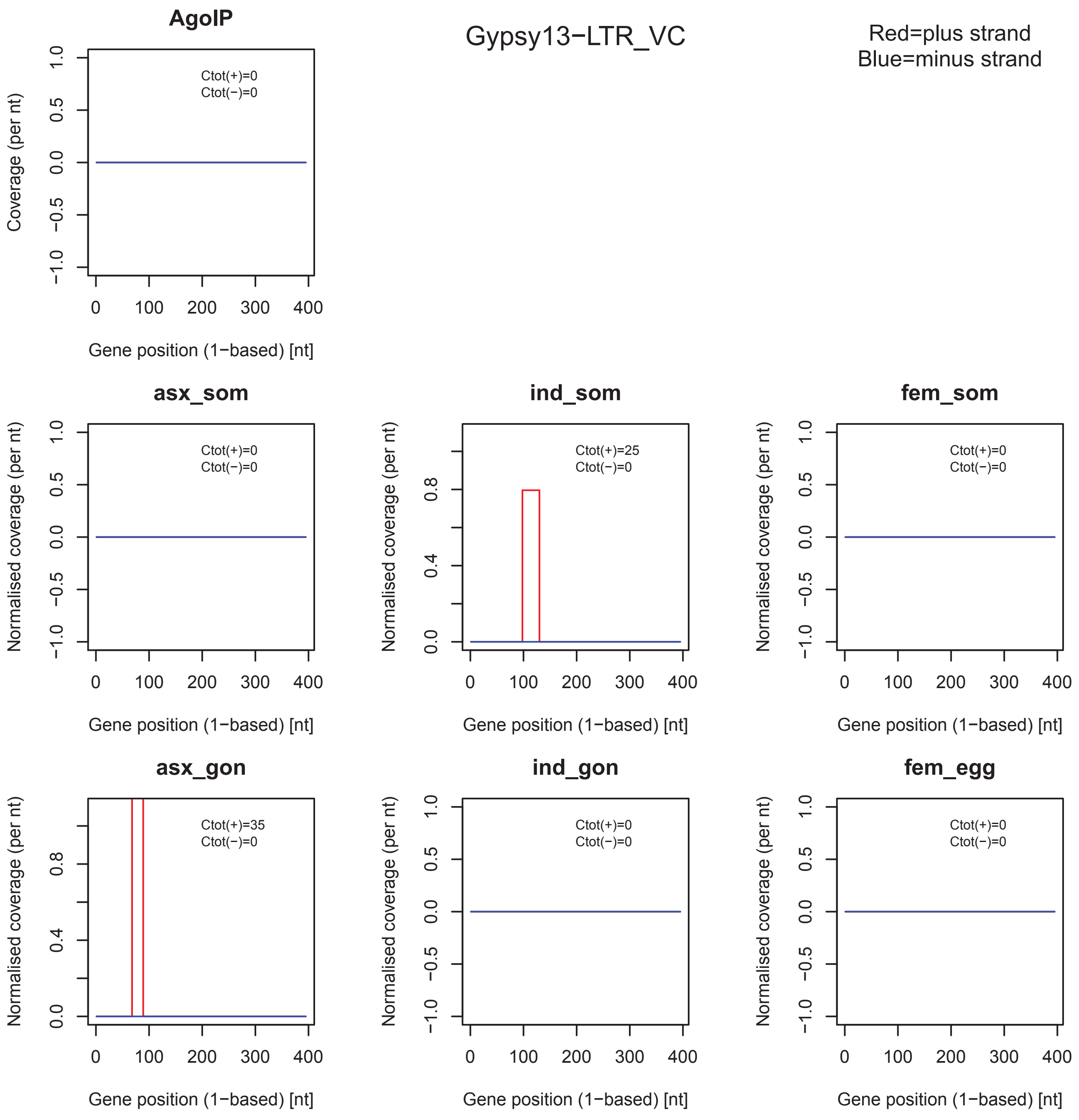

Gypsy13−LTR_VC
Red=plus strand
Blue=minus strand
AgoIP
Ctot(+)=0
Ctot(−)=0
Coverage (per nt)
Gene position (1−based) [nt]
asx_som
Ctot(+)=0
Ctot(−)=0
ind_som
Ctot(+)=25
Ctot(−)=0
fem_som
Ctot(+)=0
Ctot(−)=0
asx_gon
Ctot(+)=35
Ctot(−)=0
ind_gon
Ctot(+)=0
Ctot(−)=0
fem_egg
Ctot(+)=0
Ctot(−)=0
Normalised coverage (per nt)
Gene position (1−based) [nt]

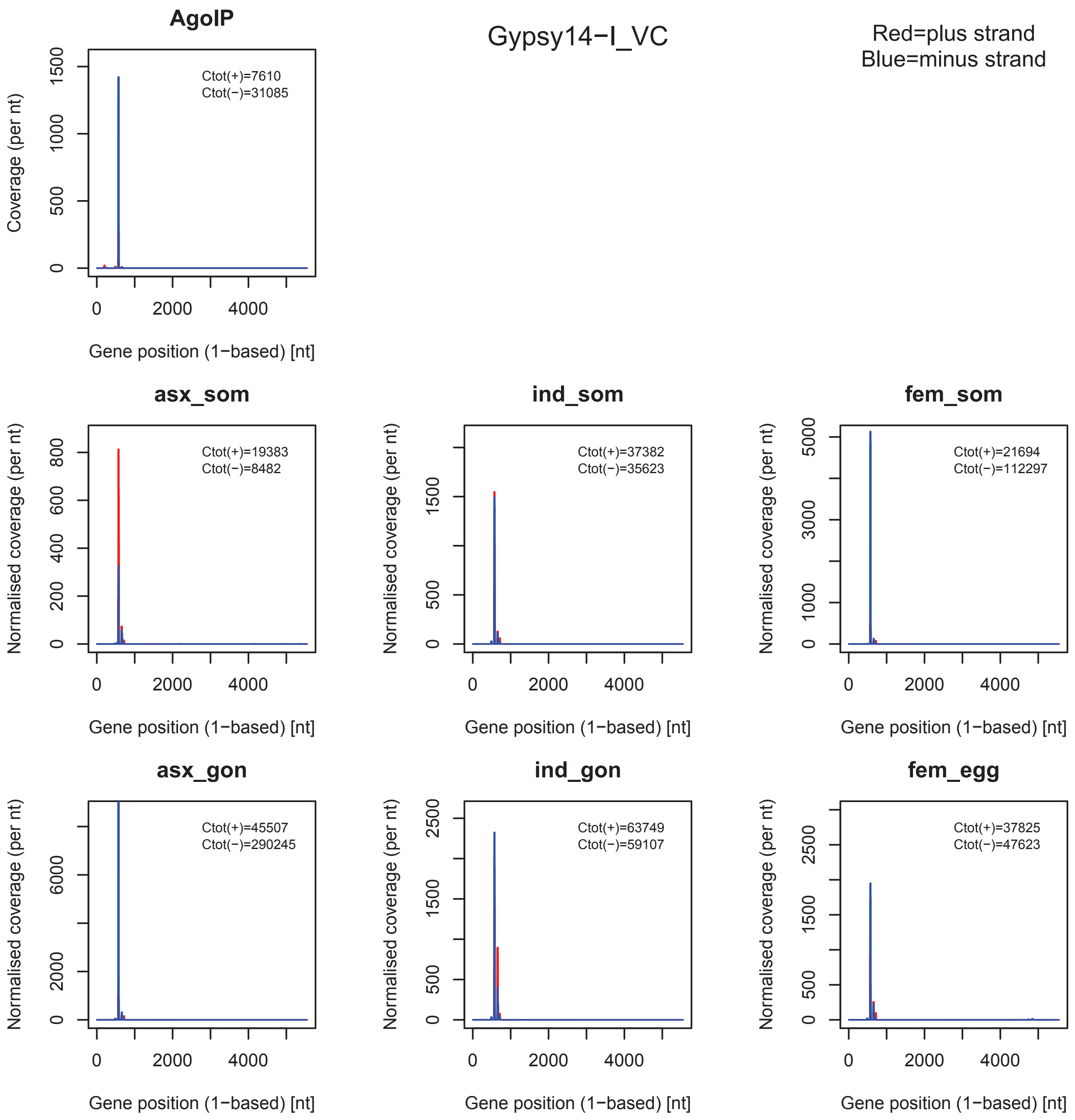
Gypsy14−I_VC
Red=plus strand
Blue=minus strand
AgoIP
Ctot(+)=7610
Ctot(−)=31085
Coverage (per nt)
asx_som
Ctot(+)=19383
Ctot(−)=8482
ind_som
Ctot(+)=37382
Ctot(−)=35623
fem_som
Ctot(+)=21694
Ctot(−)=112297
asx_gon
Ctot(+)=45507
Ctot(−)=290245
ind_gon
Ctot(+)=63749
Ctot(−)=59107
fem_egg
Ctot(+)=37825
Ctot(−)=47623
Normalised coverage (per nt)
Gene position (1−based) [nt]

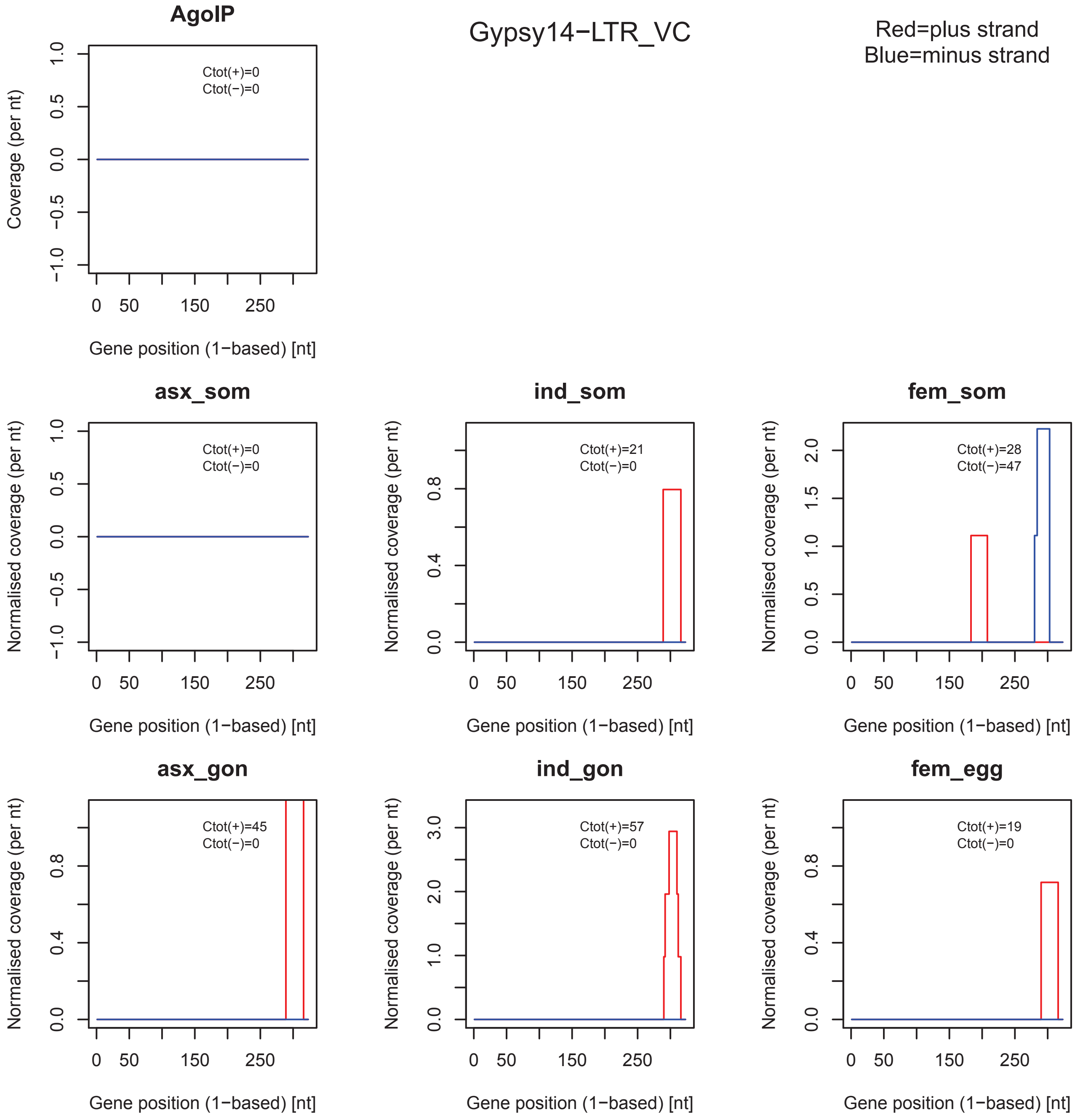

Gypsy14−LTR_VC
Red=plus strand
Blue=minus strand
AgoIP
Ctot(+)=0
Ctot(−)=0
Coverage (per nt)
Gene position (1−based) [nt]
asx_som
Ctot(+)=0
Ctot(−)=0
ind_som
Ctot(+)=21
Ctot(−)=0
fem_som
Ctot(+)=28
Ctot(−)=47
asx_gon
Ctot(+)=45
Ctot(−)=0
ind_gon
Ctot(+)=57
Ctot(−)=0
fem_egg
Ctot(+)=19
Ctot(−)=0
Normalised coverage (per nt)
Gene position (1−based) [nt]

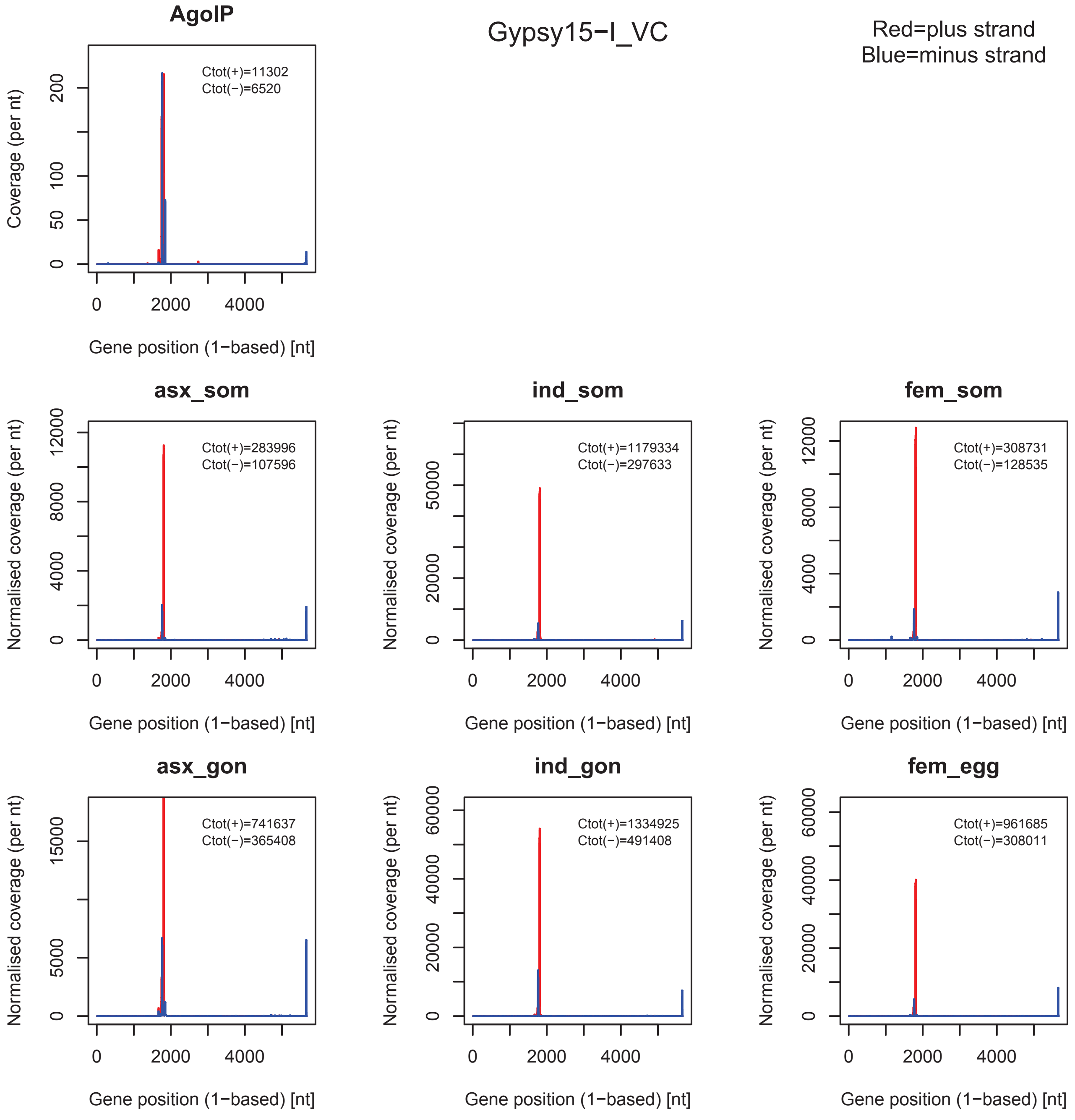

Gypsy15−I_VC
Red=plus strand
Blue=minus strand
AgoIP
Ctot(+)=11302
Ctot(−)=6520
Coverage (per nt)
0
50
100
200
0
2000
4000
Gene position (1−based) [nt]
asx_som
Ctot(+)=283996
Ctot(−)=107596
Normalised coverage (per nt)
0
4000
8000
12000
Gene position (1−based) [nt]
ind_som
Ctot(+)=1179334
Ctot(−)=297633
0
20000
50000
Normalised coverage (per nt)
Gene position (1−based) [nt]
fem_som
Ctot(+)=308731
Ctot(−)=128535
0
4000
8000
12000
Normalised coverage (per nt)
Gene position (1−based) [nt]
asx_gon
Ctot(+)=741637
Ctot(−)=365408
0
5000
15000
Normalised coverage (per nt)
Gene position (1−based) [nt]
ind_gon
Ctot(+)=1334925
Ctot(−)=491408
0
20000
40000
60000
Normalised coverage (per nt)
Gene position (1−based) [nt]
fem_egg
Ctot(+)=961685
Ctot(−)=308011
0
20000
40000
60000
Normalised coverage (per nt)
Gene position (1−based) [nt]

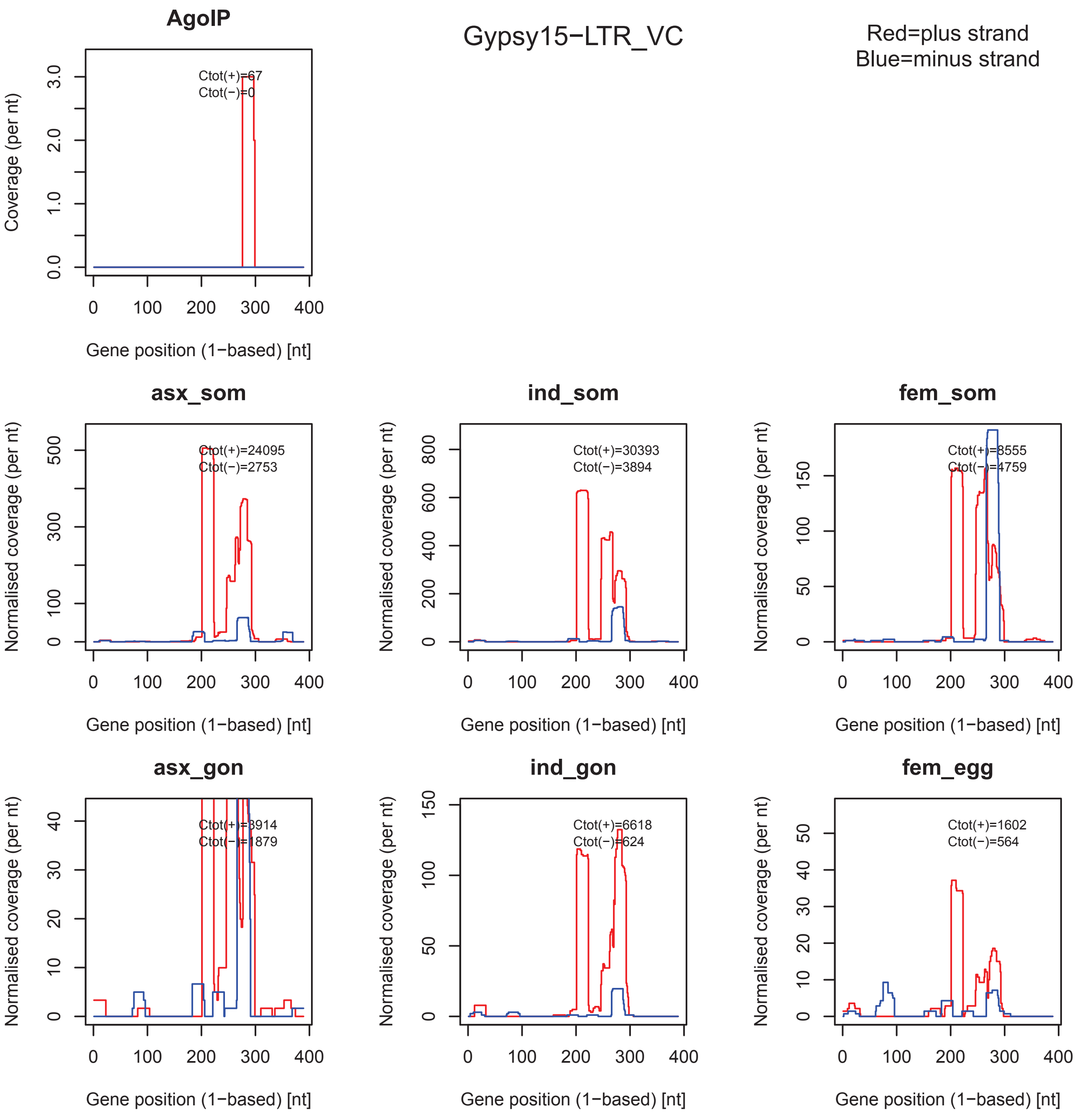
Gypsy15−LTR_VC
Red=plus strand
Blue=minus strand
AgoIP
Ctot(+)=67
Ctot(−)=0
Coverage (per nt)
asx_som
Ctot(+)=24095
Ctot(−)=2753
ind_som
Ctot(+)=30393
Ctot(−)=3894
fem_som
Ctot(+)=8555
Ctot(−)=4759
asx_gon
Ctot(+)=3914
Ctot(−)=1879
ind_gon
Ctot(+)=6618
Ctot(−)=624
fem_egg
Ctot(+)=1602
Ctot(−)=564
Normalised coverage (per nt)
Gene position (1−based) [nt]

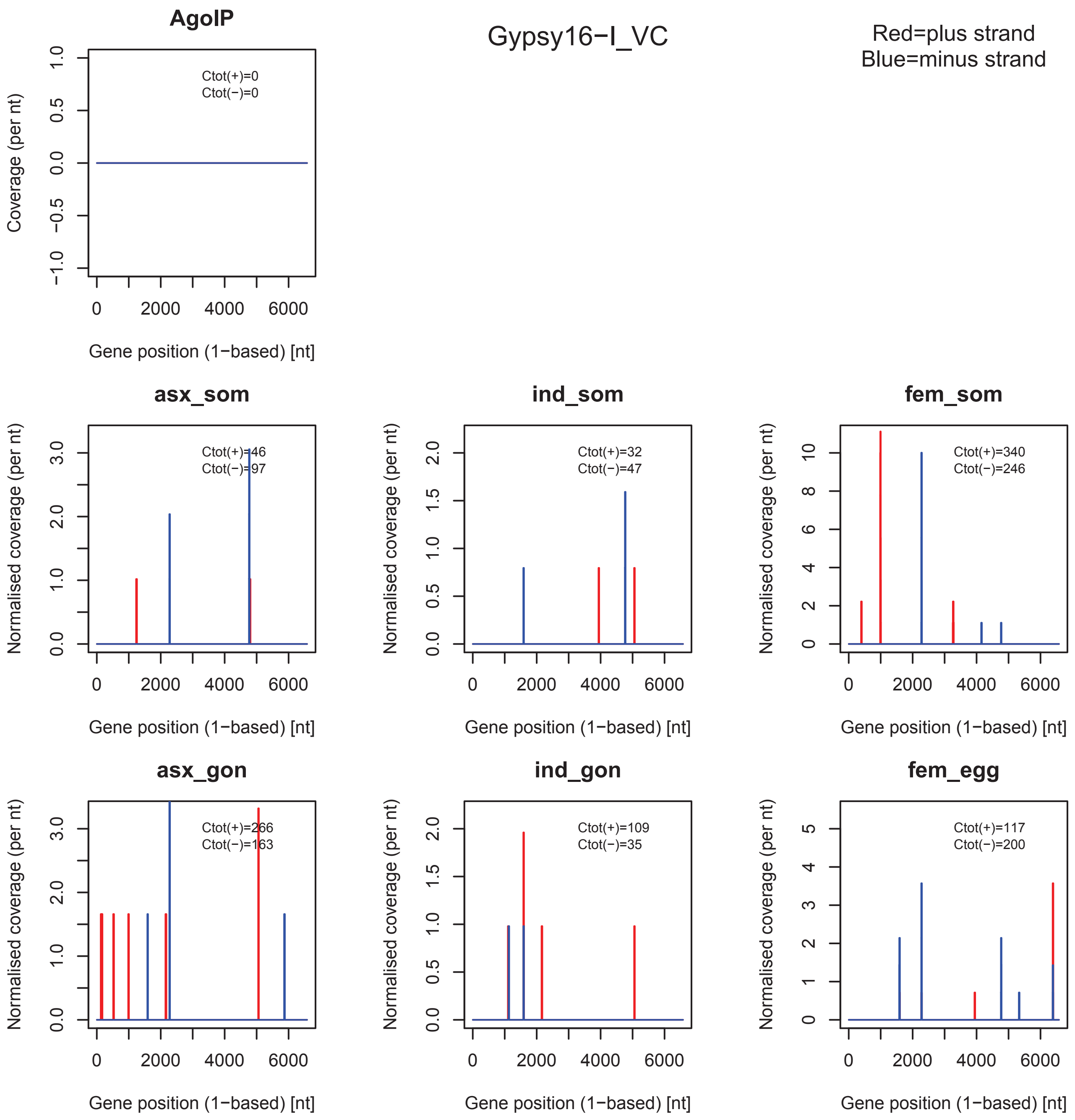

AgoIP
Gypsy16−I_VC
Red=plus strand
Blue=minus strand
Ctot(+)=0
Ctot(−)=0
Coverage (per nt)
Gene position (1−based) [nt]
asx_som
Ctot(+)=46
Ctot(−)=97
ind_som
Ctot(+)=32
Ctot(−)=47
fem_som
Ctot(+)=340
Ctot(−)=246
asx_gon
Ctot(+)=266
Ctot(−)=163
ind_gon
Ctot(+)=109
Ctot(−)=35
fem_egg
Ctot(+)=117
Ctot(−)=200
Normalised coverage (per nt)
Gene position (1−based) [nt]

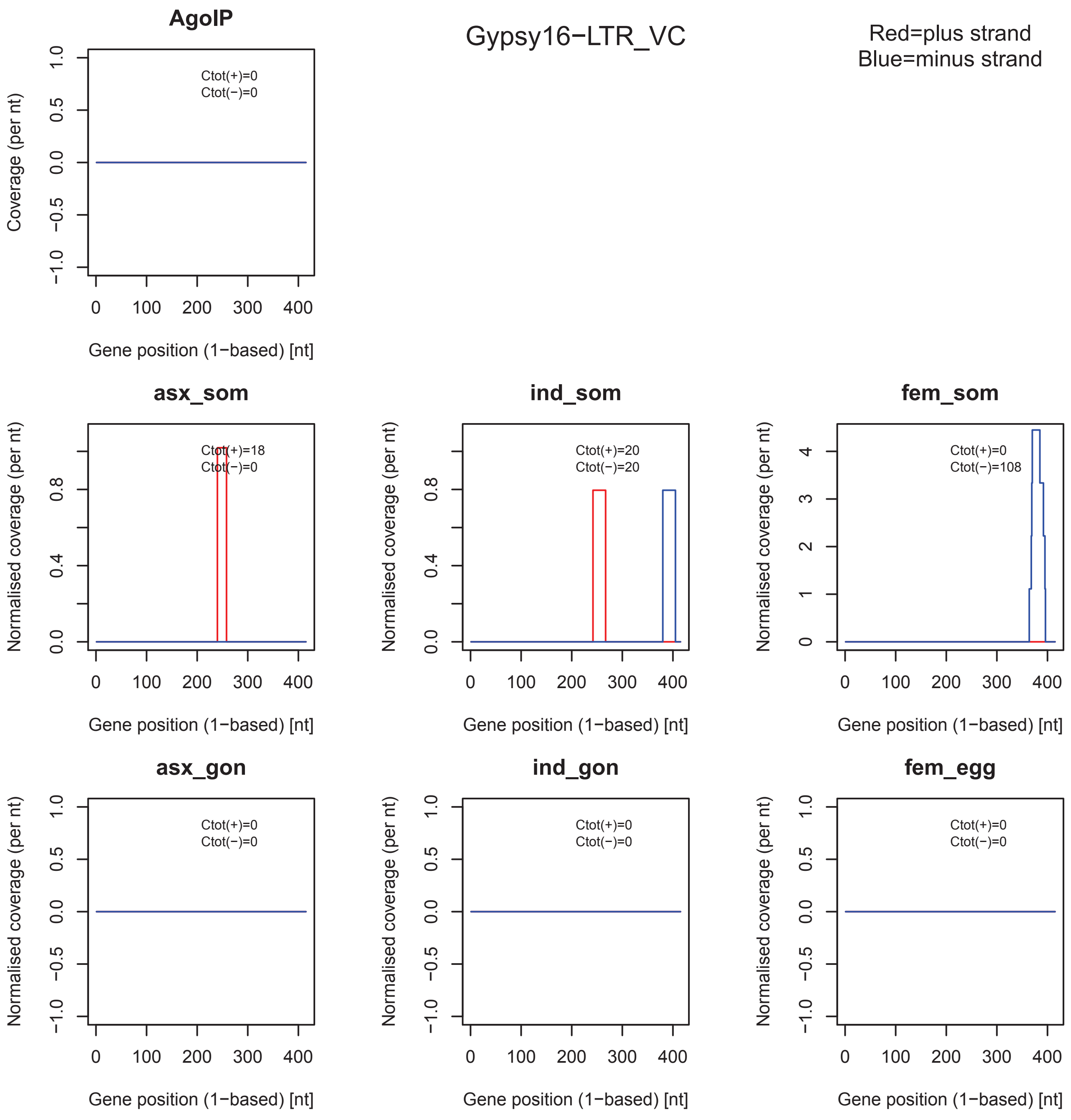

Gypsy16−LTR_VC
Red=plus strand
Blue=minus strand
AgoIP
Ctot(+)=0
Ctot(−)=0
Coverage (per nt)
Gene position (1−based) [nt]
asx_som
Ctot(+)=18
Ctot(−)=0
ind_som
Ctot(+)=20
Ctot(−)=20
fem_som
Ctot(+)=0
Ctot(−)=108
asx_gon
Ctot(+)=0
Ctot(−)=0
ind_gon
Ctot(+)=0
Ctot(−)=0
fem_egg
Ctot(+)=0
Ctot(−)=0
Normalised coverage (per nt)
Gene position (1−based) [nt]

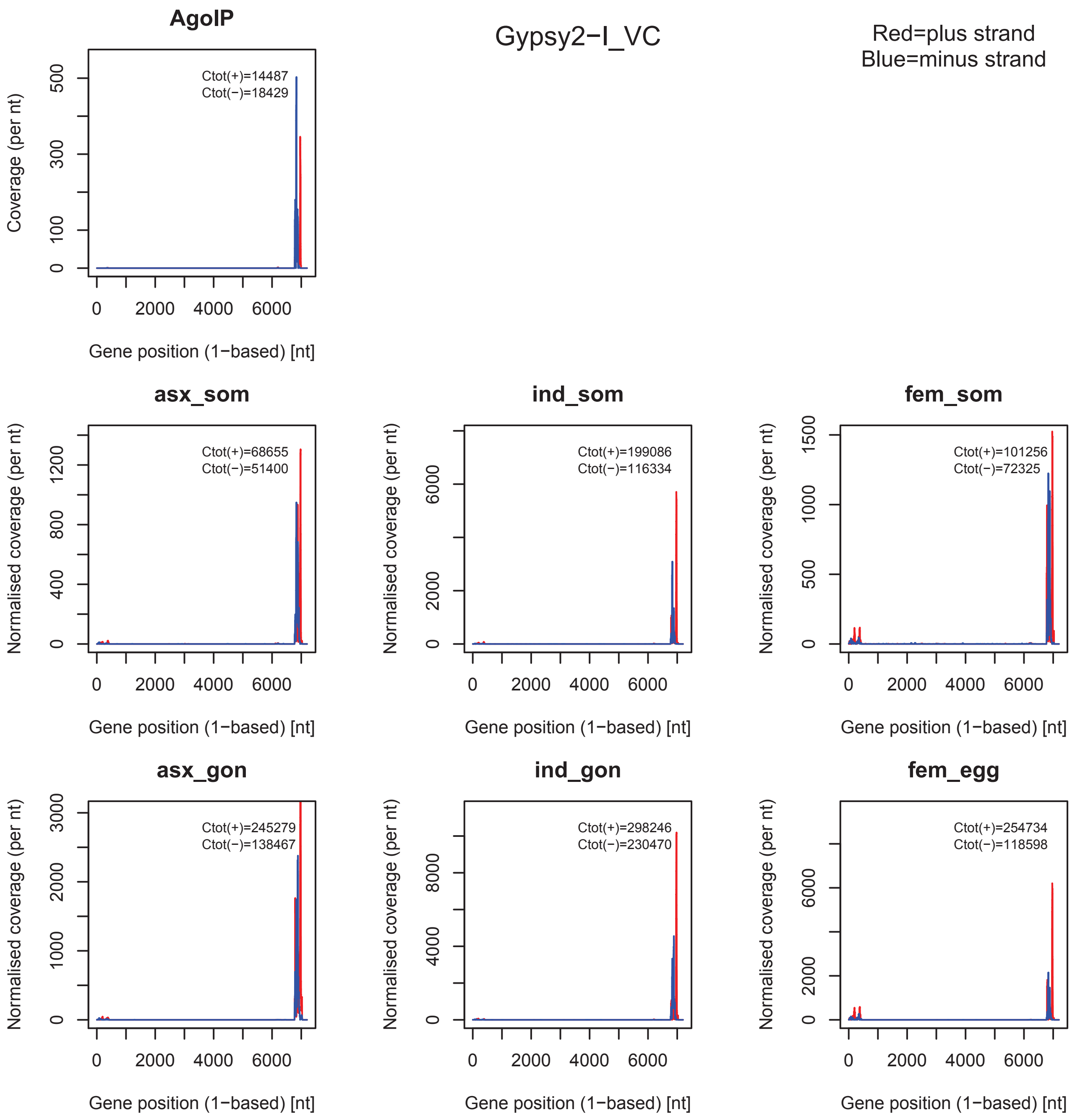
Gypsy2−I_VC
Red=plus strand
Blue=minus strand
AgoIP
Ctot(+)=14487
Ctot(−)=18429
Coverage (per nt)
0
100
300
500
0
2000
4000
6000
Gene position (1−based) [nt]
asx_som
Ctot(+)=68655
Ctot(−)=51400
Normalised coverage (per nt)
0
400
800
1200
ind_som
Ctot(+)=199086
Ctot(−)=116334
0
2000
6000
fem_som
Ctot(+)=101256
Ctot(−)=72325
0
500
1000
1500
asx_gon
Ctot(+)=245279
Ctot(−)=138467
0
1000
2000
3000
ind_gon
Ctot(+)=298246
Ctot(−)=230470
0
4000
8000
fem_egg
Ctot(+)=254734
Ctot(−)=118598
0
2000
6000

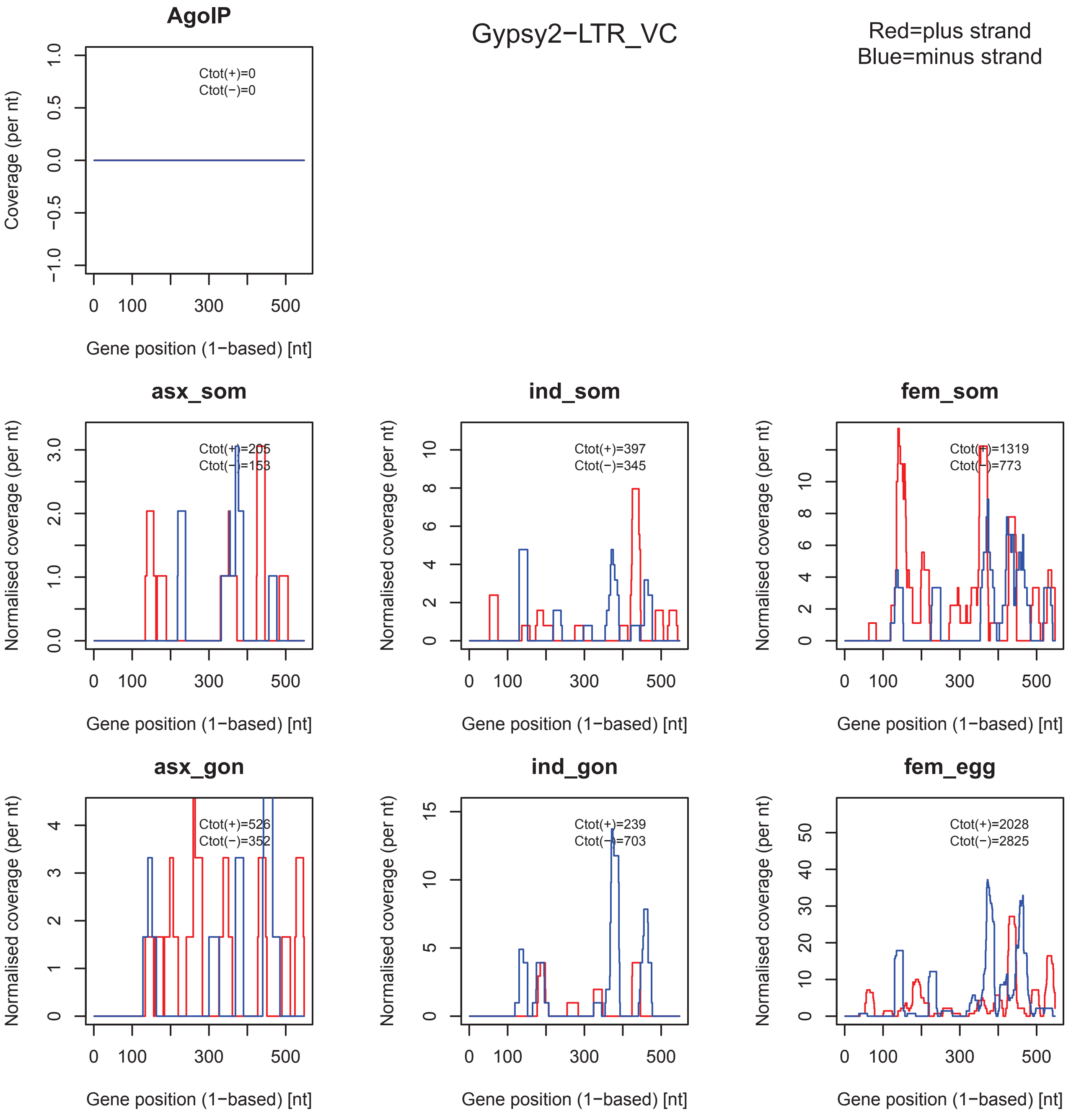

AgoIP
Gypsy2−LTR_VC
Red=plus strand
Blue=minus strand
Ctot(+)=0
Ctot(−)=0
Coverage (per nt)
Gene position (1−based) [nt]
asx_som
Ctot(+)=205
Ctot(−)=153
ind_som
Ctot(+)=397
Ctot(−)=345
fem_som
Ctot(+)=1319
Ctot(−)=773
asx_gon
Ctot(+)=526
Ctot(−)=352
ind_gon
Ctot(+)=239
Ctot(−)=703
fem_egg
Ctot(+)=2028
Ctot(−)=2825
Normalised coverage (per nt)
Gene position (1−based) [nt]

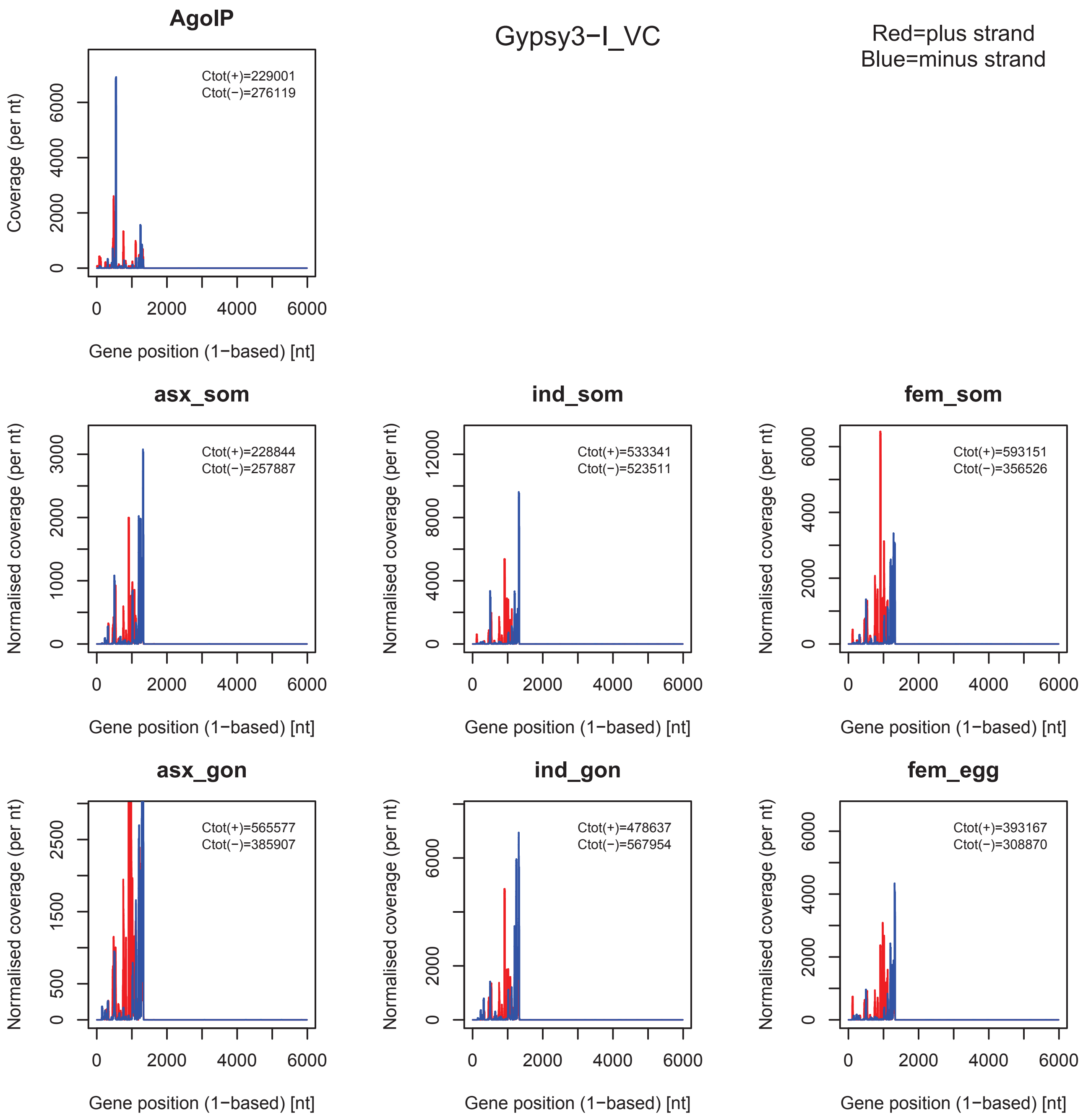
AgoIP
Gypsy3−I_VC
Red=plus strand
Blue=minus strand
Ctot(+)=229001
Ctot(−)=276119
Coverage (per nt)
Gene position (1−based) [nt]
asx_som
Ctot(+)=228844
Ctot(−)=257887
ind_som
Ctot(+)=533341
Ctot(−)=523511
fem_som
Ctot(+)=593151
Ctot(−)=356526
asx_gon
Ctot(+)=565577
Ctot(−)=385907
ind_gon
Ctot(+)=478637
Ctot(−)=567954
fem_egg
Ctot(+)=393167
Ctot(−)=308870
Normalised coverage (per nt)
Gene position (1−based) [nt]

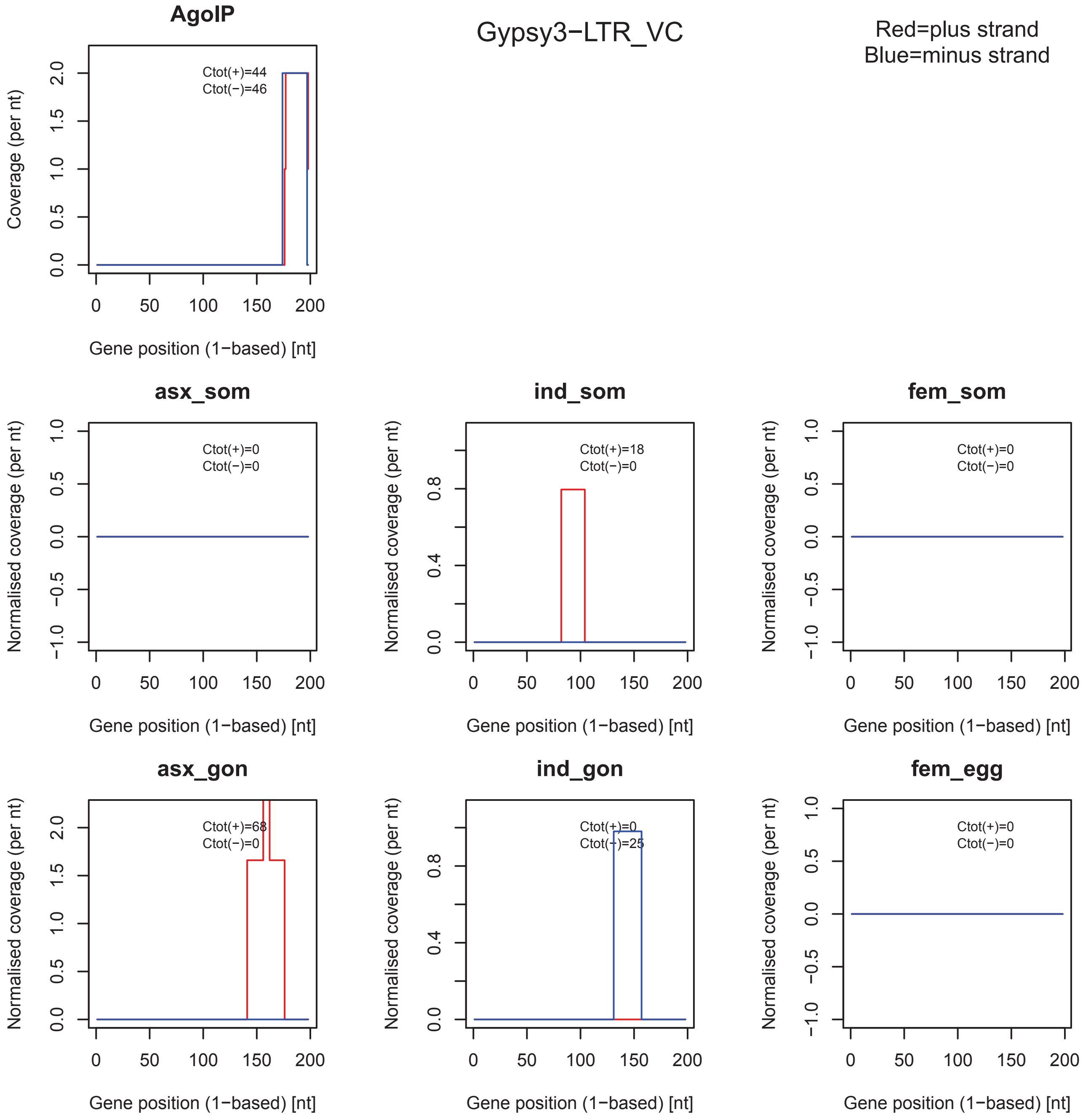
Gypsy3−LTR_VC
Red=plus strand
Blue=minus strand
AgoIP
Ctot(+)=44
Ctot(−)=46
Coverage (per nt)
asx_som
Ctot(+)=0
Ctot(−)=0
ind_som
Ctot(+)=18
Ctot(−)=0
fem_som
Ctot(+)=0
Ctot(−)=0
asx_gon
Ctot(+)=68
Ctot(−)=0
ind_gon
Ctot(+)=0
Ctot(−)=25
fem_egg
Ctot(+)=0
Ctot(−)=0
Normalised coverage (per nt)
Gene position (1−based) [nt]

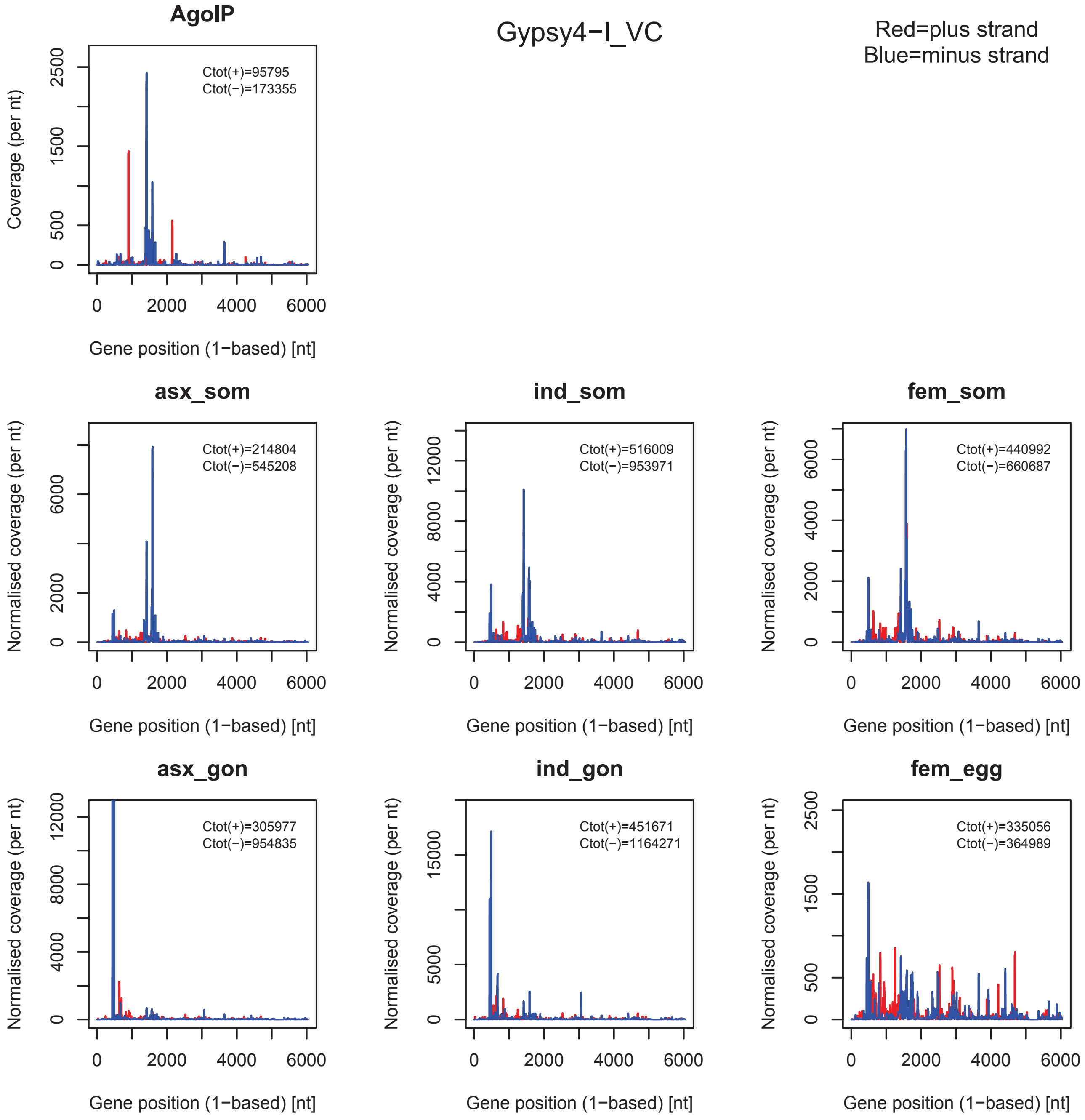

AgoIP
Gypsy4−I_VC
Red=plus strand
Blue=minus strand
Ctot(+)=95795
Ctot(−)=173355
Coverage (per nt)
Gene position (1−based) [nt]
asx_som
Ctot(+)=214804
Ctot(−)=545208
ind_som
Ctot(+)=516009
Ctot(−)=953971
fem_som
Ctot(+)=440992
Ctot(−)=660687
asx_gon
Ctot(+)=305977
Ctot(−)=954835
ind_gon
Ctot(+)=451671
Ctot(−)=1164271
fem_egg
Ctot(+)=335056
Ctot(−)=364989
Normalised coverage (per nt)
Gene position (1−based) [nt]

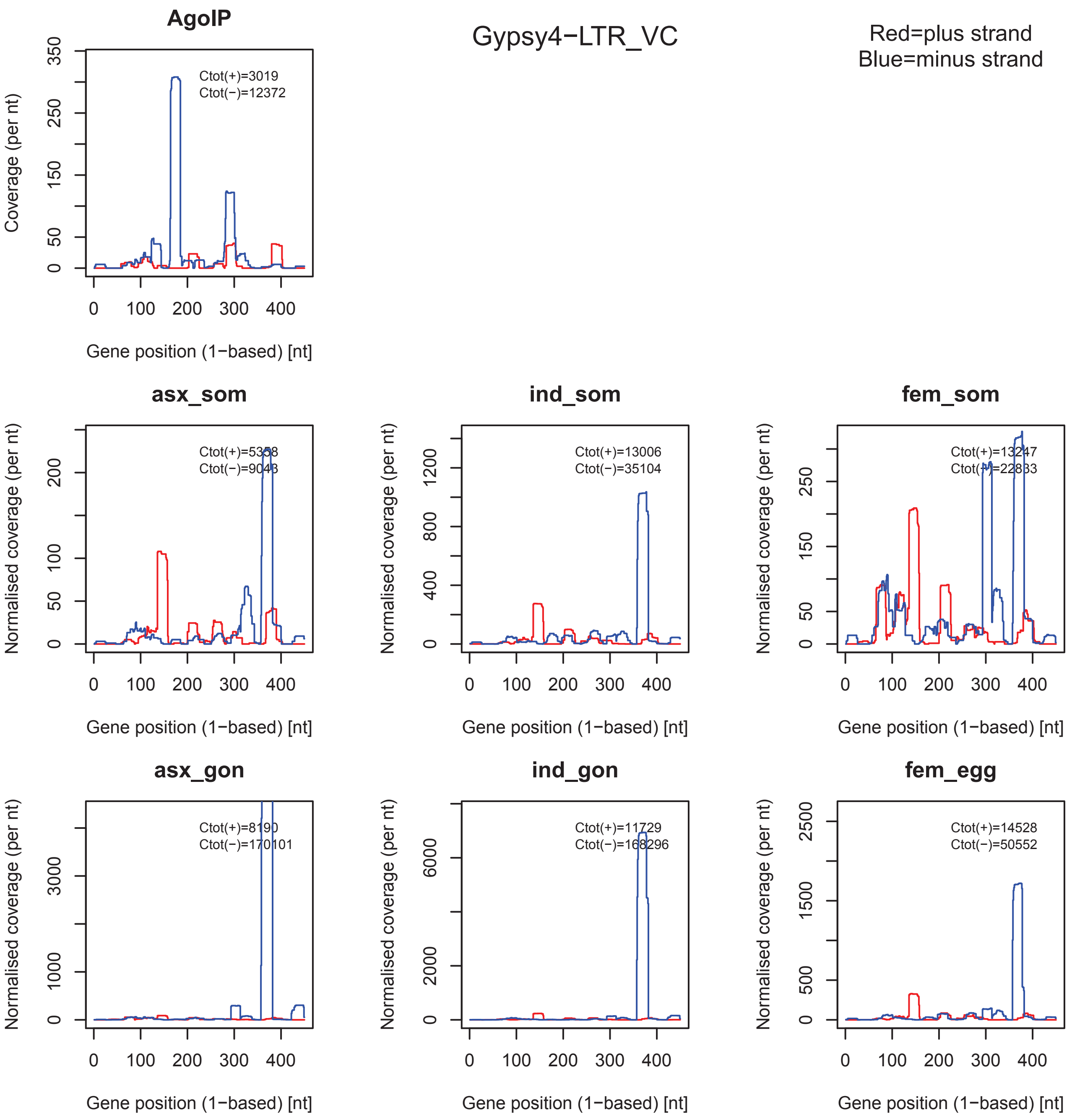
Gypsy4−LTR_VC
Red=plus strand
Blue=minus strand
AgoIP
Ctot(+)=3019
Ctot(−)=12372
Coverage (per nt)
asx_som
ind_som
Ctot(+)=13006
Ctot(−)=35104
fem_som
asx_gon
ind_gon
Ctot(+)=11729
fem_egg
Ctot(+)=14528
Ctot(−)=50552
Normalised coverage (per nt)
Gene position (1−based) [nt]

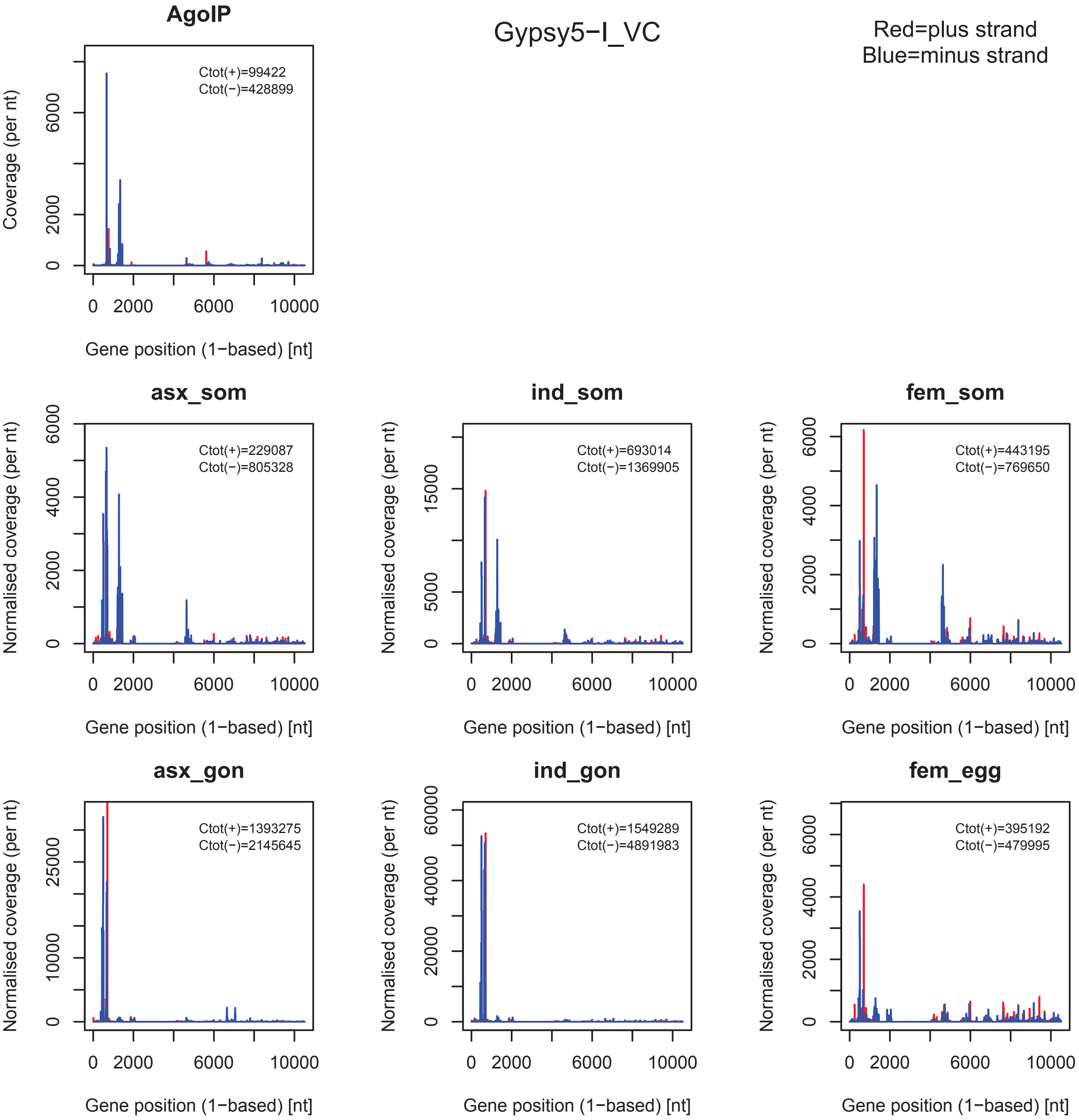

Gypsy5−I_VC
Red=plus strand
Blue=minus strand
AgoIP
Ctot(+)=99422
Ctot(−)=428899
Coverage (per nt)
Gene position (1−based) [nt]
asx_som
Ctot(+)=229087
Ctot(−)=805328
ind_som
Ctot(+)=693014
Ctot(−)=1369905
fem_som
Ctot(+)=443195
Ctot(−)=769650
asx_gon
Ctot(+)=1393275
Ctot(−)=2145645
ind_gon
Ctot(+)=1549289
Ctot(−)=4891983
fem_egg
Ctot(+)=395192
Ctot(−)=479995
Normalised coverage (per nt)
Gene position (1−based) [nt]

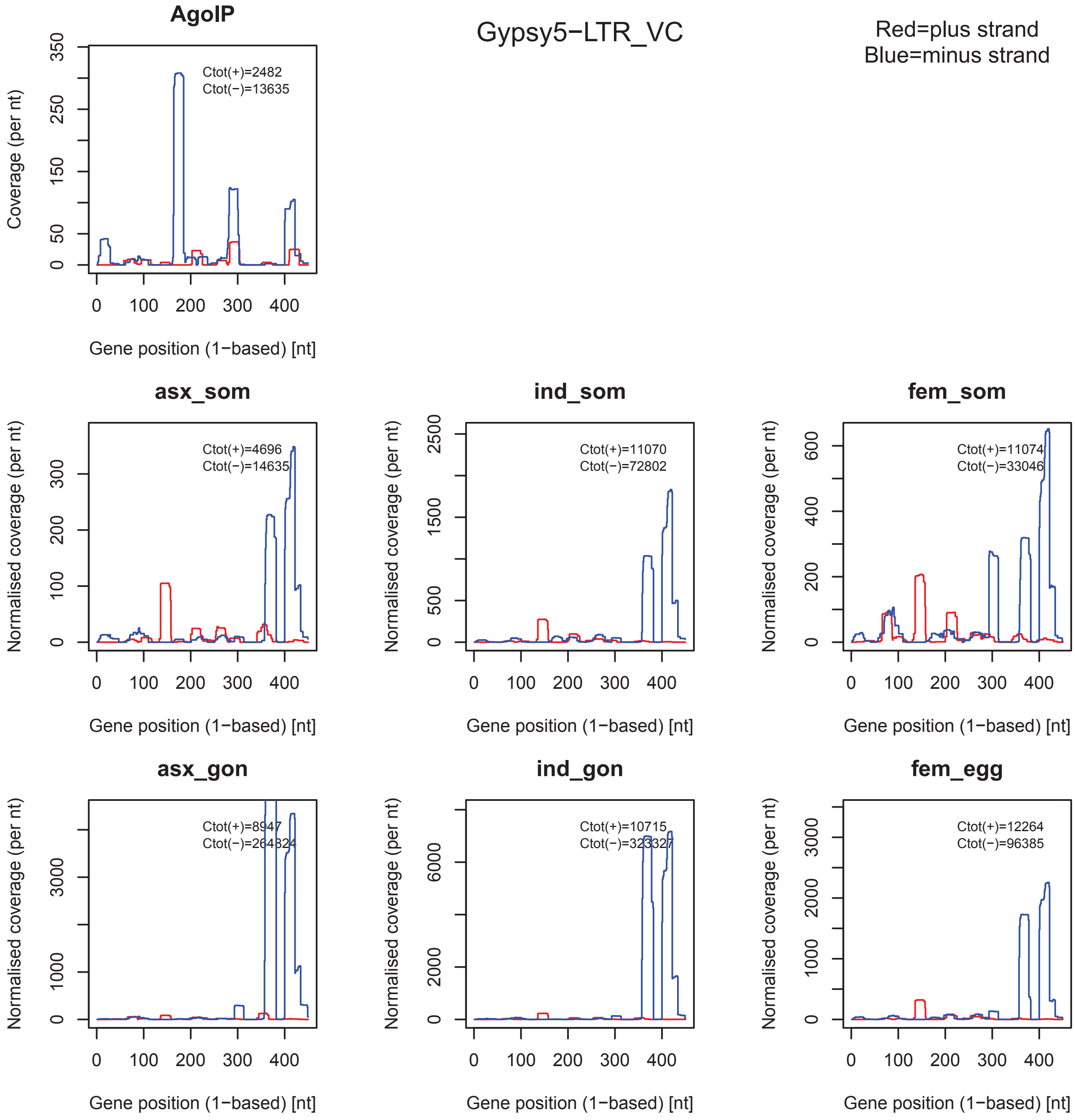
Gypsy5−LTR_VC
Red=plus strand
Blue=minus strand
AgoIP
Ctot(+)=2482
Ctot(−)=13635
Coverage (per nt)
Gene position (1−based) [nt]
asx_som
Ctot(+)=4696
Ctot(−)=14635
ind_som
Ctot(+)=11070
Ctot(−)=72802
fem_som
Ctot(+)=11074
Ctot(−)=33046
asx_gon
Ctot(+)=8947
Ctot(−)=264824
ind_gon
Ctot(+)=10715
Ctot(−)=323327
fem_egg
Ctot(+)=12264
Ctot(−)=96385
Normalised coverage (per nt)
Gene position (1−based) [nt]

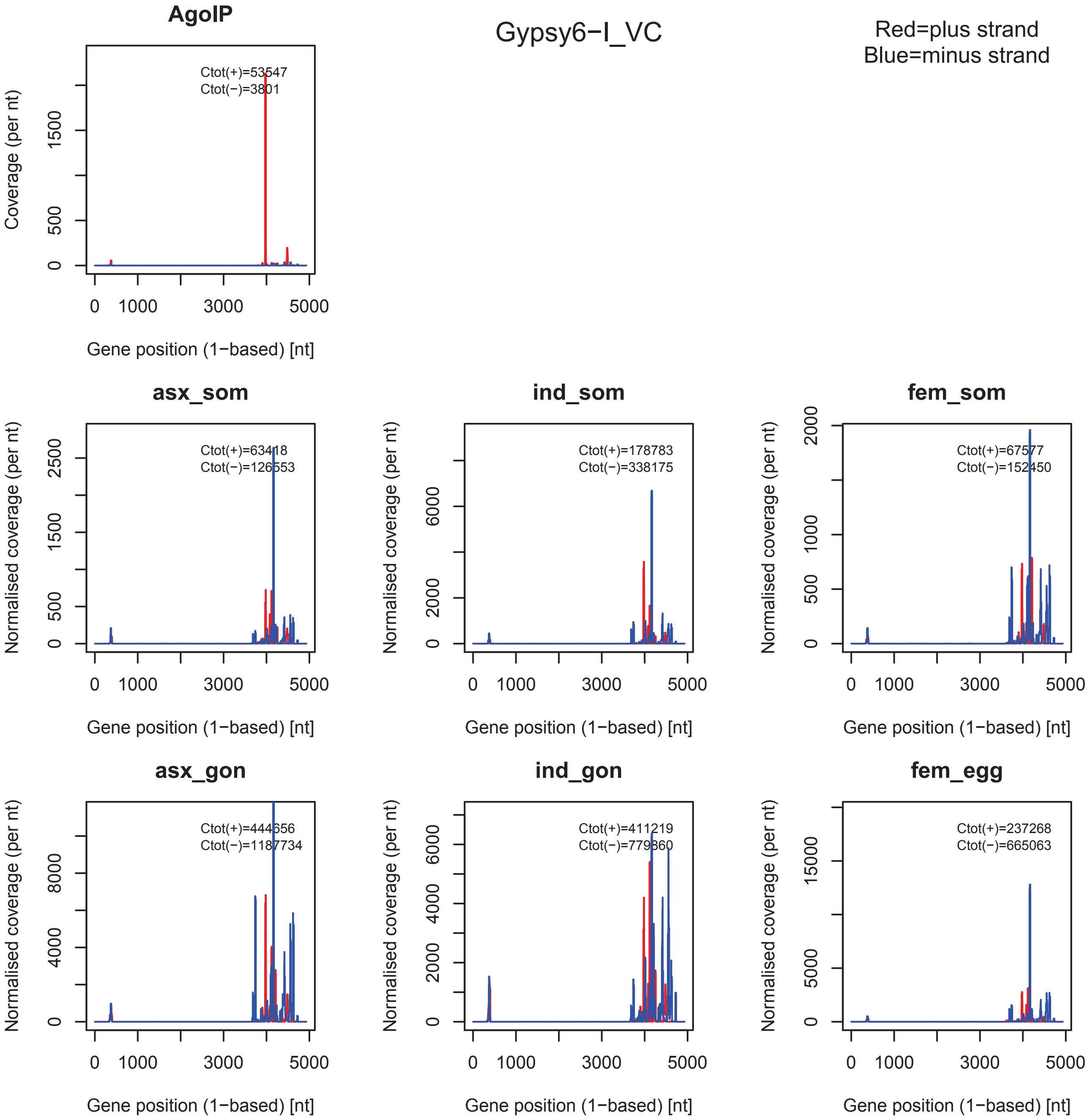
Gypsy6−I_VC
Red=plus strand
Blue=minus strand
AgoIP
Ctot(+)=53547
Ctot(−)=3801
Coverage (per nt)
Gene position (1−based) [nt]
asx_som
Ctot(+)=63418
Ctot(−)=126553
ind_som
Ctot(+)=178783
Ctot(−)=338175
fem_som
Ctot(+)=67577
Ctot(−)=152450
asx_gon
Ctot(+)=444656
Ctot(−)=1187734
ind_gon
Ctot(+)=411219
Ctot(−)=779860
fem_egg
Ctot(+)=237268
Ctot(−)=665063
Normalised coverage (per nt)
Gene position (1−based) [nt]

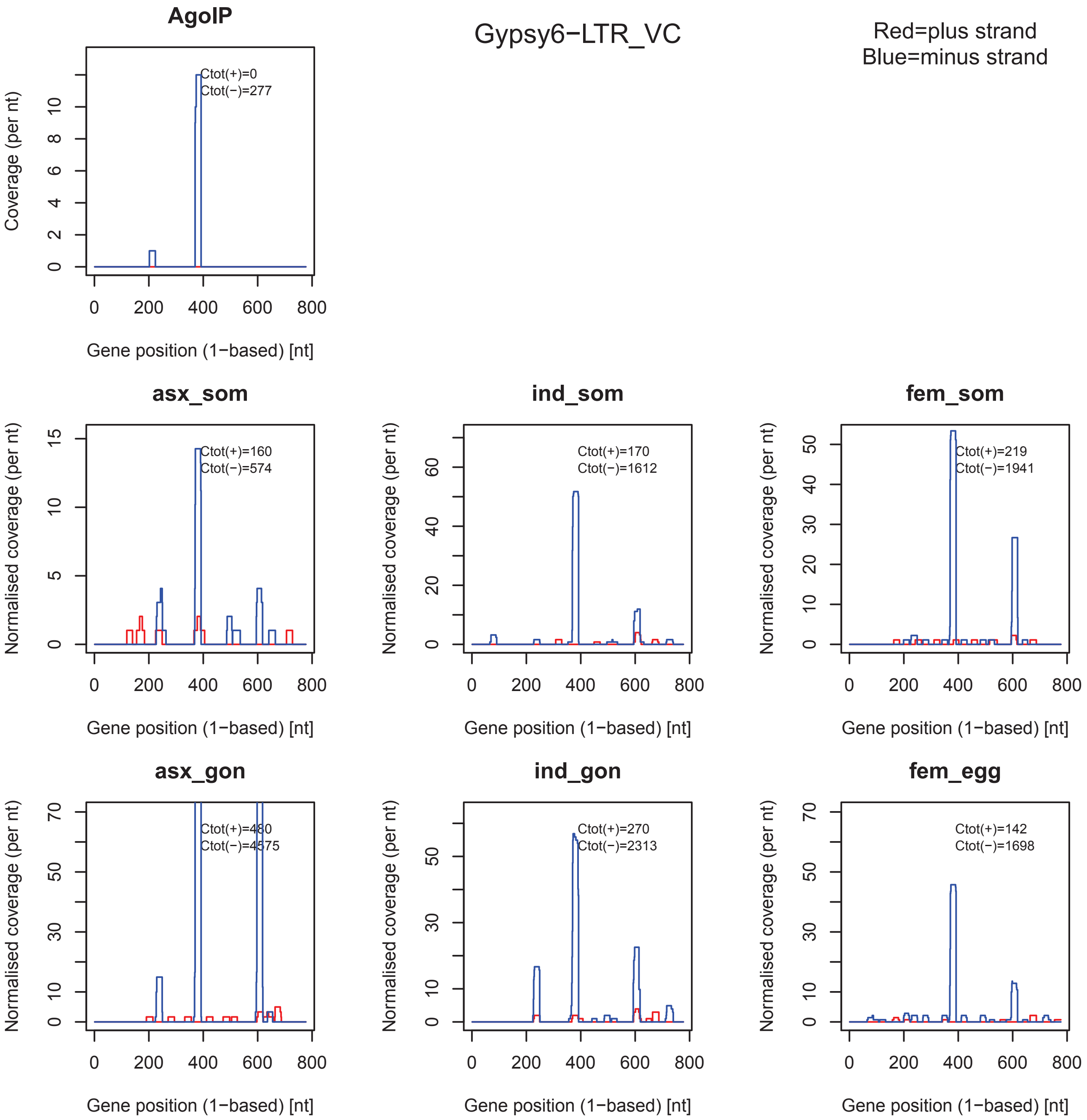

Gypsy6−LTR_VC
Red=plus strand
Blue=minus strand
AgoIP
Ctot(+)=0
Ctot(−)=277
Coverage (per nt)
Gene position (1−based) [nt]
asx_som
Ctot(+)=160
Ctot(−)=574
ind_som
Ctot(+)=170
Ctot(−)=1612
fem_som
Ctot(+)=219
Ctot(−)=1941
asx_gon
Ctot(+)=480
Ctot(−)=4575
ind_gon
Ctot(+)=270
Ctot(−)=2313
fem_egg
Ctot(+)=142
Ctot(−)=1698
Normalised coverage (per nt)
Gene position (1−based) [nt]

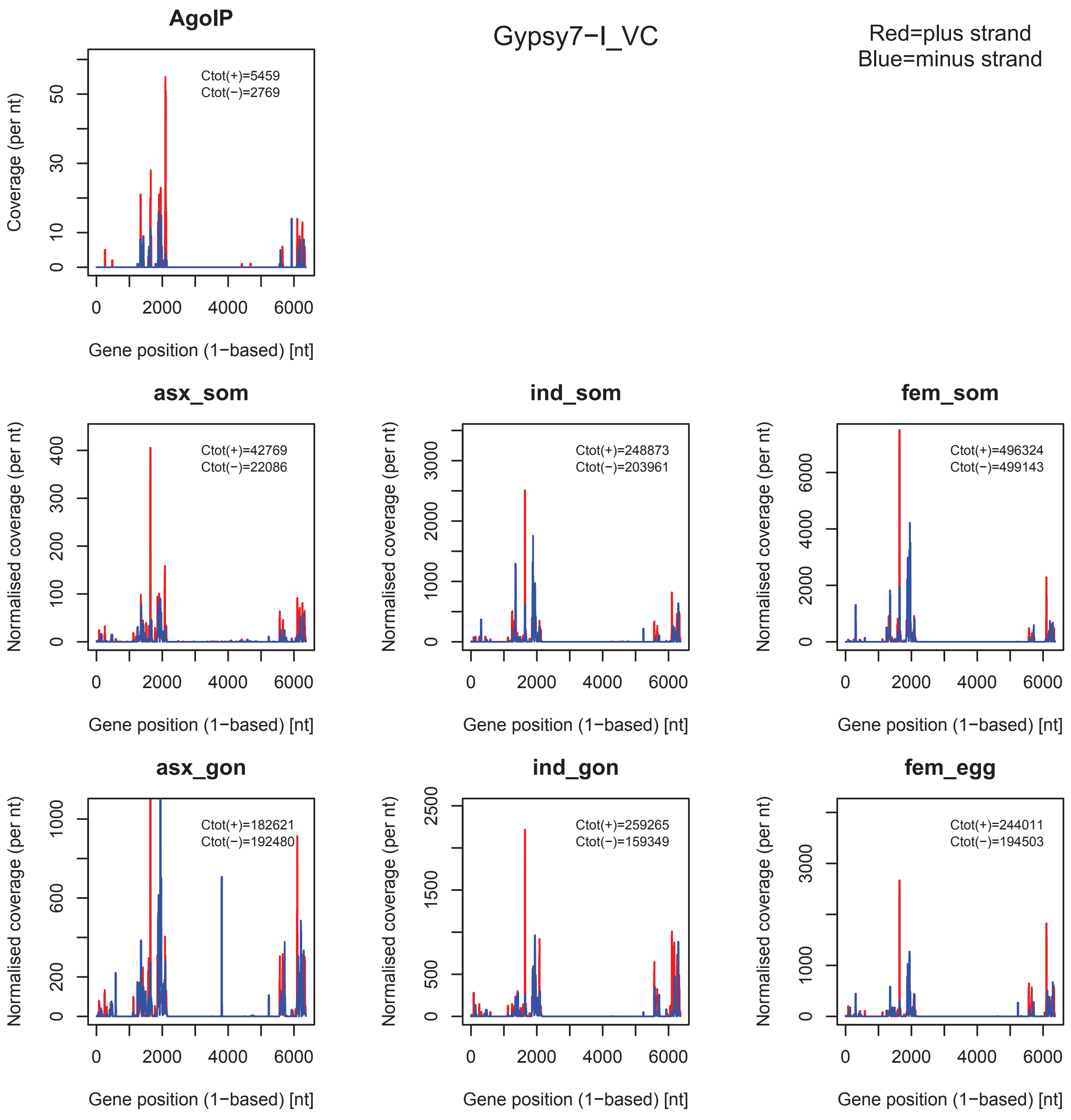

Gypsy7−I_VC
Red=plus strand
Blue=minus strand
AgoIP
Ctot(+)=5459
Ctot(−)=2769
Coverage (per nt)
Gene position (1−based) [nt]
asx_som
Ctot(+)=42769
Ctot(−)=22086
ind_som
Ctot(+)=248873
Ctot(−)=203961
fem_som
Ctot(+)=496324
Ctot(−)=499143
asx_gon
Ctot(+)=182621
Ctot(−)=192480
ind_gon
Ctot(+)=259265
Ctot(−)=159349
fem_egg
Ctot(+)=244011
Ctot(−)=194503
Normalised coverage (per nt)
Gene position (1−based) [nt]

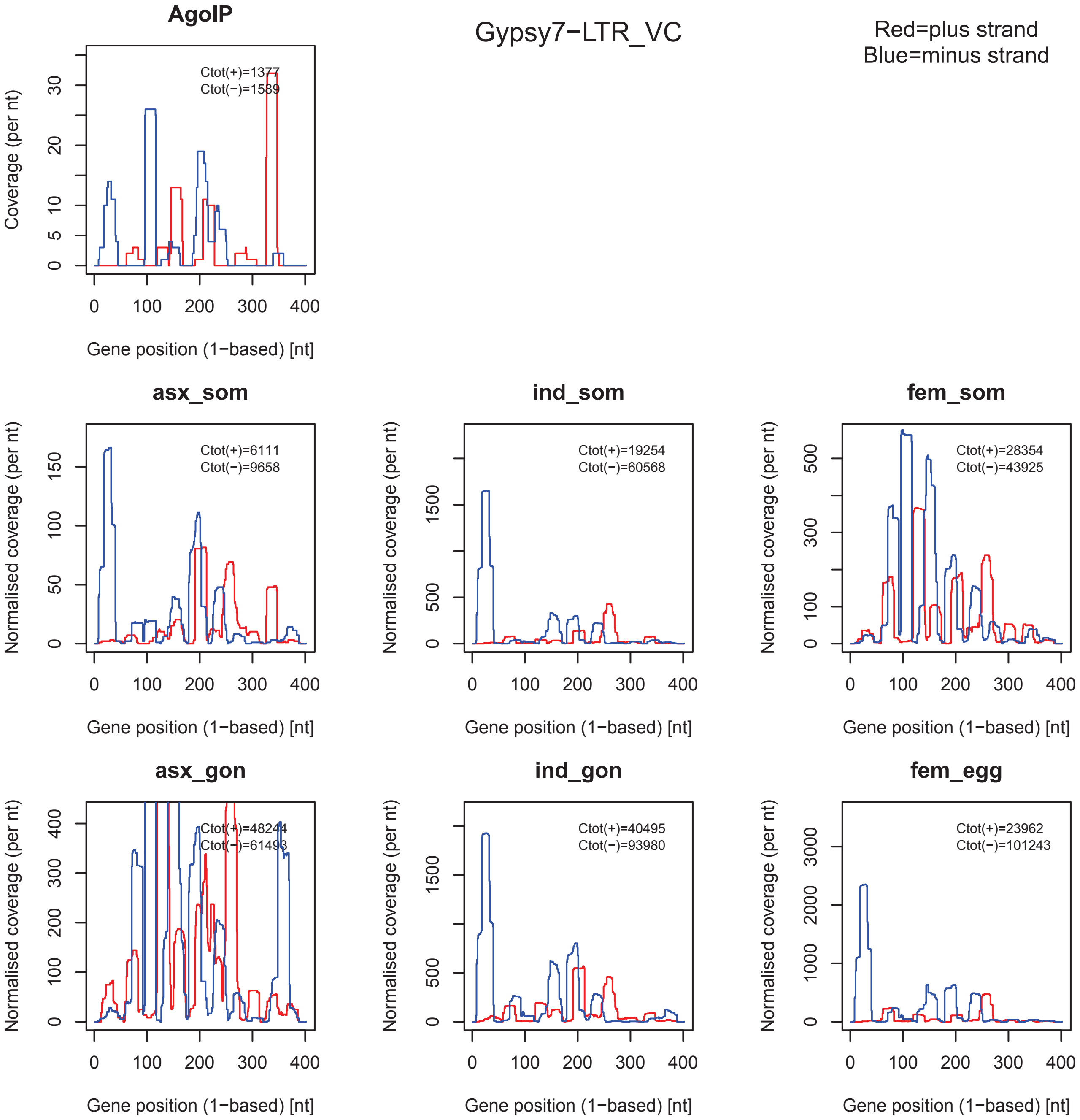

Gypsy7−LTR_VC
Red=plus strand
Blue=minus strand
AgoIP
Ctot(+)=1377
Ctot(−)=1589
Coverage (per nt)
asx_som
Ctot(+)=6111
Ctot(−)=9658
ind_som
Ctot(+)=19254
Ctot(−)=60568
fem_som
Ctot(+)=28354
Ctot(−)=43925
asx_gon
Ctot(+)=48244
Ctot(−)=61493
ind_gon
Ctot(+)=40495
Ctot(−)=93980
fem_egg
Ctot(+)=23962
Ctot(−)=101243
Normalised coverage (per nt)
Gene position (1−based) [nt]

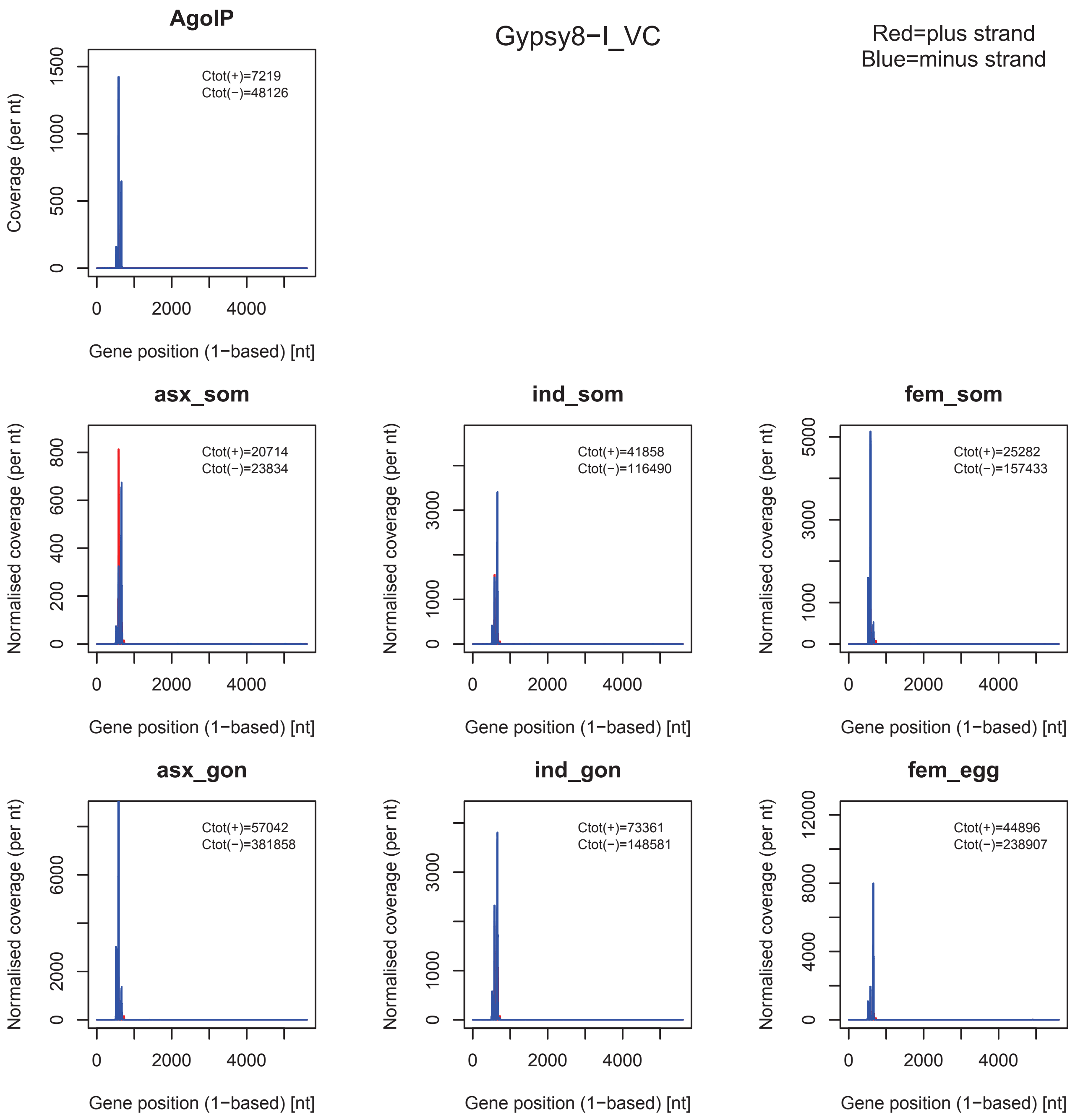
Gypsy8−I_VC
Red=plus strand
Blue=minus strand
AgoIP
Ctot(+)=7219
Ctot(−)=48126
Coverage (per nt)
0
500
1000
1500
0
2000
4000
Gene position (1−based) [nt]
asx_som
Ctot(+)=20714
Ctot(−)=23834
Normalised coverage (per nt)
0
200
400
600
800
ind_som
Ctot(+)=41858
Ctot(−)=116490
0
1000
3000
fem_som
Ctot(+)=25282
Ctot(−)=157433
0
1000
3000
5000
asx_gon
Ctot(+)=57042
Ctot(−)=381858
0
2000
6000
ind_gon
Ctot(+)=73361
Ctot(−)=148581
0
1000
3000
fem_egg
Ctot(+)=44896
Ctot(−)=238907
0
4000
8000
12000

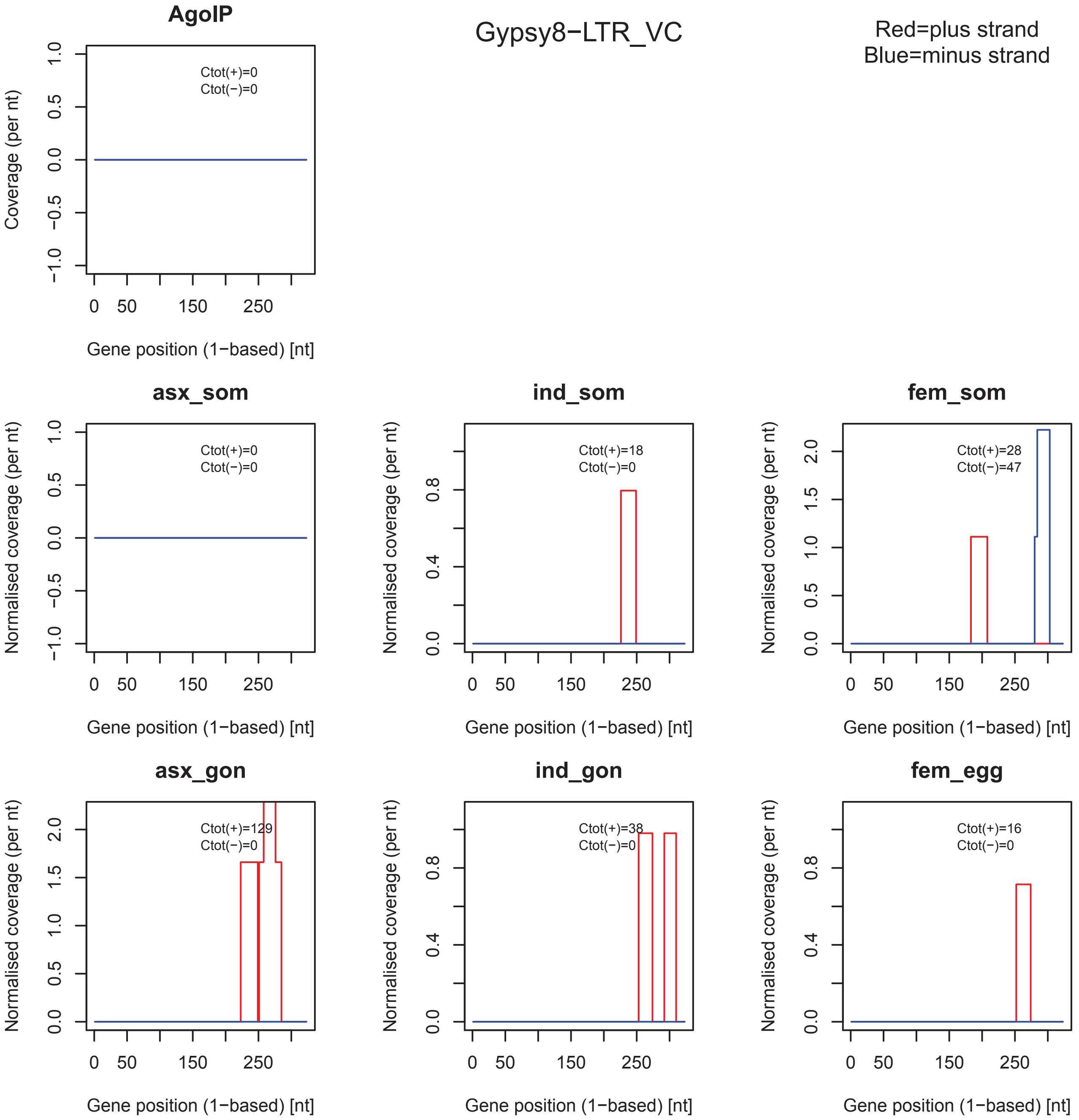
Gypsy8−LTR_VC
Red=plus strand
Blue=minus strand
AgoIP
Ctot(+)=0
Ctot(−)=0
Coverage (per nt)
asx_som
Ctot(+)=0
Ctot(−)=0
ind_som
Ctot(+)=18
Ctot(−)=0
fem_som
Ctot(+)=28
Ctot(−)=47
asx_gon
Ctot(+)=129
Ctot(−)=0
ind_gon
Ctot(+)=38
Ctot(−)=0
fem_egg
Ctot(+)=16
Ctot(−)=0
Normalised coverage (per nt)
Gene position (1−based) [nt]

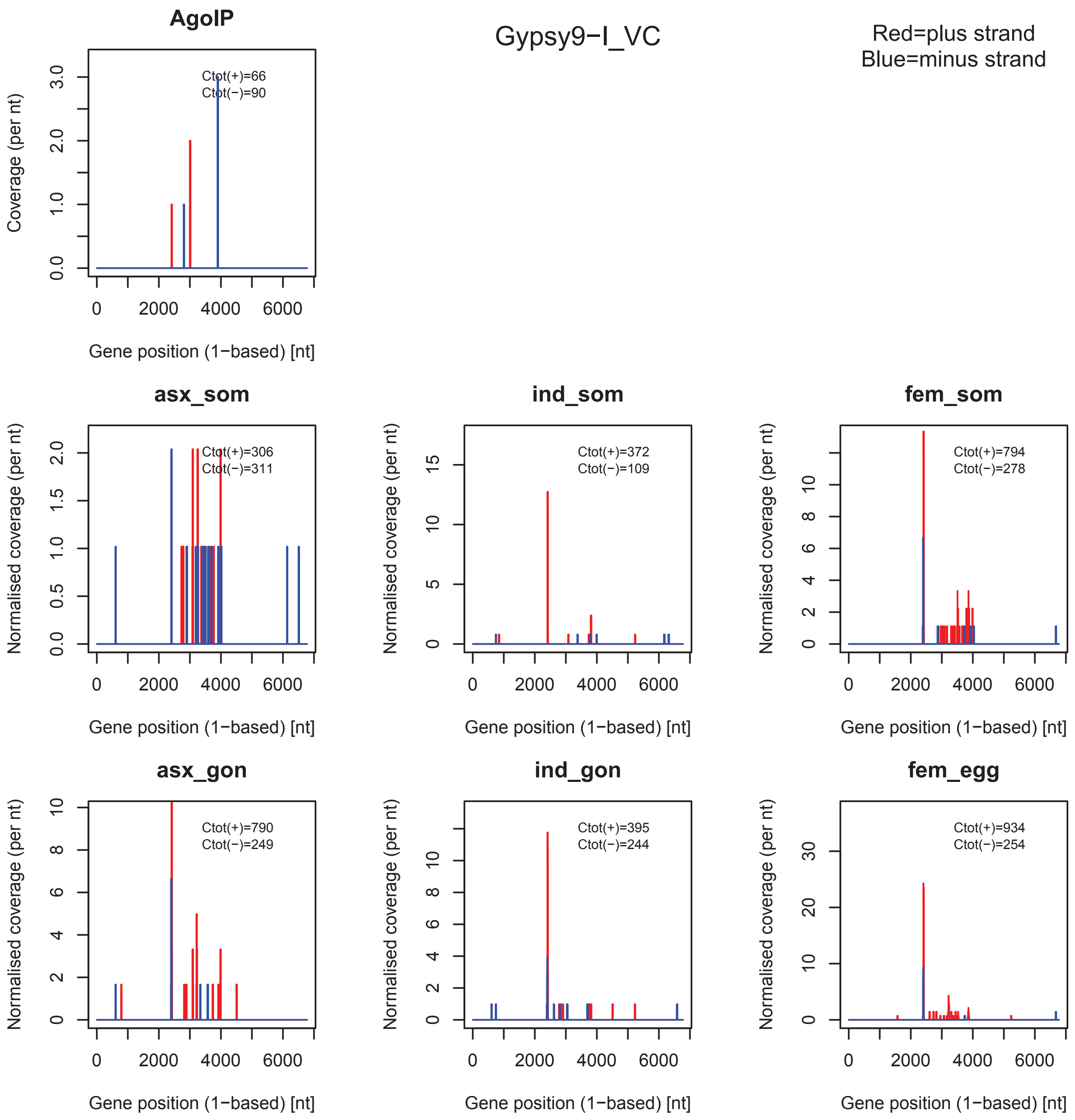
Gypsy9−I_VC
Red=plus strand
Blue=minus strand
AgoIP
Ctot(+)=66
Ctot(−)=90
Coverage (per nt)
asx_som
Ctot(+)=306
Ctot(−)=311
ind_som
Ctot(+)=372
Ctot(−)=109
fem_som
Ctot(+)=794
Ctot(−)=278
asx_gon
Ctot(+)=790
Ctot(−)=249
ind_gon
Ctot(+)=395
Ctot(−)=244
fem_egg
Ctot(+)=934
Ctot(−)=254
Normalised coverage (per nt)
Gene position (1−based) [nt]

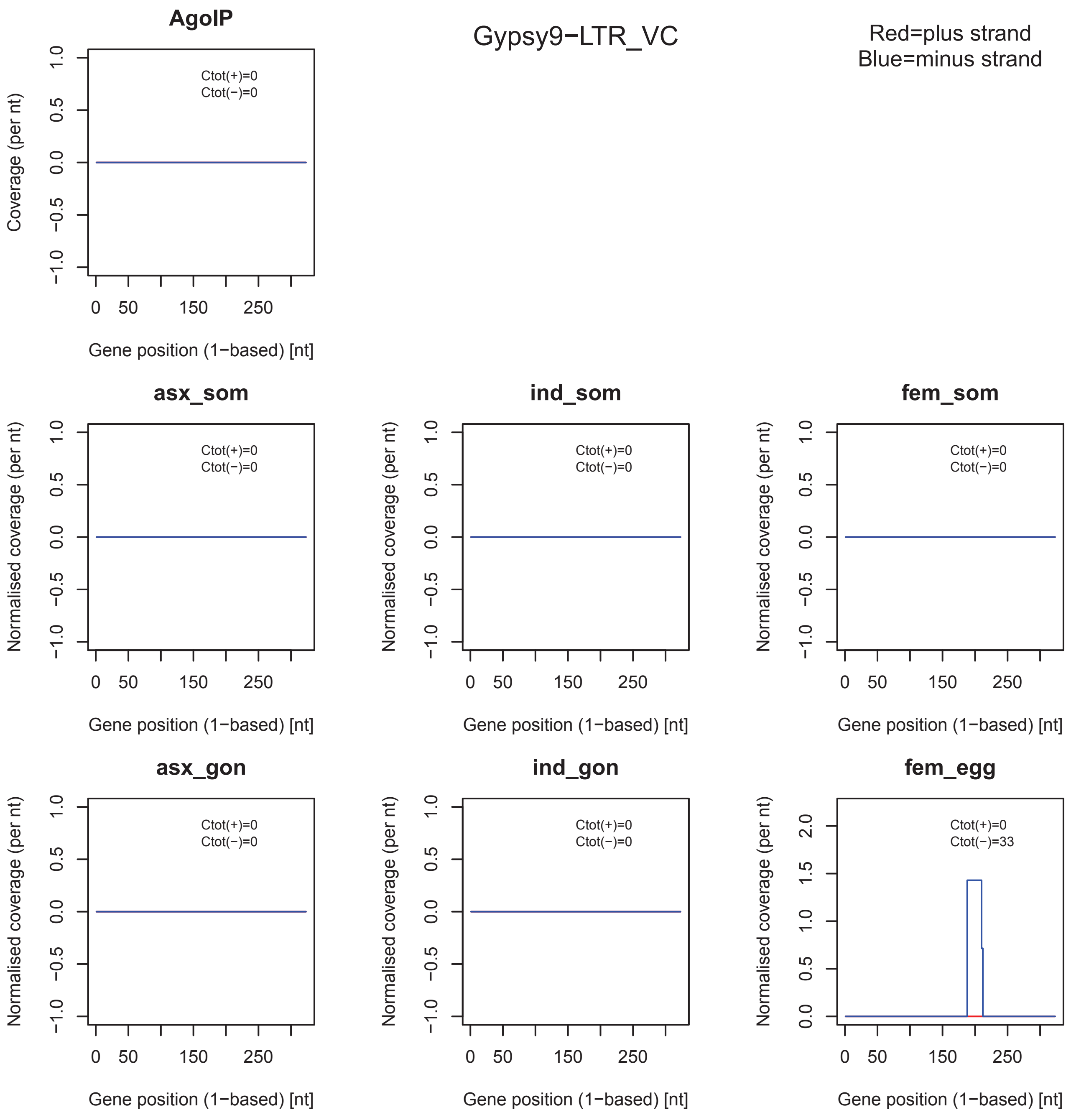
AgoIP
Gypsy9−LTR_VC
Red=plus strand
Blue=minus strand
Ctot(+)=0
Ctot(−)=0
Coverage (per nt)
Gene position (1−based) [nt]
asx_som
ind_som
fem_som
asx_gon
ind_gon
fem_egg
Ctot(+)=0
Ctot(−)=33
Normalised coverage (per nt)
0
50
150
250
−1.0
−0.5
0.0
0.5
1.0
1.5
2.0

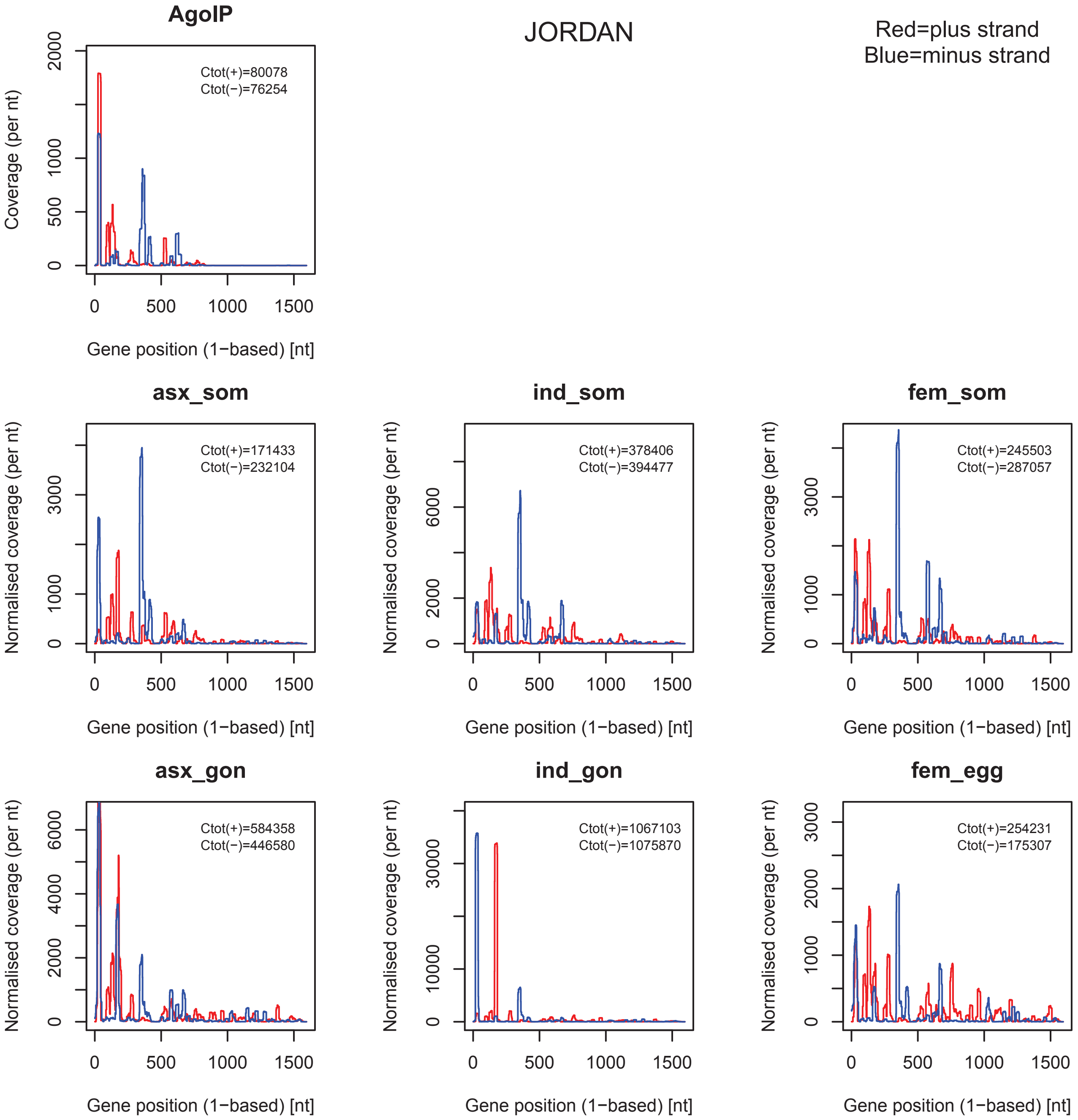

JORDAN
Red=plus strand
Blue=minus strand
AgoIP
Ctot(+)=80078
Ctot(−)=76254
Coverage (per nt)
asx_som
Ctot(+)=171433
Ctot(−)=232104
ind_som
Ctot(+)=378406
Ctot(−)=394477
fem_som
Ctot(+)=245503
Ctot(−)=287057
asx_gon
Ctot(+)=584358
Ctot(−)=446580
ind_gon
Ctot(+)=1067103
Ctot(−)=1075870
fem_egg
Ctot(+)=254231
Ctot(−)=175307
Normalised coverage (per nt)
Gene position (1−based) [nt]

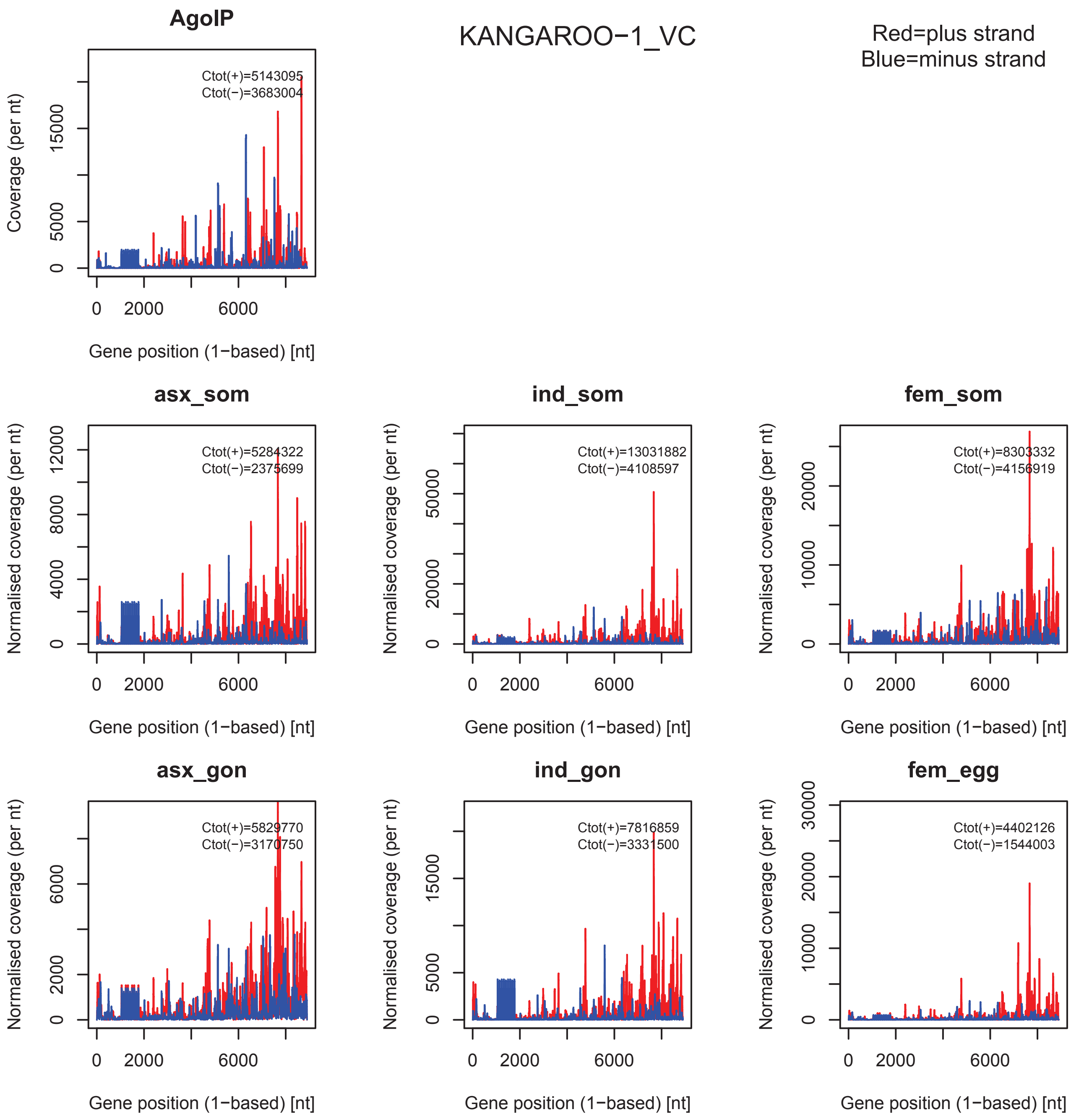

KANGAROO−1_VC
Red=plus strand
Blue=minus strand
AgoIP
Ctot(+)=5143095
Ctot(−)=3683004
Coverage (per nt)
Gene position (1−based) [nt]
asx_som
Ctot(+)=5284322
Ctot(−)=2375699
ind_som
Ctot(+)=13031882
Ctot(−)=4108597
fem_som
Ctot(+)=8303332
Ctot(−)=4156919
asx_gon
Ctot(+)=5829770
Ctot(−)=3170750
ind_gon
Ctot(+)=7816859
Ctot(−)=3331500
fem_egg
Ctot(+)=4402126
Ctot(−)=1544003
Normalised coverage (per nt)
Gene position (1−based) [nt]

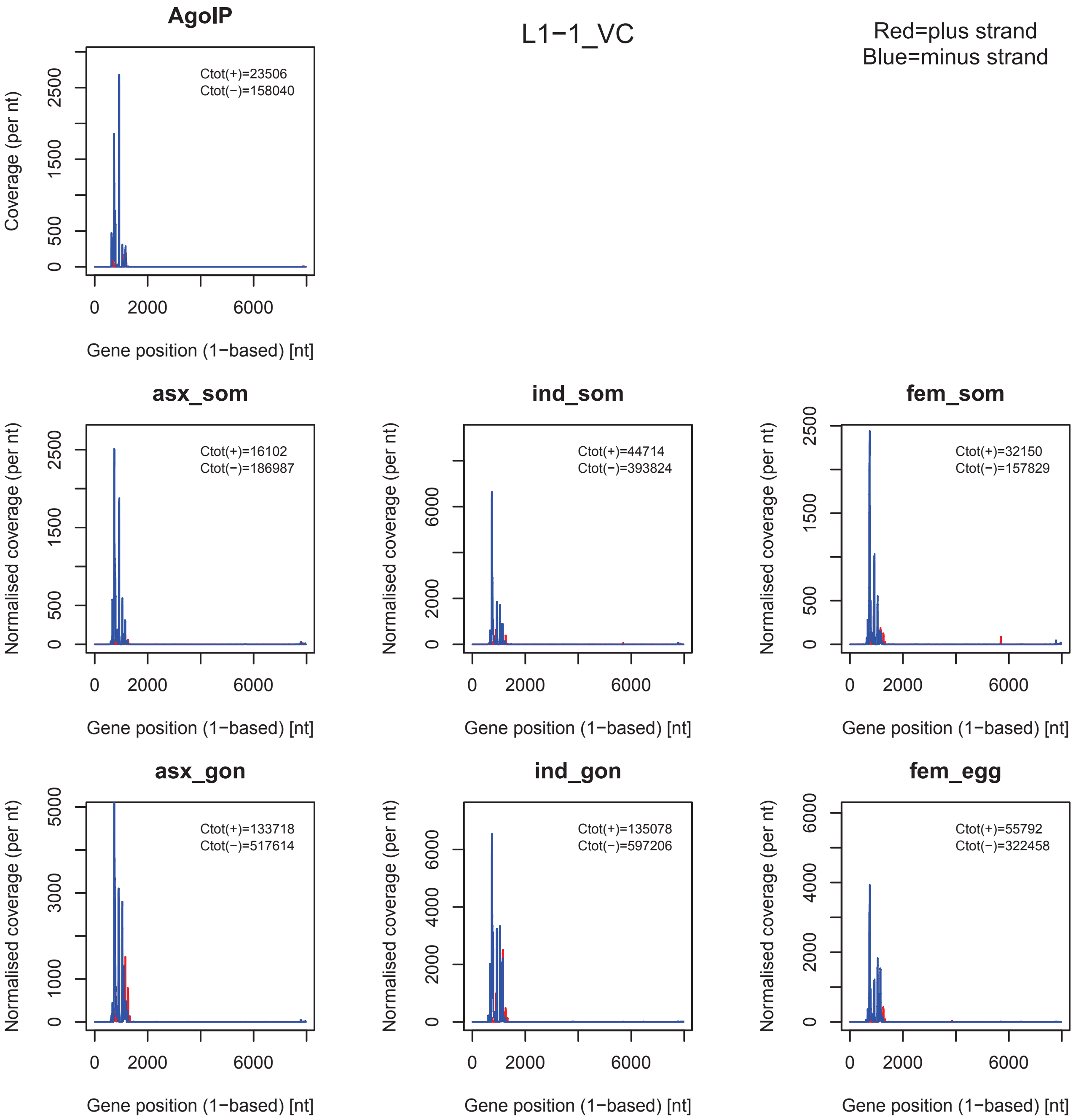
L1−1_VC
Red=plus strand
Blue=minus strand
AgoIP
Ctot(+)=23506
Ctot(−)=158040
Coverage (per nt)
Gene position (1−based) [nt]
asx_som
Ctot(+)=16102
Ctot(−)=186987
ind_som
Ctot(+)=44714
Ctot(−)=393824
fem_som
Ctot(+)=32150
Ctot(−)=157829
asx_gon
Ctot(+)=133718
Ctot(−)=517614
ind_gon
Ctot(+)=135078
Ctot(−)=597206
fem_egg
Ctot(+)=55792
Ctot(−)=322458
Normalised coverage (per nt)
Gene position (1−based) [nt]

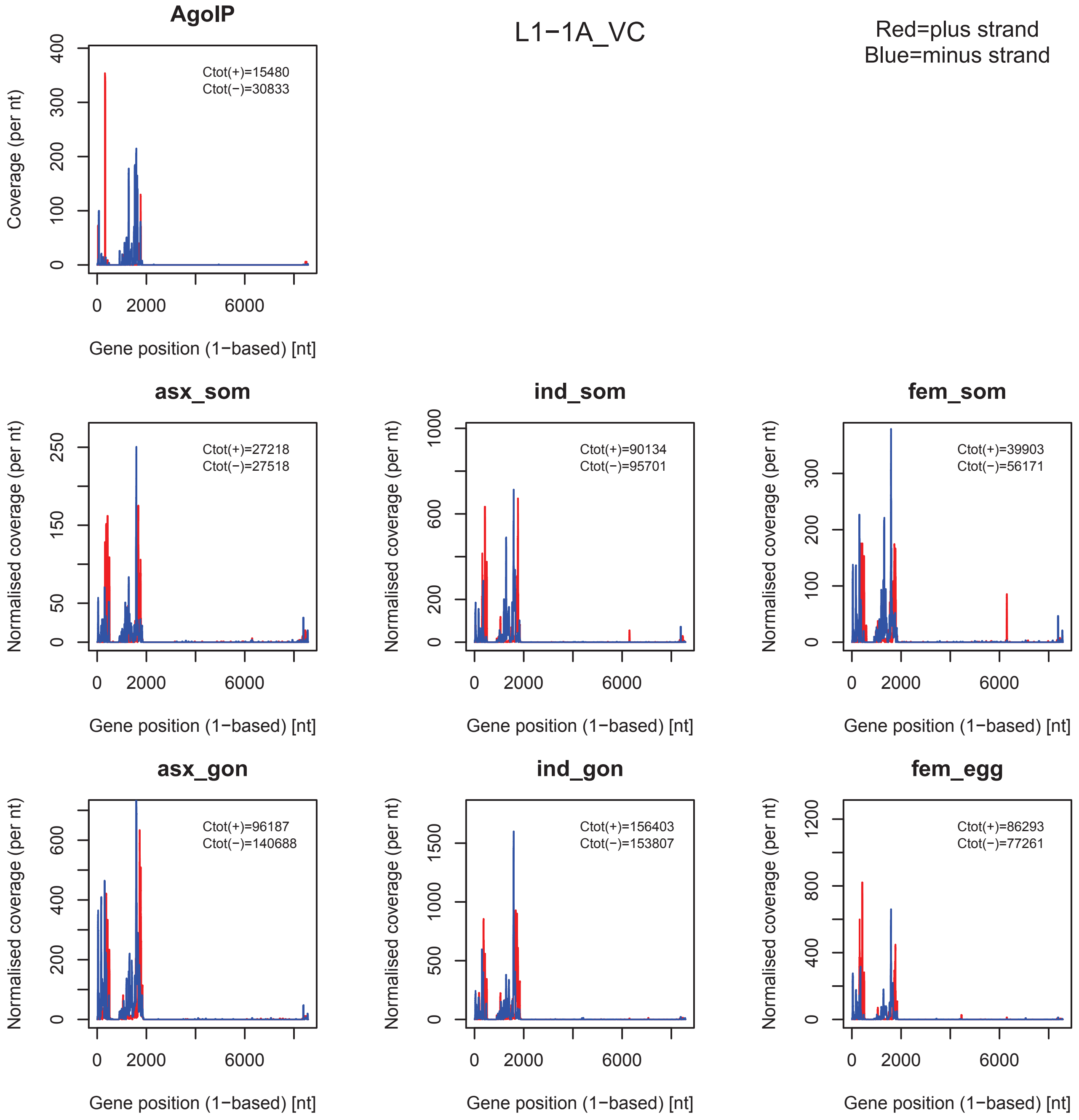
L1−1A_VC
Red=plus strand
Blue=minus strand
AgoIP
Ctot(+)=15480
Ctot(−)=30833
Coverage (per nt)
asx_som
Ctot(+)=27218
Ctot(−)=27518
ind_som
Ctot(+)=90134
Ctot(−)=95701
fem_som
Ctot(+)=39903
Ctot(−)=56171
asx_gon
Ctot(+)=96187
Ctot(−)=140688
ind_gon
Ctot(+)=156403
Ctot(−)=153807
fem_egg
Ctot(+)=86293
Ctot(−)=77261
Normalised coverage (per nt)
Gene position (1−based) [nt]

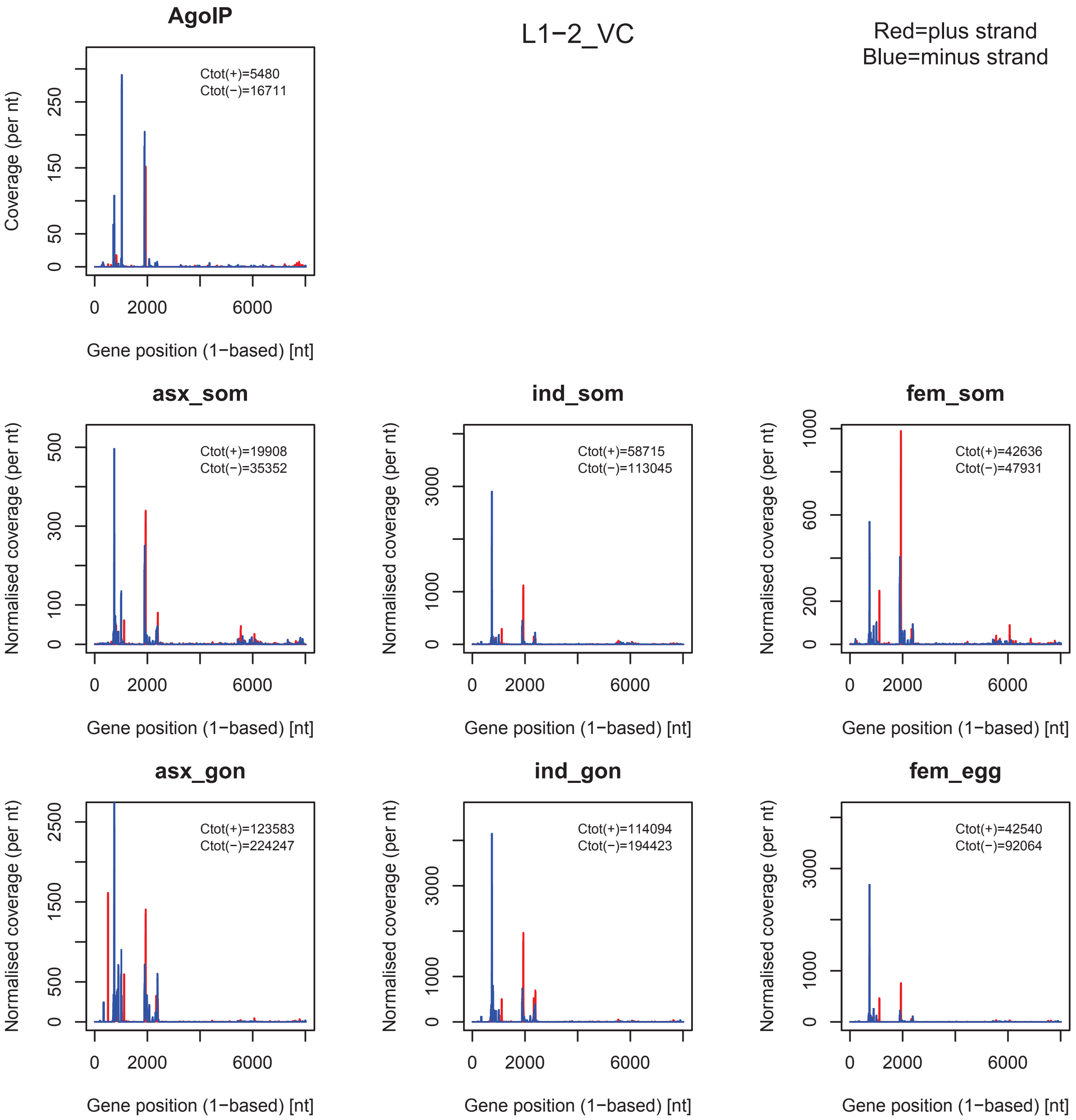
L1−2_VC
Red=plus strand
Blue=minus strand
AgoIP
Ctot(+)=5480
Ctot(−)=16711
Coverage (per nt)
0
50
150
250
0
2000
6000
Gene position (1−based) [nt]
asx_som
Ctot(+)=19908
Ctot(−)=35352
Normalised coverage (per nt)
0
100
300
500
0
2000
6000
Gene position (1−based) [nt]
ind_som
Ctot(+)=58715
Ctot(−)=113045
Normalised coverage (per nt)
0
1000
3000
0
2000
6000
Gene position (1−based) [nt]
fem_som
Ctot(+)=42636
Ctot(−)=47931
Normalised coverage (per nt)
0
200
600
1000
0
2000
6000
Gene position (1−based) [nt]
asx_gon
Ctot(+)=123583
Ctot(−)=224247
Normalised coverage (per nt)
0
500
1500
2500
0
2000
6000
Gene position (1−based) [nt]
ind_gon
Ctot(+)=114094
Ctot(−)=194423
Normalised coverage (per nt)
0
1000
3000
0
2000
6000
Gene position (1−based) [nt]
fem_egg
Ctot(+)=42540
Ctot(−)=92064
Normalised coverage (per nt)
0
1000
3000
0
2000
6000
Gene position (1−based) [nt]

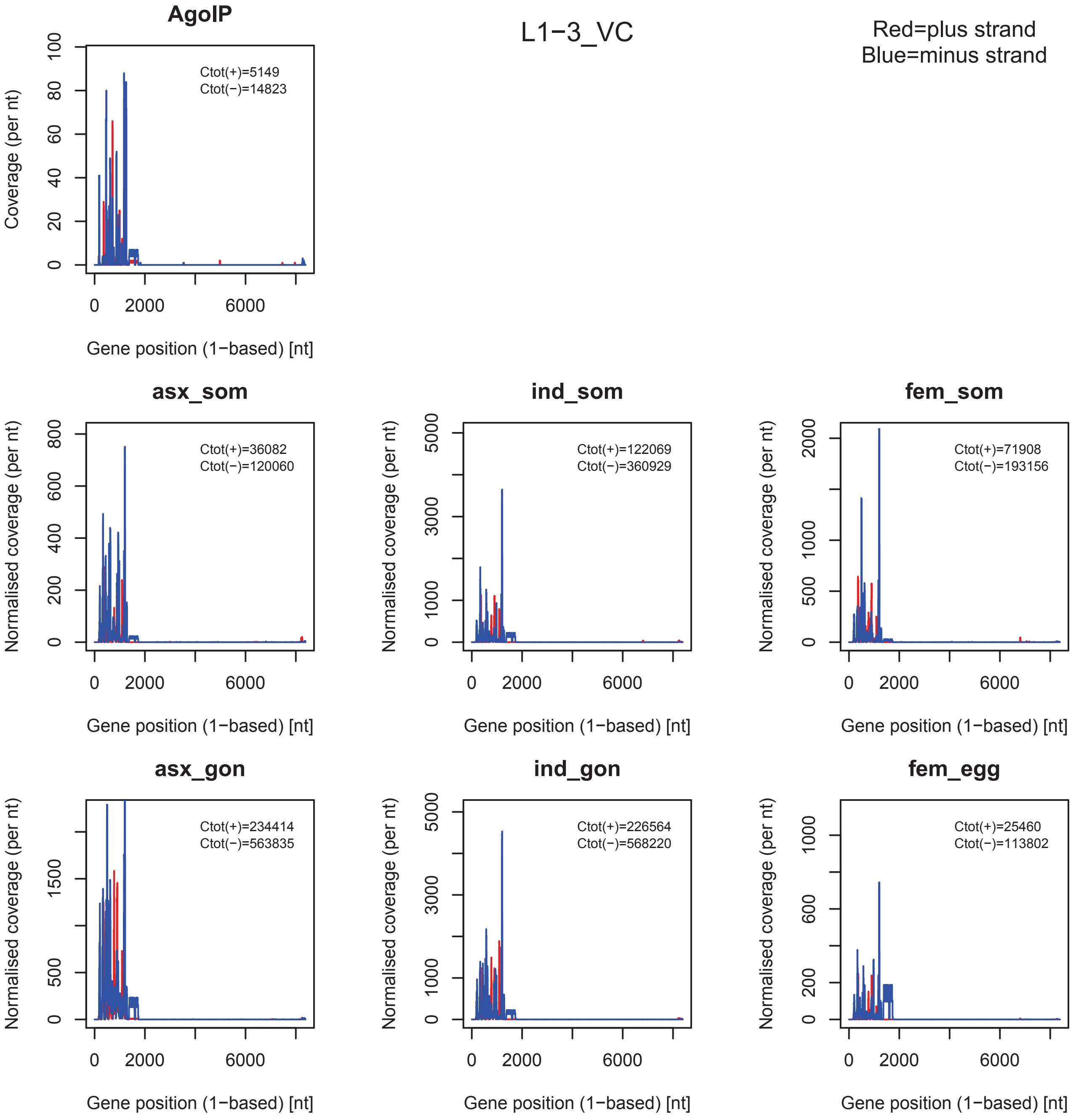

L1−3_VC
Red=plus strand
Blue=minus strand
AgoIP
Ctot(+)=5149
Ctot(−)=14823
Coverage (per nt)
0
20
40
60
80
100
0
2000
6000
Gene position (1−based) [nt]
asx_som
Ctot(+)=36082
Ctot(−)=120060
Normalised coverage (per nt)
0
200
400
600
800
ind_som
Ctot(+)=122069
Ctot(−)=360929
0
1000
3000
5000
fem_som
Ctot(+)=71908
Ctot(−)=193156
0
500
1000
2000
asx_gon
Ctot(+)=234414
Ctot(−)=563835
0
500
1500
ind_gon
Ctot(+)=226564
Ctot(−)=568220
0
1000
3000
5000
fem_egg
Ctot(+)=25460
Ctot(−)=113802
0
200
600
1000

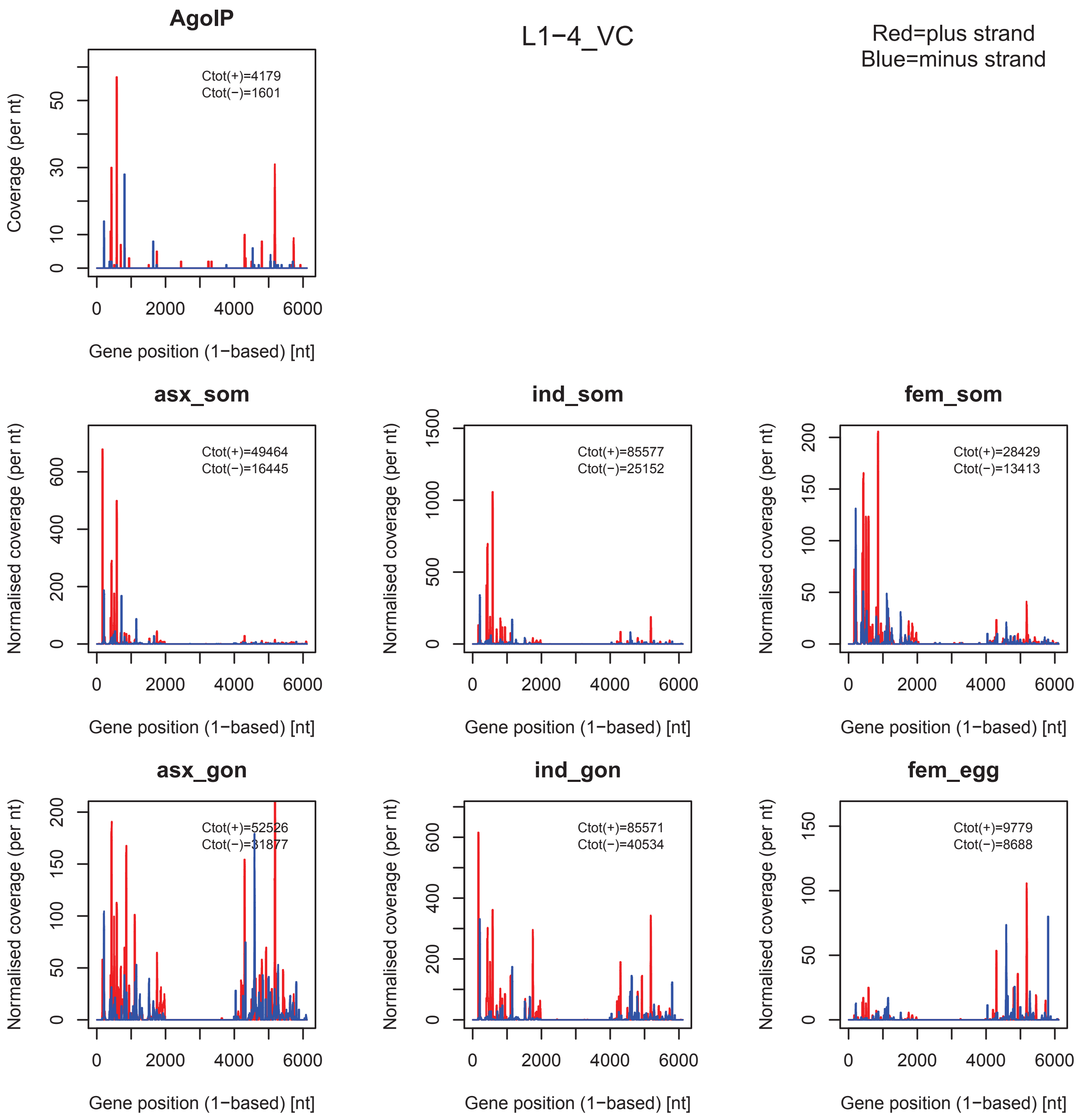

L1−4_VC
Red=plus strand
Blue=minus strand
AgoIP
Ctot(+)=4179
Ctot(−)=1601
Coverage (per nt)
Gene position (1−based) [nt]
asx_som
Ctot(+)=49464
Ctot(−)=16445
ind_som
Ctot(+)=85577
Ctot(−)=25152
fem_som
Ctot(+)=28429
Ctot(−)=13413
asx_gon
Ctot(+)=52526
Ctot(−)=31877
ind_gon
Ctot(+)=85571
Ctot(−)=40534
fem_egg
Ctot(+)=9779
Ctot(−)=8688
Normalised coverage (per nt)
Gene position (1−based) [nt]

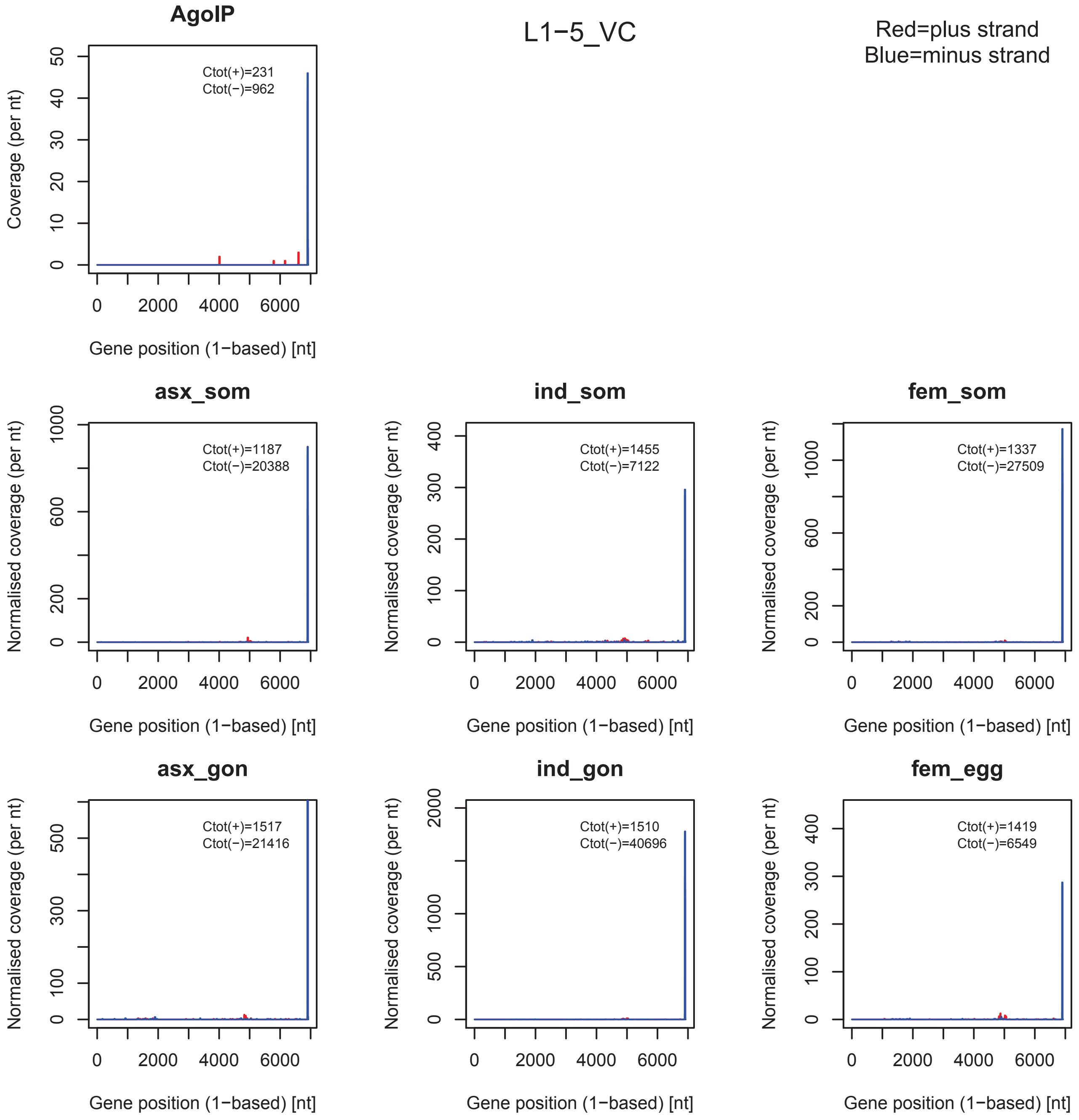
AgoIP
L1−5_VC
Red=plus strand
Blue=minus strand
Ctot(+)=231
Ctot(−)=962
Coverage (per nt)
Gene position (1−based) [nt]
asx_som
Ctot(+)=1187
Ctot(−)=20388
ind_som
Ctot(+)=1455
Ctot(−)=7122
fem_som
Ctot(+)=1337
Ctot(−)=27509
asx_gon
Ctot(+)=1517
Ctot(−)=21416
ind_gon
Ctot(+)=1510
Ctot(−)=40696
fem_egg
Ctot(+)=1419
Ctot(−)=6549
Normalised coverage (per nt)
Gene position (1−based) [nt]

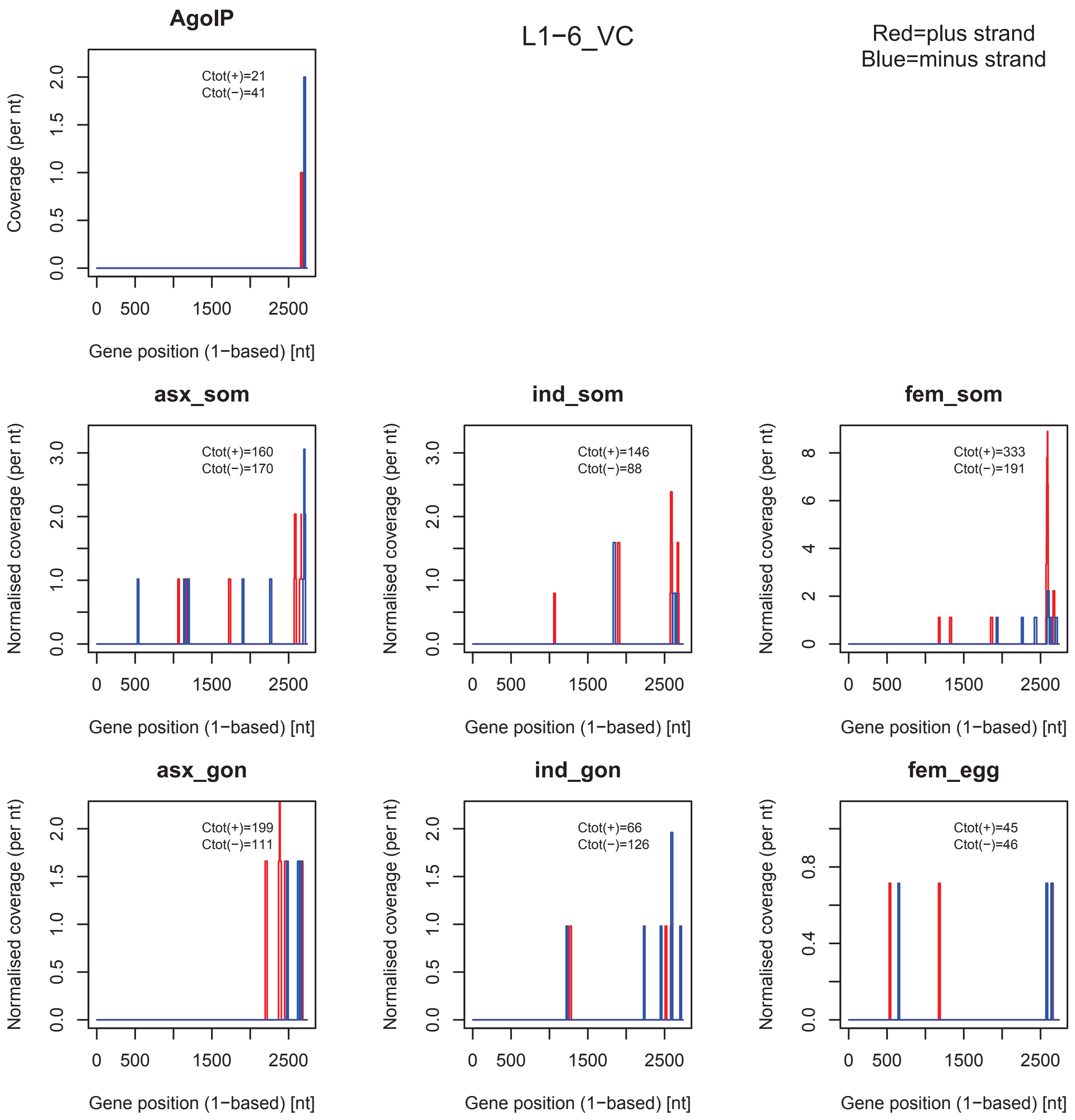
L1−6_VC
Red=plus strand
Blue=minus strand
AgoIP
Ctot(+)=21
Ctot(−)=41
Coverage (per nt)
asx_som
Ctot(+)=160
Ctot(−)=170
ind_som
Ctot(+)=146
Ctot(−)=88
fem_som
Ctot(+)=333
Ctot(−)=191
asx_gon
Ctot(+)=199
Ctot(−)=111
ind_gon
Ctot(+)=66
Ctot(−)=126
fem_egg
Ctot(+)=45
Ctot(−)=46
Normalised coverage (per nt)
Gene position (1−based) [nt]

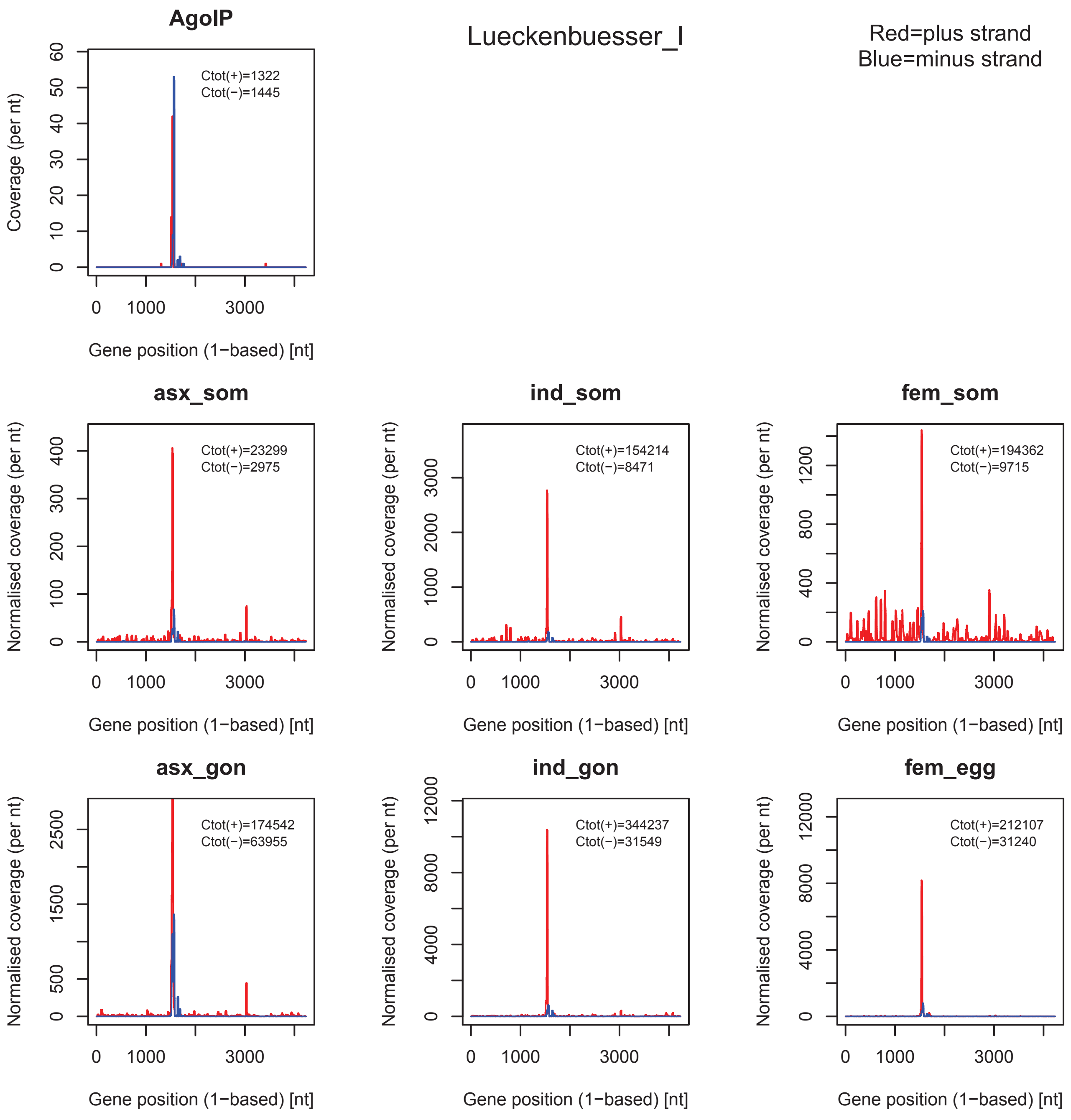

Lueckenbuesser_I
Red=plus strand
Blue=minus strand
AgoIP
Ctot(+)=1322
Ctot(−)=1445
Coverage (per nt)
asx_som
Ctot(+)=23299
Ctot(−)=2975
ind_som
Ctot(+)=154214
Ctot(−)=8471
fem_som
Ctot(+)=194362
Ctot(−)=9715
asx_gon
Ctot(+)=174542
Ctot(−)=63955
ind_gon
Ctot(+)=344237
Ctot(−)=31549
fem_egg
Ctot(+)=212107
Ctot(−)=31240
Normalised coverage (per nt)
Gene position (1−based) [nt]

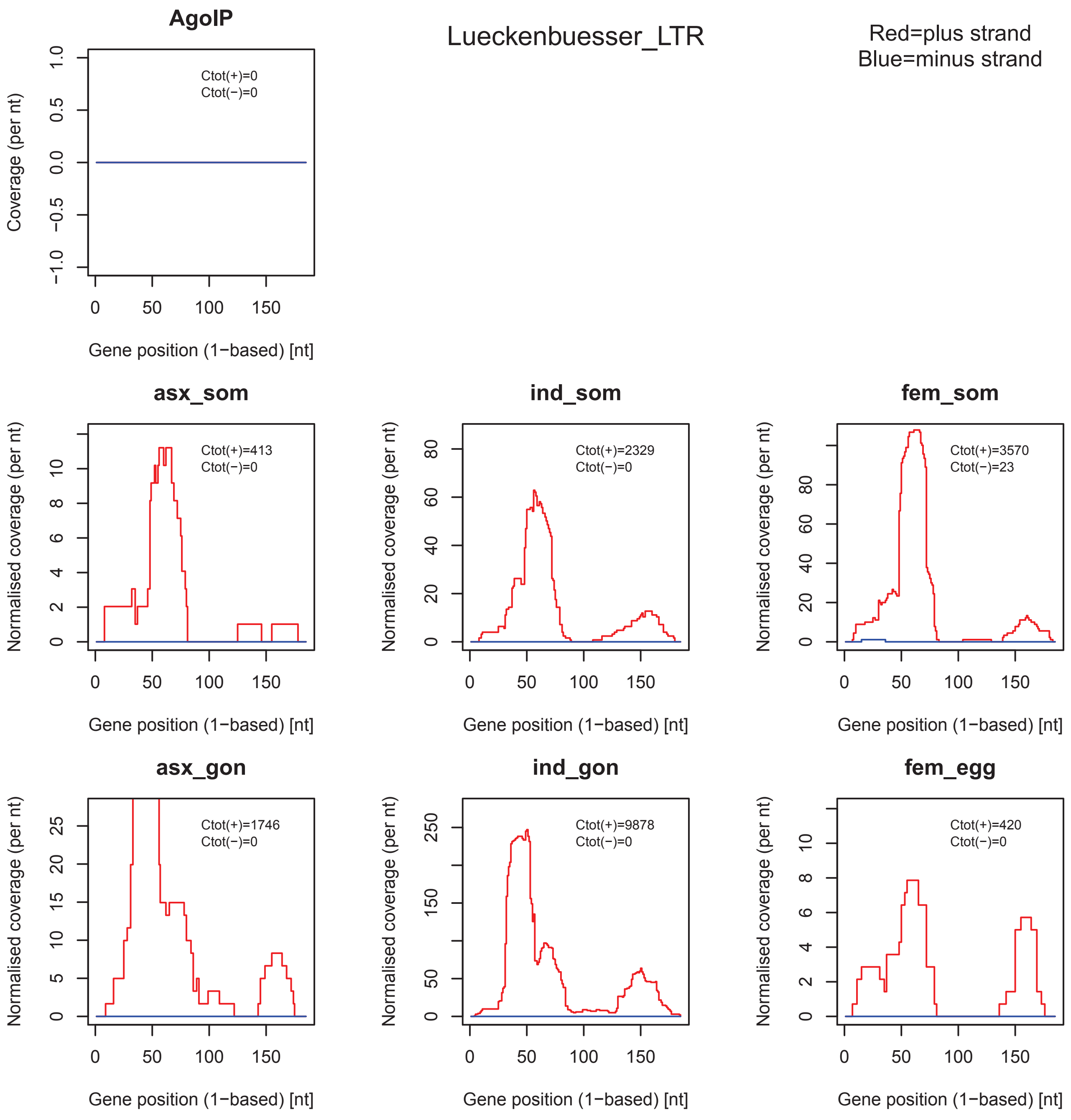
Lueckenbuesser_LTR
Red=plus strand
Blue=minus strand
AgoIP
Ctot(+)=0
Ctot(−)=0
Coverage (per nt)
asx_som
Ctot(+)=413
Ctot(−)=0
ind_som
Ctot(+)=2329
Ctot(−)=0
fem_som
Ctot(+)=3570
Ctot(−)=23
asx_gon
Ctot(+)=1746
Ctot(−)=0
ind_gon
Ctot(+)=9878
Ctot(−)=0
fem_egg
Ctot(+)=420
Ctot(−)=0
Normalised coverage (per nt)
Gene position (1−based) [nt]

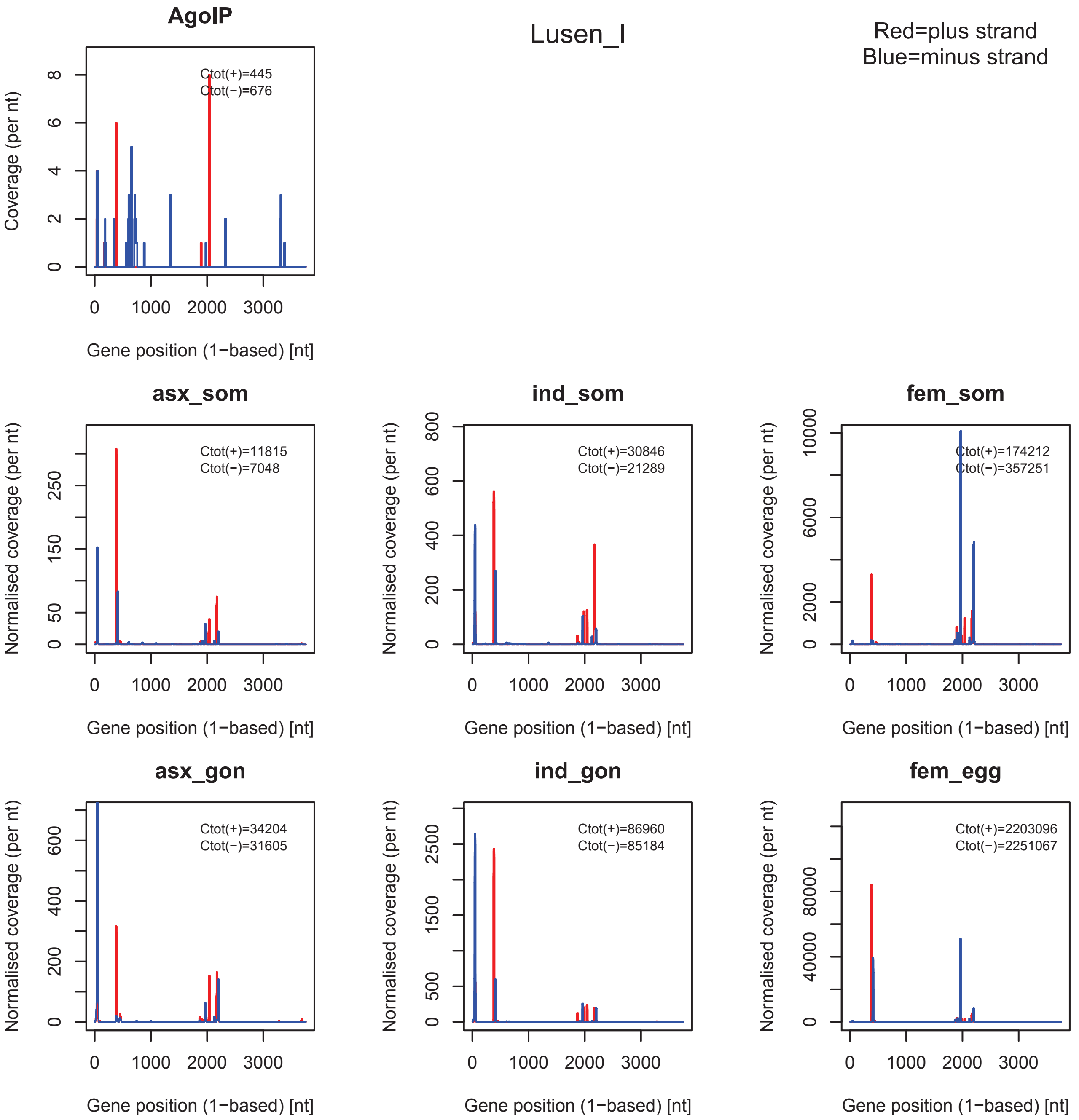

Lusen_I
Red=plus strand
Blue=minus strand
AgoIP
Ctot(+)=445
Ctot(−)=676
Coverage (per nt)
asx_som
Ctot(+)=11815
Ctot(−)=7048
ind_som
Ctot(+)=30846
Ctot(−)=21289
fem_som
Ctot(+)=174212
Ctot(−)=357251
asx_gon
Ctot(+)=34204
Ctot(−)=31605
ind_gon
Ctot(+)=86960
Ctot(−)=85184
fem_egg
Ctot(+)=2203096
Ctot(−)=2251067
Normalised coverage (per nt)
Gene position (1−based) [nt]

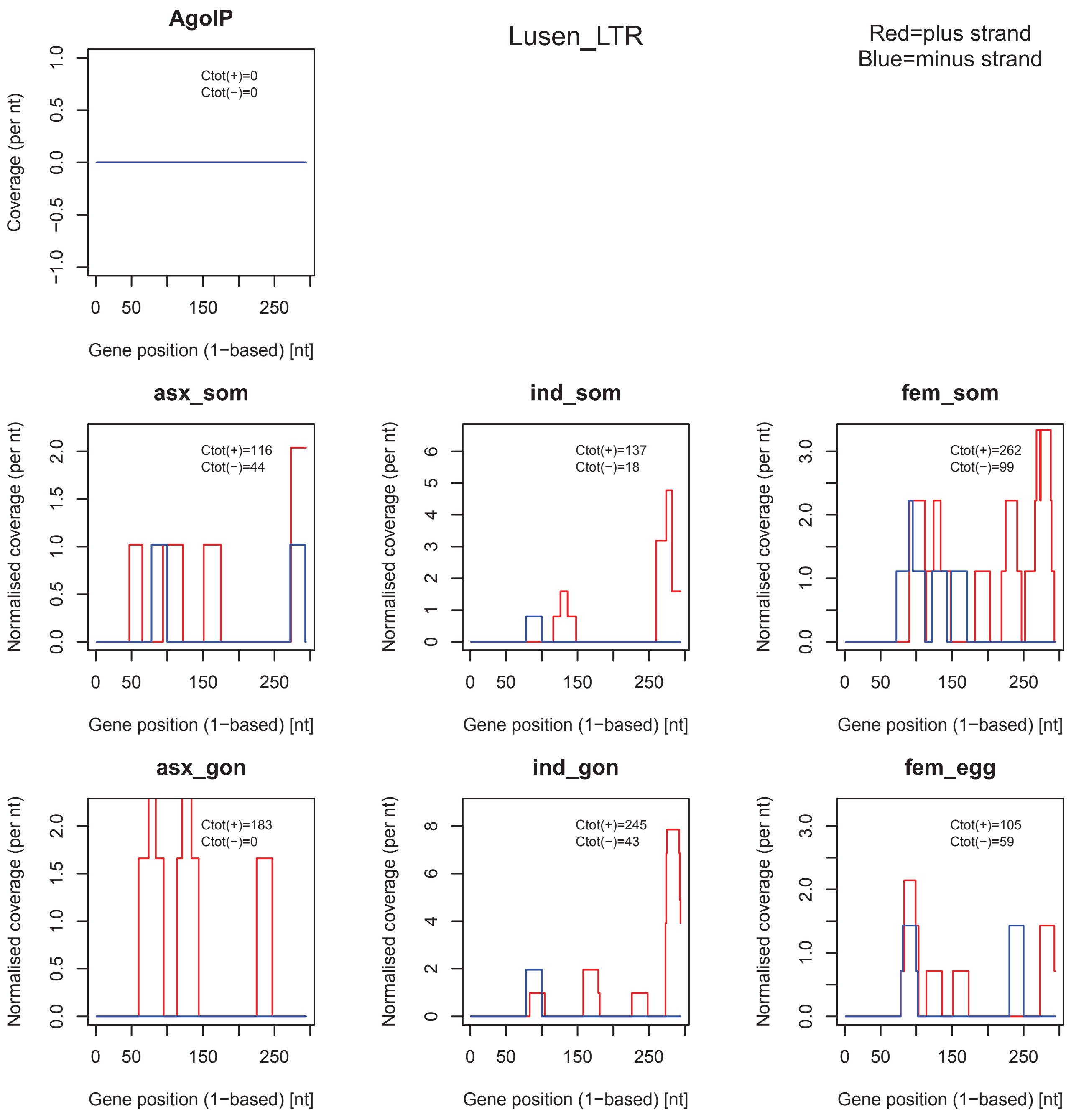
AgoIP
Lusen_LTR
Red=plus strand
Blue=minus strand
Ctot(+)=0
Ctot(−)=0
Coverage (per nt)
Gene position (1−based) [nt]
asx_som
Ctot(+)=116
Ctot(−)=44
ind_som
Ctot(+)=137
Ctot(−)=18
fem_som
Ctot(+)=262
Ctot(−)=99
asx_gon
Ctot(+)=183
Ctot(−)=0
ind_gon
Ctot(+)=245
Ctot(−)=43
fem_egg
Ctot(+)=105
Ctot(−)=59
Normalised coverage (per nt)
Gene position (1−based) [nt]

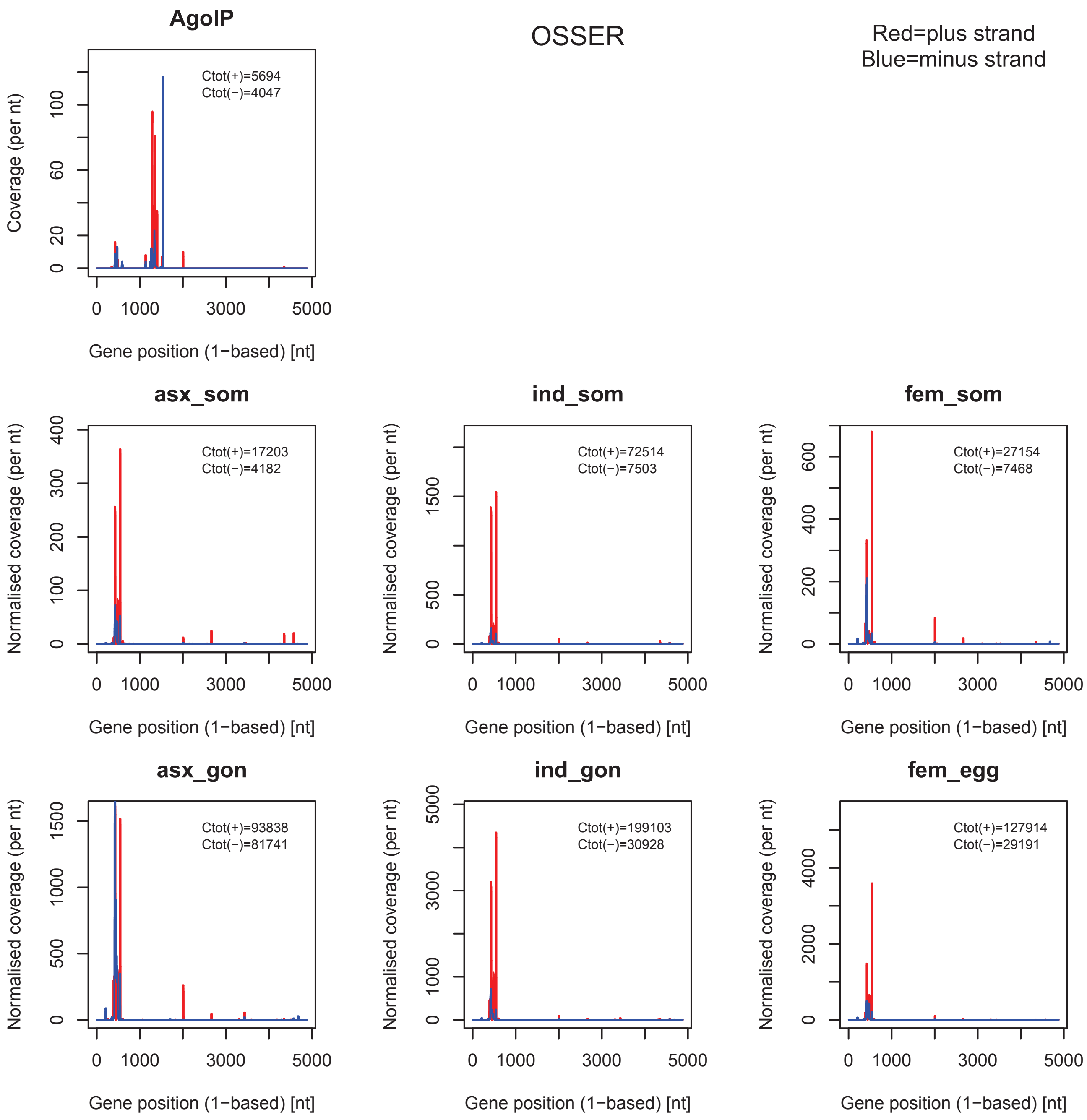

OSSER
Red=plus strand
Blue=minus strand
AgoIP
Ctot(+)=5694
Ctot(−)=4047
Coverage (per nt)
asx_som
Ctot(+)=17203
Ctot(−)=4182
ind_som
Ctot(+)=72514
Ctot(−)=7503
fem_som
Ctot(+)=27154
Ctot(−)=7468
asx_gon
Ctot(+)=93838
Ctot(−)=81741
ind_gon
Ctot(+)=199103
Ctot(−)=30928
fem_egg
Ctot(+)=127914
Ctot(−)=29191
Normalised coverage (per nt)
Gene position (1−based) [nt]

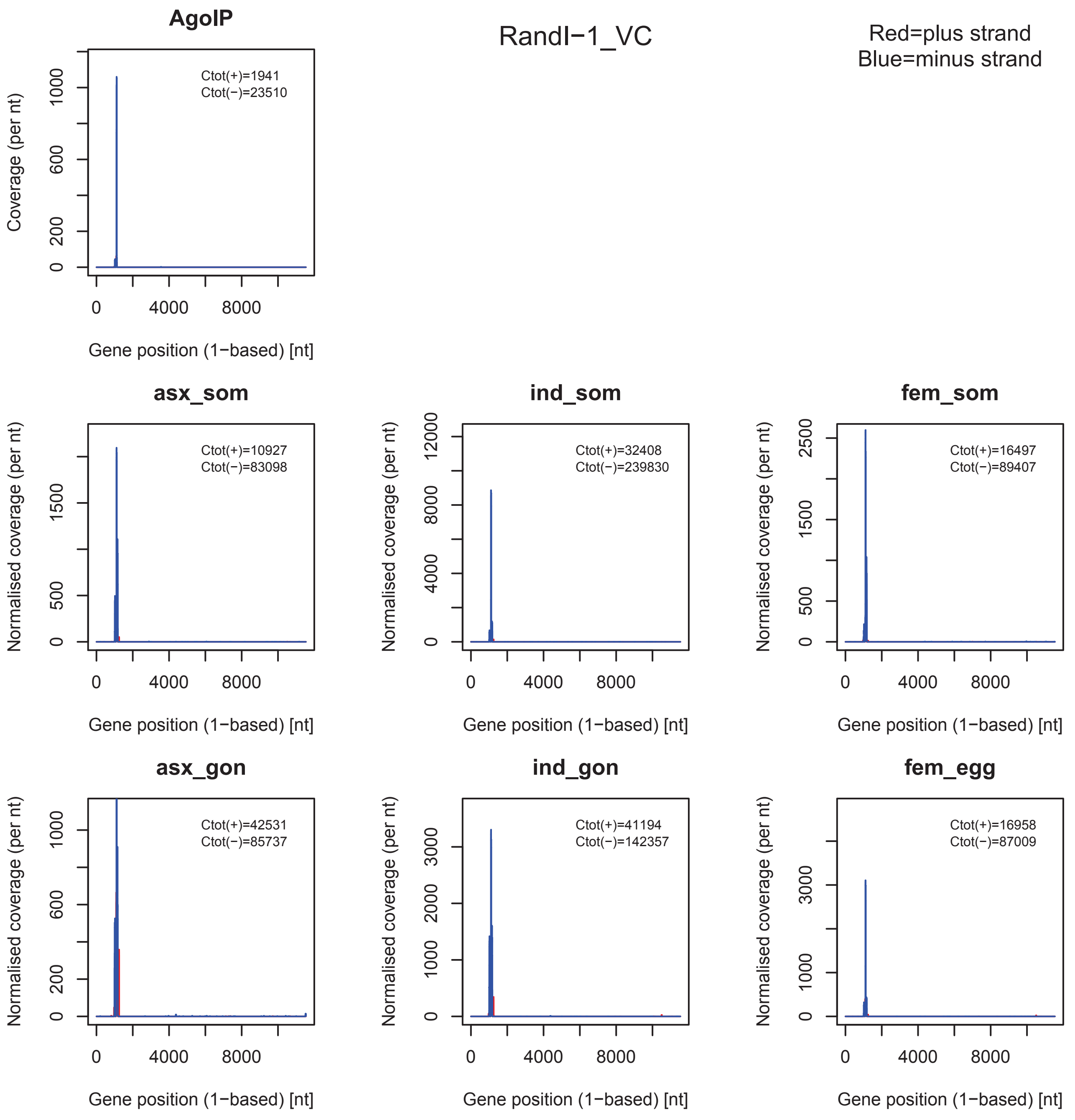
RandI−1_VC
Red=plus strand
Blue=minus strand
AgoIP
Ctot(+)=1941
Ctot(−)=23510
Coverage (per nt)
asx_som
Ctot(+)=10927
Ctot(−)=83098
ind_som
Ctot(+)=32408
Ctot(−)=239830
fem_som
Ctot(+)=16497
Ctot(−)=89407
asx_gon
Ctot(+)=42531
Ctot(−)=85737
ind_gon
Ctot(+)=41194
Ctot(−)=142357
fem_egg
Ctot(+)=16958
Ctot(−)=87009
Normalised coverage (per nt)
Gene position (1−based) [nt]

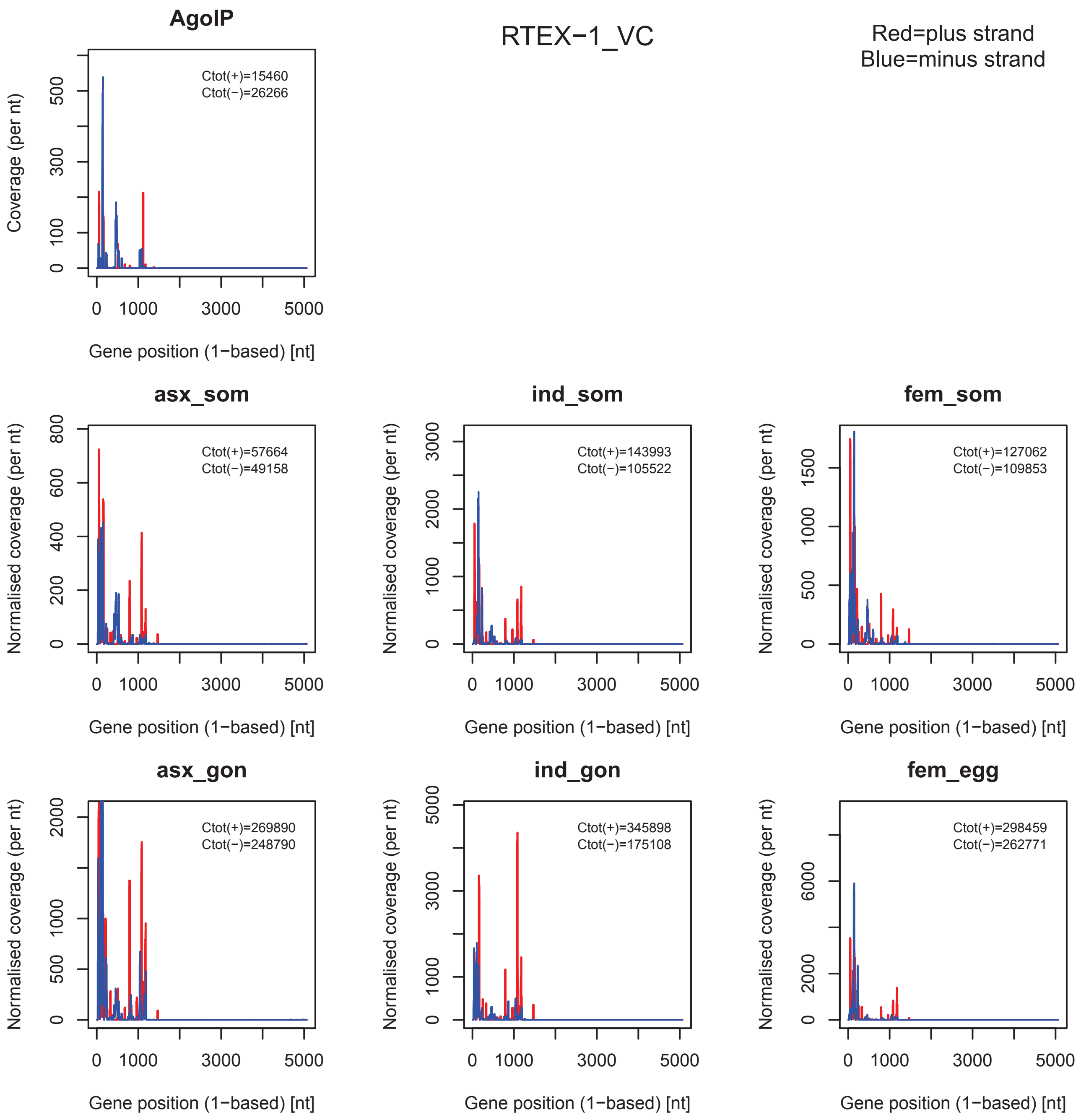
RTEX−1_VC
Red=plus strand
Blue=minus strand
AgoIP
Ctot(+)=15460
Ctot(−)=26266
Coverage (per nt)
0
100
300
500
0
1000
3000
5000
Gene position (1−based) [nt]
asx_som
Ctot(+)=57664
Ctot(−)=49158
Normalised coverage (per nt)
0
200
400
600
800
ind_som
Ctot(+)=143993
Ctot(−)=105522
0
1000
2000
3000
fem_som
Ctot(+)=127062
Ctot(−)=109853
0
500
1000
1500
asx_gon
Ctot(+)=269890
Ctot(−)=248790
0
500
1000
2000
ind_gon
Ctot(+)=345898
Ctot(−)=175108
0
1000
3000
5000
fem_egg
Ctot(+)=298459
Ctot(−)=262771
0
2000
6000

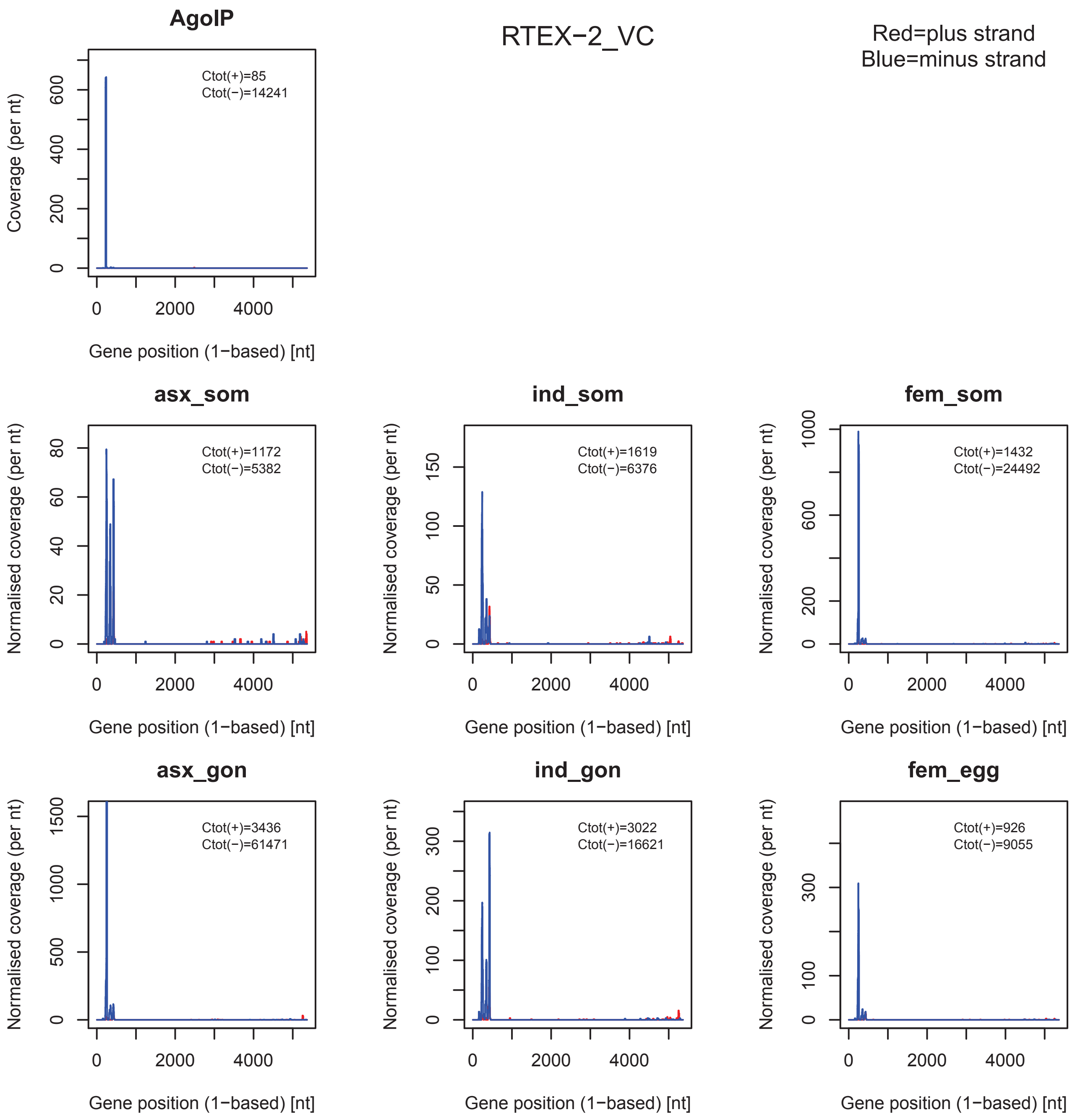

RTEX−2_VC
Red=plus strand
Blue=minus strand
AgoIP
Ctot(+)=85
Ctot(−)=14241
Coverage (per nt)
Gene position (1−based) [nt]
asx_som
Ctot(+)=1172
Ctot(−)=5382
ind_som
Ctot(+)=1619
Ctot(−)=6376
fem_som
Ctot(+)=1432
Ctot(−)=24492
asx_gon
Ctot(+)=3436
Ctot(−)=61471
ind_gon
Ctot(+)=3022
Ctot(−)=16621
fem_egg
Ctot(+)=926
Ctot(−)=9055
Normalised coverage (per nt)

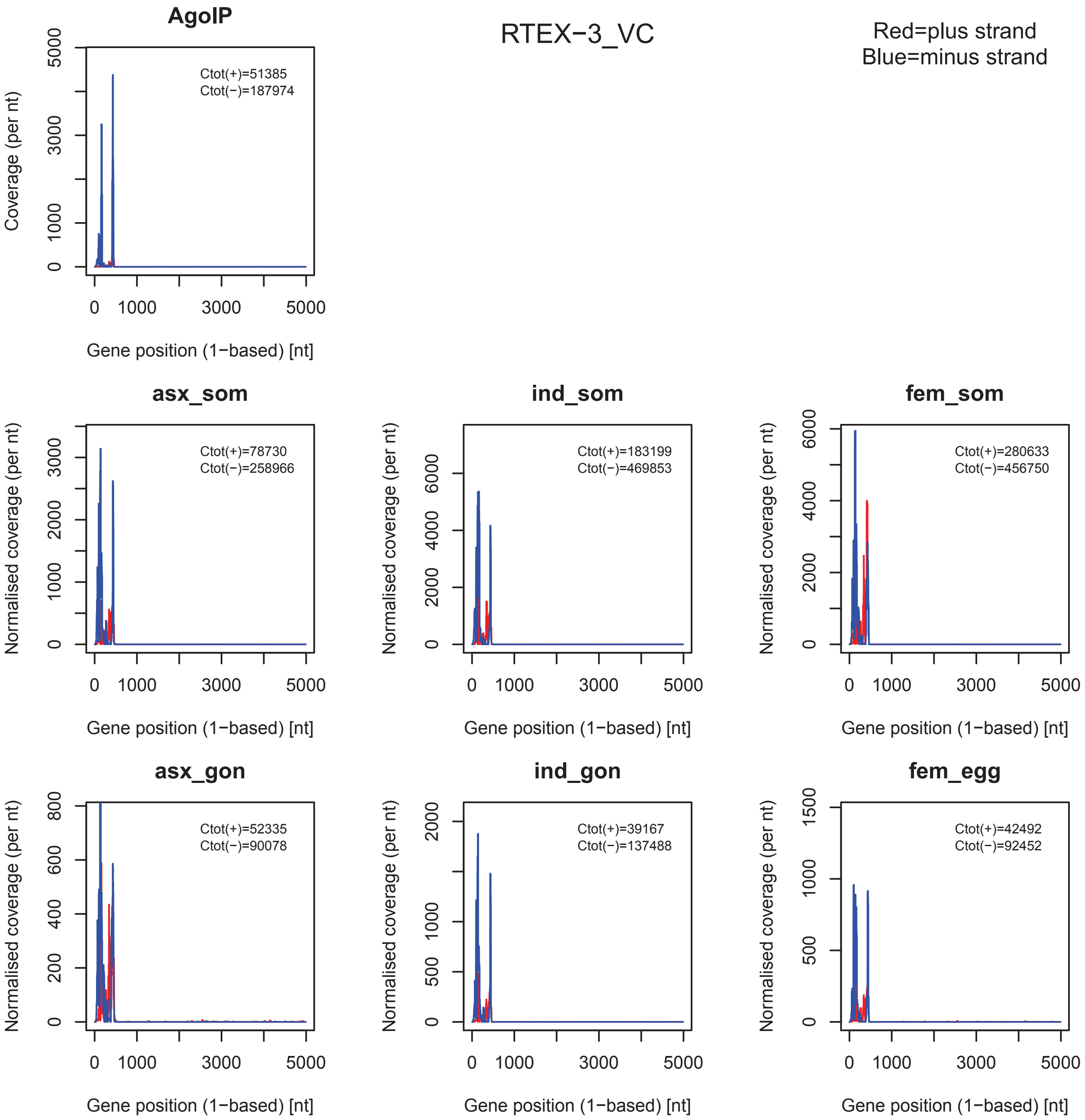
AgoIP
RTEX−3_VC
Red=plus strand
Blue=minus strand
Ctot(+)=51385
Ctot(−)=187974
Coverage (per nt)
Gene position (1−based) [nt]
asx_som
Ctot(+)=78730
Ctot(−)=258966
ind_som
Ctot(+)=183199
Ctot(−)=469853
fem_som
Ctot(+)=280633
Ctot(−)=456750
asx_gon
Ctot(+)=52335
Ctot(−)=90078
ind_gon
Ctot(+)=39167
Ctot(−)=137488
fem_egg
Ctot(+)=42492
Ctot(−)=92452
Normalised coverage (per nt)
Gene position (1−based) [nt]

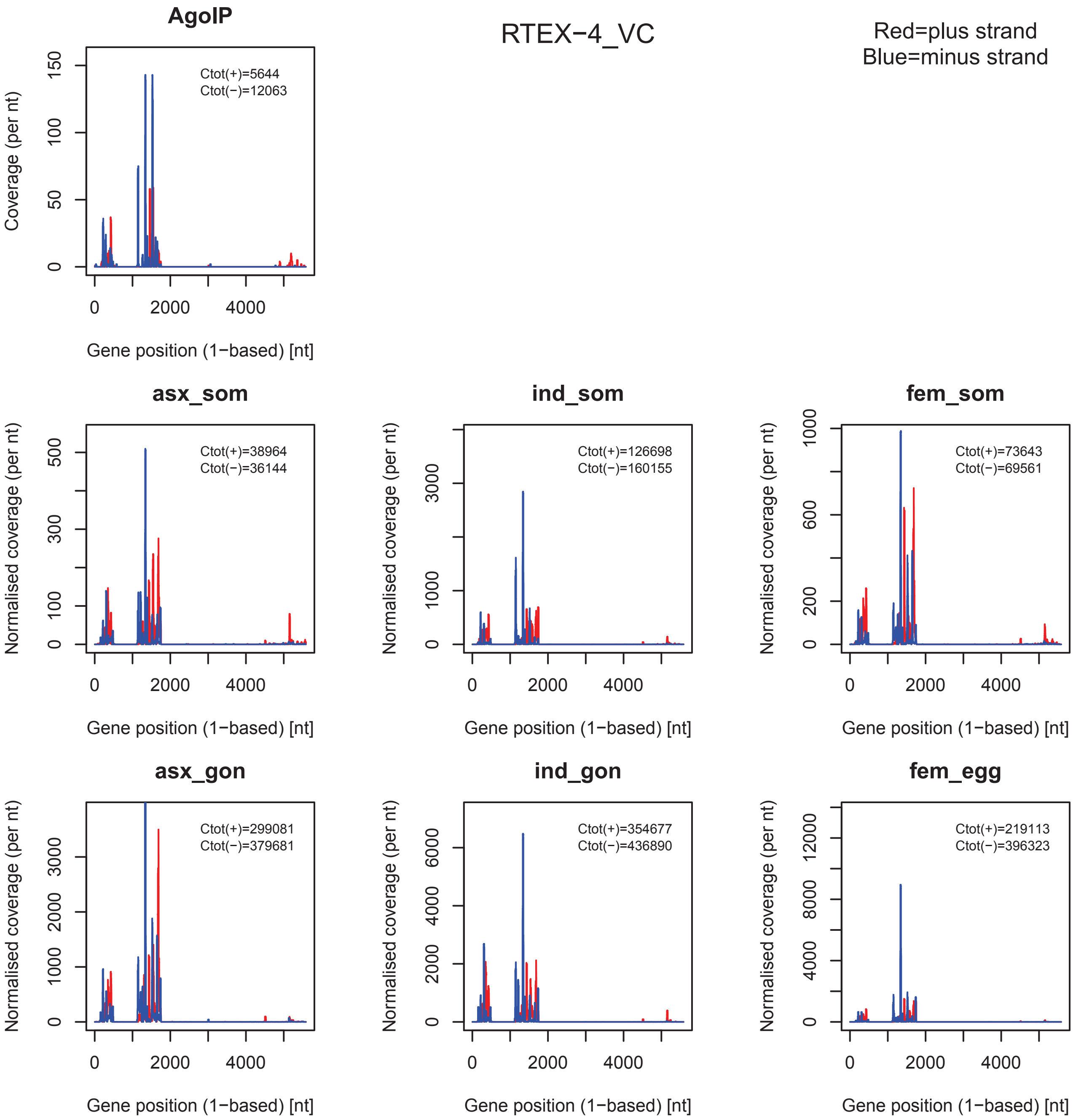
RTEX−4_VC
Red=plus strand
Blue=minus strand
AgoIP
Ctot(+)=5644
Ctot(−)=12063
Coverage (per nt)
0
50
100
150
asx_som
Ctot(+)=38964
Ctot(−)=36144
0
100
300
500
ind_som
Ctot(+)=126698
Ctot(−)=160155
0
1000
3000
fem_som
Ctot(+)=73643
Ctot(−)=69561
0
200
600
1000
asx_gon
Ctot(+)=299081
Ctot(−)=379681
0
1000
2000
3000
ind_gon
Ctot(+)=354677
Ctot(−)=436890
0
2000
4000
6000
fem_egg
Ctot(+)=219113
Ctot(−)=396323
0
4000
8000
12000
Normalised coverage (per nt)
0
2000
4000
Gene position (1−based) [nt]

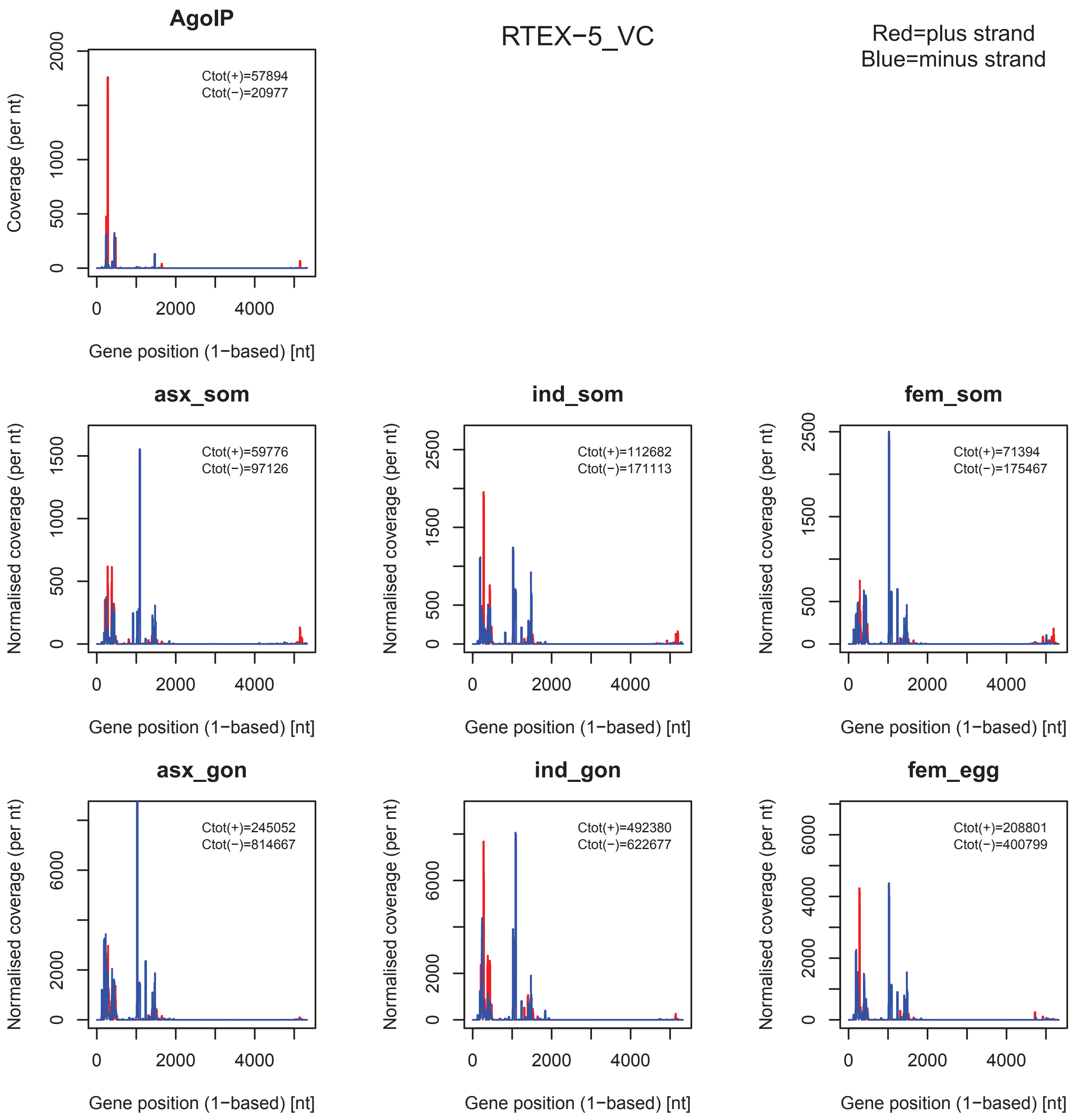
RTEX−5_VC
Red=plus strand
Blue=minus strand
AgoIP
Ctot(+)=57894
Ctot(−)=20977
Coverage (per nt)
asx_som
Ctot(+)=59776
Ctot(−)=97126
ind_som
Ctot(+)=112682
Ctot(−)=171113
fem_som
Ctot(+)=71394
Ctot(−)=175467
asx_gon
Ctot(+)=245052
Ctot(−)=814667
ind_gon
Ctot(+)=492380
Ctot(−)=622677
fem_egg
Ctot(+)=208801
Ctot(−)=400799
Normalised coverage (per nt)
Gene position (1−based) [nt]
